# Supplementary material for: Rurality representation and changes in rural tourism destination
Source: PLoS One. 2026 Apr 21;21(4):e0347226. doi: 10.1371/journal.pone.0347226 (PMC13098982; doi:10.1371/journal.pone.0347226)
Supplement: S1 File — (ZIP) [file pone.0347226.s001.zip › supporting information/世凹村录音及转译文本/jsa-5.docx]

Q: Now, you know this village is developing tourism. Are you familiar with the situation here?

A: JM: Not bad.

Q: How has the village's tourism development been in the last couple of years?

A: JM: Development started around 2014. The changes have been huge. Before, it was just a small village. Now it's been developed very well, turned into a tourist spot.

Q: What is the biggest change here?

A: JM: The biggest change... anyway, it feels like before there were no餐饮 facilities. After tourism, more people came to have fun, so roughly... people started餐饮 businesses. Those that couldn't manage it conveniently... (trails off)

But is the environment really better than before?

JM: That's definitely better.

Q: What about the houses?

A: JM: The houses are our own families', but the exteriors were renovated by the government.

Q: What do you think about the exterior renovations done by the government?

A: JM: The renovations... they still have to be separate, right? The style they built is called... what style...

Do you think it looks good?

JM: Better than our old farmer houses. Rural houses... just 1000平米 standing there for so many years.

Q: I also heard from others that the number of tourists hasn't been as good as in previous years recently.

A: JM: Now there are too many rural tourism villages. This is the reason. Now every place is doing this 'Beautiful Village' thing. So the number of people here is naturally relatively smaller. At the very beginning, it was packed with people, really good. Started getting popular from 2014, and development began in 2012. The people who came here were indeed numerous. You know, every place, every floor needs cleaning.

Q: Do you think our place has distinctive features compared to other rural villages? I see we mostly do agritainment here, the food is delicious.

A: JM: But actually, cusine isn't like the taste in hotels. It's just normal home-style cooking.

Q: What do you think is the biggest attraction of agritainment for outsiders? What brings them here?

A: JM: The famous cusine.

Q: Well-cooked dishes or cusine, right? And then, you mentioned the rural environment and atmosphere here, is that a major attracting factor for city people?

A: JM: The air is good, it's comfortable!

Q: Do you think the current rural environment here is very different from your childhood memories?

A: JM: Bungalows without multiple stories... everything is better now, regardless, like reinforcement, flowers... Before, the farm had dirt buildings, no water... just dirt buildings.

Q: Before development here, couldn't you do this work?

A: JM: Before development, this small mountain village didn't have... you don't need anything, no cleaning services or anything. Now, with this development, you need to ensure sanitation.

Right, what did you do before development?

JM: We farmed. If we didn't farm, we looked for work outside.

Q: Now with development, the rented land was all taken?

A: JM: They invested directly.

Q: So now you have no land either, just focus more on work here. Do you think life was better farming or now?

A: JM: Of course now! Farming life, income was lower. Now, working, you can earn two or three thousand yuan a month. Before, you worked yourself to death, how much income did you have? And you were exhausted.

Q: How much did you earn a year?

A: JM: Not much income. Now life is happier. Just 20,000, 30,000. And leisure time is your own.

Q: So now, when there's nothing to do, we can sing, dance. Before tourism, when we were farming, what leisure activities did you have?

A: JM: Playing cards at home, that's it. There was no square dancing. Now you can square dance. Because before, working the fields at home, you were dead tired,哪有功夫出来跳舞? Who danced? Now people are extremely happy. Tell the elderly, now you are really extremely happy compared to anyone.

Q: But now more tourists come, do we interact with them much?

A: JM: If they don't talk to us, how do we talk to them?

Q: What is your attitude towards them?

A: JM: Normal. For example, if they ask us something, we answer normally.

Q: What impact do you think these tourists have brought you?

A: JM: When they come, to be honest, of course it's less sanitary, definitely dirtier.

JM: Telling the truth. But we hope they come. Three people also have their time... you can't just tell them not to come, keep them out... then they come, look after the elderly... it increases our income, but also brings these environmental problems.

Q: Do you think their arrival, their lifestyles, have had any impact on the local farmers, the original residents?

A: JM: No major differences. Everything is a bit better. Renovations compared, household appliances are better.

Q: Now that our income has increased, has our lifestyle become more modern?

A: I JM: think... now we are urban registered residents. I just think... do you feel the rural feeling of being from the countryside has faded a lot?

JM: I think the things from childhood back then probably don't exist now. Like when we were kids, from our generation onwards... things in the rural areas before, we fundamentally understood everything, right?

Fundamentally don't understand now.

JM: Now we don't use those things anymore either. Like those farm tools from before, would you recognize them now? Someone your age wouldn't know.

Q: Do you sometimes miss the old life? It was quieter before, now with more people it's a bit annoying, and more and more strangers.

A: JM: Everyone has their own opinion.

Q: What is your opinion?

A: JM: My thinking is that it's getting better and better. Before, you were stuck in the fields, meaning one step and you were in mud. You tell me, was that good? Right?

Right. Do you think neighborly relationships have changed compared to before? Seems very good now too. But for those running agritainment, is there some competition between them, affecting the relationship between two families?

JM: Should be relatively minor. It's impossible otherwise. For example, someone runs a business, your family has more customers, my family has fewer, there's somewhat of an impact. Conversely, if a guest asks me, I can't tell them to go to your place, right?

Q: Have any relatively major problems emerged with tourism development here, or negative aspects? You mentioned before, people used to eat holding their bowls, visiting neighbors, it was very comfortable, right? Now it's different. More people, worse environment.

A: JM: Before, did this village have any festive activities, like dragon/lion dances, or gatherings?

We in this rural area, no, very few.

JM: Many places still have dragon and lion dances. Temple fairs existed before, now they're gone.

No, can't, don't have. What activities did the temple fair mainly involve?

JM: The temple fair was like a market, we just called it a temple fair. They picked a specific day every year, came once every year.

Q: Did every household bring things to sell?

A: JM: Local specialities, whatever... all the food, drinks, daily items,有理, were all brought to the market for exchange, right?

Q: Now you all shop by yourselves? Like the things in your household?

A: JM: Yes, now we all go buy ourselves.

Q: Including your family's diet, have there been any changes compared to before?

A: JM: The food we eat is better than before. But before we might often eat wild vegetables. Are there still wild vegetables like before?

JM: Before, wild vegetables, nobody ate them. Now people specifically seek out wild vegetables to eat. Before, anyway, if they had no money to buy [other food], they could only go for these wild vegetables. Now their conditions are better, they actively seek out wild vegetables to eat. They eat too much meat, so it didn't taste good back then, now they make it and eat wild vegetables. Before, if you wanted to eat well, you had no money to buy. What season to eat which wild vegetable, people look for them.

JM: We, in the past, the locals didn't eat them. Gave them to the pigs to eat, gave them to the pigs. We only came here when development started in 2012, arrived on July 25th, and have been doing this work ever since.

Q: This work should be relatively easier.

A: JM: Still need more business during holidays. Usually there are fewer people. Basic share, then working overtime inside during holidays.

Q: Normally, who are you employed by?

A: JM: Employed by the subdistrict office, not the village committee.

Q: Also, may I ask how old you are now?

A: JM: First time retiring? 53.

Q: What do your children do now?

A: JM: Work, work in a unit. None have come back. Our child works right nearby.

Q: Mhm. Now, are there many young people in this village?

A: JM: Now, the old houses are being developed. Some of them, all the old houses, they've moved to residential compounds. The young people are all outside. Not many young people.

Q: Compared to before, the roads here are better. What impact has that had?

A: JM: Transportation is more developed. More tourists come.

Q: But the rural areas before were probably dirt roads, now there are many cement roads. Do you think these roads have changed the traditional rural character? What changes do you think should be accepted?

A: Transportation is more convenient. Travel is definitely easier. Otherwise, when it rained, cars couldn't get in.

JM: Because cars... before, basically no family had a car. Now every family has a car, several per family. Everyone has a car. Everyone's income should be higher anyway, right?

Q: You think that rural tourism generally uses rural characteristics to attract city people. If our village wants to continue developing tourism successfully in the future, should it continue to highlight its rural characteristics?

A: JM: If you want to develop tourism, it definitely needs distinctive features. Without distinctive features, it won't work. Like, people coming to play ask us which family has which special dishes. The shop owners also want to find special features, they also want to innovate. Don't they want to find special features to attract tourists?

Q: Are there things here, as we understand, some city people might want to bring their children for fruit picking or farming experience activities? Do we have that here?

A: JM: Before, the peach orchard had it. Originally there was a piece of land belonging to their village community. Before that piece was the community's, now it belongs to Niushou Mountain. Right, the peach orchard area was divided into plots. Later, you bring a child, you pay a certain amount for a plot, you come regularly to plant, come to harvest when it's time, it all existed. It was like leasing a plot of land. You come on the weekend when free, work on it, then after a couple of weeks, it seems after coming, the newly ripe peaches can be eaten, right? They did that back then, the first couple of years. I thought this model was really good.

Back then they also set up a small vegetable garden.

Q: You think... meaning, now many tourists yearn for rural life. But in your ideal, JM, what are the most important aspects of rural life that should distinguish it from the city?

A: The key thing to solve is the farmland, farming the land, planting.

Q: What about the food aspect?

A: JM: City people eat Western food. Rural people eat cusine.

Q: And then some rural landscapes. Mainly it should be these aspects that highlight the differences between rural and urban areas, right? Okay, thank you. Our questions are finished here.
