# Supplementary material for: Rurality representation and changes in rural tourism destination
Source: PLoS One. 2026 Apr 21;21(4):e0347226. doi: 10.1371/journal.pone.0347226 (PMC13098982; doi:10.1371/journal.pone.0347226)
Supplement: S1 File — (ZIP) [file pone.0347226.s001.zip › supporting information/世凹村录音及转译文本/jsa-6.docx]

Q: Do you run a farmhouse inn at home?

A: Yes, I do

Q: It’s great that you have a farmhouse inn and also work as a security guard here.

A: Well, no rush.

Q: How has it been this year?

A: This year is quite special. It’s been affected by the pandemic.

Q: So when did you start the farmhouse inn?

A: I’ve been running it for a few years, about 3 to 4 years.

Q: Where is your home located?

A: Over there.

Q: How has your farmhouse inn been doing these years?

A: Not good. To be honest, there’s no money. Prices are so high now. Look, the bowl of noodles I just had cost 20 yuan—prices have gone up so fast.

Q: Yes, prices have risen sharply in recent years. Do you think the village has developed well these years? Has the environment improved compared to before, after developing tourism?

A: The environment is much better than before, way better.

Q: Do you still farm at home?

A: No farming, just growing vegetables. We still grow our own vegetables.

Q: So for the dishes at the farmhouse inns here, some are home-grown and some are bought, right? How do you think the environmental atmosphere of the village is for the farmhouse inns?

A: That’s just how it is. "Take from the people and use for the people"—don’t laugh at me for saying that.

Q: It’s okay. We’re students doing research here, so we want to learn more.

A: "Take from the people and use for the people"—that’s just it. To be honest, I think one must strive. Deng Xiaoping said, "Whether it's a white cat or a black cat, a cat that catches mice is a good cat." No matter how knowledgeable or educated you are, you can’t do anything without connections. That’s the reality of society. I’m telling you the truth. I joined the Party in 1982 and served for 6 years. I’m an old Party member. It’s all about per capita income.

Q: Who do you think usually comes to the farmhouse inns?

A: Originally, people from all over the country would come, but it’s all affected by the pandemic.

Q: What do you think attracts tourists here?

A: Mainly Niushou Mountain—it’s a great place.

Q: Are there also tourists who come because of the farm dishes and the rural experience? Do many urban people come for that?

A: We used to cook with big iron woks, but now we use gas stoves. The prices are fixed, and we have regular customers. To be honest, we use vegetables from our own garden.

Q: Do you think the village appearance has improved a lot after developing tourism? Were the houses renovated uniformly on the outside?

A: The houses—old houses—their exteriors were renovated uniformly by the government.

Q: Do you think these renovated houses make a difference? It seems the interior is still the same, only the exterior has changed, right?

A: It’s better after renovation, but to be honest, our pockets are still empty. I’m telling you the truth. An 80-year-old lady—even at 80, if she has fields at home, her sons can give her some rice and oil. That’s practical, something to eat. Filial sons will give you money; unfilial ones—you eat if you want, don’t if you don’t. I’m being honest.

Q: So the tourism development here was approved by the village committee?

A: No, it was the government—done by the government.

Q: Are there many outsiders in the village?

A: Yes. Now with tourism development, people with money can afford to travel. They go around the country. Outsiders come to Nanjing, and Nanjing people go to other places. It’s complementary.

Q: I mean, are there any outsiders running farmhouse inns here?

A: No.

Q: Are they all run by locals? Do any outsiders rent houses here to run them?

A: They can’t make it. How can they with the prices?

Q: Do you think the relationship between neighbors in the village has changed since developing tourism? Did people used to visit each other and chat more often before?

A: Not anymore. To be honest, if you come to my house and I don’t offer you cigarettes, it’s not nice. But if I do, it’s an expense. I’ll stop here.

Q: How old are you this year?

A: I’m 27.I came back from the army in my twenties, then settled locally. I always help when I should.

Q: I heard there used to be dragon dances here before. Have you seen them?

A: No, I haven’t.

Q: So you haven’t seen them. Are there any special folk customs or village collective activities during festivals now?

A: No, none. There used to be temple fairs—everyone would go shopping there. Now what’s there to buy? Mostly medicines. People beg for money, saying "Please help." But it’s not good. People go to the city to buy famous brands—there’s so much comparison. To be honest, without money, life is hard.

Q: That’s true.

A: You said you’re a student—27, so you’re in university?

Q: I’m a graduate student now.

A: Where’s your hometown?

Q: Anhui.

A: Where in Anhui?

Q: Anqing.

A: Anqing. That place is quite poor. When I was 27, I came here. To be honest, interpersonal skills are very important here.

Q: When was this road built?

A: Three or four years ago.

Q: Do you think the road has brought any changes to the village? Has it made it easier for tourists to come?

A: Of course it’s more convenient. You’re not a local, so you wouldn’t know otherwise.

Q: We want to know the real situation.

A: The real situation is—people used to be really honest, but now even if people have money, things aren’t the same. To be honest, in the past, people learned from Lei Feng and did good deeds—that was the Mao Zedong era. Now no one talks about that anymore. It’s hard to do good deeds these days, isn’t it? Am I right?

Q: Do you think your lifestyle has changed compared to before?

A: How to put it? For example, I earn 2,200 yuan a month. I spend 1,000 yuan on food, leaving 1,200 yuan. That’s for daily expenses. Saving 200 yuan is good. Interpersonal relationships cost money. If you come to my house, I have to treat you. Expenses are high now, I’m telling you the truth. Isn’t that right? Money makes the mare go—who has money is the boss; who doesn’t is a nobody.

Q: Do you think your lifestyle has been influenced by foreign tourists?

A: No. Think about it—you come to my house for dinner, I quote a price, you pay. It’s clearly priced. It’s just a business transaction. Am I right? To be honest, when I was your age, things were different. I don’t believe how things were before. Now society doesn’t value kindness—it’s all about money.

Q: So it’s all about money, focusing on material gains. Are the sanitation workers and security guards here all locals?

A: No, some are outsiders. That’s how it is—young people are valuable; old people aren’t. Yes.

Q: It’s true that young people are more agile, especially as they get older.

A: Exactly. Young people have quick minds, just like we did when we were young.

Q: Where do your children work now?

A: They work nearby. They go out in the morning and come back. They work jobs—if they don’t work, there’s pressure. Isn’t that right?

Q: They don’t run the farmhouse inn at home. Who runs it then?

A: We don’t want to make big money—just earn a little each year, that’s enough.

Q: How much does your family’s farmhouse inn earn annually?

A: About 20,000 yuan a year—gross income. That’s just me running it.

Q: No, I mean the entire family’s farmhouse inn.

A: You can ask them. Anyway, I stopped running it because I wasn’t making money.

Q: When did you stop?

A: I ran it for two years and then quit. There were no customers.

Q: When did you start?

A: I started when it first became popular. I invested so much money, but there were no customers. The village party secretary even came by.

Q: Yeah, it’s frustrating not making money. Are there many people in the village still farming now?

A: No one farms anymore. There aren’t even vegetable plots left—some families don’t have vegetable gardens at all.

Q: What happened to the land?

A: It’s been used for tourism development.

Q: So the land was converted for tourism. What do you think are the biggest changes in the village in recent years?

A: I don’t know.

Q: What do you think rural tourism here should do to develop better? It hasn’t been doing well these two years. What can attract more tourists?

A: Good advertising is key. If the advertising is good, people will come; if not, no one will. To be honest, that’s the truth. Now it’s all about advertising.

Q: Is mobile phone and internet coverage convenient here?

A: Definitely much more convenient than before. In the past, we had to shout from upstairs to call someone. Now we use phones and WeChat—calls are free. I’m telling you the truth.

Q: Do you think life is better now or before?

A: People were healthier before. Now people aren’t—because they don’t know how to take care of themselves.

Q: Are there any recreational activities in the village now?

A: Some people play cards, but I don’t gamble—that’s good. I play with my phone, sit around, walk, and chat with people when they come. That’s it. Isn’t that right?

Q: Do you think many people have moved out of the village? Where did they move?

A: To Guli.

Q: Why did you stay in the mountains instead of moving?

A: I don’t want to move. It’s voluntary.

Q: What happens to the old houses after they move?

A: The old houses have big stoves. Some are demolished, some are cleaned up. Isn’t that right?

Q: Do you think the rural atmosphere has faded since they left, and you don’t see old friends as much?

A: How to put it? They don’t come around anymore. We all go our separate ways. To be honest.

Q: Could you tell us your age? We need it for our research sample. We just need the age, no other personal information. How old are you?

A: 58 years old.

Q: How many years of education did you receive?

A: Junior high school.

Q: Having a junior high school education was impressive back then. We have gifts for you—would you like toothpaste or soap?

A: Can’t you give me both? Good things come in pairs.

Q: Sure. Thank you very much! I can help you polish this English translation to make it more natural, or convert it into a formatted document (such as Word or Excel) for easier use.
