# Supplementary material for: Rurality representation and changes in rural tourism destination
Source: PLoS One. 2026 Apr 21;21(4):e0347226. doi: 10.1371/journal.pone.0347226 (PMC13098982; doi:10.1371/journal.pone.0347226)
Supplement: S1 File — (ZIP) [file pone.0347226.s001.zip › supporting information/世凹村录音及转译文本/jsa1.docx]

Q: Are volunteers needed every day, or only on holidays?

A: JM: Just for the three-day holiday, the Dragon Boat Festival holiday.

Q: So, the volunteers are providing community service for them?

A: JM: Right.

Q: What do you usually do in your daily life?

A: JM: Usually, working overtime and looking after my grandson, that's my leisure time. If the volunteers need anything, I come out to help them.

Q: Is the volunteer program organized by our village committee?

A: JM: Yes, participation is voluntary.

Q: What was your original intention for joining this volunteer activity? Why did you decide to participate?

A: JM: To make a little contribution.

Q: Oh, so you're quite satisfied with life now. Did our village start developing tourism around 2011-2012? What did you do before tourism started?

A: JM: I worked with motors, in the garment industry.

Q: Didn't your family farm the land?

A: JM: No, haven't farmed for several years now.

Q: So, you came back after tourism started, right? Does your family run an agritainment business or something similar?

A: JM: Just a vegetable garden for the family.

Q: You still maintain it now?

A: JM: Yes, yes, we have a small vegetable garden. This area here is the garden plot.

Q: I see this whole street is engaged in agritainment and catering. That must be our biggestfeature here, right? There are quite a few 'Beautiful Village' projects now. What do you think makes our 'Shiwai Taoyuan' different from other places?

A: JM: Thefeature is the agritainment. Shiwai Taoyuan has many agritainment businesses these days, the home-style dishes are distinctive.

Q: So, catering is the main competitive advantage compared to other places. We learned from some residents yesterday that tourism was particularly booming from 2013 to 2015, but it's not as hot as before recently. Why is that?

A: JM: It was the pandemic. The two years before were very good too.

Q: How many customers can your family's agritainment typically receive in a day?

A: JM: Full.

Q: Full capacity? Roughly how many tables?

A: JM: Here, generally five or six tables, sometimes seven or eight tables. This area, we all have them, each household is private.

Q: What's your approximate annual income?

A: JM: The income... it's down below.

Q: Oh, you are down below. I asked those up there yesterday, their income is quite high. They said they make about three to four hundred thousand a year, and their family life is quite good. So, since life has improved, and the quality of life has increased, in what aspects do you feel your quality of life has improved?

A: JM: Clothing, food, housing, and transportation have all improved.

Q: Regarding housing, your houses were renovated, right? The government worked on the houses together?

A: JM: The government, including the trees here, built walls around this area, those tree flower pots... then the government fixed it up nicely.

Q: So it was based on our old houses, more like a renovation. What about transportation? I see after tourism development, were the roads dirt roads before?

A: JM: It's been cement roads for many years now. Before tourism, before 2010, they started building cement roads even before 2010. There were no dirt roads.

Q: After investing in tourism, have the roads changed in any way?

A: JM: Changes, maybe just that the roads were repaired later, if they broke, they got fixed.

Q: Is travel more convenient for you now?

A: JM: Convenient, convenient.

Q: How did you travel before? What were the transportation options like back then?

A: JM: Transportation was inconvenient before, now it's convenient. Taking the bus, going out is all by bus now.

Q: Do families now buy cars and travel by themselves?

A: JM: Yes. In recent years, many families in town have two cars. Like our family has two cars, one for my son, one for my daughter-in-law. We go to places nearby.

Q: And they take you out for fun sometimes. What are our usual leisure activities now?

A: JM: Leisure? In the evening, we just dance square dance.

Q: Do you sometimes travel out with your children? Yes, sometimes?

A: JM: Mhm, we go out once every year.

Q: That's quite happy. Before tourism, when you were working outside, you didn't have these leisure activities, right?

A: JM: Mhm, right.

Q: You feel that after tourism development, life overall is very satisfactory, and you quite like your current life?

A: JM: Definitely.

Q: Do you still miss the old life? What aspects of the past do you miss?

A: JM: I do miss it too. Anyway, life was hard back then, but we were happy. Now, with a daughter-in-law... one's actions are not one's own.

Q: Right, maybe compared to when you were young, it's like that.

After tourism started here, have your relationships with neighbors changed at all? No? But if we all run agritainment, there's bound to be some competition?

A: JM: Competition, of course there's competition.

Q: So, communication between neighbors is still the same as before, right? You still chat, play cards together, just like before, it still happens.

A: JM: Right, right, right.

Q: Have you been to other rural tourism villages? Ours is just very good, right?

A: JM: Mhm. Then you don't really want to go out. We don't really go out to play elsewhere. We go to places like Guli (subdistrict)there, Xincao there, and places like Huanglongxian.

Q: What's the difference between Gulithere and our place?

A: JM: Both are good.

Q: I see. Regarding tourism here, how has the number of tourists been maintained over the years?

A: JM: Not bad. Not too many, not too few.

Q: After developing tourism, what changes have there been in the village's environmental greening?

A: JM: Greening and the environment are just like this now. Much better than before.

Q: With many tourists coming now, has the amount of garbage increased?

A: JM: Actually, when there are more people, sometimes there are more, sometimes less. When there are fewer people, maybe someone is responsible [for cleaning]. Before, no one was responsible. So actually, the environment is relatively better now.

Q: What is your ideal vision of a rural village? What kind of rural life do you imagine would make you feel most comfortable?

A: JM: The one I choose now... definitely even better than this.

Q: Specifically in which aspects? For example, in housing, transportation, income, leisure activities?

A: JM: Right, needs improvement. It definitely needs to improve.

Q: All these aspects need improvement. So, are you dissatisfied with anything currently? About our village, what areas do you think need improvement?

A: JM: How to improve, improve... I didn't go to school.

Q: You can think about it, just your own thoughts.

A: JM: Thinking about it... making it better would definitely be good.

Q: Specifically, which aspects do you think could be made better?

A: JM: Just like where we live, if that place could be拆迁, that would be good.

Q: Complete demolition and full re-planning?

A: JM: Right. Just like this... (unclear)

Q: I understand that before tourism, you had farmland, right? We had land here, but after tourism started, the fields were taken over, there's no farming activity anymore. Do you find that somewhat regrettable?

A: JM: Some things became more comfortable, but it makes you feel a bit...

Q: Don't you miss the old farming activities?

A: JM: Back then, everything was hard. Now it's more comfortable.

Q: Oh, so the overall quality of life has improved. Look, this is a map of our village, this is No. 4 Taoyuan. Look, here is our main road. This is the map from 2003, before tourism. Look, this is our main road, and here were our fields, see? The fields here, and here was a large pond, and here were our old houses. Now, after tourism development, look, these are our agritainment businesses, the roads we built more of, see there's a road here now, and these farmlands have been turned into our small square, and we also have a parking lot. With all these changes, do you think it has affected the rural atmosphere of the village?

A: JM: No.

Q: You see, before, there were more trees and hills, and there was farmland. But now there's no farmland, it's all become buildings. Don't you think the farmland before made the village feel more rural?

A: JM: Definitely, it was really good.

Q: With the hills and trees, the village was better, right?

A: JM: Mhm, mhm, mhm.

Q: What were the rural buildings like in our village before tourism? Our rural houses, compared to now?

A: JM: Compared to now, now is definitely better. The small old houses were all demolished.

Q: What about rural cuisine compared to before?

A: JM: The cuisine definitely has its feature now.

Q: Each has its own feature. Do you think the food from the past had a stronger rural flavor, or does the food now?

A: JM: Because our own chefs definitely cook delicious food. The current food is better.

Q: What about leisure places? In our village before?

A: JM: The leisure places are good now too.

Q: There was no small square before. Where were our leisure places before?

A: JM: Before, it was inside our own homes.

Q: Oh, I actually noticed that our village has some historical sites, like Zheng He's Tomb, right? Zheng He's Tomb, and the Yue Fei Anti-Jin Fortifications. Before tourism, they belonged to our village, right? Do you think it has any impact on us?

A: JM: No impact, no particular feeling. Now we don't even burn firewood at home, we use gas. The economy isn't good now either.

Q: Right. Do you think when these historical sites and figures were part of our village, it contributed more to the rural feeling? Now that they aren't officially part of our village anymore, does that diminish that feeling somewhat?

A: JM: Doesn't diminish it.

Q: It's the same, right? Okay. What about our air quality compared to other places?

A: JM: The air is good! Look, we have mountains and water here.

Q: Good, thank you. That's all my questions. Just one last small question: how old are you this year?

A: JM: 55.

Q: 55? You look very young, 55, more like 50, really. Okay, thank you.

So, now that your village is developing tourism, are you familiar with the situation here?

A: JM: Pretty much, pretty much.
