# Supplementary material for: Rurality representation and changes in rural tourism destination
Source: PLoS One. 2026 Apr 21;21(4):e0347226. doi: 10.1371/journal.pone.0347226 (PMC13098982; doi:10.1371/journal.pone.0347226)
Supplement: S1 File — (ZIP) [file pone.0347226.s001.zip › supporting information/世凹村录音及转译文本/jsa10.docx]

Q: We wanted to ask, what impact has recent tourism had on development here? What impact on us? There must be some effects, right? It must have some impact? Presumably, life here has perhaps become a bit better.

A: JM: Life is definitely better than before. Development is development, it certainly has benefits for life.

Q: What was rural life like in your impression? What was rural life like here before in your memory, or what is it like now? Have there been any changes?

A: JM: Definitely, the changes are good ones for rural life. Look at rural life now, right here around me, it's definitely better now.

Q: What was it like before?

A: JM: Before, life was poorer, no money. Before, even having enough to eat was a problem.

Q: You farmed before, right?

A: JM: Before we farmed. Farming was so hard! Farming small plots before, all relied on manual labor, carrying things manually.

Q: You just said it's all individual families, right? Your house is being renovated. Has the amount of farmland, like rice paddies or vegetable gardens, decreased because of tourism development?

A: JM: Now we have almost no rice paddies left.

Q: The paddies are gone because they were expropriated?

A: JM: They were already expropriated before. After the expropriation, we basically... for large fields, it must be close to 20 years since we farmed them. Over 20 years... after around 2000, we almost didn't farm anymore.

Q: So, after Niushou Mountain started developing here?

A: JM: Even before any major development started, we weren't farming much.

Q: What did you do then? The paddies were gone.

A: JM: One, migrant work... waiting for work day by day, it's irregular. Then we got a green shoot compensation fee. Like, if a family had a few mu of land, they'd get two or three thousand yuan a year. So people went out to work elsewhere.

Q: Now that tourism has developed, we came back.

A: JM: Now, since we're older, we wait at home. I work as a security guard at night, and help out at home during the day.

Q: What year did your shop open?

A: JM: I opened in 2010. 2010, that's very early. Development here didn't really start until 2011, 2012.

JM: Here, most opened around March-April or October of 2012. I opened at the beginning of 2010. Because I came back and opened earlier than them. I was about two years ahead of them, more than two years earlier.

JM: I see your place is called Niushou Mountain Local Restaurant. Has transportation here become more convenient?

A: Transportation is convenient, but still can't compare to 2017 (possibly a reference point), and can't compare to suburban areas. Of course, relatively speaking, it's definitely better than before, right?

Q: Have cars increased? Has noise increased? Or has traffic become inconvenient for you?

A: JM: Of course, for our location, it doesn't affect us much. Because we aren't right next to the provincial or national highway. We're inside the area. And the cars don't affect us, no real impact. By evening, here... only during the day are there more cars. By evening, there are almost no cars coming in, you know?

Q: Is household garbage here now centrally managed?

A: JM: Household garbage is basically collected every morning. The village appearance has improved. For our place, the village appearance is relatively better than the average rural village. I see it's very clean here. Because, look, our sector is managed by the sub-district office here, the standards for some things are higher, the regulations here are more emphasized than other places, more personnel are assigned.

Q: I see many people. But are personnel like security guards or cleaners mostly local people?

A: More... security guards or cleaners or employees... are more locals?

JM: Generally speaking, people from our own sub-district... usually very few are from our specific village.

Q: Very few villagers, right?

A: JM: Because our villagers are scattered. The people in our village now, what we're talking about, the permanent residents here are only a few dozen people, right? Basically, now many are outsourced to contractors. Like their contractors, including those over there, are all contracted out to others. The residents all live in Guli Town now, you know?

Q: Are there many outsiders?

A: JM: Relatively speaking, in our village's population now, it's basically half local, half outsider, right? Our village itself only has sixty or seventy people living here. So, are they all outsiders here? Meaning, after tourism developed, outsiders came. Some run businesses in houses they don't own, because all the houses are just leased out for operation. I see, like that barbecue place with the Mongolian yurt, outsiders, still that line, including the one called 'Silk Road Style'. People from Qinghai.

Which one?

JM: They aren't the 'Silk Road Style' one. That hotel is also run by outsiders. Including this place called Xilu Academy, also outsiders. This one is also outsiders. I see it seems not open. This one is called... Jiaohua Mountain Villa.

Q: I see everyone minds their own business here. Has communication between villagers decreased compared to before?

A: Right, because each family is running their own business. That's how it is.

Q: So, neighborly relations aren't as good as before?

A: JM: Definitely not the same as before. As society develops, and as people's conditions improve, the human feeling isn't as deep as when we were poor. When there's no money, people have feeling. When there's money, they only recognize money, not feelings, understand? For any task, if you just talk about money, if there's no money involved, they won't help you, right?

Q: What is your ideal rural life like?

A: JM: Ideal life... for example, living in a relatively good environment, having a stable income. Good. You want spaciousness and goodness in the rural areas, right? Supporting facilities keep up. That's called a carefree life. Isn't that the ideal life? That's what we want, right? Life without worries, wouldn't that be a good life?

Q: Look, our rural area here must have its own characteristics, different from others, different from the city. What do you think best represents our rural area here?

A: JM: What represents us... for our small village, now, is basically just opening your own agritainment business. It's the agritainment. They can't make huge money every year anyway, maybe make one or two hundred thousand yuan a year. It's just a bit more free than migrant work.

At home, anyway, you don't want to work outside for a boss with restrictions. You can do what you want.

Q: What's your family's approximate annual income?

A: JM: Roughly, generally, also around one or two hundred thousand yuan. If doing well, around 200,000. That's how it is. It's similar to having a job, but a bit freer and better than a job.

Q: Are you a local? Do you have any activities here during holidays or festivals?

A: JM: Activities are fewer now than before. There are interactions, but now with the internet, it's less than before.

Q: What did you have before?

A: JM: Before there were... things like rural temple fairs, holding cultural performances and such. Now, relatively speaking, it's less.

Q: We see you have Zheng He's Tomb here, and things related to Yue Fei...

A: JM: That's inside the Niushou Mountain Scenic Area. Our place can't use it. Our village relies on the Niushou Mountain Scenic Area. Our... the steel gate that's not prosperous... isn't it connected? It's not the same main gate. Our gate on this side is closed.

JM: It's not that we aren't connected, we are part of it. Before, before the Niushou Mountain Scenic Area was developed, we were together with it. If you wanted to go to Niushou Mountain, to Zheng He's Tomb, to the Yue Fei Anti-Jin Fortress, we could come and go freely.

Before? Now you can't. Now it's controlled by the scenic area management, restricted by them.

Q: Right. What areas do you think still need improvement here?

A: JM: The areas needing improvement... definitely things need to be done. There are certainly things the government needs to further improve. Further improvement. Currently, for our village, regarding the 'Beautiful Countryside' aspect, they almost don't manage it much anymore.

Q: No one manages it?

A: JM: Because this place is... because it and other 'Beautiful Countryside' places... our place basically belongs to Niushou Mountain. After the scenic area development, it's basically... this Shi'ao Taoyuan area is just not important anymore. The focus is all over there, right?

JM: Here, you are left to develop on your own. It's like 'the Eight Immortals crossing the sea, each showing their divine powers' – you do your own thing. The government doesn't support like before, promote you, like they did for various counties before. They don't do that anymore.

Q: How do you think this issue should be resolved best?

A: JM: Resolving this... this problem... we can't speak for everyone. Now, regarding the government's role, they have their plans. The government's idea is to build roads in this area. The government thinks that later, isn't there the Jinling Town project? It's part of the Niushou Mountain and... this Shi'ao Taoyuan area. Later, they probably think that after developing that well, our village will benefit and develop alongside it. It's not like other places in Guli, like Huanglongxian area, which they are focusing on developing as a 'Beautiful Village'. For us, they almost... the last couple of years, nothing much, just maintaining the status quo.

JM: To be honest, now compared to before, our place feels worse than before. Including around 2013-2014, that was the best time, the peak. The government valued this area then. Right. Now they almost don't value this area anymore, they've moved their central focus entirely to other places.

JM: Is the idea that later, when Jinling Town is built, it will drive development here and make it better?

JM: Once it's built, they might drive you away, demolish things. People wouldn't be needed.

JM: Probably this area is directly within the planned scope of Jinling Town.

JM: Jinling Town isn't that thing they are building coming from Guli? With those small houses, turf houses? Right by the highway exit. Later it extends from there all the way to here. It's an area of over 800 mu.

JM: The original residents built... we look forward to it? Because we are established here, we definitely hope to stay here. We don't want to leave. Running an agritainment, doing our own small business, is definitely good, right? Living in the town is the same as living here? After I earn money, I can buy an apartment in the town (or up the mountain?).

Q: Your family already bought one over there?

A: JM: This... took out some loans, bought one.

Q: Do many people from here buy apartments in the town or the city?

A: JM: In our whole village area, not many buy in the city proper. For people like us from the countryside, truly buying in the city proper... city apartments are hard to buy. Generally, some have, but few. In the country, there are also many... intentionally... Some families got relocation money and left. Some relocated, some left. They got one or two apartments in Guli, but still have a house here in this place.

Q: Would you prefer to live in the city center?

A: JM: Living in the city... only young people. For us, we feel more comfortable in the countryside. It's the young people who consider children's schooling. The countryside... outside... the city is convenient.

JM: The city has its advantages now, the countryside has its strengths.

Q: What do you think is the biggest advantage of the countryside?

A: JM: The biggest advantage of the countryside is the good environment. The planning and design of city residential compounds, the homes are definitely more rationally designed than rural houses, more comfortable, right? But the external environment is not as good as the countryside. The home environment is definitely better than the countryside, but the outside environment is not as good as the countryside, you know?

Q: What about interpersonal relationships, countryside vs. city?

A: JM: Interpersonal relationships in the countryside are a bit better than in the city. Because in the countryside, you can just drop by someone's home, visit casually, interact with people. In the city, interaction isn't possible, you can't really interact.

Q: After starting tourism, with competition at home, have these interpersonal relationships been affected?

A: JM: Relatively speaking, there's a little bit. But overall, they are all fellow villagers. Sometimes, in some aspects, you feel it inwardly, but you still have to keep up appearances on the surface. When you meet people, you still greet them, right? Relatively, there might be some [tension], but you keep it inside. When you meet face-to-face, you're still polite and courteous. After all, you've lived here for decades, grown up on this land.

Q: Has the water quality or air quality here changed because of tourism activities?

A: JM: Water quality... because now, it doesn't really affect it. Because our sewage here has a sewage network, it's systematic. Drinking water is supplied tap water. Now water usage doesn't involve using pond water; everyone uses tap water. Water discharge goes through pipes, it's all channeled, not just flowing randomly. Relatively speaking, even if it's worse in some way, it's definitely much better than before.

Q: Good. What about transportation and communication here now?

A: JM: Transportation is relatively convenient, as I said before. Still lags behind suburban junctions, but compared to before, say 20+ years ago... back then, when we were young, in our twenties, over 20 years ago, our energy was good! We'd walk over ten li (5+ km), sometimes catch a tractor, and that felt incredibly convenient, you know? Compared to that time, now is definitely... society is developing, getting better and better. It's just the speed of development varies. But overall, it won't go back to the past.

Now we already have medical insurance handled here.

JM: For us here, it seems better than Northern Jiangsu area, definitely better than some other places. Much better than central and western regions. For example, compared to those more advanced places, there's certainly a gap. But compared to poorer places, it's definitely much better.
