# Supplementary material for: Rurality representation and changes in rural tourism destination
Source: PLoS One. 2026 Apr 21;21(4):e0347226. doi: 10.1371/journal.pone.0347226 (PMC13098982; doi:10.1371/journal.pone.0347226)
Supplement: S1 File — (ZIP) [file pone.0347226.s001.zip › supporting information/世凹村录音及转译文本/jsa17.docx]

Q: What was this place like long ago?

A: JM: Long ago, this place was just farmers' houses. After the government developed it, it became agritainment businesses. Long ago, these were just farmers' houses.

Q: You mentioned developing rural tourism here these past couple of years. What element do you think best represents the rural village?

A: JM: Especially now, it's the local cusine.

JM: We have Mongolian yurts and whole roasted lamb. This village's most distinctive feature is my family's place, because we have lamb skewers and whole roasted lamb. Where do you see roasted duck? You're from Inner Mongolia? I'm from Xinjiang, boss lady. I've been to Xinjiang, very happy. My Uyghur and Mongolian are quite good, hard to explain how I learned.

JM: I cook ethnic dishes, Xinjiang food, Inner Mongolian food.

Q: You think yours is also a distinctive representation of the rural village?

A: JM: Mongolian yurts, including tourist spots having Mongolian yurts, can also count as a highlight.

Q: Master, why did you choose to come to this place initially?

A: For the environment. The environment here is relatively good.

JM: Mongolian yurts are on the grasslands, not people inside the yurts...

Q: Did you come through a government recommendation event, or through friend introductions?

A: JM: Anyway, business isn't good, hard to continue. Information posted online.

Q: What was the countryside like in your original impression, right?

A: JM: The current countryside has flowers, plants, grass, trees, lots of fruit, down-to-earth, close to nature.

Q: For example, regarding interpersonal relationships, is there any difference between rural and urban? Or what can represent the rural aspects?

A: JM: Rural people, after all, can be petty. City people are more broad-minded, having seen the world.

JM: Rural people can be relatively narrow, probably exposed to less information. After we came here, our relationship with the villagers and neighbors is handled quite well.

JM: You think the villagers here are still quite simple and honest? Neighborly relations are all quite good. Should be no problem.

Q: What do you think the pace of life is like here? Leisurely or something else?

A: JM: And it's seasonal for them, not for me. My customers are some people from urban Nanjing, who specifically come for the whole roasted lamb. Of course, I'm not solely reliant on tourist business. We have various sources, various companies. For example, we get bookings from like hospitals. Companies also... they contact you directly, right? Direct bookings like that.

JM: When we get bookings... You think tourists they handle are seasonal? After Spring Festival, in spring, until around June. After the tourists come, maybe business is better, otherwise not great. Like me, I also do tourist business occasionally, it's not the main source. If important guests come, you could say... after October, the scenic spot is completely empty of people. My busiest season is when the lamb season arrives.

Q: Because the old street was built, the water doesn't flow anymore, is that it?

A: JM: Look, no one manages it.

JM: This water, no one manages it either.

JM: Now, what do they manage? Look at the water down below, you know, right? You can tell just by looking.

It's not good now. The situation at the end of the year isn't good, right? The worst.

JM: Our village is the most poorly developed now. Look at this place, it's terrible, and no one manages it. Fake management. They say inspections are coming, then they manage. If the inspection bureau isn't checking, they don't manage. When higher-ups come to inspect, then they manage again.

Q: You mentioned developing rural tourism here. What are the rural elements during this period?

A: JM: The people in the rural town... the cadres are... not the initial old street, the ancient town? The old street? Big problem. It's all fake.

JM: Every place is dirty beyond belief.

JM: The water long ago was good. Now the water is already undrinkable.

Q: Yes, the water quality has become very poor.

A: (Implied agreement).
