# Supplementary material for: Rurality representation and changes in rural tourism destination
Source: PLoS One. 2026 Apr 21;21(4):e0347226. doi: 10.1371/journal.pone.0347226 (PMC13098982; doi:10.1371/journal.pone.0347226)
Supplement: S1 File — (ZIP) [file pone.0347226.s001.zip › supporting information/世凹村录音及转译文本/jsa18.docx]

Q: From the outside, JM Village's landscape looks better and better now.

A: Yes.

Q: What was it like originally, roughly?

A: JM was just a mess originally, like other villages. Things were piled up everywhere, there was no real landscape to speak of.

Q: What do you rely on for a living?

A: Working away from home as migrant labor, and farming a little land at home. Now we mainly rely on agritourism. The village appearance and environment have also improved quite a bit, improved a lot.

Q: Were all these trees here before?

A: The trees were originally here, but later we added some more trees, removed the scrub trees, and planted some other types.

Q: Is your house newly built?

A: Yes, this was done later. The shed was definitely built later. The shed from 2012 was already demolished, and we rebuilt it. The 2012 shed was wooden, and over time it rotted. This one was just built last year. I heard the exterior renovation work here was supported by the government?

A: Yes, the exteriors were all done like this. So they built several horse-head walls , but this shed we built ourselves.

A: We built it ourselves.

Q: I see the overall style, the horse-head walls, the Hui-style architecture work was done by the government. Do you like the horse-head walls?

A: So-so.

Q: Do you think it represents a unique rural character?

A: I think a little bit, because the horse-head wall is part of Hui-style vernacular architecture.

Q: Did you used to farm here before?

A: Yes, we have land.

Q: Do you have poultry?

A: Yes, we raise poultry. Before they were free-range, now they are penned up.

Q: Do you still have them now?

A: Yes, we have them. The pens are in the back, in the yard. They're kept in an enclosure. Yes, before they were free-range, now they are penned up.

Q: Has garbage increased because tourists come?

A: No. Our garbage... is it collected at designated points? Every household has a trash bin. We put the garbage in the bin, and then someone comes to collect it every day. It's managed quite well. Before, garbage was dumped everywhere. Actually, there was probably more garbage before, just left everywhere with no one managing it. Now, the management is quite clean. Probably because we have more outsiders now, the domestic waste is all handled.

Q: Are there any issues like noise pollution because tourists come?

A: No.

Q: So, actually, developing tourism has had a relatively good overall impact on this place, right?

A: Definitely good. Let's look at this photo from 2000... You didn't have tourism developed then, but someone already took photos. Before and after development... I can't find the photo of the house, it's not posted.

A: Originally it was simple, rustic brick and tile houses. Now it's these multi-story ones. This is how it is now.

Q: How many years has your family been doing this (agritourism)?

A: We started in 2012. It's been 8 years now. Right at the beginning, it was the same... Before it was all natural scenery. The natural scenery was quite good too, the landscape was nice. It was all natural. Now it's developed later. Before, all this was farmland, fields, right? And the trees weren't like now. There also weren't these hardened roads. It was all just the ground in front of the houses. People might chat here or hang out in front of their houses. Look, this is a remote sensing image, it's bright here, meaning this area had no trees, it was just earth. So this is probably the scene in front of your house, right?

A: Before, the natural environment was very good, nestled against the mountains.

A: The natural environment was very good. In recent years, human traces have become more and more obvious. Our cars have also increased, regarding these houses.

A: The natural landscape is gradually disappearing, becoming man-made landscapes. It's all cultural landscapes now, cultural landscapes are increasing.

Q: Have there been any changes in water quality? How is the water quality now?

A: A long time ago the water quality was very good. Well, a long time ago in the countryside, the ponds, the water quality was quite good. Later the water was still quite good, it really was quite good.

A: Later, just this year, the water quality has gotten worse. It's not just our area, it seems widespread... It shouldn't be like this. I see the village also did drainage, did it well, but it doesn't seem related.

A: The water quality now isn't as good as when we were kids, right? The water quality was good. When we were kids, there was no tap water, we drank from the pond water. The pond water was clear to the bottom. The water quality in our village here is still okay.

Q: Maybe it's because of using things like soap, detergent, which might cause some pollution, or agricultural pesticides might cause some pollution. Anyway, the water quality has definitely changed overall. It's not just your village, other places are like this too. But because of tourism development, your water quality is still passable, right? It doesn't stink or anything, does it?

(Implied A): (Agreement implied by the following question)

Q: What's the pace of life like now? Compared to before, are there any changes? Before, maybe you went to bed at a certain time?

A: Before, it was busier during the farming season. Because we worked away from home, the two elders were at home. When it was busy, we came back to help. Now, running the agritourism, we focus on the family. If guests come today, we cook. If no guests, we rest. We still have a bit of land prepared in the back, we farm a little. Usually, we grow flowers. Actually, it's quite relaxed.

Q: What time do you go to bed now?

A: Normally we usually go to bed around 10:30. Around 10:00 to 10:30.

Q: Before tourism development, the pace was basically the same, wasn't it?

A: (Implied agreement)

Q: Was life busier around 2014, 2015 when you had more tourists?

A: That was very busy. Busy during the day, but we rested normally at night. Because mainly people come during the day, come to play during the day, and go back in the evening. There are fewer guests at night. It's not like in the city where people stay over; relatively few stay. They come to eat during the day and go back the same evening.

Q: I see you have a car. When did you buy it?

A: We've had the car for a long time. This car here, bought in 2014.

Q: Do you often go out shopping because of it?

A: Yes, we often go out shopping now. We also use the car to buy groceries. We go to the Guli market to buy food. Before, we probably didn't buy all these vegetables and such? If you don't buy, it's not enough to eat. Even before starting tourism, we occasionally bought some meat dishes. Vegetables we still grow ourselves or buy. Meat, fish, those things we still had to buy.

Q: Do you eat out now?

A: Rarely eat out. It should be very rare, right?

Q: Is the food outside better than your home cooking?

A: No time to go out. You're at home every day, and if guests come, you can't leave. At mealtimes, you're stuck there. If we eat out, it's also in the evening. I think it's very rare.

Q: How many... 'heads'? (likely referring to rooms/tables) The house...

A: 5 private rooms. Yes, the main hall is quite good too.

Q: How have neighborly relations been these past couple of years?

A: Relations have always been very good. No conflicts, all very good. Relations are quite good. Right here in this awning, you go to their house to talk. Sometimes we go down there to play, sometimes they come up here to play. During leisure time, basically we are in front of our houses, all in front of our houses.

Q: Because of tourism, is your Mandarin getting better? 'It's hard to change one's local accent'?

A: It's not possible... If people speak Mandarin, then we speak Mandarin, but it's not fluent. I see your dialect isn't too strong, I can understand it. For us, changing... it's similar to Nanjing dialect, the same. Understandable, sounds fine.

A: It's pretty much the same as in the city now, actually. Before it might have been a bit worse.

A: Before, the external environment was probably worse. A bit more messy.

Q: And household appliances, around the year 2000, you probably had fewer, right?

A: Definitely fewer appliances. Just one fridge, one TV. Now it's different, several fridges.

Q: Was your house here before?

A: Here. The house is the old house, hasn't been moved. It's the old house, right?

A: Yes, this house is right here, next to the mountain.

Q: Is there anything like tea in the mountains?

A: We farm the land in the mountains, farm up there. There's a plot of land up there. On the mountain itself? No, what land is on the mountain itself? Then, behind here there might be a small plot.

Q: Have there been any festival activities here in recent years? Festivals and activities here? Celebrating holidays?

A: Before, there was Dragon Boat Festival, we had zongzi wrapping competitions in the evening. There was also an Anime Festival, they held an Anime Festival. That was after they started developing tourism. Before tourism, there weren't really any festivals. Sometimes they organized some activities, but we had no time to go. The village organized them. We used to wrap zongzi before.

Q: What's the rural atmosphere like?

A: The rustic simplicity of the countryside is the same as before, hasn't changed much. It's fine, no issues. People are becoming more polite, more courteous.

Q: What do you think are the elements that best represent our local countryside? How are we different from the city? Where is the biggest difference that shows we are not like the city?

A: We have our unique features: good air, good environment, quiet.

Q: Besides eating and accommodation, are there other entertainment activities here?

A: We are right next to Niushou Mountain. Everyone goes to Niushou Mountain to play.

Q: Are there differences between city people and rural people? Do you feel city people are colder?

A: There are all kinds of people.

Q: But isn't there also Zheng He's Tomb and something about Yue Fei here?

A: Right at the foot of Niushou Mountain.

Q: You've been here all along, right? You've always known about these?

A: Been here all along.

Q: Is there any religious belief here? Do you believe in religion?

A: We don't believe. That stuff isn't realistic, right? We didn't believe before either, didn't believe then, don't believe now.

Q: So you just go to look?

A: No, we don't go. It requires tickets now. They don't make exceptions for anyone. Before, when Niushou Mountain wasn't developed, it didn't charge admission, we could come and go as we pleased.

Q: What was this place like originally? Niushou Mountain has undergone quite significant changes, hasn't it? There was a Phase 1 and Phase 2 project.

A: Before, Niushou Mountain had a pagoda on it, that Tang Dynasty pagoda has always been there. Then there was a quarry pit nearby... When they were almost done mining, they invested and built a big... a large hole, a big pit inside. There were also some 'Ox Noses', 'Ox Eyes' on the mountain. Yue Fei's anti-Jin fortifications were there. And also cliffside carvings, those are from long ago. Before, it was all open, you could enter freely whenever you wanted. The local elementary school spring outings, student spring outings, were all taken to Niushou Mountain to play. Then after they developed it and spent money developing it, they fenced some areas off. Now you need tickets to enter. They turned the quarry pit into an underground palace (地宫). And then they rebuilt that pagoda, that big pagoda was built later, newly built.

A: The original pagoda is still preserved there, they couldn't demolish it. The quarry pit became the underground palace. The relic (Buddhist śarīra) is placed in that underground palace.

Q: Have you bought a house? Bought a house in the city?

A: Houses are so expensive, can't afford it.

Q: You think the countryside is quite good. Do you really want to buy a house in the city?

A: Actually, I don't want to go now, don't want to go to the city. This place is actually quite good, it's really a place for retirement. But for young people, they don't think that way. My daughter feels living in the countryside is very inconvenient, transportation is inconvenient. The city is convenient, everything is convenient.

A: For work, it might not be as convenient as the city.

A: If you don't have to work, or after work, you feel the countryside is quite nice. The space is large, the air is good.

A: Living in a city apartment block, basically it's just one... it's not this big. This is very big. This must be three to four hundred square meters... This area basically has nothing... altogether it's about that.

A: Really, I think it would feel very oppressive, not as open as yours.

Q: The city is livelier.

A: The city is colder, I feel. Like, upstairs neighbors don't really interact. Not like the countryside where we might visit each other. If you live there, you basically don't even know your opposite neighbor.

A: It feels like people are constantly flowing. Everyone leaves early and returns late, busy with work, no time. You go home and close the door. You can't even get them to come out.

Q: In your impression, what was life like in our countryside before?

A: Before, the conditions weren't as good as now. Our main focus was farming, and there was a lot of farm work. During leisure times, we would just chat.

A: Now, fewer people farm, it's almost gone.

Q: Did you grow vegetables before? Do you still grow vegetables now?

A: We have a vegetable garden, for our own consumption. Yes, we have vegetables.

Q: What do you think an ideal countryside is like now?

A: This is the ideal countryside.

A: Now it is. It should have good conditions, and have this kind of environment. What we value about the countryside... having household appliances, having a car, having open space, a good natural environment. Like this.

Q: Are there any areas that need further improvement?

A: We also hope it would be even better if the subway reached here. Transportation is the only shortcoming. Although we sometimes have buses, if it connected directly to us, it would be more perfect. It currently goes to Tianlong Temple, right? If we want to go to Xinjiekou or Gulou area, it seems we still need to take a bus to Tianlong Temple first, then transfer to the subway there.

Q: How many stops is it from here if we take the subway?

A: It's quite a few stops for us to get to the subway. Driving might take about 20 minutes... and parking outside isn't easy either.

A: If that road from Tianlong Temple extended directly here, that would be good. That's the only deficiency.

Q: What is your impression of what the countryside *should* be like? I mean, the countryside from further back in time, what was it like? Do you have any stereotypical impressions? For example, do you think the countryside was relatively poor? Do you have that impression?

A: When we were kids, it was 'face to the loess, back to the sky' – cutting rice, busy farming seasons, transplanting rice seedlings.

A: The impression of the countryside from childhood is probably that farm work was very hard.

A: Busy every day, very hard work.

Q: And the conditions were poor?

A: Conditions were poor too.

Q: You think the countryside should be like it is now, not like it was before, right?

A: Now, everywhere they are building 'Beautiful Countryside'. Also, they've built roads everywhere. Now the roads are all cement roads. When you go out, you don't need to wear rubber boots anymore.

Q: You used to wear rubber boots?

A: Before, if you went out, you definitely had to wear rubber boots. The roads were all mud.

Q: Before, you probably didn't go out shopping much either?

A: Before, very rarely. Before, when we went to Nanjing from here, we all rode bicycles. It took over 40 minutes by bicycle.

Q: When was that?

A: That was when we were kids... probably before the 80s? The 70s, 80s. We all rode bicycles when going out. Later, after this road was repaired, the bus service started. The bus service started in the 90s. First, they built the road, a new road. After the road was finished, then the bus route opened.

Q: Did your family used to cook using a large wok and stove, using firewood?

A: We still have that now.

Q: Did you have to gather firewood?

A: We gather firewood, right on the mountain. No trouble getting firewood.

Q: Do you use natural gas now?

A: Natural gas hasn't reached us here.

Q: Liquefied Petroleum Gas (LPG)? The bottled kind, canisters?

A: Yes, canisters of LPG. Piped is natural gas. Natural gas isn't available here.

Q: What elements do you think can represent the countryside now? What represents it now?

A: Before it was poverty, backwardness, a remote mountain hamlet. It was really poor before. Now it's clean, tidy, good living conditions, good environment, harmonious neighborhoods. Now it's beautiful, pretty, a 'Beautiful Countryside'.
