# Supplementary material for: Rurality representation and changes in rural tourism destination
Source: PLoS One. 2026 Apr 21;21(4):e0347226. doi: 10.1371/journal.pone.0347226 (PMC13098982; doi:10.1371/journal.pone.0347226)
Supplement: S1 File — (ZIP) [file pone.0347226.s001.zip › supporting information/世凹村录音及转译文本/jsa19.docx]

Q: What changes have you felt in the countryside these years? The biggest changes?

A: What's quite different from before is... JM management has become more standardized, with higher requirements. Keeping up with the times, the management here is more standardized.

Q: What was it like originally?

A: JM originally it was more laissez-faire, relatively loose, without unified management. Now it's uniformly regulated. If there's a problem, they step in to manage it; if not, they don't interfere. Now they consider and plan things before problems arise. Planning comes first.

Q: Before, it might have been the village chief. Is it still the village chief now?

A: JM You have the neighborhood committee, village committee, team leader here. The natural village has a team leader, he's considered the team leader. Originally, it was also a team leader, pretty much the same level.

Q: What is the countryside like in your memory?

A: JM The countryside in my memory was more natural, more natural.

Q: How long have you lived here roughly?

A: JM Lived here all my life, just like this. Yes, grew up in this village.

Q: Can you describe what it was roughly like before?

A: JM Before it was dirt roads. Then they paved gravel roads. Later, as things developed, they made cement roads. Now they are all asphalt, which is much easier to travel on, convenient for getting around.

Q: Any changes in housing and residences?

A: JM Suddenly... single-story houses and tile-roofed houses were the majority. Only after 2000 did people start building multi-story houses. Now, basically, half have been demolished for relocation.

A: The local residents moved out.

A: JM They live in residential complexes now. Those not demolished were taken over by the public, rented out for development.

Q: And there are still local houses run by local residents, right?

A: JM Locals... if locals run them, they run them; if not, they rent them out to others. The original countryside mainly had single-story houses, then dirt roads.

A: The toilets are different too. Originally, they were simple pit latrines. Now they are all three-chamber septic tanks.

A: JM The original countryside probably still had rice paddies, some irrigated fields, etc. Before, everyone grew rice, wheat, rapeseed (for oil). Now it's all gone.

A: JM We swapped land for social security. Living conditions are definitely better than before, living standards are better. The only thing is you can't find the feeling from childhood anymore.

Q: What feeling from childhood?

A: JM A feeling of being closer to nature. You used to have eagles, do you see them now? No. Before there were eagles... yes, closer to nature.

A: The original environment was more natural.

A: Now there are more people here, building houses, driving cars, building squares... it's definitely different from before. Before, it was all trees, mud, right? The human element is increasing.

Q: Any changes in water quality? You seem to have a few ponds here.

A: JM The ponds are cleaned regularly, cleared out periodically. Originally, they probably weren't cleaned. Originally, it was just the pond; the water quality was okay. Before, we washed rice and vegetables in the pond. Now everyone uses tap water.

Q: Before, you would farm, grow vegetables. Now you probably buy more.

A: JM Basically all buy. Basically all buy. Every household grows some vegetables because it's more convenient to grow some at home. They grow some for their own consumption, but what they grow definitely isn't enough. They still rely mainly on buying.

Q: What do you think is the element that best represents the countryside?

A: JM Before it was more natural. Now it's more convenient, daily life is more convenient now. Before, if you wanted to buy something, you had to ride your bicycle to the town yourself, it took a lot of time.

A: So life is more convenient now. Before, the connection with nature was closer. Now it's less, but life is more convenient.

A: JM Before, every morning I was woken up by woodpeckers.

A: That's probably impossible now. Now it's the phone, now it's the alarm clock.

Q: Are there any festival activities that left a deep impression on you here? Activities or celebratory events, like during Chinese New Year or other times, some holiday activities?

A: JM Those from childhood, or even now... the New Year atmosphere was stronger. Children liked the New Year. Because usually living conditions weren't very good, so during New Year the food and drink were better. Clothes were also better.

Q: What element most represented the countryside in the past? You just said the natural environment was better.

A: JM In the past, there were rice fields, natural landscapes. Things like wild rabbits, pheasants were much more common. Now, once developed, they're all gone. Can't find them. There were some natural elements. Before, the mountains were wild, rarely visited by people. Now, after development, they're open to the public. Before, it was remote and rarely visited; now, everywhere has traces of people.

Q: In terms of behavior and habits, do you think the countryside was a certain way, and the city is now a certain way? What about behavioral habits?

A: JM Regarding garbage, people are more conscientious now. Before, they just threw it everywhere.

A: Before, a lot of the garbage was naturally decomposable. There weren't so many convenience bags and such, no plastic. Originally, when they caught fish, they used straw to tie them, right? Yes. The elements were more natural.

A: JM Yes, more pure and natural.

A: Before, there was a garbage dump site at the village entrance. Now it seems every household has a trash bin.

A: JM Every household has one; they issued a trash bin to each.

Q: In the past, what do you think were the key behavioral elements of the countryside? What most represented the countryside in terms of behavior? For example, lifestyle habits, any characteristics? Then food, housing, transport. Nothing that particularly represents anything?

A: JM Before, people didn't eat much meat. Now there's meat every meal. Every family's conditions are different.

A: Food, housing, transport... housing we've already discussed. Transport, before, there were no cars.

A: Originally there was a regular bus service, now it's gone.

A: JM The flatbed cart was for hauling goods, needed for pulling things like rice. Before, every household had an ox, or a few households shared one ox. Later, tractors appeared, and they started using tractors. At that time, the ox was still used for plowing the fields or for farming, it was a means of agricultural production. And you still had to herd the ox.

Q: Spiritually, what do you think the countryside was like in the past? Spiritual elements, meaning the rural atmosphere? For example, values?

A: JM It felt like neighbors visited each other more frequently every year. Before, an entire clan might live behind one main gate, meaning relations were closer. Now it feels a bit more distant.

A: Now it's because every household is clearly separated.

A: JM Relationships between people are now mainly based on economic interactions, that's basically how it is. Relations between villagers are more economic.

Q: What is the countryside like in your impression? Do you have any stereotypes about the countryside? That the past countryside was poor, full of hardship, or very hard work? For the new countryside, what do you think the countryside should be like now?

A: JM There's happiness, but perhaps it's still about being content and finding joy, having a better mindset.

Q: So you think people in the past probably had some hardships.

A: JM Yes. Everyone worked a bit harder, but they could still eat their fill and stay warm.

A: Even though it was hard work, it might also have been a kind of happiness. Yes, now conditions are better, but perhaps the communal atmosphere isn't as strong. That is also a kind of happiness.

A: Each has its good points, right? So you think the difference between past and present is that conditions are better now, but actually neighborly relations might be worse.

A: JM The spiritual level isn't as rich as before. Now it's mainly focused on the economy. Not entirely focused on it, but it depends on one's mindset? The economy has become more important, people don't value other things as heavily, don't value them as much.

A: Nowadays, the information people access is more diverse and abundant.

A: JM There's television.

Q: Phones... originally, it was mainly watching TV, right?

A: JM Before, didn't watch TV much... anyway, the village itself was more fun.

Q: What were the main channels for receiving information before?

A: JM It was newspapers, newspapers... before we still read newspapers. Radio was also common. Now, we don't even listen to the radio while eating. Before there was radio, right? There was radio. You're not that old. 2018... you're an 80s child. 80s kid, how old are you now? 34.

Q: Okay? Read newspapers as a child? Didn't have contact with that? Must have listened to the radio, surely.

A: JM Before, that kind of... Sanyo? A very big one, that played tapes, CDs? A three-in-one machine, right? You mean that kind, yes, very big. Radio recorder.

Q: Your education level should be undergraduate?

(Note: This question seems directed at the interviewer/user, not the interviewee JM. Skipping translation of demographic questions about the interviewer.)

Q: I wanted to ask, having lived here for over 30 years originally, what aspects of the village's houses and their changes do you find most obvious?

A: JM Before, basically every house leaked. The roofs had mats installed. Brick and tile houses came later. Multi-story buildings only appeared after the new century.

Q: I heard from the village committee, and from others, that the houses are apparently still the original ones, just repaired on the original foundation.

A: Which year... some houses were completely newly built, some were repaired, right?

A: Probably the newly built ones are still relatively numerous.

A: JM The horse-head walls were all created as part of the development. The external packaging is uniform. Because the really bad houses were basically all demolished. If they were truly beyond repair... without adding a proper roof, they would collapse. Yes, if the quality was really no good. Those affecting the road widening were all demolished.

Q: So, you actually don't have a stereotypical impression of the countryside? You think although there were some negative aspects to the past countryside, it might also have had good points. It depends on how you look at it, right? You don't have a fixed objective impression; yes, it's a process of change. What is the new countryside like? What do you think the ideal countryside should be like?

A: The ideal countryside would be a combination of the past and present, that would be better. Spiritually, people need to progress a bit further, return to a simpler truth a bit more, okay? Now people feel more restless, more bored. If they're not in business, they feel like there's nothing to do. Like before, the feeling of visiting each other was happier. Now, visiting should be relatively less common, yes, doors are shut tight.

A: JM The spiritual level needs some regression. For example, materially, it should actually be like this... you think this is the countryside.

A: JM Now the government can create the conditions. If individuals work hard, they basically won't be poor. Anyway, there are all kinds of insurance, so materially it's basically satisfied. Spiritually, there is room for improvement.

Q: Do you think two-story buildings like these are also part of rural living conditions?

A: Ideal... can't really call it that. What should it be like?

A: JM Personal preferences for style differ.

Q: What kind of residential housing do you imagine for your ideal countryside?

A: JM Like this... anyway, these houses originally weren't Hui-style. Forcing them to become Hui-style feels imposed. Yes, it lacks that natural formation, natural development from before.

Q: You think if it's the countryside from your impression, the countryside of your childhood was definitely like those simple two-story vernacular houses. So a countryside naturally changes, evolves naturally. But now, after all, there is societal participation, government leadership.

A: Because everywhere they are building these Hui-style buildings with horse-head walls. Actually, Hui-style architecture and horse-head walls are relatively authentic in Southern Anhui. Here, they are all built? There, it developed naturally. Back then, horse-head walls were for fire prevention; those were real horse-head walls. Now it's mainly to create a rural atmosphere, so the horse-head walls are built to tidy up your village appearance, to make it look more... probably to have a bit more rural feel, right?

A: It should develop naturally. What it was originally, that's what it should be.

A: JM After the government's unified development, it feels like each household has lost its distinctive features. The government intervenes a bit too much, right? If they want the walls white, yours must be white.

Q: On the material aspect, what do you think the countryside should be like? Regarding the environment.

A: JM Now it's all done uniformly. Every household... if your grass is too long, you must manage it. Actually, sometimes keeping a little is better, it feels more... keeping a little of what? Your own family's characteristics. It feels like personal autonomy is restricted. Everything has to follow the government's arrangements.

Q: What are the material elements of the countryside?

A: More individuality, not uniform and identical. It feels particularly monotonous at a glance, everything looks the same.

Q: You think the countryside should develop naturally.

A: JM The city and the countryside are different after all.

Q: Developing naturally... I see you have a built a Mongolian Yurt barbecue place here, right?

A: JM Rented. Rented by outsiders, right? Ethnic minorities, Inner Mongolian, right? Mongolian, and also Qinghai.

A: But their habits are different from ours. Yes.

A: JM They probably don't interact much with you either. Sometimes you can't find them.

Q: What do you mean, can't find them?

A: JM They sometimes operate at night, don't come out during the day. Strange. We offered them lanterns for New Year, they said they didn't want them.

Q: How did they come here?

A: JM Government investment attraction. Introduced by friends, right?

A: Here, it's mainly the government taking the lead to attract people to invest here, or something like that.

A: JM When they first opened, the government probably had some support policies. Now they basically rely on themselves. They let you do whatever you want.

A: JM Because this village is under the dual management of the community and Niushou Mountain. Basically, it's like this. Yes, Niushou Mountain wanted to take over everything first. They wanted all the local residents to leave, then have you all go, so they could have unified management. But they couldn't afford the demolition, funds weren't in place.

Q: The local dialect... probably because of tourism, everyone has started speaking Mandarin. Is the dialect being lost a bit?

A: JM The younger generation probably uses Mandarin more, but it's still mainly the dialect.

Q: Neighborly relations have become more distant, right?

A: Interaction with them is relatively less.

A: JM I've asked everyone. Are there any more questions? The interview should be about enough, pretty much similar. For example, you still haven't painted a picture of your ideal countryside for me.

A: JM Can't paint it. Maybe this way is good, that way isn't. That is to say, there isn't a fixed concept. It should be a naturally growing village.

A: JM That is, not many people, not much, not a lot of human interference. The most important thing is that it's mainly natural. Yes, isn't that so? Now, it's mainly that human traces are heavier, roads, traffic...

A: Humans and nature are always a contradiction.

A: JM The countryside is mainly about greater harmony between humans and nature. It's mainly about people being closer to nature. In the city, there is more interaction between people. You can't be closer to nature, but it's more convenient. Because the city is definitely more convenient, but the countryside is more intimate with nature.

Q: Do you prefer the countryside or the city?

A: JM I feel living in both is better.

A: JM If you stay in the countryside too long, you feel it's not very lively, and daily life isn't very convenient. But if you stay in the city all the time, you feel quite stifled.

A: Probably now, people who stay in the city a lot say they'll come down to the countryside, come to play on weekends, Saturday, Sunday. This provides some adjustment. Or, if you live in the countryside too much, go to the city to adjust.

A: Some business owners, they don't do much, they also come here every now and then to enjoy life, improve their quality of life.

Q: Do you have a strong sense of identification with this place? Do you like this place? This village?

A: JM One's own hometown is definitely loved. Identification is more or less the same. Anyway, if common people's lives are better, it's definitely better.

A: The countryside's economic development is definitely not as good as the city's. If in the city, economic income would be higher.

A: JM Differ by several levels, right?
