# Supplementary material for: Rurality representation and changes in rural tourism destination
Source: PLoS One. 2026 Apr 21;21(4):e0347226. doi: 10.1371/journal.pone.0347226 (PMC13098982; doi:10.1371/journal.pone.0347226)
Supplement: S1 File — (ZIP) [file pone.0347226.s001.zip › supporting information/世凹村录音及转译文本/jsa2.docx]

Q: How has tourism development been in this village over the last couple of years?

A: JM: It started around 2014. Up until 2020, you could basically say... the company is managing it very well now, it's become a tourist spot.

Q: What is the biggest change here?

A: JM: The biggest change is the cusine. Before, there were no people, now there are more people coming to have fun. Anyway, every aspect has been developed quite well.

Q: Is the environment better than before? And what about the houses?

A: JM: The houses are our own, but the exteriors were renovated by the government.

Q: What do you think of the renovations?

A: JM: The renovations... each has its own style, definitely better than the houses we built in the rural areas before. Rural houses before were quite shabby.

Q: In this area, have the number of tourists been a bit lower than before in recent years?

A: JM: Now there are relatively too many rural tourism places. Every place is developing rural tourism now, so the number of people here is naturally relatively smaller. The first few years were crowded, very good. It's been over ten years... it started getting popular around 2014, and development began in 2012. Not much has been built in recent years, it's maintained the status quo. Every rural area is doing development.

Q: Do you think our place has any distinctive features compared to others?

A: JM: Not really... it's all muddled, that's right, I don't really know.

I see we mostly have agritainment here, the food is delicious.

JM: Anyway, it's all cusine?

It's not like the taste in hotels now, it's just like normal home cooking, I think it depends on the household.

Q: What is the biggest attraction for outsiders? What brings them here?

A: JM: The scenery, things like that... it's a village, you see, it's famous, the food is good.

Q: Do you think the rural environment and atmosphere here is a major attracting factor for city people?

A: JM: The air is so good, it's comfortable.

Q: How does the current rural environment here compare to your childhood memories? Have there been changes?

A: JM: Everything is better now, cleaner, greener. The rural areas before had dirt roads, no cement.

Q: What did you do before this area was developed?

A: JM: Before development, it was just a small village. No cleaning services or anything. Now, with this development, there's much more talk about cleanliness and sanitation. She wants to love it, but if it's not done well... anyway, before development, you farmed the land, didn't make money, had no choice but to constantly look for work outside.

Q: Now the development has taken the land, right?

A: JM: The land was all acquired. Now you have no land either. We work here now, don't migrate for work. Do you think life was better farming before? Life is better now, much better. Normally speaking, income was lower before. Now, working, you can earn two or three thousand yuan a month. Before, you know how much income you had? You worked yourself to death, how much could you earn? Not much income.

JM: Meaning life is happier now. Furthermore, I love just going to work, eating, having fun, being able to sing, dance. And there's more leisure time, taking walks.

Q: Before tourism, when we were farming, what leisure activities did you have?

A: JM: Besides playing cards at home, there was no square dancing. Now you can square dance. Because before, working in the fields, you were exhausted,哪有功夫跳舞? No one danced. Now it's extremely enjoyable. Elderly people say, now it's really extremely enjoyable.

Q: Now that many tourists come, do we interact with them much?

A: JM: If they ask, 'Where is the restroom? Where is that?' You definitely have to tell them.

Q: Do you think these tourists coming have brought you benefits? Or...

A: JM: To be honest... when they come, of course... the sanitation is definitely dirtier, there's more garbage, let me tell you the truth. But I also hope they come, it makes things more lively, right? You can't be selfish. Saying 'don't come, it's too dirty'... So it increases our income, but also brings these environmental problems.

Q: Do you think their arrival, their lifestyles, have had any impact on the local farmers, the original residents?

A: JM: No, no major conflicts. Originally, it seems there weren't any, seems everything is very good.

Q: Now that our income has increased, and our lifestyle, do you think it's become a bit more like an urban lifestyle? A little bit, right?

A: JM: I think we are now also registered as urban residents. Feels a bit like city people. But I feel the rural feeling of being from the countryside has faded a lot.

JM: That feeling from our childhood is gone. Like when we were kids, there were things in the countryside you wouldn't recognize now, you simply wouldn't understand those things. We don't use such things anymore. Like before, those were farm tools, would you recognize them?

Q: Do you sometimes miss the old life? It was quieter before, now with more people it's a bit annoying, and more and more strangers, before it was all acquaintances.

A: JM: Everyone has their own opinion. I definitely feel it's getting better and better. Like before, in the fields, one step and your foot was in mud. Was that good?

Q: Would there be some competition between them, affecting relationships between the two... (likely meaning between neighbors/businesses)

A: JM: That's their inner thought. We definitely... for example, if two families run businesses, your family has more customers, mine has fewer, I'd definitely be a bit annoyed. Somewhat. For example, if a guest asks me, I can't tell them to go to your place, right? Definitely try to pull customers to my own place.

Q: What major problems do you see with tourism development?

A: JM: More restaurants mean dirtier, more people. Sanitation definitely needs work, the environment is worse.

Q: Did this village have any festive activities before, like dragon/lion dances?

A: JM: There are very few people here like us. Because we are here... like in Northern Jiangsu they still have some... like what did they have... right, many rural areas have dragon and lion dances. But temple fairs existed before. Temple fairs existed before, now it's been many years without them.

Q: What kind of goods were sold at the temple fairs?

A: JM: Temple fairs sold practical, needed things. The temple fair was held on a set date every year, came once a year. People brought their things to sell. Usually sold special local products. Then you had food, drinks, daily items, just like market exchange.

JM: Now you all shop by yourselves. Now for shopping, you just go out, to the shopping malls.

Q: Regarding your family's diet, are there any food-related stories?

A: JM: Food is definitely better than before. Before, wild vegetables in the countryside, nobody ate them. Now, wild vegetables, people seek them out to eat. Now they look for wild vegetables to eat. Before, if you had no money to buy [other food], you could only eat this meal. Now, conditions are better, they actively seek out wild vegetables to eat. They feel... then they feel it doesn't taste good? Now people have high this, high that, they naturally think about eating them once?

JM: Before, if you wanted to eat well, you had no money to buy. Now city people pay attention to wild vegetables, what season to eat which wild vegetable. Before, we, the locals, didn't eat them. They didn't eat that. They ate... gave it to the pigs. I only fed them to chickens, geese. Nobody ate them.

Q: How long have you been doing this job?

A: JM: Since 2012, right from the start of the development, I've been working here. The work should be relatively easier. Not bad. I see he says he... he's also very proactive, very diligent... that person, right. Usually there are fewer people. This should be... forget it, it's our share anyway. Then others, kids, parents, working overtime at home normally for a few minutes... Like, who are you employed by?

JM: Employed by the subdistrict office.

Q: What do your children do now?

A: JM: They all work outside, none have come back.

Q: They all work outside?

A: JM: Our kids work right nearby, just here in our area, don't go far.

Q: Are there many young people in the village?

A: JM: No young people. It's all old grandmothers running restaurants at home. Some had their houses demolished, the young people live in the resettlement housing. Not many young people.

Q: Compared to before, the roads here are better, right? So these improved roads, what impact have they had on us?

A: JM: Better roads mean better transportation, more developed. Then more people came later. Now transportation is convenient again. But the rural areas before were probably all dirt, now it's all cement. Two cars can pass each other.

Q: Do the roads make a difference to the traditional rural appearance?

A: JM: Now it's definitely convenient. Originally, when it rained, cars couldn't get in. Of course, if it rained, you couldn't go anywhere for a day. Actually, which family in the production team had a car? Now, which family in the production team doesn't have several cars? Everyone has a car.

Q: So with higher income from tourism development, people changed to cars? Next year, with more economic strength, life becomes more settled locally?

A: JM: Before, where were there cars in the countryside? If you could drive a car, it was a big deal.

JM: And cars, every family has one. Which young person's household doesn't have at least two cars?

Q: So, you think that for our rural tourism work, we actually use rural characteristics to attract city people. If our village wants to continue developing tourism successfully in the future, should it continue to emphasize rural characteristics?

A: JM: You definitely need distinctive features to attract people. Without distinctive features, it's useless. Actually, those who come... which of our families has a special dish? So some talk about special features. Anyway, if he doesn't go home, if he doesn't innovate, doesn't he want to find special features to attract tourists? Right?

Q: Do we have things like... as we understand, some places have products... would you like to come back here to do some fruit picking or farming experience activities?

A: JM: We had them before. Before, there was the peach orchard in front. Now the peaches are gone. Originally it was the land of their village community, now it belongs to Niushou Mountain. They developed the peach orchard. Before, it was divided into plots. For example, you bring a child, you rent a plot for a certain amount, you come regularly to plant, and come to harvest when it's time. It existed. It was like leasing a plot of land. One person, one small plot. You would mark out one small plot per person. You bring the kid on the weekend, work on it, then after a couple of weeks, when the peaches are ripe, they can come pick. They did that back then, the first couple of years. Now it's been assigned to Niushou Mountain. They had farming experience activities back then. Now it's gone.

Q: What do you think... meaning, now many guests yearn for rural life. What are the most important aspects of your ideal rural life?

A: JM: It's about the farmland, farming. City people eat Western food, right? Rural people eat.

Originally, it's these aspects that highlight the difference between rural and... you mentioned some distinctions. Right, later, a smaller point, I see we have something about harmony here, right?

JM: Zheng He's Tomb is inside Niushou Mountain. Before Niushou Mountain was developed, even Niushou Mountain itself belonged to this village, to this community. Now it's been delete.

Q: Did this village have any famous historical culture before?

A: JM: Here is Yue Fei's Anti-Jin Fortress, it's counted as being inside.

Q: Do you think our village's tourism development hasn't utilized these resources?

A: JM: Its... the first village development still relied on Niushou Mountain. Meaning this village's development depends on Niushou Mountain.

But like these, for example, Yue Fei, Zheng He, these historical cultural resources, are actually still within Niushou Mountain. We here don't have this kind of history and culture.
