# Supplementary material for: Rurality representation and changes in rural tourism destination
Source: PLoS One. 2026 Apr 21;21(4):e0347226. doi: 10.1371/journal.pone.0347226 (PMC13098982; doi:10.1371/journal.pone.0347226)
Supplement: S1 File — (ZIP) [file pone.0347226.s001.zip › supporting information/世凹村录音及转译文本/jsa20.docx]

Q: What was this place originally like as a countryside? What was it roughly like before?

A: JM An ordinary one, just like this.

Q: This is a remote sensing image of your village. It was like this in 2000, and now it's like this. Can you briefly describe the basic situation for us first?

A: JM Originally, how to put it... every household in the village had enclosing walls. If you were from this family and wanted to visit that family, you had to knock on the gate in the wall to get in. Now, to build a 'Beautiful Countryside', they demolished all the walls. Every household can move around freely.

Q: Has communication between villagers become more frequent?

A: JM Actually, now communication between villagers is less.

A: JM Why? Because originally, before the relocation, it was basically all native residents. Everyone, whether old or young, knew each other. After the relocation, some people moved away. Secondly, after so many years of agritourism operation, many villagers have rented out their houses. There are many outsiders, so people aren't very familiar, which leads to less interaction.

A: Communication between villagers is less.

A: Yes, because people aren't too familiar with the outsiders who came later.

Q: Have relationships between villagers changed? Is it more focused on economic interests now, whereas before it was more about helping each other?

A: JM In this aspect, there is some competition.

A: JM Competition among the agritourism businesses. There's some of that. But it's also related to the fact that communication is less now. Basically, everyone runs their business independently. Actual competition then leads to less and less communication between you. Yes, because if you visit too much, for example, they might think you are copying their dishes or something, right? It happens.

Q: So, that's the basic situation of this village.

A: JM Originally, it was just an ordinary countryside. The roads were simple cement roads. Now they are covered with asphalt. Then, for greenery and environment, there are dedicated personnel for cleaning, and specialized greening departments maintain the landscaping. Originally, it was an ordinary small village with no one managing it.

Q: Originally, its village appearance was actually not bad either.

A: JM The village appearance should have been okay anyway. Basically, every household maintained hygiene uniformly. There was a garbage collection point over there in front. Every household threw all their garbage there. Now, every household has a trash bin.

A: JM Also, originally there was land. Originally, we still farmed for a year. Because our village had very little farmland. Now, basically, earlier, everyone swapped land for social security. Basically, there's no land left. Swapped land for pension insurance.

Q: So, the pastoral landscape has actually decreased.

A: JM Yes. And if every household runs agritourism, they also have no time to farm.

Q: Do you still grow vegetables now? Before, you probably grew your own?

A: JM Now, in our village, basically there is no private plots of land. They were all basically taken back. All used for greening.

Q: So you mainly buy vegetables.

A: JM Mainly rely on the land of surrounding farmers and locals, growing vegetables.

Q: Regarding this matter, what do you think best represents the countryside? You are engaged in rural tourism, right? What is the element that best represents the countryside? Is there something that attracts them?

A: JM Here, it's mainly that when Jiangning started the 'Five Golden Flowers' project, our village's positioning was to provide supporting services for the Niushou Mountain Scenic Area, mainly focusing on rural cuisine. So after nearly 10 years, our Shi'ao Taoyuan's reputation is already established. Basically, 70-80% of Nanjing citizens know about Shi'ao Taoyuan.

A: Mainly rural cuisine.

Q: So, what do you think is the representative element?

A: JM Actually, for visitors, besides the citizens... another characteristic we have here is, since ancient times, it's 'Spring at Niushou, Autumn at Qixia'. Especially in spring, regardless of whether there was agritourism before, when spring comes, urban people, Nanjing citizens, like to bring their whole families to Niushou Mountain for outings.

A: There's history. Historically, there's this habit among urban residents; in spring they would come here.

Q: What image comes to mind when you think of the countryside?

A: JM My impression is that basically, when we were kids, many houses were thatched cottages, earthen walls.

A: JM When we were kids, at our age, in that era, the first thing upon waking up wasn't brushing teeth or washing faces, but taking a dung basket and going around the village to look for dog dung, pig dung. It was like that. Now, as age increases, along with the gradual growth of the country's economic strength...

A: JM Later, the government built roads, water projects, roads reaching every household. From that time on, basically, our countryside began to change quite significantly, completely different from the previous gravel roads, dirt roads. Originally it was dirt roads, then gravel roads, then later changed to cement, then asphalt.

Q: The infrastructure is getting better and better, right.

Q: Do you often go out to watch movies or travel now?

A: JM Movies... basically, I haven't watched a movie in 25 years. Saw one when I was a kid... Back in the collective era, I experienced that... previously called the commune. The commune had a film projection team. They would go to each production brigade to show films in rotation. Now it probably doesn't exist. Doesn't have that now. Now you also have no time.

A: JM Firstly, no time. Secondly, it's mainly no time. Plus, mobile phones and the internet are basically widespread now. Sitting at home, you can watch whatever you want.

Q: Do you travel for leisure?

A: JM Travel... currently haven't this year. Would travel, but not this year yet, mainly due to time. No time, can't get away.

Q: Originally, there were probably fewer cultural and artistic activities.

A: JM The main activities in the countryside originally were watching movies, and watching TV at whoever's house. Mainly watching TV. Now, the feeling of watching TV isn't as enthusiastic as when we were kids. Because when we were kids, especially when shows like 'Huò Yuánjiǎ' were on, maybe several brigades had only one TV set. One or two hundred people would crowd around that one TV, waiting. The owner would turn the TV on.

Q: Are there any particularly impressive customs here from your childhood or that people had?

A: JM Here, actually... one is adults making a new set of clothes for children. Then, when we were kids, because we were poor then... those small red paper cutouts hung above the door, and door couplets. The red paper cutouts were all carved by ourselves. We found a stencil and then carved them ourselves, using a hacksaw blade ground into a kind of pointed knife.

A: JM When we were kids, for New Year, I feel it was a custom where we did things ourselves. Now it's basically all bought.

Q: Now it's basically all bought.

A: JM No one carves them themselves now. Basically, every household... when we were teenagers, we would sit there with a knife, carving bit by bit. It was a custom from our childhood era. Yes, the customs still exist now, but people don't carve them themselves anymore.

Q: It's all commercialized now.

Q: Have the values of rural residents changed in any way?

A: JM Values... over so many years, thinking has become more open. Ideologically more open, exposed to more information. They are more accepting of external things. Before, people's thinking was more conservative. Basically, the villagers are still relatively simple overall. There's no scheming against each other, or doing illegal things for profit. Basically, that doesn't happen.

Q: Do you like the countryside? Would you buy a house in the city?

A: JM I have several houses in the city, but I don't like living in the city. I like the countryside.

A: My child... no, my child is in a big city.

A: JM But personally, I still want to stay in the countryside. Because I was born and raised here, I still prefer the life here. I really like the countryside, accustomed to the life here.

A: JM I identify with the countryside.

A: JM I identify.

Q: You think rural culture is actually something to be confident about?

A: JM I think the pace of life, the air, and getting around here are all suitable for living here.

Q: Meaning the city's pace of life...

A: JM For me, I'm past the age of striving. So of course, I still hope my son continues striving in the city. When he reaches my age, he can come back. This is also a place for retirement.

Q: Has the rural atmosphere changed in recent years?

A: JM The rural atmosphere... how to put it? The communication between neighbors has decreased. Feel like it's not as harmonious as before? For example, if one family had something happen, every family would come to help. This kind of thing basically doesn't happen now. It's relatively rare now; basically, unless you specifically go and ask for help. Not like before, where if someone had something, they would automatically come to help.

A: Now this phenomenon... is less common.

Q: Because of tourism development here, has your dialect improved or changed?

A: JM Mandarin, etc.? Basically, every household, every person has some... closer to Mandarin. Before, basically many people, including now, the way we in Guli speak is considered quite 'local'. Yes. Now Mandarin is more common. Including myself, I'm also speaking Mandarin with him.

Q: Here, when interviewing them, I can basically understand what they say.

A: JM So, our Guli accent is a bit blunt/harsh. So when we go outside, as long as I am from here, no matter if I'm in Guangdong, Shanghai, Beijing, as soon as I hear someone speaking like us here, I know they are from here.

Q: In the countryside, originally, what kind of vehicle did your family have around 2000?

A: JM 2000... in 2000, our family mainly relied on bicycles. Because at that time we were working outside, but they were at home. Yes, at home, my parents basically relied on bicycles. Now it's cars. Life is much more convenient now. Before, you probably rarely went out shopping, right? Yes. Rarely went out. Now the way of shopping has also changed. Basically, online shopping is also more common.

A: JM Regarding housing, your house is now three stories. Originally it should have been one story, a common vernacular house.

A: This three-story... did the government help build it? We say the government... some we built ourselves. The government only did the environment, the exterior walls, they renovated them.

Q: Sacrificial activities should still exist, right?

A: At our Niushou Mountain, the one that remained longest was the February 2nd temple fair. Niushou Mountain has the Hongjue Temple, at the South Mountain Gate location – that's the pond you pass now when visiting Niushou Mountain. After coming down, there's a 90-degree bend. Go up a little from there, that place was the South Mountain Gate in the past.

A: JM Actually, it existed even in the Republican era. At the South Mountain Gate, they held a February 2nd temple fair every year. Now this no longer exists, hasn't for many years. Old people said it existed even in the Republican era... the Republican era is a bit far back.

A: JM Yes. Later, the Japanese mined at Niushou Mountain. The temple... the temple wasn't... it was the Japanese who... yes.

Q: The Japanese mined secretly, right?

A: JM Not secretly. At that time they occupied Nanjing. Wherever there were mines, they plundered them.

Q: So some activities have actually decreased or disappeared.

A: JM Also, for example, we in Guli used to have a temple fair, actually we call it a temple fair, a rural goods exchange fair, on the 6th of the 3rd lunar month. The common people farmed. Every year, through this festival, they would trade piglets, also some bamboo ware, wooden items like Eight Immortals tables... If your family didn't have one, you could buy it at the market. After the market fair, basically, usually there was no buying or selling. Goods exchange fair. Basically, common people raising pigs – didn't everyone raise pigs before? – would go to the exchange fair to buy, get a piglet to raise until the end of the year and then slaughter it. That's how it was.

Q: Can I ask for your basic information? Age? 49. Occupation should be considered self-employed/individual business. Your education level?

A: JM College diploma from outside, studied outside.

A: JM Here, it's junior high school graduation.

A: JM I left here at 19, returned 10 years ago, 29 years? Left here at 19, yes. Worked outside for 23 years. Returned 10 years ago. Your family annual income is over 100,000.

Q: What do you think are the elements that best represent the countryside? Developing rural tourism? What are some typical elements of the countryside?

A: JM I think... I think it's relatively quiet, no industrial pollution, good air. At night, if there's the slightest movement, the dogs bark, very fiercely, continuously.

Q: Sounds of insects and birds.

Q: This is basically the material aspect, the environment.

A: JM In terms of interpersonal interaction, you feel that neighbors basically all know each other. Neighbors know each other, this is a characteristic of the countryside. That's why there's the saying 'Hide a great person in the city' – the difference lies here. If you come to the countryside, if a fugitive comes to the countryside, it's hard to hide. If a stranger comes to which family, everyone knows. If a stranger comes here and hides in a city, in a town, people basically don't know each other, right?

Q: On the spiritual level, what do you think is a representative element of the countryside? How to put it in this regard?

A: JM There isn't anything particularly representative. Anyway, basically, every rural household has WiFi, has internet. Basically, it's very close to the outside world.

A: JM Basic information from outside isn't blocked.

Q: Earlier you talked about your childhood situation. Before, anyway, every household... because before there was no TV, at night... at that time, our village relatively benefited from the iron ore mine at Niushou Mountain. Our village was formerly known as 'Little Hong Kong, the City that Never Sleeps'. Look, when we were kids, catching gamblers... at that time, rural life had few cultural entertainment options, just playing cards, yes, gambling. But now, gambling... you can play openly in mahjong parlors. Before, they had to catch gamblers. The police never entered our village. Because our village, every household slept with the lights on. In other villages, they slept with lights off.

A: JM The police, when patrolling the village, would look at which house had lights on, and the police would suspect that house had people gathering to gamble. When they came to our village, they never, ever came. Because every household slept with lights on. So our village had the name 'Little Hong Kong, the City that Never Sleeps'. No one turned off the lights. Because we used electricity without paying, all from Niushou Mountain's power, together with them. Because when Niushou Mountain operated the mine, the mine tailings flooded our village's land.

A: JM Our village was probably the earliest in Guli to have tap water in every household.

A: JM Spiritually, it's mainly... spiritually, it's basically everyone minds their own business. This thing... the government doesn't organize large-scale events or anything for people to come watch performances. Basically, every household just plays on their phones. Watches TV.

A: JM Originally, was it more simple? Now is it more modernized, more individualized?

A: JM Before, there were still a few households, for example, people who liked to go to whoever's house, those who were eloquent, would go to their house in the evening to chat or something. Now it's basically less. Look at us running restaurants, basically every household... in the afternoon. When we stop in the afternoon, what to do? Gather a few people to play 'Guàndàn' (a card game). Just for fun, no money, play a couple of rounds of Guàndàn, then go home.

Q: Originally, this place of yours... look at the house?

A: JM My original base was red. This is Qingheju. Yes, originally my roof was replaced. I spent 25,000 RMB to replace the roof.

Q: What is your home called now?

A: JM Hetang Renjia (Lotus Pond Household).

Q: Did you used to chat at your doorstep? Does this count as your public communication?

A: JM No, now... before, the place where our village originally farmed was where? The farming area should be this area. Originally, this area was also given to others... originally it was our village's public threshing ground. The threshing ground also had a place to tether oxen, an ox pen. The ox pen was here.

Q: And you might have come here to chat?

A: JM No, that was from childhood. Summer. At that time, there were no fans. No fans. In summer, every household would roll up a mat, spread it on the ground, grab a good spot, away from the grass, where the ground was drier, and enjoy the cool there. Every household spread out their mats and slept there. Every household was like that.

Q: Also in the afternoon?

A: JM Evening, around 7:00, as soon as the sun set, they went over.

A: JM Come back in the evening to chat?... just sleep there and come back the next morning directly. The next morning around 6:00... Look, this place is relatively cooler because this ground has no mountain behind it; behind is a large depression. The wind from over there can blow directly over. So why was that place the threshing ground? Because if you winnowed the rice there, it would all blow away.

A: JM Originally, your public production... our brigade's production team... Team Leader Xie... showing movies was also here. Now, it's basically gone. It's just at the doorstep now, chatting at the doorstep. This place should have changed too. Now houses are built there.

A: JM Originally, there was no this path. This path is newly built. Because this road is an access point for our village. Yes. This Sanbao Road is newly built. Because our village's former private plots for growing vegetables were in this area, taken back. Because originally this village area was all fields, for growing rice. Then there was a lake here. Yes, this one, there were on both sides.

A: This pond was contracted by my family before, called Shitang. That's what it was called originally. Now it's contracted by my family. For fish farming.

A: My family and my younger sister-in-law's family, two households together contracted Shitang. Now it's... Niushou Mountain, Niushou Mountain's Kan Yan Lake was expanded. There are many koi inside now. Originally it was like this. The changes are quite significant.

Q: This photo, I don't know which year. 2003. Zheng He's Tomb should be here. This place is Zheng He's Tomb. This place is Zhenghe Tomb. Some drawings are inaccurate. You should give a clear explanation.

Q: Can you enter Zheng He's Tomb now?

A: Our village hasn't gained any advantage from Niushou Mountain. Now Niushou Mountain is called Niushou Mountain Tianque Cultural Creative Park. Before it was Niushou Mountain Iron Mine. One was a state-owned enterprise, the other is now also a public institution? Because they are now considered public institution staff, public institution. Anyway, it's run by the government.
