# Supplementary material for: Rurality representation and changes in rural tourism destination
Source: PLoS One. 2026 Apr 21;21(4):e0347226. doi: 10.1371/journal.pone.0347226 (PMC13098982; doi:10.1371/journal.pone.0347226)
Supplement: S1 File — (ZIP) [file pone.0347226.s001.zip › supporting information/世凹村录音及转译文本/jsa21.docx]

Q: What has been the biggest change brought to this village by tourism development in recent years?

A: Mainly changes in the village's appearance and environment due to the overall development. Also, an improvement in the villagers' quality of life. We rely on the development of Niushou Mountain. If Niushou Mountain attracts more visitors, we get more people here too.

Q: How was the tourism development here initiated and managed?

A: We organized meetings with villagers to understand their willingness to participate. We took them on study tours to other places (like Chengdu) to see successful examples. Initially, there was resistance as they were farmers unfamiliar with business concepts. After the tours and with government support (policies, funds, help with business licenses, chef training, promotional efforts to attract tourists), their businesses gradually started. The government invested in the overall development: building renovations, exterior facade upgrades, demolishing auxiliary sheds/walls to reduce building density ("subtraction"), increasing greenery and infrastructure ("addition"), enhancing local culture ("multiplication" - local cuisine, folk culture like "Guli Mingge", Buddhist culture from Niushou Mountain, Zheng He's culture), and creating a one-stop service center combining community and tourist services ("division"). This is our "Beautiful Countryside Characteristic Operation Method".

Q: How has the tourism offering evolved?

A: It started as version 1.0: mainly rural cuisine . Then version 2.0 added tea houses and homestays. Now version 3.0 incorporates cultural industries like wellness culture (Traditional Chinese Medicine), Guoxue (Chinese classics) lectures, and a RV camp introduced in the last two years to enrich the formats.

Q: Who manages the tourism here?

A: Primarily our community manages it, unlike other tourist spots run by specialized companies. It's community-based territorial management Cleaning and landscaping are outsourced, but general management and promotion are done by the community ourselves. Staff are mostly locals.

Q: What was the village like before tourism? What did people do?

A: Before development, it was quite poor. Most young people worked as migrant laborers in Nanjing. Now, many have returned to start businesses. Some are university graduates who came back. Their operations are different from those of older villagers.

Q: Are the businesses run by locals or outsiders?

A: Mostly locals. We encouraged locals to operate. If they couldn't or didn't want to, they could choose relocation or rent their houses out. We helped find tenants. There are a few outsiders who rented houses to run businesses, like one homestay.

Q: How many tourism businesses are there now?

A: Currently 17 agritourism restaurants and 2homestays. There used to be more (around 23-24), but some closed down or transformed as they couldn't sustain the business.

Q: What is the tourist volume trend since development started in 2012?

A: The peak was around 2013-2015, with about 500,000 annual visitors. It has decreased somewhat since, now around 300,000-400,000. This is due to increased competition from other "Beautiful Countryside" projects and maybe lack of new attractions.

Q: Where do the tourists come from, and how do they travel?

A: Mostly from Nanjing and surrounding areas like Ma'anshan (Anhui province). Both self-drive and tour groups. Many also come on foot. Public transport access is okay.

Q: How is the relationship between villagers and tourists?

A: Generally positive. Tourists' quality has improved compared to the early years when we struggled with traffic and order. Now it's more regulated. Many are repeat customers. During the pandemic, businesses relied heavily on regulars. Relations are good.

Q: How has tourism impacted villagers' lives?

A: Incomes increased significantly (some households earning 400,000-500,000 RMB/year pre-pandemic). Lifestyle changed from traditional rural to more urbanized – many bought commercial apartments in town. Overall quality of life improved.

Q: Are there any historical or cultural elements here?

A: Yes. Zheng He's Tomb (an cenotaph) was nearby. The descendants of the tomb guardians lived in a small village (Zhengjia Village) which has been relocated. Niushou Mountain has sites related to Yue Fei's battles against the Jin, including defensive fortifications. Our village's name evolved: it was originally "Dashi'ao" (Big Corpse Gully), later changed to "Dashi'ao" (using the character for "master/strategist", implying military garrison), and now "Shi'ao" (World Gully), reflecting a more peaceful era.

Q: What do you think is the most representative element or main attraction for tourists here?

A: The rural cuisine is the main draw. People come for the good, authentic food. Experiential elements related to farming culture are less prominent now. We previously had a pick-your-own farm area in the peach orchard, but Niushou Mountain took over that land. So, currently, it's primarily about the food. The harmonious coexistence with nature is also an important factor.

Q: Is farming still practiced?

A: Yes, but not much. Land is scarce. Locals generally don't farm much as it's hard work with little profit. Some vegetable greenhouses are rented to outsiders. There might be small tea gardens on the hills, but overall, agriculture is not the main focus anymore.

Q: Has tourism made the place more orderly?

A: Yes. We followed the principle of "Planning First". We made a plan first, then carried out construction and renovation accordingly-houses, greenery, infrastructure all according to the plan.
