# Supplementary material for: Rurality representation and changes in rural tourism destination
Source: PLoS One. 2026 Apr 21;21(4):e0347226. doi: 10.1371/journal.pone.0347226 (PMC13098982; doi:10.1371/journal.pone.0347226)
Supplement: S1 File — (ZIP) [file pone.0347226.s001.zip › supporting information/世凹村录音及转译文本/jsa3.docx]

Q: So, it was quite severely affected by the pandemic?

A: JM: Mhm, right. No business even on weekends.

Q: Not great. Do you think it might get better tomorrow?

A: JM: Don't know.

Q: What do you think about the tourism development here? How has the situation been in the last couple of years?

A: JM: This year is definitely at a loss.

Q: Not good, huh? The pandemic impact was too severe. Where do you think the tourists who come here are from?

A: JM: More are from out of town. People from the surrounding area, if they've been here before, they've been. People from here, once they've been, they don't come again.

Q: What do you think is the biggest attraction for them?

A: JM: The attraction... this place is the natural scenery. Here it's the cusine. Spring Festival is relatively good. No matter how you plan it, it's no use. Thediscuss wasn't handled well.

Q: Right, you said the government suddenly stopped supporting it here, right?

A: JM: Right. Now it's not working. You can open, that's fine. But the government, well, they just gave you the direction. Said in previous years there would be some training and such.

Q: Are they still supporting us?

A: JM: The last couple of years, seems like no reaction.

Q: What did your family do before opening the agritainment business?

A: JM: Farmed the land.

Q: And now? Do you still have land?

A: JM: No, the government took it all back. Now they built... 33 thousand? 30 thousand? You can run agritainment. If you don't run a business there, they don't give it to you.

Q: Is it rented out to others? Are there still many locals running agritainment here? There are outsiders running them, right?

A: JM: It's generally about half and half.

Q: Generally, do locals run better businesses or outsiders?

A: JM: Locals... whether the food is good or not... outsiders basically have to ask me... Xihu Li Road... if it's no good, they just leave, forget it. The average is two seasons: one spring season, one autumn season. Summer and winter are no good.

Q: Roughly how many people?

A: JM: During the peak season, when people go up the mountain to play for a while... quite a lot come with tour groups.

Q: Do the agritainment businesses hire people, or do we manage it ourselves?

A: JM: Some hire people, some do it themselves.

Q: I see your house here is being renovated, is that right?

A: JM: Yes.

Q: I see the exteriors here all have a unified style. This was done by the government. Compared to the old rural look, what do you think? Did it feel more rural before, or did those old buildings feel more rural?

A: JM: Anyway, I didn't pay much attention. But it's much nicer looking now, right?

Q: The village appearance has improved. I see a lot of greenery in the front.

A: JM: That was all done after the tourism development.

Q: Right, the government worked on the basic house structure, we just modified our own previous houses.

A: JM: Just modified our own houses like this. The exterior work they did, the backing, whatever they put in, is still quite pretty.

Q: I see. Regarding your family, did you make any changes specifically for tourism? Those companies, including renovations?

A: JM: If it's your own house, whether you manage it yourself or rent it out, you're preparing this thing... definitely aiming for a somewhat better environment.

Q: So compared to before tourism, the environment in the whole village and at home is better.

A: JM: Definitely much better.

Q: Including greenery, transportation.

A: JM: Yes, right, right, right.

Q: I see you're still using the old-style...

A: JM: Ah, right, this is an old-style clay stove. Cooking with the clay stove is fine.

Q: Do you still use it now? Is it also one of our features?

A: JM: Yes.

Q: What's the difference between the two, in your opinion?

A: JM: Using this for stir-frying is too slow.

Q: For stewing? Cooking rice, stewing dishes.

A: JM: You generally don't use it for stir-frying. You use it for cooking rice, large pots of rice. Eating large pot rice is fine too.

Q: How have the tourists been since we started the agritainment? What was the peak number? Like, when they came to our place to eat?

A: JM: Spring peak business was very good. Just one season, then it's no good afterwards.

Q: For example, what do we do when it's not busy?

A: JM: There's a little bit of wasteland opened on the surrounding mountains, we do some farming. Usually not much else to do.

Q: Before opening the agritainment, when we were farming, what did you do in your free time? What leisure activities did you have amongst yourselves?

A: JM: Usually those who have jobs are working. Mostly everyone in the family has jobs, basically one or two people at home. Usually not many people are maintained... either the family hires one cook, or the old man/old lady stays home. Young people all work outside. During busy times, they can come back at noon or in the evening to help out. Because when it's especially busy, you add the aunts and uncles, and they are there helping, that's how it is.

Q: What leisure activities do you have now?

A: JM: Usually when idle, not much to do. Some people play cards. Basically, not much... it's the countryside, right? Visiting this house, visiting that house, playing Guangdan (a card game?), that's it, right?

Q: Us too. But these vegetables are bought, right?

A: JM: They are basically grown by the farmers themselves. If not enough, then buy from those old ladies over there.

Q: These cured meats, are they like this? Made at home?

A: JM: All cured ourselves.

Q: I heard there used to be dragon lanterns here before?

A: JM: Seems like there weren't any here.

Q: So, there was very little land before, right?

A: JM: Before was similar, there was some, haven't seen any now. During holidays, you should let people see... seems like a few years ago they had temple fairs, you could still see them. The last few years, seems no reaction.

Q: These festive folk customs have become fewer.

A: JM: Don't really feel it.

Q: So compared to before tourism, this festive atmosphere is weaker.

A: JM: Mhm.

Q: What about neighbor relations? For example, my relationship with my neighbor.

A: JM: Neighbor relations, rural neighbor relations are just like this, right?

Q: Is there any competition or...?

A: JM: Competition is very normal. After opening agritainment yourself, there will be competition. You know the saying, 'those in the same trade are enemies', right? It's very normal.

Q: But before opening, when we all farmed, it was probably better, right?

A: JM: Rural areas are like that too. You are also from the countryside, you know what rural areas are like. Then there's a bit of envy, very normal. Right.

Q: Do you interact much with those outsiders? Like people from other places who also come to open shops?

A: JM: It's like sometimes the government organizes them to come.

JM: Whether they interact or not, this kind of thing... they just come here to look, look at the style here, eat at the big hotels here, that's it. What interaction do they have with us? Right?

Q: What about interaction with customers, the tourists coming from outside?

A: JM: With tourists, interaction is definitely more, for your business.

Q: And we probably have quite a few regulars, repeat customers here.

A: JM: Our family was the second one to start here. Very many. The key relies on repeat customers. If not relying on repeat customers, then the business is no good, won't last.

Q: Do you think these tourists coming here to eat, besides increasing our income a bit, have any other impact on us? The outside tourists, because of, for example, their lifestyles, way of dressing, speaking, has it brought any influence to you locals?

A: JM: Now, not really.

Q: Like locals' Mandarin has improved?

A: JM: Still speak the local dialect.

Q: For example, after starting the agritainment, income increased. After income increased, compared to life before, has your lifestyle become a bit more like an urban lifestyle?

A: JM: Mhm, pretty much. Now, meaning, rural people definitely want a better life. Then all aspects of life are changing. Can't still be as rustic as before, right? Overall, it's developing for the better, right?

Q: Meaning the overall quality of life has improved somewhat.

A: JM: Right, right, right. Running agritainment is definitely very... for the local common people.

Q: But perhaps with tourism, our previous farming lifestyle has changed greatly.

A: JM: Definitely. No need to say.

Q: Which way do you think is better?

A: JM: After all, we've lived here for a long time, don't really feel it. But overall, it's definitely better than before. Better, no question.

Q: Do we sometimes miss the quieter life before?

A: JM: That... seems like you don't feel it.

Q: Our main focus here is rural tourism, right? What do you think your ideal rural village should be like?

A: JM: Rural village... no particular feeling. Anyway, overall, this place is not bad.

Q: What problems or shortcomings or areas needing improvement have you noticed in our rural tourism, in our area here?

A: JM: Areas needing improvement? Like what?

Q: I mean, for the overall rural tourism development here, what do you think should be improved?

A: JM: If the government could support, that would be best. Without support, now you are left to fend for yourself, right? And now, previously all aspects... the West Gate isn't open either, very hard to survive.

Q: So, do you think there are some things here, like rural farming culture or something, worth developing to make our tourism more attractive? For example, could we set up some fruit picking, or some farming experience activities? Do you think that would attract tourists?

A: JM: This definitely would.

Q: Are there such projects here now?

A: JM: Seems like here... anyway, the government, we aren't clear. And truly reaching us, reaching each farming household, seems there aren't any.

Q: Actually, our development here mainly relied on the government managing it well before, now they don't care.

A: JM: Now, the specifics, we aren't clear either. But the last few years, definitely no support.

Q: Have there been any changes in customs like weddings and funerals here? The wedding/funeral aspect.

A: JM: Still the old ways.

Q: If there's a wedding in a family here, will neighbors help out?

A: JM: Definitely need to help. It's like every household participates in welcoming the marriage. People from all areas go to help. This thing, it's just according to human decency.

Q: Right. We were previously agricultural registered residence, right? Now it's urban, urban residence.

A: JM: Right, right. Now it's 'land for security', the government took the land back. Then, based on that, rural residence became urban residence.

Q: Do you think rural residence is better or urban residence is better?

A: JM: Rural residence has its benefits. For example, if there's demolition, the compensation is different, etc. But being urban residence now... the distance... what policies the government has, we aren't clear. 'Land for security' still has benefits. All aspects are more convenient, right? More secure, better.

Q: Right. What do your children do now?

A: JM: Work. Now all work outside. Right, all work outside.

Q: Do they support us... us running the agritainment?

A: JM: Support.

Q: Are the young people here mostly outside?

A: JM: Right, young people are basically all outside.

Q: So young people still aren't coming back to do this?

A: JM: There are, very few. The one in front called Hetang Renjia, he was developing in Shenzhen. He's young... anyway, meaning 10 years ago, annual salary over 100,000, should be pretty good, right? Later he came back... his operation is doing very well, much stronger than a job, many times over.

Q: What's the approximate annual income here, for example?

A: Before it was okay, the last few years are no good.

Q: How much was it the year before last?

A: JM: A few years ago, anyway, every household after running this, bought houses, bought villas, it's true. But the last two years, if you started only recently, definitely not good.

Q: Roughly how much is the income per year in the last couple of years?

A: JM: Just getting by, roughly... if this house is rented out to others, 100,000 to 200,000 plus is possible. He can only make the rent money. Just managing to get by. If it's an outsider renting here, it's a loss. They also have servers, cooks... they all lose money. Basically can't sustain, so more of them are leaving. Right.

Q: For example, we do the cooking ourselves. Before tourism, for example, did we already have the skills, or did they specially train us for tourism?

A: JM: The government trained people earlier. Trained in cooking or management. Yes, they had this for this purpose.

Q: So our own cooking skills have improved.

A: JM: Basically, now, after doing it for a long time, you start... you should hire a cook, hire a cook. Later, if business is bad, you do it yourself. Basically, you learn a bit from colleagues, learn for a year or two, you can basically manage a bit.

Q: So your own cooking ability has greatly increased.

A: JM: Definitely, right? Even just by watching, you learn by watching.

Q: Have prices changed here? Without reason? Because generally in Chinese habits, things at scenic spots are more expensive. What do you think about the prices here?

A: JM: Overall, the scenic spot resources are okay. But everyone... I am investing at his place, I've worked there for 7 years. I'm not from his family, otherwise I wouldn't say this. His place... the dishes are still reasonably priced. You said the boss is a Party member, chairman... chairman of the Jiangning District Catering Association. Anyway, you found the right place, found the right people. He's retired.

Q: Where are you from?

A: JM: I'm from Huaian.

Q: Later you got through to him, right?

A: JM: Right.

Q: How many years have you been here?

A: JM: I've worked here 7 years. Quite a long time too. So I've witnessed this village's development.

Q: So you mean you specialize as a cook?

A: I'm a professional.

Q: JM: Very skilled. How old are you roughly?

I'm 46.

Q: JM: Very impressive. Okay, brother, how much schooling did you have?

I have a junior high school education.

Q: JM: Anything else? No more questions? Okay, thank you. Sorry to disturb you for so long.

Okay, bye-bye.
