# Supplementary material for: Rurality representation and changes in rural tourism destination
Source: PLoS One. 2026 Apr 21;21(4):e0347226. doi: 10.1371/journal.pone.0347226 (PMC13098982; doi:10.1371/journal.pone.0347226)
Supplement: S1 File — (ZIP) [file pone.0347226.s001.zip › supporting information/世凹村录音及转译文本/jsa4.docx]

Q: With rural tourism development over the years, there have been significant changes. In your impression, what are the biggest changes? What is the biggest change?

A: JM: The biggest change... what changes are there? The roads were upgraded, and there are activity spaces now. What were the activity spaces before? Where could people do fitness activities? The brigade/village committee area was developed, for people to play basketball and such. So there are some public activity spaces now. Right. Originally, there weren't any.

JM: Originally, there were public areas for drying rice here. Now, your evening activity is going to the square for a walk after dinner.

Q: What was the original rural village like?

A: JM: The original rural village, how could it be as good as now? No, it definitely wasn't.

Q: But how was the original village not good?

A: JM: No proper roads, no paved roads. It was bad. It was gravel roads, definitely no asphalt like this.

Q: What else?

A: JM: What else... I married into this village, I don't know either. I've been married here for twenty-thirty years. When I first came, it was like this.

There was farmland.

JM: Before there was farmland, now there isn't. Now it's all expropriated land. I don't need it anymore either. Originally, for buying vegetables... we used to grow some ourselves, because before everyone grew their own. Now, if you have some land, farmers can still use the edges and corners, you can grow stuff. We can plant. Now it's a bit less, only enough to eat. Do we still buy vegetables? Yes, we buy vegetables. Chickens, ducks, all have to be bought. Now you're not allowed to raise them either, right?

Q: You probably rarely ate out before, right?

A: JM: Definitely. Now we have our own restaurant, can't go out, basically don't go out. It's less, right?

Q: The quality of family life originally, for example, you probably had fewer household appliances.

A: JM: Before, where were there air conditioners? No air conditioners, only electric fans, nothing else. Now the conditions are a bit better.

Electric fans... original cultural/recreational activities were probably fewer. Now we have people, originally there were none. Now we have them, now, now we often have them, like elderly activities, this and that kind of cultural performances. Public welfare activities, now there are many of these performance public welfare activities.

Q: Regarding religious beliefs, originally, did this place have clans? Your family...?

A: JM: Now people believe in that, what's it called? Church. The church is Christianity, definitely Christianity. Anyway, we don't go. There's a church over there. Now it's actually more free, right? Originally, there probably were... now it's all public spaces.

Q: Did you buy an apartment in the city?

A: No, we are... the old people were relocated. Relocation housing for demolition. No apartment [bought]. You seem to strongly identify with this rural identity?

JM: Definitely don't want to go to the city either. Those few kids... we don't want to go to the city. Whether these kids buy later or not, we don't know. We definitely don't want to go. Just like this, living in the village is good, we're used to it. Right, nothing bad about it. In the afternoon, play cards, do business if you have business, just have fun.

JM: Our home is on the 5th floor. After climbing to the 5th floor, you don't want to come down. Once up, don't want down; once down, don't want up. That tires him out. Is there an elevator? Right.

Q: This area is developed for rural tourism. What elements do you think best represent the rural village, in this aspect?

A: JM: The original rural village had a lot of farmland. Now it's basically all gone now. The original rural village had very good water quality, that situation, now it's gone too. Originally there were more trees. Not like now they plant flowers and grass, no big trees. The big trees were all cut down. Originally they planted flowers and grass themselves. It's because of the landscape renovation, so they definitely cut what needed cutting. They did some tidying up, that's about it.

Q: Was your house originally like this?

A: JM: Originally it was like this, didn't have this thing. The government developed it a bit, they added a horse-head gable. Basically the same for every household, just added a horse-head gable. The horse-head gable was uniformly added by the government. Right, these houses, these farmhouses are all ours. It's just that they uniformly did the development once.

Q: From the perspective of behavior and habits, did the original rural residents live at a slower pace?

A: JM: They were more idle then. Now they are more idle.

Now, what do you think farmers can do now?

JM: Originally, if you farmed, you'd be busier, have things to do, would be very tired, life would be very tiring. And the conditions, food was not good either. Definitely, you ate poorly, and lived relatively worse. Now it's definitely better.

Q: The wealth gap originally probably wasn't very large either.

A: JM: Originally it was large. Originally it wasn't this good. Originally people were poor. Some poor, some rich. People in the past were better than now? In the past, there weren't so many economic disputes.

Q: Actually, it's different from the natural development of your rural village here, because the Hui-style... do you agree with it?

A: JM: Agree or not... they made it quite nice-looking. Think it's good-looking, definitely better looking than before. Can't deny it looks good here. Looks better than before.

Q: How is household waste handled? How was it handled originally?

A: JM: Before, didn't you just dump it? No one managed it. Now the places have waste sorting. Now it's sorted. And then one is... without any procedures.

Q: What about here... because I heard many are outsourced, not operated by locals. Do you think outsiders coming here have a big impact on you?

A: Each does their own, each minds their own business. Willing to come eat, then come eat. We don't call out [to customers].

Q: Don't call out, right?

A: Don't call out.

Q: Do you interact with them much? Interact much?

A: Don't talk with them either. They are also from out of town, from Xinjiang.

Q: How did they get here? Do you know how they roughly connected? For example, if I want to rent my house out, do I go through the village committee to connect, or do I find people myself?

A: They found it themselves, contacted themselves.

Q: Has the local dialect changed because of running agritainment, so your Mandarin is better? We originally speak Mandarin, the dialect isn't very strong, everyone can understand the dialect. Originally good.

Q: Are there still any religious sacrificial activities? Are there any religious sacrificial activities?

A: We don't have any.

Q: Tomb-sweeping at Qingming Festival, that's always existed, right?

A: That definitely exists, always has.

Q: Have the rules for weddings and funerals been simplified? Any changes compared to before?

A: Before, where were they so grand? Now they are not grand? Money now... where was it like this now? But before, there might have been some rituals that were more cumbersome. Right. But it might have been more grand.

JM: Now it's because of tourism development. Before, the road we used to take is blocked off for them. You, Niushou Mountain, you develop your scenic area up there, you blocked the one road us farmers used to take. I saw that road is blocked. Before we could go up the mountain. You probably aren't using this road. We could go directly to Hohai Universitythere, directly to Jiangningthere, this road. This road was blocked by them.

JM: It's because of tourism development. You can open a road for how to pass, right? This point is not good. Our farmers' road was taken by you. Right? You could open one next to it for us to pass directly, right? We aren't going up your mountain to play anyway, right? You took our road, you could build a road for us to get through.

Q: What do you think the ideal rural village should be like? You are developing rural tourism, right? The rural aspect is the main reason attracting tourists, because you are rural.

A: JM: The rural village now is developed half-baked, then they stopped, dropped it and don't work on it anymore. Look, you build some amusement park, build supporting facilities for kids to play, build something, right?

JM: Okay, we say now can also be... the previous two years, previous years... now basically no people. Those who come to eat, come to eat. Those who don't, don't come.

Q: Fewer people coming to eat?

A: JM: Shitang Zhuhai... they all go to Shitang Zhuhai. Because they developedthere better than us here, Shitang Zhuhai. You haven't been? No Shitang Zhuhai? Thatthere, and Jijiayuanzithere, developed very early, flowers and grass, incredibly beautiful. Loads of people go there to play. Meaning the follow-up supporting facilities here didn't keep up. Including the road here not being opened, this scenic area gate not being opened. So it should have a relatively big impact on your business.

Q: Your village's reputation is actually quite high, because it's connected to Niushou Mountain.

A: JM: We farmers originally could get to Hohai Universitythere in about 10 minutes. Now we have to go from Donghang Bridge, go around from Dalian, have to detour half an hour for us farmers to get through.

Q: During the pandemic, were there many people coming down? Did many people come here to eat?

A: JM: Not as many as when there was no pandemic. If the West Gate hadn't been closed before, more people came here. Now it's closed, basically very few. Nothing much.

Q: What is the rural atmosphere like? Is it peaceful and harmonious?

A: JM: The atmosphere is still that way. Square dancing, it's really lively.

Q: What was it like originally?

A: JM: Originally it should have been... relatively quieter. Now it's more lively.

Q: Length of residence?

A: JM: Let me see, I was 26 when I married here. Should be 18 years.

Q: Annual income from participating in tourism?

A: JM: 100,000 yuan.
