# Supplementary material for: Rurality representation and changes in rural tourism destination
Source: PLoS One. 2026 Apr 21;21(4):e0347226. doi: 10.1371/journal.pone.0347226 (PMC13098982; doi:10.1371/journal.pone.0347226)
Supplement: S1 File — (ZIP) [file pone.0347226.s001.zip › supporting information/世凹村录音及转译文本/jsa7.docx]

Q: What do you think is the main reason that attracts tourists here, including those from Taiwan? Or what do they come here to experience?

A: JM: The Beautiful Countryside.

Q: What do you think is the most important aspect of this rural area?

A: JM: It's theoretically... Buddhist theory... state religion... (Response seems unclear or fragmented).

Q: What are some of the distinctive features or characteristics of our rural area here?

A: JM: Like now, they've done beautification and upgrades, you know, the horse-head gables, the new rural corridors. These didn't exist in the old countryside.

Q: Do you think the rural environment and atmosphere here are reasons that attract them?

A: JM: It's hard to say for the visiting tourists. He doesn't encourage... Nobody... Personal opinions differ. Some feel the current countryside is different from before – it used to be dirt roads, now they are paved roads, cement roads. The environment is different now. You can't farm like before... now it's unnecessary, the land was taken away. The land has all been acquired.

Q: So no farmland now, right?

A: JM: Life is better now. For us elderly, it's definitely better than before. We had such a hard time when we were young! It's incomparable to now.

JM: Now, rural people dare to say, you can eat whatever you want. Before, you ate what was available through their cooperation... What did we eat? When we were your age, it was tough. Working in the fields every day, carrying river mud, pumping water drums... You probably don't know about these things? Where is the reservoir?

Q: What would you say is the biggest change after developing rural tourism?

A: JM: The biggest change in the county now... still compared to state-owned enterprises? (Response seems unclear).

Mainly in your aspect.

JM: The houses changed. Now this place has changed too. Lifestyle has changed. Where you live now... before, going to Nanjing required a long trip. Now, with this... a bus, a phone call, a car comes immediately. Every family has private cars, which family doesn't? Before, if a family had a bicycle, they were extremely happy. That meant they had money. Now, families have two or three cars. There are just too many of these things now.

Q: The way we earn money and this lifestyle is a bit more like the city, right?

A: JM: Now it's transformed into what's called rural urbanization. Isn't it all like this now? These houses... Before, what could ordinary people eat? Now with refrigerators, air conditioners, color TVs... Back then, having a black-and-white TV was a huge deal. "Wow, his family is rich, they have a TV!" Now they talk about big TVs, tens of inches. Before, a 9-inch small TV cost 12,000 and was incredibly attractive. Current conditions can't be compared to the past. Now, everything has improved beyond recognition.

Q: Are there still many tourists?

A: JM: Usually not many people. It's crowded in spring. Our place is crowded in spring. Our old saying goes: 'Spring for Niushou, Autumn for Qixia'. Niushou means...? Autumn is for visiting Qixia. Anyway, when autumn comes at Qixia, with those things like fiery leaves... now things by the roadside, actually everyone says it's not novel anymore. We have a lot here too. We usually work here. Living conditions now can't be compared to the past. Look, now you can eat whatever you want, it's all available. Wear whatever you want...

JM: Before, if you wore foreign brands, people said you were rich. Now, families with a little money all wear name brands, all dressed in name brands.

Q: You mentioned these name brands... do the outside tourists influence us? Do we see their lifestyle and then learn from it?

A: JM: Lifestyle... it's not about learning. In our home, now... different places have different customs and habits. Now here, we feel the customs and habits are different from, say, Hubei there. Customs and habits... they live in their own areas. He says... clothing, food... now it's basically balanced. Unless you're in a place with slightly worse conditions, there are more good places now. Public security is much better now.

Q: So the overall environment has been greatly upgraded, right?

Q: Do you think the current living environment matches your ideal rural life?

A: JM: Comparing current life and ideal life... it's still hard to describe clearly.

Q: Are you satisfied now?

A: JM: I'm relatively satisfied. For us elderly, we are definitely satisfied. For you young people, it's hard to say, right? Some of you have jobs right after graduation, some don't after graduation. The levels are different. Some go to university... feeling a prestigious brand is also university.

And look, when you go... people are in school studying. Now the ideal... looking with our current perspective... is just living life. Your generation is different again. What did our generation have? For our generation, it was about clothing and food. So, not farming the land, modern people are much happier. Now they say the Communist Party's benefits are like this, like we found... we still get a few hundred yuan a month. At our age, where would you get money before? Now, for you young people coming up, life is easier. Why?

First, as I tell you second, I don't ask others for money. With a few hundred yuan, I can buy what I want to eat. If in a few years you have no income, what will you eat? You have to ask your youngest son. Children's families can't always have money either, they also have times without money. Current living conditions are definitely much better than before.

Q: Like in the past, in our imagination, everyone would do farm work together. What do you think is a major characteristic of the current countryside?

A: JM: Before, it was all human and animal power. Now it's all machinery. Now, like over there, if the fields aren't harvested... that piece of plowing is all done by machine. Planting rice is done by machine.

Could we have imagined this step before?

Right.

JM: Now, you talk about spending money every day? Getting a few hundred every month? In our past, how many days did you have to transplant rice?

JM: Before, transplanting one mu of land per day was fast. Now one machine transplants dozens of mu per day.

Right. Talking about current society compared to my past, it's incomparable, can't even describe it. Like us old folks say... Before, our family... back then we... we talked about realizing 'electric lights and telephones, upstairs and downstairs'. Where did that come from? Now we have it all. Electric lights, telephones... now people without landlines have mobile phones, far exceeding that. Meaning if you look for me, I look for you... if you have this phone number, you can find me anytime, handle any matter, or tell them to come back for a meal, just one phone call, "Okay, see you later." In our past, if you needed to find someone for something, you had to run, and it took half a day. You run for half a day, and they might not even be home. In summer... now with mobile phones, as soon as the phone rings, you pick it up immediately.

Q: What kind of leisure life do you generally have here now?

A: Take walks, dance square dance, play Guandan (a card game).

Q: What leisure activities did you have before?

A: JM: Before, wake up in the morning, open your eyes, eat breakfast, work on your one point three mu of land. After dinner, think about what? Also that one point three mu. Now, for your generation, work during the day, if asked to work overtime at night, they grumble. You didn't dare back then. You earned work points, only by working could you get money. In our time, working a whole day earned 1 mao 9 fen. Now you say you wouldn't even pick up a 1 mao coin dropped on the ground. Would you young guys pick it up? A 1 mao coin on the road, would you pick it up?

JM: Times are changing. Back then, our family income was 19 kuai 8 mao 8. How to explain now?

Now it's red bills, 50s. In our time, the biggest bill was 10 kuai. You couldn't find anything bigger, there were no bigger bills.

Q: Now we actually rarely use cash, right?

A: JM: Young people don't even carry money now.

Q: Right, I didn't bring any money.

A: JM: Like us back then, that stuff couldn't happen, could only use cash. You go buy something, directly take out the phone, scan a code.

JM: Back in 2012 when we just started the agritainment business, cash payments were more common then.

Back then it was basically all cash payments.

From when did people stop using cash?

JM: Later, gradually, slowly, everyone stopped using cash.

Q: So it's related to communication and network technology.

A: JM: At the beginning, few [used mobile payment]. Back then, few. Generally, people couldn't use the things you play with now. Like my niece, teenagers, niece, going somewhere, "Mom, give me the phone, I'll buy that thing."

Q: Do we use this scanning payment method now?

A: JM: Now, for people like us, we're old, elderly, illiterate. If you're illiterate, you can't use those things. We feel... the phone can only answer and make calls. For example, using WeChat... anyway, everyone uses internet stuff now. Other times, we take cash to buy things.

Q: For example, when tourists come here to eat and pay by scanning, is there someone else watching? (Likely meaning managing the transaction)

A: JM: Don't need to watch those. Anyway, my daughter and them have WeChat, the WeChat is theirs. Anyway, if you eat here today, after scanning to pay, it goes to their phone. Our old folks' phones don't have it.

Q: After tourism development here, have relationships with neighbors changed? Not as good as before? Or about the same?

A: JM: Now, fundamentally, no relationship. I'd say it's still normal, about the same. Why say it's about the same? Now, with every family's walls built up, that area is yours, this area is mine. There's no normal interaction anymore. No farming, no raising chickens or ducks. Back then, arguments were about your pig ate my chicken, this chicken ate my vegetables. That doesn't exist now. Now people have flowers and grass. If you like it, you plant it; if you don't, you don't. If weeds grow too long, there are maintenance personnel. If the grass at your door gets deep, there will be a team of people. For example, the Beautiful Countryside construction has a team managing these things. There's no reason for disputes, no basis for arguments. Now, people just talk less. Why talk less?

JM: You ask me to describe the next dish, I think... he goes out to work, he goes out to play. Now, generally, couples... friends eat breakfast in the morning, after dinner in the evening they might visit me, stroll around the village. That doesn't happen now. Because now, look, we cook in the evening for them to come home and eat. After eating, we wash dishes, watch TV if we want, go play cards if we want. Those things don't exist. For example, we often play Guandan outside. After dinner, as soon as we finish eating, a few people know and gather at your house. After eating, they go to your house.

Q: Now people's awareness is higher. Even on the bus, if someone slightly older stands up, immediately a young person will offer their seat. Often that seat is empty, but they still stand up for us elderly. It's the mindset. Yes.

JM: Thirdly, progress. But the mindset is definitely much more progressive than before. Now we also argue less with elders/seniors. Why say that? (Interrupted)

Q: May I ask how old you are?

A: JM: 60.

Q: Roughly how much annual income does your family's agritainment business generate?

A: JM: Roughly 50-60,000 yuan a year.

Q: Where are the tourists here from?

A: JM: The tourists come from all over. There are from Taiwan, also from Fujianthere, all over.
