# Supplementary material for: Rurality representation and changes in rural tourism destination
Source: PLoS One. 2026 Apr 21;21(4):e0347226. doi: 10.1371/journal.pone.0347226 (PMC13098982; doi:10.1371/journal.pone.0347226)
Supplement: S1 File — (ZIP) [file pone.0347226.s001.zip › supporting information/世凹村录音及转译文本/jsa8.docx]

****Q:**** Are many people from outside renting houses here?
****A:**** JM: Yes, a large portion are rented by outsiders. There are people from Qinghai, Sichuan, Inner Mongolia, from all over. Some original operators couldn't sustain their businesses, so they rented the houses out. Others just rent their houses out for others to run; they couldn't manage it themselves.
JM: Sometimes they aren't very good at business. Also, there's no promotion now. When they first started, there was promotion, but now there isn't any.
JM: Initially, there might not have been proper screening of managers. Later, after a couple of rounds, big data was used... management personnel were assigned here through big data to achieve results, complementing each other somehow. Secretary Li? After Secretary Li came, he focused on performances. They introduced university students here, just a few hundred meters away. Some did promotional work. This place was very bustling back then, famous far and wide. Then they were transferred to Guli Subdistrict, some even went to Huligong... they became part of Huli now. There are too few management personnel now. If your child ends up in the village, there are no kids here anymore... his focus is still on the family, meaning there are no rules. When faced with these difficulties, he would come here.
JM: Actually, the government's promotional efforts greatly impact your business.
JM: At first, it was very bustling, a large number. But this year, not to mention this year, there's the pandemic.
JM: So, regarding this, the department head needs to manage operations more dynamically.
JM: They were more dynamic around 2007, engaged in cultural news, promoting village tours historically in Nanjing's cultural promotions. This place was developed quite well then. Business was very good those years. When road repairs happened, it became bad. It became extremely difficult. The Niushou Mountain scenic area didn't open.
JM: Probably because the scenic area doesn't divert people to us. Their economy isn't linked with ours. They developed the routes focusing on that area. Back when we started, why was business so good? Every family had customers. The Hohai University was here. People could just cross the hill and come over. Now they've blocked all the roads. The original road we took to Dongshan has been cut off for us. It's not easy to come over now.

****Q:**** Teachers from Hohai University?
****A:**** JM: Teacher Tang also brought them, several tables at a time to my place. The university students would carry the tables outside. Teacher Tang brought the students to my place together. Not enough tables, they carried them down to eat. It was very fun.
It was very lively back then. Now Niushou Mountain scenic area is under unified management, people can't easily come over anymore. They have to go via Dong'an Bridge to get here, it's a detour. Our original route was very convenient, everyone could pass through, people could come over. Now they've blocked our road. Back then, the front gate of Niushou Mountain was right here by us. They built Zheng He's Tomb in front of the former front gate. The front gate became the back gate. It's wrong, the design is wrong. Historically, the front gate was here inside our area. Niushou Mountain has a Buddhist temple. Buddhist temples have always faced west. How can it face east? It's the temple that Niushou Mountain changed.
JM: Originally, it was Niushou Mountain's big temple with 99 bays. Its main gate was right here by us. And Zheng He's Tomb originally also faced west. How can it face east? It was changed during this Niushou Mountain renovation.
JM: Originally, the Hohai University side wasn't the east. Now they built a main gate facing east. The main gate was originally on our side. I feel it's wrong after the change. Previously, I guess Niushou Mountain faced upwards, now it's the east gate.
We locals had no objections initially, but the later change was wrong, the design was wrong. They designed it favoring the development zone side.
Furthermore, this area is completely enclosed now, all tangled up together. Mr. Xie could criticize back and forth, but probably people still can't get in. How do you get in? So, fewer people come over now, it's less prominent.

****Q:**** When did this happen?
****A:**** JM: When this place was being developed. When this place was blocked off, the common people made quite a fuss. Around 2012, the common people protested quite a bit. So around 2011-2012, I don't remember clearly.

****Q:**** From which year did you start the agritainment business?
****A:**** JM: Agritainment... these bases are similar... originally this place was very noisy and bustling.

****Q:**** What's good about this place? Why does it attract tourists to come here?
****A:**** JM: Because of Niushou Mountain, the Niushou Mountain scenic area, the new pagoda built, the sarira (relics). Zheng He's Park is also here. Now, regarding Zheng He's 7 voyages, he didn't actually return himself, his queue (pigtail), his clothes were buried here, a cenotaph. They mainly didn't find him abroad, right, didn't find the person.
JM: The queue from his head, his clothes are buried here, this place. The place where he died is also called Guli, our place is also called Guli. They had to specially build a tomb here. The pagoda on Niushou Mountain and Zheng He's tomb form a straight line. Who knows, maybe it looks good to some, some might say there's a underwater formation... Shanghai is also quite good... dividing the year... tourists come mainly to see the feng shui, and then eat out together. People came to eat. Now they opened an east gate... busy these years, but can't really go astray, right?
JM: Right. If it hadn't been for the pandemic not opening, I'd feel bad. Because of the pandemic, this east gate is also closed.
Now Beijing also has this situation, it's not major. He said there's really no way, if a place has problems, you just have to accept it.

****Q:**** This place is developing rural tourism. The countryside is different from the city, right? There must be differences from the urban area. What do you think is the difference? What is the special characteristic of our place? What is the rural feature?
****A:**** JM: The feature is our agritainment. We are the first of the 'Five Golden Flowers'. Originally people lived here, now it's agritainment. But it's much better than before. Before, it was 'face the yellow earth, back to the sky', every day in the fields. Before, you had to work hard. If it were before, we'd be in the fields working right now. Now, it's like we managed to get... didn't the common people protest? Saying 'this place of yours is wrong, you occupied our farmland, didn't you expropriate it?' Originally, the land below Zheng He's Park over there was all our land, this whole area was our fields. The trees weren't expropriated, didn't give us a single cent. He built the houses. Then the common people protested, because the common people need to live. After this protest, they gave us common people a little money, okay, told us not to protest, and provided us with resettlement. That's how it was. Resettlement means giving you a living allowance for the elderly, that's the idea.
You were originally an agriculture-based lifestyle, now it's agritainment, belonging to the tertiary sector, the service industry. Then it's just like that. Elderly living allowance, like for us who don't have retirement, they give 750, then it increases a bit every year, increases a bit every year. Starting from last year, it might have begun reaching 1040. That's the idea, it increased. You get 750. After resettlement, they give you a living allowance, you get 750.

****Q:**** For the house renovations here, did we pay ourselves or did the government pay?
****A:**** JM: The houses are all our own. The horse-head gables were done by the government. The horse-head gables, the plastering, the exterior work was done by the government. The lime wash for every house was done by the government.
JM: The small bricks were done by the government. They did a bit for each household, first did small blue bricks, that was done by the government.

****Q:**** Originally, when you did agriculture, life was quite hard. Besides farming, did you grow vegetables? Raise livestock?
****A:**** JM: Now, besides agricultural production, we also have a vegetable garden at home. We have a small garden, grow some vegetables. When guests come to eat, we don't need to buy vegetables.
You grow your own at home now? Then homegrown is healthier, tastes better. If there's something to eat, we pick it ourselves. Some things taste good homegrown, some things bought outside taste good. When people come, we are right there selecting them.
JM: Last time, wasn't it when we delivered food, not family members... careful... people came to my house to eat, said it's fresh, fresher than bought from the market.
JM: Why? They say, whatever young people do, whether succeeding at the Forbidden City or being some official, they need to maintain contact with the common people, have awareness of connecting with the masses. I remember, because doing things, no matter what, it's good to unite with the common people. Common people have things to say about state-owned enterprises. Why could Chairman Mao's cadres, cultural propaganda, even though living conditions were poor, they were lively, mentally upbeat, isn't that right?
Now people are too rigid. So for developing tourism, you actually still need to complement each other, attract people's attention, otherwise people won't come.
JM: Look not just at Beijing, look at Chairman Mao. Fighting wars, wherever he went, he would gather a group of young women, go to any village to do propaganda. If you don't promote, the common people in the past wouldn't understand. He did some propaganda, fully mobilized the masses, won some hearts, people felt comfortable, and listened to him, right? If you make people uncomfortable, like the Kuomintang made people uncomfortable, people wouldn't listen.
JM: When people communicate, people feel happy. The poor, starting revolution, anyway had no fields, no land. In the past, the landlord families had fields and land, which common people had any? Work as laborers? Being a laborer, even at year's end, you might get nothing. Those with conscience gave you some grain, those without conscience, you got empty hands, nothing at all.

****Q:**** Because of tourism development, roads were built, more cars. Has this caused any inconvenience to our lives?
****A:**** JM: This place has roads. But back then, no buses came here. There should be a bus stop... which year was it that the bus stop was built? It got a bit better. The transportation is relatively convenient now. Back then, no buses came here. Then buses from the Guli residential area started coming here. Public buses, there's a stop ahead. But no buses come directly from Nanjing. There's a bus station in Nanjing, but people coming to visit need it to be more convenient. Jiujiatang has one, but it doesn't connect to this place.
The transportation here still isn't very convenient.
JM: For elderly people who want to come visit, they must come with family. When they have time off, Saturday, Sunday, off work, then they can come out. Elderly people can't drive, taking the bus isn't convenient, they also find walking tiresome.
