# Supplementary material for: Rurality representation and changes in rural tourism destination
Source: PLoS One. 2026 Apr 21;21(4):e0347226. doi: 10.1371/journal.pone.0347226 (PMC13098982; doi:10.1371/journal.pone.0347226)
Supplement: S1 File — (ZIP) [file pone.0347226.s001.zip › supporting information/世凹村录音及转译文本/jsa9.docx]

Q: Have many people moved away after the demolitions?

A: JM: Some people left, some didn't. The houses aren't returned. Being in this location... the government paid for it, so that's fine. But the government paying means these funds... we pay for it ourselves? (Unclear). JM: It should be determined by the government. All the development is done by the government.

Q: How old are you currently?

A: (Response about education/culture is unclear or missing). I have little education.

Q: How long have you lived locally? So, how old are you? You've lived here since childhood, right?

A: JM: Not here when I was little. I'm from elsewhere, I married into this village.

Q: How long have you lived here?

A: JM: About 20 years.

Q: What is your approximate annual household income?

A: JM: I just have this job. My husband is in decoration. Roughly over 100,000 yuan a year, sometimes there's work, sometimes not. You are probably employed by a company? Here in the community, I'm here dealing with those matters for them. It's like the government... we, the village committee, are also like this. Sanitation.

Q: You've been here about 20 years, right? What do you think are the biggest changes here? Quite significant changes?

A: Right.

Q: In what aspects? For example, food, daily use, the villagers, or your life – any changes?

A: JM: Okay, anyway, speaking of... like housing, things like that. Guli has developed very fast. Ten years ago it wasn't developed like this. The development after those ten years has been very good?

JM: It's because they started agritainment here.

JM: Agritainment was especially good here those years, very good for a while. Later, every family did it, lots of people, and the pandemic had an impact.

Q: After development here, do you think the water or air quality has changed?

A: JM: The air here is still good.

Q: What do you think is the biggest difference between here and the city? We have rural life here, right? It must be different from city life. What is the biggest characteristic of rural life here?

A: You mentioned this area... I think the biggest difference between here and the residential compounds over there is what?

JM: The difference seems... living here isn't as good as the compounds. More mosquitoes, lots of mosquitoes in summer, right? Summer is not good.

Q: What is your ideal vision of rural life?

A: JM: Rural... we were all in the mountains before. You were in the mountains before.

Q: Are there any folk customs or festive activities here? Did they have activities in previous years? Any you know about? Can you give an example?

A: JM: There are performances, yes. They have dance performances. Each community organizes them like competitions.

Q: Has transportation here improved compared to before?

A: JM: Yes, better. Before the roads were just a little wide, now the roads are nice and wide. Before there were just small paths you could walk on. Now it's all flat land.

Q: Has the layout of our town here changed? Was it replanned?

A: The difference is definitely huge. Much nicer looking than before.

JM: When chatting with others here, they said the original farmland has basically been built over with houses. Who knew the government would do it, the government took it.

Q: What do you think is the most attractive aspect of Shi'ao Taoyuan? Is it the food, the environment, or something else?

A: JM: The attraction is... like in spring, the peach blossoms are already blooming. The peach blossoms here bloom... they're beautiful. I missed it. Like in spring, the beginning of spring, it's all flowers.

JM: Look, at this time [of year] there's nothing for you to play with, nothing to see. Seeing people go to agritainment... generally, people can't dislike eating, right?

Q: Over there, I remember there was a peach orchard you could enter?

A: JM: You can go in, no ticket. Is that where the peach blossoms are? There is a peach orchard over there.

It was also later, after Zhongcheng specifically focused on tourism, then changed to... (unclear). Originally it probably wasn't that place either.

JM: Before there wasn't any. The renovations made it all into Shi'ao Taoyuan. It's all peach blossoms now.

Q: Do you think life here is better, or life in the city is better?

A: JM: But the environment here is good. For living, city life is definitely more convenient for city people. The city is convenient. But if you talk about good air, it's better here. The environment is good, the air is good. Wake up early, run, take a walk, the air is good. Now here it's almost the same as the village, right? Better than the city.

Transportation and everything has been repaired, very convenient.

Q: You've been here just over a year? Based on your understanding, what are the relationships between people like here?

A: JM: It's really quite good. Since we came here, the village people have been very nice, all very good, very polite when talking to you. Like they've been doing it [likely agritainment/hospitality] for a long time.

Q: Have you brought your family here for tourism, or to eat here?

A: JM: No. Probably too accustomed to it here. Come too often, so don't want to come.

Q: Have you been to Niushou Mountain?

A: JM: We have many tourist spots here. Some charge, some don't, many are free. More are free.

Q: What was rural life like before?

A: JM: Life before was of course not good. The roads weren't good either. Didn't go out at night, didn't come out to chat at night or anything. It was just those few families living together, coming out to chat, walking to the doorway, chatting. If you didn't go out, there was nothing to play with, right? Look now, every village has something like a small square, things for small children, all that. Before there wasn't any.

Q: What changes have you seen in the village these past few years? What changes in your village recently?

A: JM: The changes in the village... I'm not too clear, I just came. Can't figure it out clearly.

Q: What areas do you think need improvement here?

A: JM: Over there... are there some of the old houses here that have already been demolished? Few were demolished, basically none were demolished, they were all renovated.

Q: You know that events like weddings and funerals are quite important here. Have you noticed any changes in how they are done?

A: JM: It's definitely different. In our time, there were no wedding dresses, no emcees.

JM: Before, neighbors all came to help, the village was lively. Now sometimes they go to restaurants, less trouble. It was livelier before.

JM: Here, quite a few people were relocated, they gathered you all together quite a bit.

JM: Right, relocation. Basically, they would have you move into the communities.

JM: In the town, there's a centralized resettlement place. There's a very large area over there. During relocation, did you see? That house, if it could be demolished there, could it be demolished? They demolish it, level the land there, build residential compounds. We... (unclear about small money). Why isn't this area planned together all along?

Q: Do you go out shopping more frequently now?

A: JM: Yes, go out regularly. Before, wouldn't go out for a week. Now it's all better, go out often.

Q: Is there public bus service here now?

A: JM: Yes, convenient. Usually every half hour. Quite convenient. Right, more buses, available everywhere.

Q: What about buying groceries?

A: JM: For buying groceries, ride a bike to the market, go to the market. Before, also went to the market. Before, in rural areas, near larger communities, there would be people selling groceries. Now it's gone, right? Now it's all planned together, all go to Guli Town to buy, it's been planned that way. All in the community. Over there, definitely where the supermarkets are concentrated. In this regard, look, normally nearby, how do you see... It's different from before. I also can't say I won't discuss it, right? It's fine.

Q: Do you prefer... are you now registered as an urban resident? Or are you from a Hunan village? If you wanted to change to urban registration, wouldn't you want to try living there? Is it... (unclear sentence about Northwest Ma and conditions). The money came back... haven't you been here almost a year?

(Potential incomplete/questionable Q&A sequence here, likely transcription issues).

Q: During the pandemic, do you think many people came? To visit Niushou Mountain? Were there many people using it? Not many people eating. The gate here wasn't open, wasn't watching? Meaning, a year ago, before it was operating, was that gate open or not?

A: JM: Didn't open this year, right. When it was open, were there many people eating here?

If this gate is closed, some people don't come. Those who come can't get in, only people who specifically take vehicles, right?

Q: Has the household garbage here increased?

A: What garbage? Still about the same as before.

JM: Definitely less during the pandemic. If it was like last year, then more. Also more management personnel.

Q: Did you originally speak the local dialect here? Is Mandarin more common now?

A: JM: I don't speak Mandarin. Speaking Mandarin is quite good. I wanted to ask, don't you speak the original local language anymore?

In Guli, you speak Guli dialect. The dialect isn't the same from one place to another.

Is Mandarin more common now?

JM: We don't speak Mandarin.

Q: Are there any temples or similar places here?

A: (Response not provided in text).
