# Supplementary material for: Rurality representation and changes in rural tourism destination
Source: PLoS One. 2026 Apr 21;21(4):e0347226. doi: 10.1371/journal.pone.0347226 (PMC13098982; doi:10.1371/journal.pone.0347226)
Supplement: S1 File — (ZIP) [file pone.0347226.s001.zip › supporting information/世凹村录音及转译文本/ysa-8.docx]

Q: I'm curious, what was it that attracted you to come here?

YK: Leisure, leisure. The air is good here.

Q: Is this your first time here?

YK: I come often.

Q: You've been coming since early on. Compared to before, what changes have you noticed here?

YK: The changes aren't very significant.

YK: I've been coming since it was first developed.

Q: What do you think, since this is a rural tourism spot, what elements should it include to attract tourists?

YK: This place has good greenery, fewer people. Yes, and the food is alright.

Q: What do you think is the biggest characteristic of this village?

YK: This village is quite leisurely, not taxing.

Q: How has the transportation been in the last couple of years?

YK: Transportation is very convenient.

Q: Is the landscape maintenance and construction a form of protection, or is it a better form of protection for it?

YK: It can't really be considered damage. I'd say it's better now than before. Before, before this village was renovated, it was definitely shabby and run-down. The earliest I came was around 2012. When I came it was already quite good, already like this. Now it's even cleaner.

Q: What did you think of the village's architectural style initially?

YK: The architectural style is quite good, quite distinctive. Yes, it has it. Those black and white ancient-style dwellings, that style, right?

Q: Have you experienced any rural activities here, like picking or farming?

YK: That kind? No.

Q: If they were available, would you be interested?

YK: Yes, definitely would take kids. Would take kids, definitely. No choice (laughs). We come just to sit, rest, drink tea, eat. When the kids are older, we'll bring them.

Q: Zheng He's Tomb is also part of this area, there are some historical and cultural sites there. Are you interested in those?

YK: Not too clear about the past history, can't be bothered to learn about those things. We just come to have fun.

Q: What is your ideal, yearned-for countryside like?

YK: Just like this. Exactly like this. The countryside is different. It's about having fewer people. For people our age, that's more comfortable.

YK: Many landscapes... artificial ones are actually worse than the original. This place doesn't have any local specialties. Maybe some dishes... haven't really seen any vegetable plots here either. I haven't seen many households with fields.

Q: They don't have fields now.

YK: Yes, this area used to be fields. Originally it was farmland, now it's gone.

Q: There might still be some now, maybe some families still have small private plots, grow some vegetables. There might still be a little bit of that.

YK: There is a chicken farm further back though. I think that's quite good, this planning. This place, the Hui-style architecture is quite alright. The countryside is just like this, just like this. They had to change the houses into Hui-style architecture first, that looks quite nice. It's the same as the nearby Shitang Renjia. It seems basically all the developments around Nanjing, in Jiangning area, are like this. All are this style.

YK: Before tourism development, according to these pictures, it was so unattractive. When I first came, it had just started developing, so it felt incredibly poor. But now, after the development, it feels very beautiful.
