# Supplementary material for: Rurality representation and changes in rural tourism destination
Source: PLoS One. 2026 Apr 21;21(4):e0347226. doi: 10.1371/journal.pone.0347226 (PMC13098982; doi:10.1371/journal.pone.0347226)
Supplement: S1 File — (ZIP) [file pone.0347226.s001.zip › supporting information/世凹村录音及转译文本/ysa11.docx]

Q: So, they say that's why the agritourism businesses here basically have very few customers now.

YK: Yes, I searched for a long time before finding this place.

Q: You can look at these two images first. This one is from around 2000, and this one is from last year.

YK: I've been here before. This is roughly the situation.

Q: And your age is approximately?

Q: What is your occupation?

YK: Work for a unit.

Q: Work for a public institution?

YK: Yes.

Q: You are a university graduate?

Q: Are you a local resident? Or...

YK: From Nanjing city, right?

Q: Your annual income should be...

YK: Over 100,000.

Q: Anyway, for the questions we ask, just answer based on your own feelings. Looking at these two images, what changes in the countryside over the years do you feel are quite significant? For example, interpersonal relationships, or modes of production, or original lifestyle habits, or things they used before.

YK: When I came last year, it was pretty much the same.

Q: Based on the countryside you knew as a child, or its overall change until now, what aspects do you feel have changed noticeably?

YK: It's definitely getting cleaner. Cleaner, right? More hygienic, right?

Q: Do you think there have been major changes in interpersonal relationships compared to the rural relationships you knew as a child and those in the countryside now?

YK: Now people all want to earn money. The economy is like that too. Now the local cuisine restaurants are becoming more commercialized. The dishes aren't as well-made as before.

Q: Do you think the primary mode of production in the countryside you remember was originally agriculture, and now it should be mainly agritourism?

YK: Yes. Now it's all quite commercialized.

Q: In your impression, were rural people originally more simple or honest? And now, how do you feel about them? Still acceptable?

YK: Definitely, it's all commercialized now.

Q: Still commercialized? So you mainly think commercialization is quite severe? Commercialization, you understand, includes a lot; they pursue profit.

Q: And perhaps before they started agritourism, the wealth gap here was smaller, life conflicts were fewer, and villagers helped each other more. But now, because they are all running agritourism businesses, each is busy with their own work, so mutual help or communication between them might be less.

Q: Did you come here recently, or...?

YK: I've been here before.

Q: You should have some impression of what it was like before?

YK: No. I came after it was renovated. Came after the renovation was done. 2018? 2019? Roughly like that.

Q: What do you think of the transportation here?

YK: The transportation is okay. I came by car.

Q: What about the water quality here now compared to before? Any major changes?

YK: The water quality is okay. We all focus on environmental protection.

Q: Do you think the surrounding countryside now suffers from issues like too many tourist cars causing noise pollution or similar?

YK: There definitely must be a bit, right?

Q: When you come here and communicate with them, their language, the communication, do you think it's okay? Understanding, etc.?

Q: Since the beginning, from what we understand, the agritourism development was initially arranged uniformly by the government. Do you think it's better for the government to arrange it uniformly, or is it more reasonable to hand it over to company-type enterprises? Just based on your own understanding.

YK: Government-led... they definitely still need to introduce social capital. Social capital comes in. Where would the government have the money?

Q: What do you think of the overall village appearance and environment here?

YK: Clean and hygienic, it's okay. You can see now, with fewer people, it's much cleaner. If there were many people, it might not be.

Q: What about the houses here? For example, maybe there were some old buildings originally that have now disappeared. Are you deeply impressed by that?

YK: No. When I came, I hadn't been here before. No.

Q: What do you think of the houses built here now?

YK: They're okay?

Q: Do they match the feeling of the countryside you have in mind?

YK: More or less.

Q: When you come here for tourism, is it because of the distance factor, or do you have a deeper understanding of this place? What is the biggest reason attracting you here, to Shi'ao Taoyuan or Niushou Mountain?

YK: Niushou Mountain... Niushou Mountain is developed quite well. The scenic area inside is quite well done.

Q: When you first came, what left a deep impression on you here?

YK: Niushou Mountain... Regarding meals, eating is quite convenient, right?

Q: What aspects here do you think might need further improvement or enhancement?
