# Supplementary material for: Rurality representation and changes in rural tourism destination
Source: PLoS One. 2026 Apr 21;21(4):e0347226. doi: 10.1371/journal.pone.0347226 (PMC13098982; doi:10.1371/journal.pone.0347226)
Supplement: S1 File — (ZIP) [file pone.0347226.s001.zip › supporting information/世凹村录音及转译文本/ysa4.docx]

Q: Are you a local?

YK: I'm a local.

(Note: Seems there is initial confusion about who is the local and who is the tourist between the speakers)

Q: We thought you were a tourist too.

YK: I am a tourist. I'm not (a local). That person is the local. I'm the tourist. Not sure what happened.

Q: What is your occupation?

YK: IT.

Q: And your education level is approximately? Master's degree?

Q: Roughly how old are you?

YK: What are you investigating?

Q: We are investigating your understanding of the countryside, a concept called 'rural character'. We are about rural tourism, right? Yes, but the concept is 'rural character', and its changes. The countryside needs to have changes.

Q: Have you lived in the countryside before?

YK: No.

Q: So, what do you think the countryside should be like?

YK: I think the 'Socialist New Countryside' here is quite good. The countryside in Southern Jiangsu is all similar, pretty much the same.

Q: What differences do you see between the countryside here and other rural areas you've visited?

YK: I've been to villages in the North and West, for business trips. Those places aren't as good. The Jiangsu-Zhejiang area is better. Yes.

Q: What are the main characteristics of the Jiangsu-Zhejiang countryside compared to other regions?

YK: The villagers' thinking is more active. They develop things like agritourism. There's less of that in the North. Here, they integrate concepts like mountains, rivers, and culture, blending in cultural elements. The development in the North is either based on 'red tourism' or focuses on local cuisine. These might temporarily attract the older generation, but young people aren't very willing to go there. Things like 'small bridges, flowing water' – everyone is willing to come here for that.

Q: What is the biggest feature of this village, in your opinion?

YK: I think the biggest feature is its proximity to Niushou Mountain. Usually, the number of people coming to play might not be too high, but there's a Pujue Temple and a public cemetery at Niushou Mountain. During the Qingming Festival (Tomb-Sweeping Day), the number of people coming to sweep tombs is especially large. So, for that month, this place should be a hotspot. Yes, generally, people from Nanjing eat a meal after tomb-sweeping.

Q: Do you often come to this village to visit?

YK: Not really. Because I'm in Jiangning area... Anyway, our family Xiao Wang wouldn't come here to eat.

(Note: 'Xiao Wang' likely refers to a family member or friend)

Q: So you've been here a few times for work, right?

Q: When was the first time you came?

YK: Many years ago, many years ago. It wasn't developed so well back then.

Q: It was developed in 2012. Did you come before 2012?

YK: Roughly. At that time, there were only one or two (businesses) developed.

Q: This village has been developing tourism for so long and is relatively mature now. What changes have you noticed during this tourism development process?

YK: I haven't felt any major changes in recent years. Because it was just agritourism back then, and now it seems it's still just agritourism.

Q: So it's been very stable.

YK: Yes. It has always been like this.

Q: What about the roads and infrastructure?

YK: Usually, coming here to visit, like today, is certainly fine. But once it's the peak tomb-sweeping period, parking becomes a problem, right? Then people who come based on its reputation might leave disappointed because they can't get a meal.

Q: Too many people.

YK: The passenger flow is too large. Other aspects are okay. And its location is on the opposite side of Niushou Mountain. Unless you have a private car and know about this place, people will come to eat after visiting Niushou Mountain. Generally, few people seem to know about it.

YK: I also found this place once by searching nearby after tomb-sweeping. If you are genuinely visiting Niushou Mountain, few people know about this place.

Q: Before, when you came, were there still many farmlands? And now they are basically gone?

YK: Roughly the same, pretty similar. That road has been under construction continuously.

Q: Do you have any contact with the villagers here?

YK: The boss/proprietor. I've eaten here and finished my meal. There's a bit more over there... You can't have people eat at every single one. Anyway, I've eaten at two or three places here. Actually, the villagers I contact are just the bosses.

Q: What do you think of the villagers here?

YK: Quite good. The villagers are quite simple and unpretentious. The general folk customs here are still very good.

Q: What elements of the countryside here do you like the most?

YK: Leaving aside the taste of the food... For example, if there was a small pavilion to eat in, or like now, sitting here, swinging on a swing, something like that would be better. We mainly come to the countryside for a leisure experience, for this kind of slow-paced life. This is an agritourism spot.

Q: Do you like the pace of life here?

YK: It's okay. But you are here to spend money. You still have to go back to work. I like it temporarily. Coming, for example, on weekends to have a meal or something, I think it's quite nice.

Q: Do you think the water or air quality has changed? Over the years you've been coming?

YK: Changed? It has been managed, should be okay. No pollution problems, comparatively.

Q: Previously, when you came, did you feel the overall village appearance was improving?

YK: It's quite mature now. Back then when I came, there were only two or three businesses, and many facilities weren't developed yet. I think it's still developing slowly, overall developing for the better.

Q: If we don't talk specifically about this village, what do you think an ideal, beautiful countryside should be like? Can you simply describe what elements it includes, or what scenes it might have?

YK: These (businesses) are small businesses run by the farmers themselves. Normally, it should be agritourism in the front, and farmland or similar in the back. Then things like China Telecom, mobile networks – information reaching the village. And each (household?) should have its own small house. Also, education should be relatively universal. And everyone should have mobile phones, TVs, internet access – these should all be fine.
