# Supplementary material for: Rurality representation and changes in rural tourism destination
Source: PLoS One. 2026 Apr 21;21(4):e0347226. doi: 10.1371/journal.pone.0347226 (PMC13098982; doi:10.1371/journal.pone.0347226)
Supplement: S1 File — (ZIP) [file pone.0347226.s001.zip › supporting information/世凹村录音及转译文本/ysa6.docx]

Q: Have you lived in the countryside before?

YK: Yes.

Q: What was rural life like in your opinion?

YK: At our age, if you're talking about how it was before, that's hard to describe... it's a long story.

Q: Are you familiar with this village?

YK: I just arrived two minutes ago. We saw your place and came over.

Q: So, first time in this village?

YK: Yes.

Q: What do you think is the biggest difference between rural life and city life?

YK: Rural life is more open, more open. People can be more casual. City life, right? You step out the door and it's the main street, right?

Q: Which kind of life do you prefer?

YK: At our age, of course we prefer rural life.

Q: Do you think rural life is suitable for you to relax in, or to live in?

YK: To live in.

Q: You just prefer and are accustomed to the environment.

Q: Have you been to any of the many 'Beautiful Countryside' scenic spots near Nanjing for tourism?

YK: Rarely. Because we are migrant workers.

Q: Have you been to any of these rural spots? There are many now developing 'Beautiful Countryside'. Do you think different 'Beautiful Countryside' spots have different characteristics?

YK: We've been to very few, so we don't know about the differences. It's like this all over the country now, everywhere is doing it. Our hometown in Huaian is also doing it. Some are probably quite similar, many are.

YK: Of course, some places aren't like this place. This place was originally... personally, I think the topography with mountains and water is better, so it's developed quite well. In our place, it's a waste of effort and money – it was originally just a plain. Our place is just flat land. They piled up so much earth to make it like small hills. And how much good farmland, land that grew grain, was destroyed... At first, when it was just developed and opened, it looked quite nice. Many people went to see it. But you can't look at it every day; life isn't about going to see it every day. You need to have money to live, and only then go see it. If you can't even eat your fill, would you still go see that stuff, right? Like the one near our place, I went once. It's been over 600 days now, almost two years, I only went that one time, just for a day, and only for a few hours. For me, having seen it once is enough.

Q: I see. What did you think of that place?

YK: What was it like? I didn't have the thought to go again. I went once and never went again. For example, in our Jiangsu Jinhu, we have the Lotus Pond. Have you heard of that place? I went there. To see the lotuses, you have to go in summer, they only bloom around May-June. If you go then, it's unbearably hot. In winter, or on mild days, you could go, but there are no flowers.

YK: So it's different depending on the season.

Q: What impact do you think this kind of rural development has had on the local residents in your hometown?

YK: It actually added a... basically, it's mostly acceptable for kids. For working people, not much significance. Yes, because you still focus mainly on work, right? Students focus on school, right?

YK: You can't... For nearby residents, it's more like having a... if I have nothing to do, I can run over and take a look, right? You can't, if it's dozens of kilometers away, just drive there today for no reason. You need some economic means to go, right? You can't just... look, drive there in the morning to relax, then drive back, then go eat breakfast... we don't have that kind of reality. This is just the view of someone our age on this matter.

Q: So, for example, if this village develops tourism, occupies farmland, and changes it into buildings, then the villagers can't farm anymore. What do the villagers get? For example, people here run agritourism businesses; some make money, some don't. What are the villagers in the place you mentioned doing now? How do they make a living?

YK: Now, very few farmers farm the land. Even if you mainly farm, a family, even with more people, 10 mu of land. If you stay home farming all spring, one person normally staying home for a year, farming 10 mu, after deducting farming costs, and only if you have a good harvest, you might make over 10,000 RMB, nearly 20,000 RMB. Do you think it's worth having one person stay home for a year to farm? So, no one farms anymore. If someone occupies it, they occupy it, right? No one really disputes it. They give a one-time buyout. For example, if my family has 5 mu of land, maybe the compensation is over 20,000 per mu... over 100,000 RMB? I get the money in hand, right? I can use that 100,000+ RMB elsewhere. I'm not counting on the land to get rich, right? But when a real famine year comes, and there's no food to eat, then they will know. But this is long-term. Maybe you'll never encounter it in your lifetime, or maybe it's right around the corner. As people say now, who knows whether accident or tomorrow comes first? Right.

Q: So, you think now that their land is occupied, they might participate in the development...

YK: It's different from what I described. It's different here, after all it's in Nanjing, with lots of people coming and going. Our place is just a small county town, over 200,000 people. Developing this stuff... I'm not criticizing here, I'm criticizing our hometown's situation.

Q: What impact do you think this kind of tourism development brings to the countryside?

YK: Of course, if you don't develop and utilize it, the countryside remains countryside forever, and no one comes...

Q: You think when developing tourism, the village's overall appearance actually improves, right?

YK: The environment and such also improve. And income is the same. Income... it doesn't mean you lose out because of this change. Because with tourism, they might do some sideline businesses and earn more money.

Q: Do you think this village has encountered any negative impacts during tourism development?

YK: Because I just arrived at this place, not this place... you can talk about the village you mentioned earlier.

Q: The bad aspects...

YK: Because we don't go to that place often either. We go occasionally, don't know the locals, don't have any interaction, so I don't really know. But just from my own perspective, at my age, with my viewpoint, is investing so much able to be recouped? But one can only say that this place has such a scenic spot.

Q: Yes, that's how it is. But how many people go?

YK: It seems besides the initial opening days when people went, it's still relatively deserted now, not many people. Occasionally when there's a holiday, like a major festival, people nearby might go in to play for a while.

Q: Having lived in the countryside for so long, do you have an ideal 'Beautiful Countryside' in your heart? What is it like? A countryside you particularly yearn to live in?

YK: For people our age, this idea is: being able to not worry about food or clothing, receiving a pension every month, that's enough. Nothing more. No illness, that's enough.

Q: Regarding the environment... because when we get old and retire, we also need leisure, right?

YK: Leisure... now like we live in the county town, we also have parks, etc. It's a large concentrated area of people. In places with concentrated populations, doing this is necessary. In places with no people, I think it's unnecessary, right?

Q: So you think your ideal rural...

YK: Like large county towns, they build parks, squares, etc., to give people leisure. You say the countryside, places in the middle of nowhere, it's like this.

Q: Maybe in the future, you yourself would still want to live in a place with a slightly better living environment, like a county town?

YK: Yes. But county towns also have their...

Q: But some people, for example, in a well-developed, environmentally good countryside like this, would you be willing to live here?

YK: Of course he wouldn't be willing? But you can't come here.
