# Supplementary material for: Rurality representation and changes in rural tourism destination
Source: PLoS One. 2026 Apr 21;21(4):e0347226. doi: 10.1371/journal.pone.0347226 (PMC13098982; doi:10.1371/journal.pone.0347226)
Supplement: S1 File — (ZIP) [file pone.0347226.s001.zip › supporting information/世凹村录音及转译文本/ysa7.docx]

Q: Is this your first time coming to this countryside area? Have you been here before?

A: No, I haven't. Today is the first time? Yes.

Q: What are your impressions of the countryside here?

A: YK: The environment is nice, definitely good. The tourism development here is quite alright.

Q: Where is your hometown?

A: YK: Huaian, Jiangsu.

Q: What do you think rural tourism here should be developed into to be better? What would you like to see, what experiences would you like to have?

A: YK: Experience the original appearance, I suppose. That means environmental improvement – the environment should be good, like the original state, the rural character, including some villages, architecture.

Q: Any expectations regarding their local cuisine?

A: YK: Regarding food and beverage , you can't make it like a shopping mall outside, right? If it's like a shopping mall, we might as well go shopping, right?

Q: What about productive agricultural activities, like picking or farming experiences? Are you interested in those?

A: YK: That's also good, also good. It's an experience of rural life. Yes.

Q: For this village here, do you think there are any areas for improvement if it wants to develop further?

A: YK: I wouldn't know, because I just arrived, I'm not very familiar with it. Yes.

Q: Do you live in a city or a county town? What do you think—in the past, and now seeing how developed and modernized this rural area is — do you prefer the slower pace of life from before, or the current modernized version with convenient transport and daily life? Which one do you think might be better?

A: YK: Personally, I think those who permanently live in the city yearn for the mountains; those who live in the mountains yearn for the city. Everyone thinks differently, right? No matter how good the environment at your doorstep is, you might still think the outside environment is better. Going out to travel... right. People have different opinions. Right.

Q: Overall, regarding this issue... What do you think, for a village, are folk customs, festival celebrations, including things like dragon and lion dances, important?

A: YK: That's also good. But can it attract and gather people? Nowadays, many businesses can't manage that. Right.

A: YK: In our place, because there are many recreational spots now, it's not easy to attract a crowd.

Q: Yes, probably also faces issues like young people being unwilling to participate, skills being lost...

A: YK: Yes, and also... I remember when I was little, we still had dragon and lion dances in my hometown.

A: YK: Unless you set a specific date, decide on a specific festival for it... that might work.

Q: In the past countryside, this was also a great collective activity. Now, in this countryside, I understand that elderly people generally play cards, dance, etc., and young people seem to be mostly absent.

Q: What do you think now, how about here... What do you think about the current situation where every household has air conditioners, color TVs, cars... this lifestyle, does it indicate that rural life is getting closer and closer to urban life?

A: YK: Yes, you feel that now they are basically converging, everyone aspires to modernization.

Q: (YP?): In this context, how can villagers better develop the countryside? Should it follow our country's 'Beautiful Countryside' construction, meaning the countryside should develop in this direction in the future, or should it hold onto its most primitive aspects?

A: YK: It's not about being the most rural... if there are many scenic spots, it becomes indifferent . After all, a city is only so big. If the city is large, and the surrounding areas have a good environment, then the influence spreads. If it's a small city, people from far away won't be willing to come. No matter how well you develop it, you can't become like Huangshan Mountain, such a famous landscape, that's not realistic, right? Some places rely on their mountains or waters. Here, you can only rely on radiation from the Nanjing urban area to attract people here for recreation, right? For example, opening some agritourism businesses, you need to attract people. If no one comes, it's also a kind of torment, right? It increases the financial costs, right?

A: YK: Yes, but people in this countryside area are the same. You can't expect everyone, as some say, to get rich from agritourism. At the very least, it should support the family. But some people will definitely go outside to work other jobs to earn money. They might not necessarily rely on this, right? Right. You can't expect that just because this place develops tourism, every family can make a living from it. You probably don't think that's very realistic either, right? Some people will always... unless you run a large restaurant and work as a server, right? You can't expect everyone to be a boss; that's also unrealistic, right?

Q: (YP?): Looking now, actually, this rural area feels like it has been well-planned, the environment is quite clear, that's its characteristic. But before, I understand there was a lot of farming here. Where we are now used to be farmland. Do you think this is a kind of regret?

A: YK: But listen to me, that's correct. It has such a good advantage, being next to the mountain, such good resources. Why isn't mountain development allowed now? This thing is a non-renewable resource. Once it's excavated, it's gone, right? Some places, after excavation, become bare mountains. It's true, anywhere can have flat land, you can build buildings there too. But there's no place that can 'create' a mountain. They say the environment is best, it's non-renewable for the future. If you deteriorate the environment, it might be possible to restore it, but at what cost? After the environment is damaged and you try to restore it, the health of the people living around might already be ruined. It might take one or two generations to restore it to green mountains and clear waters.
