# Supplementary material for: Rurality representation and changes in rural tourism destination
Source: PLoS One. 2026 Apr 21;21(4):e0347226. doi: 10.1371/journal.pone.0347226 (PMC13098982; doi:10.1371/journal.pone.0347226)
Supplement: S1 File — (ZIP) [file pone.0347226.s001.zip › supporting information/大山村漆桥村录音及转译文本/DS-JM 01.docx]

Basic Information:

(1) ID: DS-01 (e.g., SA/DS/QQ-00)

(2) Gender: Female Age: 35 Occupation: Owner of a farmhouse inn (agritainment business)

(3) Role: √ Resident □ Tourist

(4) Education Level: □ Junior high school and below √ Senior high school (including technical secondary school) □ College and Bachelor's degree □ Master's degree and above

(5) Years of residence in this locality:_35 Participation in tourism: Yes

(6) Annual household income: □ ≤10,000 □ 10,001~50,000 √ 50,001~100,000 □ >100,000

(7) Sources of household income (multiple choices): □ Farming √ Tourism-related service industry □ Others (e.g., migrant work, salaried employment)

(8) Tourist's Occupation (if applicable): □ Enterprise employee □ Professional (doctor, lawyer, teacher, etc.) □ Self-employed / Freelancer □ Student

Q: How many years have you lived here?

A: I've been here since birth.

Q: What changes do you think have occurred since the establishment of the Slow City here?

A: I'm still not satisfied.

But there is this situation: before the Slow City was established, people would work outside the village, or the slightly older ones would work as migrant laborers elsewhere. Now, after establishing the Slow City, basically many people have returned. Also, the appearance has changed; the roads have changed too. If the Slow City hadn't been developed, they wouldn't have been able to build the village into what it is now. There wouldn't be so many scenic spots, and the roads wouldn't be like this either.

Q: Do you feel any sense of 'slowness' or have any 'slow' experiences?

A: It might be because I've always been here. Since I'm originally from Gaochun, I don't feel it much.

Q: So, you mean the 'Slow City' is just a label, and there isn't really a feeling or experience of 'slowness'?

A: Compared to the outside, it is indeed different. But because I've always been here, I don't really feel it because there's no comparison. (The life here itself is leisurely, that's why a Slow City could be established here.)

Q: What cultural experiences do you think the Slow City provides for tourists during their visit?

A: Originally, there were some agricultural activities and such here. But now, for various reasons – probably some were leased from others, and due to various factors – they've been changed. This experience has become worse.

> But things like Gaochun ceramics, for example, weren't promoted like this before. Now, basically, many outsiders know about it, and it has even been used in state banquets. That's quite good.

Q: What about changes in less visible aspects? For instance, the slow pace of life, quality of life, living atmosphere – could you talk about that?

A: There are still such changes. Because originally, before the Slow City was developed, people were basically not at home. Sometimes, you wouldn't see your neighbors more than a few times all year. But now, basically everyone is at home, and visiting each other has increased.

Q: Do you think the development here is different from other rural tourism destinations? Have you been to other rural tourism places?

A: There are differences too. Because many places are actually developed better than here. Some places have more culture and inherent scale. For instance, in some places, what each household does doesn't repeat; you do this, I do that, forming a complete, integrated concept. Here, there's too much repetition of offerings. All the farmhouse inns are just farmhouse inns; it's very homogeneous.

> Then, if you have something else, like on one street, you serve breakfast, I serve lunch, and another family sells snacks, linking it all together. You serve tea, I make wine, that would be even better. Each household has its own specialty. This way, visitors can go to your place and then also go to others'. Otherwise, if they come here for a meal, they eat at your place and won't go to another place to eat again; it still creates a kind of conflict [competition].

Q: Where do you think the deviation lies between the ideal concept of slow tourism and the reality here? That is, when we heard this area was going to become a Slow City, what kind of picture did you have in mind, and what is the actual outcome after its establishment – what's the gap between the two?

A: Actually, from the start, everyone had a rather vague concept of what a 'Slow City' was. And now, persistently, it's just rural tourism. Apart from knowing it's called a 'Slow City', you can't really tell the difference from other places; it's just a name.

Q: What elements do you think best represent the countryside? Things like landscapes, forests, fields, lakes, a leisurely pace of life, rural simplicity – which one do you think it is?

A: It should have its own agricultural products.

Q: What do you think *should have been* the elements representing the countryside in the past?

A: I think it's agricultural products, because this is something only the countryside has; it's impossible in the city.

As for landscapes like mountains and water, you can find them anywhere, and they can even be imitated.

Also, people. I think people are the most important.

Q: What is your ideal vision of the countryside?

A: It's close. I think our current state is already quite close to that ideal state. If it could be better, I think it should address the issue of the 'benefit chain' we mentioned before. That way, it could alleviate a lot of the neighbor disputes brought by tourism.

Q: What impact do you think the massive influx of transportation, information, capital, and tourism into the countryside brings?

A: It's a shift in income models.

> Also, developing tourism is better for the environment. If it weren't developed, look, people wouldn't handle garbage like this. Now they've started waste sorting, so the overall appearance is better. Without this, trash bins definitely wouldn't be collected by dedicated personnel every day like now. But without tourism, there wouldn't be so much household waste generated either. The amount of garbage from meals now is indeed high, but if handled properly, it's not a problem. Our family often takes it out and deals with it ourselves, and it's fine. After all, it's ours.

Q: Do you think it has any impact on behavior?

A: Mandarin. Before, basically, the slightly older people all spoke dialect. Now they all speak a bit of broken Mandarin.

Q: What about spiritual aspects, like identity, festive activities, etc.?

A: Because we've always had a 'Long Street Feast'. Basically, every year around the end of the year, in the lunar December, we have a feast along one street, setting up hundreds of tables. Similar to the long street feasts in places like Yunnan. Also, sometimes around the Winter Solstice, there's a free lunch for the elderly.

Q: These are all impacts brought about, right? Including what you mentioned at the beginning, that people are willing to come back and stay in their hometowns, and relationships between neighbors have become more harmonious.

A: And it alleviates urban employment pressure.

Q: Could you please help fill out this diagram? What were the elements that best represented the rural characteristics of Dashan Village in 2005? Correspondingly, what do you think are the elements that best represent its rural characteristics now? About 5 or 6 items each.

Q: 15 years ago, it seems the roads inside weren't like this, there were no proper roads, different from now.
