# Supplementary material for: Rurality representation and changes in rural tourism destination
Source: PLoS One. 2026 Apr 21;21(4):e0347226. doi: 10.1371/journal.pone.0347226 (PMC13098982; doi:10.1371/journal.pone.0347226)
Supplement: S1 File — (ZIP) [file pone.0347226.s001.zip › supporting information/大山村漆桥村录音及转译文本/DS-JM 10.docx]

Q: I'd like to ask, have you lived here since childhood?

A: Yes.

Q: After the development of slow tourism here, what kind of experiences, particularly slow cultural experiences, do you think are provided for tourists? Cultural experiences specific to this area.

A: I'm not really clear about that.

Q: For example, are there any landmarks or symbols indicating our Slow City? Or some folk activities, cultural events?

A: I really don't know. It seems pretty much the same as before. Do you know about any distinctive local festivals, like the Golden Flower Festival or the Long Street Feast? What do you think of them?

A: I honestly don't go to those. My wife goes, I don't. According to my wife, she says the Long Street Feast is nice, the Golden Flower Festival is also good. She tells me about it when she comes back, but I don't go. I stay home.

Q: Before, our area was entirely rural farmland. Now there's no land left, nothing. The land was taken back for regional improvement, now there's nothing left.

Q: The original residential land was taken by the government?

A: It was contracted out to others.

Then they pay 800 RMB per mu per year. Just 800 RMB per mu. How much did your family get per year? My family gets over 7,000 RMB, for over 9 mu of land.

Q: How do you feel about the compensation?

A: I feel it's actually not bad. If you farm it yourself, you still need fertilizer, pesticides, it's exhausting. Now, I can find work or go out, it's relatively better. Before, having land meant I couldn't leave.

Q: Do you feel that after tourism development here, the village's appearance has changed? Become cleaner or neater?

A: Changed a bit. It's indeed still not great, the village appearance isn't good, but it's much better than before. Anyway, since I came here, I feel the environment is quite good, the environment is okay. We moved here from the village below? Anyway, looking at it now, it seems much better than before.

Q: Do you think the development of tourism has affected paddy fields and vegetable gardens?

A: Paddy fields, vegetable gardens... if you're willing to work hard, they still exist.

Q: First, are there still vegetable plots after tourism development? What's the difference compared to the past?

A: There's a difference. In the past, everyone farmed the fields, grew vegetable gardens. Now some people do, some don't. Now only the elderly farm them.

Q: Is there poultry raising?

A: Probably not. Not much. Some households still raise chickens for their own consumption. In the past, everyone raised them, it was the same. Now it's much less. Many people don't raise them anymore, only some households still do.

Q: How is the water quality in the ponds around here?

A: Not as good as before. But we have tap water anyway, the water quality itself doesn't affect us much. Having tap water makes it cannot be designated as.

Q: Has the layout and overall appearance of the village changed significantly compared to the past?

A: Changed a lot. Some villages have improved greatly, very well.

Q: I see many ancestral halls have been renovated?

A: Some places have been renovated, some haven't. Certain villages might have been renovated.

Q: So, has the ancestral hall in our Dashan Village been renovated?

A: Yes, it has been renovated.

Q: After the development here, has your pace of life become slower or faster?

A: Actually, it has become faster.

Q: Okay. Do you feel any changes in kin relations or neighborly relations after the development?

A: No real change there. It's still pretty much the same as the past situation.

Q: How has tourism development affected your... sense of identity? Do you still consider yourself a citizen, or do you think of yourself as a city person now? Or...

A: Actually, I'm still a farmer. That can't be changed.
