# Supplementary material for: Rurality representation and changes in rural tourism destination
Source: PLoS One. 2026 Apr 21;21(4):e0347226. doi: 10.1371/journal.pone.0347226 (PMC13098982; doi:10.1371/journal.pone.0347226)
Supplement: S1 File — (ZIP) [file pone.0347226.s001.zip › supporting information/大山村漆桥村录音及转译文本/DS-JM 11YG.docx]

Q: I'll ask some questions based on your own feelings. What major changes do you think have occurred in your village over the years?

JM: Major changes in GDP, and also in lifestyle. Before, our village mainly relied on farming the local land, working the fields day and night. Now, it's primarily farmhouse inns; the whole street is full of them, all engaged in tourism farmhouse inns and accommodation.

JM: Before, people were mostly farmers; now they've become business owners. Everyone runs small businesses now. Some operate tourist accommodation or tourist catering. The sources of income have changed, primarily shifting to the tertiary industry.

Q: In your memory, what was the village like when you were a child? Can you recall?

JM: Back then, our village was probably the poorest one in the surrounding area, very poor. Other villages had cement roads paved, but our village had none at all. They were all mud roads. On rainy days, it was unwalkable. If you wanted to go from our village to another, people wouldn't come through our village because we were so poor.

JM: When I was a child, the population was average, I only had a few playmates. The population is a bit larger now.

JM: Compared to when I was little... were there many young people back then? Compared to now...

JM: At that time, for young people, if you didn't get into university after finishing school, you would just go directly to Nanjing as a migrant worker. Basically, they moved to towns and cities, either to Nanjing city proper or to Gaochun county town.

Q: What is the countryside like now? Can you describe a typical daily scene in our village now? What do you think it's like?

JM: Tourists come in to eat at our places.

Q: Compared to your childhood?

JM: The change is huge. When I was little, my family was very poor, we hesitated to eat anything nice. Now, it's all possible.

Q: What elements do you think best represent this rural area? For example, houses, cattle, sheep, means of transportation, or landscapes like mountains, water, forests, fields, lakes.

JM: It's still culture. The most representative, symbolic thing I feel is culture.

Q: Do you think what was most representative in the past has changed compared to now? What were they respectively?

JM: In the past, mainly, I felt every village was pretty much the same. Actually, no village had unique culture back then. Now everyone has developed, they bring in some culture to attract others.

JM: You think culture can represent the present now, but in the past, they definitely didn't excavate this stuff.

Q: What about things like transportation, e.g., newly built highways or high-speed rail, and information, like the internet age now, and also capital—meaning, since you're doing tourism here now, there must be some inflow of external capital too, right? And the developed tourism—what impacts has it brought to your area?

JM: Tourism brings publicity (fame/recognition), the publicity has increased. Also, various... let's say environmental development. Before, there weren't two ponds in front of my house, there was nothing. In front of the house, there was only one pond. Then, to make it look nicer for you all, they dug another one, and a pagoda—this was dug.

Q: Roughly when was it dug?

JM: When I was 17 or 18, about 10 years ago, they started slowly improving the village's appearance. These things definitely brought you impacts, right? Huge. For example, digging a pond, besides making you feel it's prettier, maybe in other aspects, for daily life, it's more pleasant to look at. Before, it was just stagnant water.

JM: Underneath my small pavilion was a stinky ditch. Now it's filled in. Before, underneath was all stinky ditch, couldn't look at it.

JM: The change is really huge. Coming here now, I think the environment is very good. Walking by the river, you can feel cool and refreshed. For example, now because transportation is developed, information is developed, it definitely brings more tourism, meaning tourist flow and such things.

Q: What impact do you think these things have on the material elements of your countryside? Let me give examples, like the layout of your village/town, your natural landscape/water, your water quality, your family's vegetable garden, including some ancient bridges, ancient features... What impact has this large-scale flow brought to these rural elements?

JM: Before, you had poultry running all over the roads, now that's gone. It's more standardized now. With more people, everyone became more regulated. The local authorities definitely had to implement some standardized policies. Actually, it's not fully handled yet; some households might still let them out, but when visitors come, they don't see it anymore. Regarding layout, roads were built, asphalt was paved. At least you don't have the situation like before where after rain, people couldn't walk, right? Also, many trees were planted along the roads, but some original areas were also destroyed. Originally, in front of my house, there was a very large patch of rapeseed flowers. I have photos from childhood, a huge patch connecting to the mountain. After they dug this pond, now only a small patch remains.

Q: So there was also some destruction, right? It's changing. I think it's mainly for the better, but individually there might be a little damage, right? Besides the impact on these objective things, there must be many impacts on everyone's behavior, or your pace of life, these regular patterns.

JM: Behavior has changed a lot. The internet era has a big influence. For example, mobile phones have a huge impact. Before, people might just focus on work, farming. Now everyone holds a phone to play, the impact is quite big. Also, many people make Douyin (TikTok) videos, everyone films, right?

JM: Lots of people film. My mom often asks me to film with her, it's too embarrassing for me.

Q: I see, now even the parent generation is starting to play with them.

JM: Yes, I can't stand it anymore.

Q: Do they visit each other's homes now? In the past, when you were young, you probably visited frequently.

JM: Visiting still happens quite a bit, especially among older people. After all, just playing on the phone gets boring. Sometimes on cooler summer nights, they gather together to chat.

Q: Your transportation before was probably bicycles or something, now you should all have...

JM: Basically every family has a car now. No need to worry about that. Including places to buy things, locations for shopping.

JM: I think before, when buying things, you mainly didn't go out to buy. There were very few small shops anyway. Selling some candy, melon seeds... When I was little, buying those small melon seeds for a few cents, or candy, made me extremely happy.

Q: How do you mainly buy things now?

JM: When I go home, there are tons of packages, 7 or 8 a day. There have also started to be pickup points for various express deliveries, but the main ones are still in the towns. My parents go to work in the city company every day, so they bring them back.

Q: Are there any changes in your diet?

JM: Yes. Don't think that farm food (farmhouse cuisine) just developed naturally like this; actually not. When tourism started booming, our farm food offerings increased. Naturally, to cater to tourism needs, it gradually became more varied, and then what we ourselves eat also changed somewhat, right? It's better.

Q: Do you think there have been changes in people's spiritual aspects? For example, their religious beliefs, moral habits, cultural confidence.

JM: This... not really. No significant changes.

Q: Look, from your perspective, with all the tourism facilities in place here, it must also make you feel more recognition of this place, more confident. And your rural atmosphere, including some village rules and agreements—compared to your childhood, for instance, you have an ancestral hall, some family genealogies, maybe the family has some rules, things passed down—has that stuff changed? Do you think it was stronger in childhood, or is it stronger now?

JM: It's stronger now. When I was little, nobody paid attention to it. But now, my father is the one revising the family genealogy. People are starting to have this awareness, to protect the culture.

Q: What do you like most about your countryside now?

JM: Still the air... the environment is very good.

Q: What areas do you think can be improved now?

JM: If we're talking about our village now, it definitely focuses on tourism. In the future, I think we still need to develop some more tourism projects. There isn't much to play here; you can only come for a meal. Besides this kind of leisure, unless you make people spend a lot of money to come, your economy might be further boosted.
