# Supplementary material for: Rurality representation and changes in rural tourism destination
Source: PLoS One. 2026 Apr 21;21(4):e0347226. doi: 10.1371/journal.pone.0347226 (PMC13098982; doi:10.1371/journal.pone.0347226)
Supplement: S1 File — (ZIP) [file pone.0347226.s001.zip › supporting information/大山村漆桥村录音及转译文本/DS-JM 12 YG.docx]

Q: From your childhood until now, what aspects do you think have seen the most significant changes in the countryside over the years?

JM: Mainly in water sources, mountains, fields, forests, and also buildings. The changes in these aspects are relatively large.

Q: In your memory, what were these aspects like in the countryside when you were a child?

JM: Relatively simple and crude. Back then, there was nothing. For example, this pond wasn't here before; it seems like it didn't exist. Probably there were fewer structures and facilities in the past. Now they are there; things have become more numerous and richer.

Q: What things do you think best represent the countryside?

JM: Wenfeng Pagoda, then the tea plantation land, the reservoir – the largest areas. These can all represent the countryside.

Q: Do you think the things that represented the countryside when you were little have changed compared to what represents it now?

JM: Yes. Before, there were also more fields, and people raised chickens and ducks, etc. They still do now, but it's less common.

Q: Do you think these things can reflect the real life of the countryside, right? What is your ideal countryside like?

JM: The way it is now is quite ideal. The environment is pretty good.

Q: What impacts do you think things like the improved transportation (e.g., high-speed rail), the information brought by the internet, external funds from tourism development, and the influx of outside tourists have brought to our countryside?

JM: It doesn't seem to have much impact. Before, there probably weren't this many tourists coming. Now there are more tourists because transportation has become more convenient, and information is more widespread – people learned about this place; many didn't know before, but now they do, and travel is easier. So these people came and brought some changes to the countryside.

JM: Sometimes, I observe the environment... issues like littering. Before, there might not have been; with more people, such uncivilized behavior occurs.

Q: Roughly when did this impact start?

JM: It seems it started around two or three years ago, during busier periods.

Q: Is the impact significant? Does it interfere with your life?

JM: Yes, it does.

Q: For example, with the large number of tourists coming now, does it impact the rural elements you mentioned earlier? Elements like your paddy fields, some natural scenery, your poultry, and the natural environment? Do you think these people have brought any impact on these things?

JM: Probably no major impact.

Q: Does it affect your daily habits?

JM: No, it doesn't.

Q: It doesn't affect your lifestyle either; you carry on as before, right? And what about impacts on spiritual aspects? For instance, your cultural confidence, sense of identity? Do you have that feeling? Like, before our village might have been isolated and poor, but now with some tourism development making it more prosperous, do you feel more confident?

JM: Yes, there is that.

Q: Basically, that's it. Do you think there are any areas in the current rural tourism that need improvement or correction?

JM: Regarding the ponds and such... how is the environment...

Q: Improvements?

JM: Make it better, improve the environmental greening.
