# Supplementary material for: Rurality representation and changes in rural tourism destination
Source: PLoS One. 2026 Apr 21;21(4):e0347226. doi: 10.1371/journal.pone.0347226 (PMC13098982; doi:10.1371/journal.pone.0347226)
Supplement: S1 File — (ZIP) [file pone.0347226.s001.zip › supporting information/大山村漆桥村录音及转译文本/DS-JM 13.docx]

Q: Has this village undergone any changes in the past two years?

A: JM: No changes. It hasn't changed for several years.

Q: When did tourism start here?

A: JM: Tourism has been going on for about 10 years now.

Q: What changes has tourism brought since it started?

A: JM: It's been better for the agritourism businesses at the front of the village. People don't have to go out for migrant work anymore. Before, the women worked outside, now they basically run agritourism. The households along the main road are all doing it.

Q: Is this an 'Agritourism Street' created by the government? Why is it popular?

A: JM: It seems people just got used to coming this way, and more people came. Each household now has regular customers. This street formed a market. The scenery is nice too, with lights in the evening and planned, nice-looking agritourism places.

Q: Was the landscape worse before tourism?

A: JM: Definitely. Over a decade ago, the roads were dirt and gravel, not asphalt like now. The government develops and maintains the roads.

Q: So the government does the roads, but what about the agritourism businesses?

A: JM: The agritourism businesses are all our own effort. The houses were built by ourselves.

Q: Does the government support tourism activities with subsidies?

A: JM: No, no subsidies for agritourism. It's all our own effort.

Q: Does the government help with overall renovations?

A: JM: The government did the Ma Tou San (horse-head gables?), and the exterior wall painting. The rest is all done by ourselves. As tourists increased, more agritourism businesses were built.

Q: How was business, especially during the pandemic?

A: JM: Business was good in peak seasons previously. It dropped a bit during the serious pandemic period.

Q: Has the physical environment improved due to tourism? What about farmland and water?

A: JM: Our farmland was mostly expropriated by the government, only small vegetable plots remain. We don't farm anymore. The pondsides were improved by the government. Water quality was better before, less polluted. Now there's some pollution from people washing things, but the government has installed sewage pipes underground, which is good. They also put utility lines underground, so it looks much better.

Q: Has the village layout expanded due to tourism?

A: JM: Yes, there has been expansion. A smaller village, Xiao Rui Jia, was merged into our Da Shan Village. New houses were built there, and those people now belong to our village.

Q: Is everyone in this village surnamed Rui?

A: JM: Basically, yes. Women who marry in have different surnames. Some new agritourism operators who moved here are from Xiao Rui Jia.

Q: Are there any typical rural cultural features?

A: JM: The Rui Family Ancestral Hall. There's also an old house, which has been repaired a bit. There's an ancient tree next to it. The old house collapsed and was rebuilt with bricks.

Q: I see a pagoda on the hill, Wenfeng Pagoda?

A: JM: There was originally a small pagoda, and it was rebuilt taller. There's also an opera stage next to it, which has always been there.

Q: Is the stage used by the village?

A: JM: Yes, for opera performances during the fair on March 18th of the lunar calendar.

Q: Is it held every year?

A: JM: Yes, every year on March 18th. It existed even before tourism.

Q: What kind of opera?

A: JM: Yue Opera and Huangmei Opera.

Q: Sung by locals?

A: JM: Funded by locals, but performers are invited from outside. It's said the opera is for the Bodhisattva to listen to, and there's a crowd watching below. It's like a fair, very lively. People invite relatives to watch. It lasts for three days. This is a local custom that has continued. It wasn't held this year due to the pandemic restrictions.

Q: Is it for a Bodhisattva's birthday?

A: JM: Yes, but actually, it's not exactly the birthday. It's performed for the Bodhisattva, but the date is fixed on March 18th here. It's our local tradition.

Q: What about Da Shan Temple?

A: JM: The temple was newly repaired. Some parts are new, some are old. The lower part with the Eighteen Arhats is new. The temple near the large Bodhisattva statue was newly repaired, and we agritourism operators donated money for it. The whole village pooled funds.

Q: Have neighbor relations changed because of running agritourism?

A: JM: It's still good. Our relations are very good, similar to before. We help each other. If we lack some ingredient when guests come, we borrow from neighbors, and they are willing. We borrow from each other. If food is insufficient, we share. Neighborly relations are fine. You mind your business, I mind mine.

Q: Is basically the whole village doing agritourism?

A: JM: Not like in Lü Jia village, where they are all business-minded and solicit customers. Here, we don't solicit. Over at Peach Blossom Fan Square, you might have old ladies calling you to eat, but here no one does. Our village has regulations; we have an Agritourism Association. Customers choose where they want to eat. Our houses are our own, we don't aggressively solicit. If you want to eat here, fine; if not, that's okay. The customs and atmosphere are different in other villages; they have stronger competition and less regulation.

Q: Have living habits changed?

A: JM: Living habits have changed. Before, we couldn't go out easily. We always had to have someone at home to open the door, as regular customers might come, or people call around 11 AM saying they are coming for lunch. It's hard to mind the business, tied to the home. Before, when working jobs, we had weekends off. Now we can rarely go out, especially on weekends.

Q: This area is promoted as a 'Slow City'. Does it offer cultural experiences for tourists? Like Slow City标识, folk handicrafts? Has anything been revived?

A: JM: Yes. There's tea-making. Tourists can experience making tea themselves, pulling bamboo shoots, digging sweet potatoes. All are available. Digging sweet potatoes is around March or a bit later in spring. If tourists want to participate, if the activity requires a fee, we pay it; if not, then we don't. There are these experiential activities. We also take tourists picking fruit.

Q: Picking what?

A: JM: Currently, grapes, dragon fruit. We take guests picking. If the farm charges a fee, we pay it. We can take guests because they eat at our place, it's like being friends.

Q: Are there other rural experience activities, like the Long Street Banquet?

A: JM: The Long Street Banquet started only after tourism developed. It's held on this street, from the village entrance... it's very fun. There's Horse-Lantern Dancing, opera performances. Even if it snows, people eat at the agritourism places happily. The food is kept warm with alcohol stoves. There's pig slaughtering, goat butchering, putting up couplets, with specialized calligraphers helping write them for free. It's great.

Q: Is the government helping organize this? Or sponsoring?

A: JM: The government also sponsors. We agritourism operators basically just cover costs for the banquet. I know it's 500 RMB per table, but we don't get the full 500, the government takes a part. We cooperate for the promotion of Da Shan Village. That day is very happy. There were even foreigners dancing on the stage last time! I still have the videos on my social media.

Q: Are there any traditional handicrafts?

A: JM: Yes, fans, woven baskets, local products, old people sewing tiger-head shoes for children... these are handicrafts.

Q: Are they sold now?

A: JM: Not currently for sale, but people still make them. It's not the season now.

Q: If it's a 'Slow City', how is the 'slowness' embodied for tourists?

A: JM: The slow lifestyle... for instance, there isn't even a supermarket here, just small shops. Everything is slower, more backward compared to others. No supermarket, only small shops.

Q: Your transportation seems convenient?

A: JM: Transportation is slow too. The public bus stops running after 2:30 PM. There's no other option. But we have our own cars.

Q: What makes this place different from other villages doing rural tourism?

A: JM: Our area is better because there are mountains, so development happened here. Because the resources are better, the development is more apparent.

Q: What should the cultural experience of 'Slow Culture' be like? Any areas for improvement?
