# Supplementary material for: Rurality representation and changes in rural tourism destination
Source: PLoS One. 2026 Apr 21;21(4):e0347226. doi: 10.1371/journal.pone.0347226 (PMC13098982; doi:10.1371/journal.pone.0347226)
Supplement: S1 File — (ZIP) [file pone.0347226.s001.zip › supporting information/大山村漆桥村录音及转译文本/DS-JM 14.docx]

Q: I'd like to ask you, as you've lived here for 17 years, about the changes in this village. From when you arrived until now, could you talk about them?

JM: When I first came here, this place was really tough, very poor. To get home, I either had to detour five or ten kilometers if riding a bike, or walk along the ridge paths. In summer, the grass was this high. The time it took to walk back then was about the same as cycling or driving now. It didn't necessarily take much longer, but there were no proper roads home; you had to walk on the ridge paths. The road conditions were bad back then, the transportation was poor.

Q: What about other aspects of the village back then, like the fields, ponds, and buildings?

JM: What can I say about the people here? If they had a little money, they would build a house. They would save every penny to build a house. The houses back then were okay, decent enough. When I first came, their family raised seven or eight pigs. They had a lot of land. Their main income was from farming. The elderly, they could only farm, grow some wheat, rapeseed, rice—some for themselves to eat, and sell the surplus. Then they raised pigs and some poultry, did farming. They relied on these as their main source of income. Or maybe they had a fishpond for raising fish, things like that. Mainly agriculture and aquaculture. Then, once the kids were a few years old, I went out to work.

Q: What about leisure activities back then?

JM: We were quite busy with work. On holidays, we'd just buy daily necessities or some snacks, take the kids out often, go shopping, things like that.

Q: What do you think the village is like now?

JM: The conditions are much better now. Before, having a basic mobile phone was good enough, but now smartphones are very common. Everyone's always on their phones, and online shopping, right?

Q: What about daily habits?

JM: For example, there were very few cars here before, but now they're very common. Basically, every household has one. That's partly because the roads are better, transportation is more convenient, and with the improvement in living standards, yes, basically every family has a car now. Also, people's sources of income have changed. For the older generation, they did odd jobs. People here are quite hardworking—tea picking in spring, harvesting bamboo shoots, some still do aquaculture like crab farming.

Q: And now?

JM: Now, many are involved in tourism. Like this row of houses in the front, they're doing tourism-related things, and some work as migrant laborers elsewhere. Only the front row is really into tourism; further back, they still rely on migrant work and agricultural production. Rural people have less farmland now, just enough for their own consumption.

Q: What elements do you think best represent the village, both in the past and now?

JM: In the past, it was definitely agriculture. Agriculture represented the countryside. Now... how to put it? Mornings here, the traffic flow isn't as heavy as in the city. Now I feel there's not much difference between the countryside and the city. I mean, it's quite convenient here now, transportation and everything is convenient. The houses, because every household is independent, has its own small courtyard, right?

Q: So you think now the difference between the countryside and the city isn't big. What is your ideal countryside like?

JM: The ideal one... how to say? It should be quite... Now life is better, but I actually prefer a bit more privacy, a more private space.

Q: So you hope your ideal countryside, perhaps on the current foundation, would add more privacy. Understood.

Q: In your opinion, how have changes in this era—the internet, information, transportation, tourism, and the influx of visitors—impacted the village? Can you be specific?

JM: Maybe back then, they would just find locals here to act as translators if needed, couldn't really handle outsiders properly... I wasn't specifically introduced to it, but anyway... Yes, people's attitudes are different too. Their way of thinking has probably changed.

Q: Specifically, which aspects have changed?

JM: People's concepts might be influenced by outsiders. For example, some elderly people, who before might just kept things for their own use, now they think about selling anything they can spare. They've developed a business awareness.

So, before, society was more about self-sufficiency, and now people think about making a profit. This change is quite noticeable.

Q: Roughly when did this influence start?

JM: Also about three or four years... the last three or four years.

Q: Okay. So, regarding the changes we just discussed, like transportation or tourism, have they had any impact on the physical elements of our village?

JM: For example, our farmland, ponds, buildings—have they been affected?

Q: For instance, the ponds?

JM: To be honest, the water quality now isn't as good as before, right? The water quality has deteriorated.

Q: What about the flows, like tourist flow, information flow, transportation? Have they influenced village behavior—your daily routines, habits? Think about it.

JM: Yes. Before, after work, we'd just come home and rest, watch some TV. But now, we play with our phones, scroll through our phones.

Q: Do you stay up later?

JM: It depends on family needs. For my son, because of night self-study sessions, he comes back quite late, so we definitely have to go pick him up.

Q: So, entertainment methods have changed, from watching TV to scrolling through phones, probably things like Douyin (TikTok), right? Have these things had any impact on the village's spirit, like farmers' sense of identity, cultural confidence? There should be some impact, perhaps making people more confident, right?
