# Supplementary material for: Rurality representation and changes in rural tourism destination
Source: PLoS One. 2026 Apr 21;21(4):e0347226. doi: 10.1371/journal.pone.0347226 (PMC13098982; doi:10.1371/journal.pone.0347226)
Supplement: S1 File — (ZIP) [file pone.0347226.s001.zip › supporting information/大山村漆桥村录音及转译文本/DS-JM 15.docx]

Q: You can speak freely about the changes in our Dashan Village over the years, from over a decade ago until now. You can talk about any and all aspects, like the natural environment, including everyone's living habits, food, housing, transportation, tourism, shopping, and entertainment.

JM: What can I say? To be honest, since developing agritourism, our living conditions, quality of life, and all aspects have basically doubled. The quality of life, naturally, and also people's... quality, everyone's general quality has improved. And how to put it? Although we're doing business, the relationships here are still quite harmonious. People say 'harmony brings wealth'. It's not like having business leads to arguments between families. We neighbors get along very well. For example, if you're missing a certain vegetable and another family has plenty, well, you can borrow from them. Neighborhood relations are quite good—harmony brings wealth.

JM: And, how should I say? I feel that before, when we were farming the land, we really couldn't earn much money. Now, after opening the agritourism businesses, it feels like life is actually easier. People are more relaxed, incomes are higher, and it feels like everything is improving quite quickly. We're relatively satisfied with the current situation. It should be okay.

Q: In the past, everyone in the village farmed.

JM: Did migrant work, then. Earned one or two thousand yuan a month. Life back then was, to be honest, quite plain.

JM: Back then, how to say... we didn't have these kinds of... Now, although our mindset is also quite good, more peaceful, right? I actually feel our mindset back then was even 'better' than it is now. Better in what way? I think it was like we didn't have much ambition, we were content with the situation at the time. Nobody thought much about it, everyone could earn a little money. I never thought about buying a house or a car, didn't even consider it at all. Now, with the economy developing like this, people naturally have aspirations, people definitely want to progress, right? So, we think about buying houses, buying cars, and then all aspects—clothing, food, everything—have become more abundant. Material needs are more plentiful. So children live better too. So, certainly, the quality of life has improved in all aspects. When the economy is better, life and thinking naturally...

Q: Over 10 years ago, everyone lived like this in the countryside.

JM: Just farmed, it was relatively leisurely. Some people, as they say, got up in the morning, tended their small vegetable plots, went to check the fields. Transplanted rice when it was time, harvested rice when it was time. We just did those tasks. Didn't have many entertainment options, didn't have much ambition. That's just how it was.

Q: Were there more rice paddies and such back then?

JM: Yes, all that was vegetable fields, plenty of them. Now, every household has some... every household grows rice, vegetables, rapeseed, small vegetable plots. Every family had them because we relied on that for living back then. And for younger people, there weren't as many factories as now. Now, you can find work anywhere; anyone can support themselves. It wasn't like that before. Factories were few, very few. We were limited, just some garment factories nearby, not many. Just worked in those garment factories. Later, development slowly started, after this tourist village construction began, after it started over at Wangyu Island, more and more was built. Later, they came to our village to develop this agritourism model. We came back to start the agritourism business.

Q: Roughly how many years ago did it start?

JM: It should be 10 years. Has it been 10 years? No microphone? You were 11 years old then, right? Agritourism started when I was in third or fourth grade? (Editor's note: This seems like a conversational aside/correction regarding the speaker's child's age).

JM: At 11 years old... should be 10 years. Yes, roughly 10 years. My son is 19 this year... 9-10 years.

Q: What do you think best represented the village in the past? What was the most representative thing about Dashan Village in the past?

JM: The lifestyle of farming the land, the way of life and production—that could represent it. Yes, because it was all rural back then, that was the strongest representation. Migrant work... you could say it was mainly farming. There wasn't much idle time, maybe a few months with nothing to do. Or, the women farmed at home, and the men worked. If there was nothing particularly important, they might occasionally go out for migrant work. It wasn't highly representative; mostly it was farming back then.

Q: And what represents it now?

JM: Now, how to say... now it's things like agritourism, focusing on tourism, and staying at home to run businesses... Actually, regarding the current rural construction, to be honest, agritourism is a relatively representative feature.

Q: The development efforts are also quite significant. What is your ideal countryside like?

JM: My ideal... I actually... how to say? As long as my mindset is very good, as long as our agritourism... the surrounding environment could be somewhat improved. And then, for ourselves... it's not that I want to make the business absolutely top-tier, because there are too many agritourism businesses now. You have to be realistic, face reality. I just imagine... as a woman at home, if I could achieve the most ideal scenario, it would be earning over 100,000 yuan a year, enough to support the family, not worry, not rely on my husband's money, earn my own money to spend myself, and be able to take care of the child. That would be ideal.

Q: What aspects do you mean by improving the surrounding construction?

JM: For example, building more attractions that make visitors feel it's worth staying, more recreational projects. Something that feels refreshing, makes people feel they can play for several days. Because, you see, our Dashan agritourism has been around for nearly 10 years. From when I started my agritusiness until now, I feel like it's almost unchanged. Saying 'unchanged' might be too harsh.

JM: Overall, it feels like there's not much novelty. When visitors come, it's still the same, right? Not much change. The visitors themselves feel it too. They might think... because the competition is too strong now. The agritourism businesses built later, they learn from your experience, and they do better and better, right? So you paved the way, but for our agritourism, to be honest, we started just like that, step by step. Actually, we didn't have others' experience to borrow; we started relatively early here. We figured things out ourselves, no prior experience. Then others came, looked, set things up—they had examples to learn from. More and more people are designing, they're bound to do better than you, right?

Q: The latecomers surpass the old-timers, as they say?

JM: Some places are done particularly well, right? Really. For our place, to be honest, it feels a bit tiresome. Really, it's like that. It's just that if someone happens to come play, they feel the air is good, this original state exists, but they might feel it's a bit messy, still needs more organization.

Q: After the tourism development here, including the improved transportation, what impact has it had on our village? Both good and bad.

JM: To be honest, how should I put it? The advantages outweigh the disadvantages. How to say? Maybe if too many people come, there could be some air pollution, probably. There is pollution, definitely, because with more cars and people, certainly. But overall, it's still beneficial. Actually, it has brought... the more it develops... Not much, not much negative impact. On the contrary, it has increased, enhanced our people's income. The impact isn't major, but there is a little pollution, probably a little. It can't be all good and no bad, right?

Q: After people started coming, has there been any impact on our village's elements, like trees, grass, the river, or buildings?

JM: These, not much impact. But sometimes, how to say... ultimately, to be honest, people are different. Their personal quality varies. Some people from out of town come to travel, some elderly folks, they see the vegetables we grow ourselves, they can't help but take a little. Probably many people have mentioned this, I've heard them say it. They talk about this messing around. Actually, not everyone is like that. If everyone did it, that would be terrible.

Q: Has it affected everyone's life? Your lifestyle habits?

(Note: The transcript shows the interviewer asking this question, likely rhetorically or summarizing, then immediately moving to the next point about 'spirit').

JM: (No direct answer recorded to the previous lifestyle habit question before the topic shifts).

Q: What about the spiritual aspect? Has communication increased because of the influx of outsiders? You mentioned quality improved earlier; spiritually, has it become richer, or is there a stronger sense of identity with the village?

JM: It should be okay. How to say? When city people come here to visit, it definitely broadens our rural people's knowledge and perspective, expands our horizons.

JM: (Interviewer continues): And overall now, do you feel prouder to be from Dashan Village compared to before?

JM: Well, when we go out, people say we are from the 'Slow City', certainly there's a bit of... seems like our village performs better than some others. Yes, a bit happy. It has brought some sense of pride.
