# Supplementary material for: Rurality representation and changes in rural tourism destination
Source: PLoS One. 2026 Apr 21;21(4):e0347226. doi: 10.1371/journal.pone.0347226 (PMC13098982; doi:10.1371/journal.pone.0347226)
Supplement: S1 File — (ZIP) [file pone.0347226.s001.zip › supporting information/大山村漆桥村录音及转译文本/DS-JM 16.docx]

Q: I'd like to ask you next, as someone born and raised here, about the changes in our village over these years. Having lived here for twenty or thirty years, what particularly significant changes do you think have occurred in the village?

JM: The changes are huge. The mountains and water are better, the people are better, work is better, spirit is better, everything is more convenient.

Q: The roads are better, spirits are better, right? In your memory, what was the village like when you were a child, or twenty or thirty years ago?

JM: Back then, you couldn't get out. There were no roads, only small paths. They were generally all dirt roads. When it rained, you couldn't go anywhere. To get to the town, 5 to 10 kilometers away, it took three days to get there.

Q: You couldn't get out back then, the roads were impassable.

JM: When we were kids going to school, did we have a pair of cotton shoes, a pair of cloth shoes? We arrived at school soaked, and didn't even have rubber boots.

Q: Life was tougher then.

JM: We had it very hard when we were young, really. There was no... the village had almost nothing for sale. Going to the countryside, just one pair of cloth shoes, arriving at school drenched. On rainy days, just one pair of small rubber boots... that's how we managed. Rubber boots were even hard to buy back then. I also... in the 1950s, when your mother and them were born, your parents already knew. Times were incredibly hard then, everyone was like that. There wasn't much freedom then, just after liberation.

Q: What do you think best represents our village now?

JM: Now the countryside is wealthy, the roads have all improved.

Q: Things that best represent our village now, for example, the mountains, water, forests, fields, lakes, or the pace of life, or perhaps simplicity... what do you think?

JM: Everything is fine now, it's all gotten better.

JM: Every household relies on air conditioning. Before, summers were unbearably hot, we slept outside overnight. Now every family has air conditioning. Nobody uses those old ways anymore. Speaking of 30 years ago, 30 years ago, the changes in this place have been incredible. Now every family has air conditioning, the local character is completely different, it's just amazing.

Q: But when you mention our village, what do you first introduce to people?

JM: Everyone comes to see the scenery, the landscape.

JM: Currently, the most representative thing is still the landscape scenery.

Q: In the past, what would you introduce about the village?

JM: In the past? Look, the mountains were bare, there wasn't much scenery. They were destroyed back then, several plots of land were ruined, temples were destroyed too. Now it's recovered, it's all been restored to how it was twenty or thirty years ago.

Q: In the past, when people talked about the countryside, they probably mentioned everyone building together...

JM: Mention the countryside back then and everyone shook their heads, "Aiyo, it's hard!" They all shook their heads.

Q: Everyone farmed back then, right?

JM: Rice paddies and such... I even built roads... In the past, they also mentioned rural life was hard, the way of life, right?

JM: Collective labor, no source of income.

Q: In the past, talking about the old countryside made people think of poverty and hardship. Now, look, transportation roads have been built, information in the internet age is developed, and now we're developing tourism. There must be some outside investment coming in too.

JM: Outside investment, outside tourists come to our place. We travel outside, within the country. We go abroad.

Q: Yes, they come here, we go there. What impacts do you think the changes in transportation and the overall development of tourism have brought to our countryside?

JM: When people flow, wealth comes, they build bridges and roads. People come to our place, taxis bring them, we go out to other places, they bring things back. When they come here, they shop, buy local specialties, which to some extent stimulates the economy, attracts people to stay, because we've set up homestays. Last year over there, eating, drinking, shopping – a one-stop service.

Q: Has this development had any impact on the physical aspects of our village?

JM: You mean, on our landscape, farmland, or things like roads, sanitation? Not much impact.

JM: It's better now. Our village has sanitation workers. Basically, there isn't really much impact, right?

Q: No impact? Has it affected everyone's life?

JM: No problem.

Q: And regarding the spiritual aspect, has developing tourism made everyone more confident, for instance?

JM: For the better ones, they are quite happy. Earning money makes them happy, and then gradually they become more confident, right?

Q: Good. That's mainly all.
