# Supplementary material for: Rurality representation and changes in rural tourism destination
Source: PLoS One. 2026 Apr 21;21(4):e0347226. doi: 10.1371/journal.pone.0347226 (PMC13098982; doi:10.1371/journal.pone.0347226)
Supplement: S1 File — (ZIP) [file pone.0347226.s001.zip › supporting information/大山村漆桥村录音及转译文本/DS-JM 17.docx]

Q: I'd like to ask you about the changes in the village over the years. Your family has been here for twenty or thirty years, right? You've lived here for over 20 years. Could you talk about the changes in the village during this time? Any aspect is fine.

JM: In the past, I remember when I first came, the village roads here were no good. The changes now are huge. For 10 years, since Lulu started development and built the roads, the roads have been good. Good.

Q: Apart from the roads, what about the houses? Have there been changes?

JM: The houses... this type of house... the houses allowed agritourism to develop further. So people built houses taller, more of them. Then agritourism developed, and every household started getting into the tourism business.

Q: Good. What do you think are the things that best represent the countryside? What are the specific changes between past and present?

JM: Greenery and that sort of thing are all good. The greenery... one thing I'm a bit embarrassed, I do know, anyway I know about these projects, I know a bit about them.

Q: Things that best represented the village in the past – what might come to mind? If you had to describe the past to someone, what things would you most likely mention?

JM: If you ask me to explain specifically, I can't really say it. You can probably imagine the scene, but I can't really express it, right?

Q: Yes. Past life was about casual chats, there was no way... right.

What would you most tell others about the village now? If you went out and chatted with others about Dashan Village.

JM: I'd say the development in our village is good. Things like the roads, the Wenfeng Pagoda and such, the surrounding environment... just these things. Basically, that's it. There's nothing else for me to say, really.

JM: If our place hadn't been developed, it would be a really backward area. Before development, it was very backward. After development, it changed; everyone started becoming wealthier. It was no good before.

Q: Right. And now that our roads are built, meaning transportation has improved, and tourists can come in, what kinds of changes has this brought to our village? What impacts do you think it has had on our village?

JM: In business, in wealth. In wealth, it has increased everyone's income.

Q: The impact in this aspect is quite significant. And what about on our local landscape, this area? Has there been any impact on the environment, any damage, or no damage? Any improvement? For instance, has the area become cleaner or more orderly?

JM: There has been improvement, no damage. It's quite good, right?

JM: Personal awareness has also improved a lot. In the past, things left outside our doors would get stolen. Now, nobody steals them. Everyone's general quality is quite high. The quality of our local people here has gradually improved.

Q: The impact brought is very good. Has it affected everyone's living habits? They must be much better now. For example, before people might have slept early and risen early. Now, has the pace of life become faster because of the city people arriving, or any other changes?

JM: How should I answer... Has your pace of life changed? If yes, you can say specifically what kind. If not, then no. Based on the actual situation, talk about it. Say, after this tourism, for example, after the roads were built and tourists came in, did it affect everyone's living habits?

JM: No, we still maintain our original living habits.

Q: Mainly, the aspect of general quality, you feel has improved? Because of interacting with outsiders, or because you need to run agritourism businesses, right?

JM: That is to say, personal quality has improved.

Q: What about our cultural confidence here? Has becoming wealthier from tourism made everyone more confident? More identified with their status? Proud to be farmers?

JM: Yes.

Q: Good. Now I'd like to know your age.

JM: I'm 55.

Q: You don't look it! And your occupation?

JM: Run an agritourism business at home.

Q: Not at home?... Running your family's... Should be considered self-employed. Okay.
