# Supplementary material for: Rurality representation and changes in rural tourism destination
Source: PLoS One. 2026 Apr 21;21(4):e0347226. doi: 10.1371/journal.pone.0347226 (PMC13098982; doi:10.1371/journal.pone.0347226)
Supplement: S1 File — (ZIP) [file pone.0347226.s001.zip › supporting information/大山村漆桥村录音及转译文本/DS-JM 18 .docx]

Q: You just mentioned the biggest changes in the village over so many years.

JM: Let me see... not many changes. It's prettier than before, just prettier. The plants, greenery, flowers, and the houses have been fixed up. The houses were renovated. Everyone's house was renovated, they all added sunrooms or something in the front. The horse-head gables were also part of the later renovations. Yes, the houses were renovated to be more beautiful, and they have those Hui-style characteristics now – horse-head gables, Hui-style architecture. Also, the road changes are especially big.

Q: What was the village like in your memory, in the past?

JM: It wasn't this pretty before. Not much change really, I see the changes aren't extremely huge. Probably it wasn't this aesthetically pleasing before, and the roads were worse.

JM: Before, people everywhere raised chickens and ducks, they defecated on the roads and such. The sanitation was poorer before. Now it's more orderly, more sanitary, cleaner, and tidier.

JM: Also, in the past, people left things outside... people still leave things outside now, look at both sides here.

JM: No, since I've been here I haven't seen it... You might still see some people put things out, we can look around here later.

JM: It stinks! When it rains, it still happens. They've talked about it many times, but then you have to mention it and it hurts harmony, right? The cadres don't say much either.

Q: So there are still some free-range animals, but much fewer than before.

JM: They don't consider others. Look, if I were developing, would I put it here? You should pen them in, just fence them in, wouldn't that be cleaner?

Q: If you were traveling now and met new people, and they asked about your Dashan Village, what do you think you would most tell them about?

JM: I'll tell you, I'm never proud of our Dashan Village. Never proud of Dashan Village. Everyone is selfish, completely selfish. They are all quite selfish.

Q: If someone asked about your hometown, what would you say to them?

JM: Nobody really asks me that much anyway. I don't have much to say. It has changed a bit, but not as well done as other places.

Q: Now, with the roads built, transportation improved, and tourists coming in, what impacts do you think this has brought?

JM: A bit more income. More income, in terms of the economy. A bit more from agritourism.

Q: People's business methods have probably changed. Some who didn't do this before are now doing it, earned a bit of money, the economic situation is a bit better.

JM: Elderly people sell some small vegetables, some dried vegetables – those local agricultural products, specialties. If they are promoted, then it's a bit better here.

Q: What about the impact on the rural scenery, fields, ponds, and the water quality? Do you feel any effects?

JM: Now, I told you, the water quality isn't very good either. The water quality has worsened.

Q: Regarding the division of labor in the fields, or people's opinions... it's okay, speak freely. We just want your personal feeling; everyone feels differently, we want to see different people's perspectives.

JM: Some people like to boast, I don't. I prefer more down-to-earth, practical. Seek truth from facts.

Q: After the tourism development here, has it had any impact on everyone's living habits?

JM: Of course it has an impact. Look, people are doing business now, right? If they see your business is good, they aren't happy; see his business is good, they aren't happy either. Between each other... people's hearts aren't as united as before, not as simple and honest as before. There's probably some competition between them.

Q: And what about daily routines, like what time people sleep or get up?

JM: That's just personal preference, it's not really affected. That belongs to the individual. Some people, whether they can walk outside at night before sleeping... Look, I finish my work, take a shower, then I watch TV.

Q: Personal habits. After our tourism development started, has it had any impact on everyone's spirit? Like you said just now, not being proud of Dashan Village – has it gotten slightly better now? Do you feel it's gradually getting better and better, more identified with Dashan Village?

JM: Didn't I tell you? Okay, you can't say there's no improvement at all, right?

JM: The roads, everywhere is cleaner, right? You can't say there's no change, right?

Q: Do you feel more confident and proud as a villager of Dashan Village?

JM: No. The peaceful atmosphere of the countryside has actually worsened somewhat. Because of the competition between each other. Before, neighbors got along well, could give a little to you, give a little to him. Now it's not possible. Now, anyway, we don't really argue much, no arguments, but it's just not as good as before, not as warm and familiar as before. Before, you'd go to their house to play, they'd come to yours. Now it's basically like in the city, nobody goes out anymore.
