# Supplementary material for: Rurality representation and changes in rural tourism destination
Source: PLoS One. 2026 Apr 21;21(4):e0347226. doi: 10.1371/journal.pone.0347226 (PMC13098982; doi:10.1371/journal.pone.0347226)
Supplement: S1 File — (ZIP) [file pone.0347226.s001.zip › supporting information/大山村漆桥村录音及转译文本/DS-JM 19.docx]

Q: Could you talk about the changes in our village over the years? You returned around 10 years ago, and it's been about 20 years... wait, 10 years since you came back. So, over these years, what changes have you seen? You can talk about any and all aspects.

JM: The environment has changed significantly. The villagers now know about waste sorting and very consciously implement the 'doorstep sanitation responsibility' system. Although there are dedicated sanitation workers, the villagers are very self-motivated; if they see trash in front of their homes, they consciously pick it up. So, people's quality has improved. On one hand, the environment has changed, and on the other hand, people's general quality has been elevated.

JM: Also, regarding caring about national affairs... in this aspect, people's cultural level has improved, they pay more attention to current affairs and politics. When they encounter problems, they don't act impulsively like before, such as getting into fights. They know to use legal weapons to protect themselves; they understand the law. How to describe income? The general living conditions of the residents have improved, living standards are better than before, and incomes are higher.

Q: Anything else, mainly these points?

JM: Mainly these.

Q: What was the village like in your memory? You lived here as a child. What was your impression of the village back then?

JM: In the past, it was relatively remote, quite out-of-the-way, and poor. Our village now... it's somewhat like a urban-rural junction, gradually becoming a bit urbanized.

Q: What do you think are the things that best represent the countryside?

JM: What best represents the countryside is the social atmosphere and customs.

Q: Is there a difference between the social customs of the past and now?

JM: I tell you, the difference... in a certain sense, there is a difference. Because the previous environment, that impoverished context, and the current environment are certainly different. Back then, there were many more illiterate people. Awareness of the law was weak. Right. And regarding environmental management, there was definitely a gap. Because back then, people couldn't even eat their fill, they wouldn't...

JM: Now, rural life isn't just about basic sustenance; they seek quality. Life has a certain quality. It's not just about eating one's fill today, but having the conditions to eat well, and everyone focuses on health preservation. The demands are higher.

JM: The social customs might have evolved from being relatively ignorant before to being more elevated now. But the relationships between neighbors, the mutual care... that simplicity and honesty are still there, just like before.

Q: Now that our transportation and environment have improved, and tourists are coming in, what impacts has this brought to the village?

JM: Impacts on the environment? Yes, there are. Because people... not everyone starts from the same point. To put it most directly, it's a matter of quality. Among the tourists, some have good quality, some have poor quality. Those with good quality are reasonable and understanding with you.

JM: Of course, the people here, our village, every agritourism household treats others with sincerity. You won't find practices like price gouging tourists, as happens in some scenic spots. This goes back to the social customs of our Dashan Village. They wouldn't say, for instance, upon seeing someone, 'My house has food' (to solicit business aggressively). They are quite genuine. They won't insist 'Come in, come in!' with excessive enthusiasm or pester you. Anyway, sitting at the door, I usually greet guests with a smile, but I never say 'My house has food'; I never utter that phrase.

Q: Very good, very sincere, but not overly enthusiastic. So, the incoming guests, probably those with relatively high quality, through mutual interaction, might even help elevate the quality here, right? What about the impact on our local pastoral scenery, ponds, or natural landscape? For example, water quality or something?

JM: Currently, no negative impact. Because, how to say, the sewage treatment aspect is handled very well now. There are dedicated sewage pipelines. For instance, we have a sewage separator. Everything from the kitchen, drains, sink wastewater goes down, and there's a sewage separator, so that aspect is fine. Now the village is equipped with full-time sanitation workers. The environment, including the pond water quality, isn't negatively affected; on the contrary, it's better managed than before. Because before, every household relied on raising pigs and such to increase income, so those feces and urine went into the ponds, right? So, comparing now and then is like heaven and earth.

Q: Any impact on everyone's behavior? For example, your dietary habits, living habits, like sleep schedules, what time you sleep or get up? Any changes in the pace of life?

JM: Probably, everyone, for livelihood... if you have guests at home, you need to get up earlier. When there are guests, you definitely have to prepare, certainly. Get up a bit earlier, it becomes somewhat busier. Without guests, you do what you like, what you enjoy, just like before. It depends on whether there are guests.

Q: And regarding the spiritual aspect? Has tourism development and the economic improvement made everyone feel prouder to be Dashan Village villagers, more identified with this place?

JM: Certainly. The overall quality has also improved.

JM: First, when you go out, and people ask where you're from, and I say 'I'm from Dashan Village', I can say that very loudly and proudly. But before, would I say it? Before, there was no confidence; now there is confidence. The reputation has also grown.

Q: No need to be polite, we're almost finished chatting. Good. May I ask your age?

JM: 51.

Q: Okay.
