# Supplementary material for: Rurality representation and changes in rural tourism destination
Source: PLoS One. 2026 Apr 21;21(4):e0347226. doi: 10.1371/journal.pone.0347226 (PMC13098982; doi:10.1371/journal.pone.0347226)
Supplement: S1 File — (ZIP) [file pone.0347226.s001.zip › supporting information/大山村漆桥村录音及转译文本/DS-JM 2-LDY.docx]

[File Name]: DS-JM 2-LDY.docx

[File Content Begin]

Basic Information (This information form is not with me):

(1) ID:DS15 (e.g., SA/DS/QQ-00)

(2) Gender:Male Age:?? Occupation:Owner of 'Xiao Rizi' Farmhouse Inn

(3) Role: √ Resident □ Tourist

(4) Education Level: √ Junior high school and below □ Senior high school (including technical secondary school) □ College and Bachelor's degree □ Master's degree and above

(5) Years of residence in this locality:?? Participation in tourism:Yes

(6) Annual household income: □ ≤10,000 □ 10,001~50,000 □ 50,001~100,000 □ >100,000

(7) Sources of household income (multiple choices): □ Farming √ Tourism-related service industry □ Others (e.g., migrant work, salaried employment)

(8) Tourist's Occupation (if applicable): □ Enterprise employee □ Professional (doctor, lawyer, teacher, etc.) □ Self-employed / Freelancer □ Student

Q: What changes have occurred in our village?

A: The village... now the main issue is the improvements related to building the 'Beautiful Countryside' and such.

Q: The 'Beautiful Countryside' renovations are mainly architectural, right?

A: I think it's been years now. Currently, the main focus is still on infrastructure. The biggest change is the asphalt roads built later. Then, the surrounding areas can be contracted to large landscaping companies, turning them into gardens. The overall environment is better, it's better for the environment, including the ponds where there are flowers and such... The key point is, originally there were companies [involved], but later now... the ponds here have been contracted out to others. To prevent water source pollution, I mean, previously they were all contracted out. It's better now. Expecting everything around here... this area still isn't great either.

Q: These things are mostly just for ornamental purposes, right?

A: Right. But the most important aspects are still the road surfaces and the landscaping. You mentioned housing renovations, that was years ago. A long time ago, around 10 years ago? Probably 4 years ago. It's okay now, hasn't been running normally for many years.

Q: What do you think a rural village should be like? Let's go back a bit further in time.

A: Go back further... Also, for us, the most important thing is the environment in and around the village center, the weeds, there are too many others, the environment was quite messy and disordered. What we just discussed was about it being neater. Now, it's relatively neater.

Q: We are developing rural tourism here, right? What elements do you think best represent the countryside?

A: The residents represent it, and them... We are different from city people. First, the farmhouse inn aspect, it's definitely different from the city, right? Then, this area is a tourist zone, the air is a bit better, better than the city air, and the space is more open. But then again, maybe the city has air too, but it's more cramped, right?

Q: Are there any differences between the characteristics of rural people and urban people?

A: The countryside now... actually, people of our generation communicating with you, for instance, in terms of population and all aspects, it's very different from before. Before, rural people were rougher, right? Now rural people are more civilized. The population is smaller, and the quality of the rural population is getting better. I don't know if it's because later... many before didn't have such high [standards?], the quality was worse before, now it's better. To be honest, sometimes the tourists staying at our homes feel that city people's manners aren't as good as rural people's now.

Q: Have there been any major changes in the farmland here?

A: The farmland has changed. How is the farmland now? Now the main focus is on flowers. As I mentioned about the characteristics of the setup, it's contracted out to a large landscaping company. But, we don't know the specific situation. To put it bluntly, it could also be a big company trying to get some kind of state subsidies, right?

> Actually, if the farmland is contracted out but not fully utilized, with many fields left idle, it's actually a bad situation.

Q: Are there still home vegetable gardens now?

A: Probably very few households have them, for example, 40%.

Q: People raise poultry now, but it's less than before, right? Fewer households, fewer animals?

A: A small portion, not much less. Because now, for environmental protection, basically everyone has signed agreements.

Q: The water quality has improved, and household waste should be managed uniformly now.

A: Household waste management is okay now. Every morning, for example, they collect according to regulations.

Q: Has the layout of the village changed? Was this area originally local to this place?

A: Now what's been done is we moved here from below.

Q: Is it residential now?

A: Let's say the overall village layout has probably expanded.

> How to explain this... it's like merging small towns into big ones. But down there, there's already a company developing tourism somewhere. Because originally, there was a village called Xiaorui Family... isn't it further ahead? Yes, half an hour ago I saw that area is empty now, isn't that the Dashan Slow City Tourist Center area? Right, all the new buildings there are newly built, like real estate, right? It might also be that the local government wants them to build uniformly. Actually, bringing us here, we weren't willing. Maybe the local government has some plan. Originally, we were building a 'Beautiful Countryside' over there, so they probably gave the whole plot to them to develop. There are still four or five households with land rights there, still undergoing changes. Seeing that area... he takes care of people... recognition... still relatively low pressure, right?

Q: Okay. Has the pace of life here become busier compared to before?

A: The pace of life has changed. Before, if you weren't busy, it was with farming, right? Now everyone is engaged in restaurants, homestays, and such. Relatively speaking, it should be much more comfortable than before, right? Before, it was daily work on houses and farmland, very laborious. For farmers to transition to tourism is definitely good.

Q: In that case, do you often go out shopping and such?

A: Should be more frequent, and more convenient. I mean, surrounding towns have those large supermarkets, right? Now maybe some young people have small investments, it's quite convenient for some.

Q: Your family also has a car, right? Have neighborhood relations changed in recent years?

A: Neighborhood relations are relatively better than before. Because before, when farmers worked the land, there were contentious issues, right? For example, during drought, they would fight over water, causing conflicts. Now there's no such work, so fewer conflicts.

Q: Is there any competition?

A: Competition definitely exists. For example, if someone's business is good... generally, conflicts don't occur, unless you have your own method. If your business isn't good, your interpersonal relationships aren't good, then there's no help for it.

Q: We are developing 'slow culture' here, right? How do you think this 'slow culture' is manifested here? Including visible and invisible aspects, behaviors, things they sell.

A: Comfortable and slow... actually, relatively speaking, it's not really 'slow', right? Actually, everything has caught up. Now almost every household has a small car, right? Anyway, how to put it... as they say, life in the countryside feels a bit slower. There's still some labor, like growing vegetables and such. I think it's reflected more in these aspects. Like the products, getting vegetables... we grow them ourselves here, from the farmland. Compared to before, it's definitely much less laborious in these aspects. But if we're talking about 'slowness' in this regard, it should be relatively slower in some aspects, the agricultural part reflects our 'slowness', not selling other things. But compared to before, or compared to the city, it hasn't slowed down that much, right?

Q: Do you think there's a difference between here and other rural tourism places you've been to before? What's the difference between here and ordinary rural tourism destinations?

A: How to explain this area... Currently, our area is still considered key/focus, relatively speaking. Compared to other more remote places, finding a location, business, and all aspects are definitely much better. Even compared to two or three, three or four years ago when you were developing, right? Now being designated as key isn't over there; it has stopped there.

Yes, the shops here in our area are still developing quite well.

Q: What did you imagine a 'Slow City' should be like? What's the difference between the current reality and your imagination?

A: Actually, the houses shouldn't be too tall, all like this, right? The most important thing is pastoral life. For instance, emotions, all aspects... like fields, country paths, made neat and tidy. Now when we go out, it's like I said before, full of weeds. For the whole area, for example, I bought this plot, the first year it was still well maintained, they planted flowers, right? This year, in the first half of the year, we called the government and said, 'Aren't you supposed to maintain it? You have so many people running around every day working on 'Beautiful Countryside', right?'

> And the whole entrance area was full of weeds. I mentioned it two or three times before they finally dealt with it. Now it means the farmers do it themselves, in the vegetable plots. Originally this area was weeds.

Q: They stopped managing it, and you started planting vegetables again?

A: Right. It should actually be... how should the western talents plan uniformly, building offline things like this... The government handles things like this: originally, my entrance area, I had already fixed it up long ago, built stone benches, a small pavilion, you could all go there to chat, right?

> Now we're waiting for the government's planning and operational scope. We've waited for years, almost 4 years now. He just... How many tourists have come to me and commented that your entrance area isn't maintained? I say, 'Sorry, just wait for the government to plan it out. If you're going to tear it down, just forget it. You know, it only cost 150,000 RMB, I don't understand, I won't say more.'

> Now, actually, maybe we can't blame the local leaders, maybe it's just our national condition. Because China's situation is, for example, if the government gives an order to do something here, they immediately start. But if it's not mentioned for a year or half a year, it will definitely stop. Policy from above has a huge impact. It feels like there are urgent difficulties. Actually, I've said, for our operations, even if you just draw the line, we don't need you to do it, we'll do it ourselves.

> Probably in November, or there's a 'Long Street Night' in November? There's a winter one in December... Actually, it's still quite present. And on March 6th or so, don't we have a Golden Flower Festival to see the rapeseed flowers? Yes, relatively speaking... I don't know much, but the scale is relatively larger.
