# Supplementary material for: Rurality representation and changes in rural tourism destination
Source: PLoS One. 2026 Apr 21;21(4):e0347226. doi: 10.1371/journal.pone.0347226 (PMC13098982; doi:10.1371/journal.pone.0347226)
Supplement: S1 File — (ZIP) [file pone.0347226.s001.zip › supporting information/大山村漆桥村录音及转译文本/DS-JM 20.docx]

Q: Ma'am, have you always lived here?

JM: My family is from here, right? I'm from Lushui, married into this village, and have lived here ever since.

Q: So it's been many years, you've lived here for many years, right? Our area has been developing tourism in recent years, right? After tourism development, what changes do you think have occurred in the village?

JM: The village has changed quite a bit. Everything has changed.

Q: What's different compared to before?

JM: Life is definitely better.

Q: Has the overall appearance of the village changed too?

JM: It's changed too. Compared to before, including the roads in the front, it's completely different now from before.

JM: Yes, different.

Q: Regarding tourism development now, do you think your village has become cleaner and more hygienic compared to the past?

JM: Much better than before. It was dirty before, now it's clean. Just like that. It's much cleaner now than before; nobody managed it before.

Q: Is the street cleaning done by dedicated people on a regular schedule now?

JM: There are two people, right? Specifically assigned to handle it, right? And what about the river water?

JM: The river water... it doesn't need managing, it's live water, flowing, right? It doesn't need people to manage it.

Q: So, with the continuous development of tourism in the village, has it had any impact on your life?

JM: Impact on life? It definitely has an impact. For example, those who have agritourism businesses, their life is certainly better. But those of us in the middle or back of the village, definitely not.

Q: Your location is stuck right in the middle, so it's not suitable for developing agritourism? Can't develop it?

JM: Even if you set it up, people wouldn't come in. They stop at the village entrance and don't come inside.

Q: So you've never started an agritourism business, right? You didn't develop one?

JM: No. Even if you invested money, you wouldn't earn any. Why would you now?

JM: That's how it is. I've always... relied on doing some odd jobs, then selling them fish... still have to do migrant work.

Q: Is it just you living at home now? Or...?

JM: Not now. My husband... he does migrant work. He goes outside the village for work.

Q: Currently, it seems tourism has a relatively small impact on you personally, right?

JM: It's not that... can't find... nobody manages it.

Q: Do you think the overall development here isn't as good as in places outside?

JM: Not as good.

Q: Are there many tourists coming here now?

JM: Not many. There's nobody now.

JM: The last couple of years... the people who came before... there were many people before, now there's nobody.

JM: A few years ago, there were many people. A few years ago, there were people every day. Last year, there were none.

JM: Last year, with the pandemic development... there were many people before, lots of people before. Now, completely none. There were many people before.

Q: So currently, are the people in your village who were running agritourism businesses still doing it?

JM: They are still operating, but truly, there are no customers.

Q: Now, with tourism development, does your family still have farmland?

JM: No farmland. It was all taken away from us. We have no farmland at all.

Q: So you rely purely on your own labor, right? Earning some money, you must work.

JM: Must work to get by.

Q: So, your annual income is probably a bit less compared to theirs, right?

JM: Certainly.

Q: Roughly how much per year? Give me a range, like 50,000 to 100,000 RMB, or more?

JM: Not that much. We can't earn much money. Currently, we can't earn much money.

Q: In the village now, are there many people like you, who aren't individually involved in tourism?

JM: Where would there be? There certainly are some, but much fewer.

JM: Before, there were people every day. Now, there's nobody. So, two or three households might have someone... but not particularly many. There were especially many before. Now, especially few.

Q: But are the people who were previously involved in tourism still at home? Are those people still at home now?
