# Supplementary material for: Rurality representation and changes in rural tourism destination
Source: PLoS One. 2026 Apr 21;21(4):e0347226. doi: 10.1371/journal.pone.0347226 (PMC13098982; doi:10.1371/journal.pone.0347226)
Supplement: S1 File — (ZIP) [file pone.0347226.s001.zip › supporting information/大山村漆桥村录音及转译文本/DS-JM 21.docx]

Q: I'll just ask you a few simple questions. Grandpa, you've always lived here, right? Your family is from here. I believe you've lived here since you were young. Roughly how many years have you lived here?

JM: 83 years old, so lived here for 83 years.

(The sun's things on the body? Early years like this, always, grandparents lived here) [Note: This part seems unclear/metaphorical in the original Chinese.]

Q: Where are you from?

JM: I'm from Anhui, but I go to school here, so I'm here. We've been on holiday, because school hasn't been in session due to the pandemic. In recent years, they've been developing tourism here. What changes do you think have occurred since they started developing tourism a few years ago?

JM: Before, you couldn't even walk on these roads. These mountains were barren, wild mountains. The roads were terrible, no proper roads before, and the sides were all barren mountains. Now the roads are fixed, these roads are paved.

JM: The outside areas were developed by the public authorities, the houses we fixed up ourselves.

Q: Having lived here so many years, what things do you think now best represent the local characteristics of your Dashan Village? What things best represent the features of your local countryside?

JM: The mountains and water, the pagoda, the reservoir – all are well developed. The roads and everything are fixed, the greenery is all done very well.

Q: What impact do you think tourism development has had on your local area over the years? For example, transportation...

JM: Now your transportation has become convenient.

Q: But looking at the environment now, it seems improved, but are there more impacts? Any negative impacts on you? For example, bad influences?

JM: No, it's brought only very good things.

Q: Including the ponds and water features in front, they are good? Is sanitation handled by people on a regular schedule now? For example, is there someone for the trash in front?

JM: The village committee people come to clean every day. There are people doing it every day.

Q: Does your family still farm the land or anything? Do you still farm?

JM: No land left long ago.

JM: Taken by the public authorities, taken by the government.

Q: Do you raise any chickens or ducks or things like that now?

JM: Can raise them. Can raise chickens and ducks now. Raise old hens for others to eat, specifically for others to eat. Can also sell them, can sell them. Hens are good, old hens are good!

Q: Talking more about now, compared to before, do you raise more?

JM: Now other families raise them. Not a huge amount, but they are for when guests come, to eat.

Q: Now, for example, on weekends or holidays when people come, does it affect your life? Can it impact your life?

JM: It's good for life. Those coming to my old place to eat, we earn money together.

Q: Does it affect you, for example, disturbing your morning rest or...?

JM: Rest is fine. No impact in that aspect.

Q: I also want to ask, because of the tourism, how are the relationships with your neighbors compared to before?

JM: Good too. Everyone is a bit better, all very good. Yes, very good to guests. No arguments or anything. Our village isn't one for arguing. Guests come, eat. If you want to eat at his place or her place, it's fine. Nobody solicits customers. The village has always had this custom.

JM: No soliciting. Whether from Nanjing, Shanghai, wherever they come from, nobody calls out to people. This custom is good. But over there, in Lüjia Village, someone comes and asks if you've eaten, that's not good. Our village has never had that.

Q: Now, because of the tourism, have your local folk customs, for example, ancestor worship or other things, changed at all?

JM: That aspect hasn't changed.

Q: For example, opera performances?

JM: We have opera every year. Only this year we didn't, because of the pandemic situation.

Q: No impact, you continue them. After starting tourism, do you earn more money now?

JM: Everyone earns money.

Q: Now, is it just you working at home, or are your children also at home?

JM: Now I'm old. Just gave it to... always to his wife... their two houses...

JM: Master, now the one doing it is the son... at that time in Nanjing City... his son also doesn't come home, daughter-in-law also doesn't come home. Who is this house assigned to?

JM: Granddaughter. The house for doing business. And he doesn't come home.

JM: Two... still have two, three... one lives in the high-rise, one often comes back, to look after us old folks. So, for us, it's like this. Quite good.

Q: Roughly how much money do you earn in a year now?

JM: Before it was okay, now not much. Others earn more, 40-50,000 yuan a year. When there were many people, they needed us to handle the cars, and handle the people taking cars. One person couldn't manage, had to hire help. Now, not many people, he manages it alone, looking after us two old folks.

Q: He can manage by himself, can keep up?

JM: He can't manage alone. One person can't manage.

Q: Now, your family per year... he alone at home, manages to earn about 50-60,000 yuan? I don't need it, things... also about 50-60,000.

JM: Looks after us, because you are also old.

JM: This house was originally yours, it's mine. Anyway, you just let him handle it. While he conveniently earns money, he can also look after you, right? Mainly he can look after you.
