# Supplementary material for: Rurality representation and changes in rural tourism destination
Source: PLoS One. 2026 Apr 21;21(4):e0347226. doi: 10.1371/journal.pone.0347226 (PMC13098982; doi:10.1371/journal.pone.0347226)
Supplement: S1 File — (ZIP) [file pone.0347226.s001.zip › supporting information/大山村漆桥村录音及转译文本/DS-JM 22.docx]

Q: Has your village changed much over the years?

JM: It changed quite significantly in the first few years, but not much in the last couple of years. The changes haven't been big recently.

Q: How long have you lived here?

JM: 11 years. 11 years.

Q: What was the village roughly like when you first arrived?

JM: The roads were small. This road wasn't even a cement road back then. The one in the front was a broken, dilapidated cement road. It's been repaired since, widened, and paved with asphalt.

JM: The most direct feeling is that the roads opened up. That's been the biggest change these years. Also, the overall environment in your village, do you feel it has changed?

Q: It has changed, right? And what elements do you think best represent your village? What things are most representative of your village?

JM: The Slow City symbol, the snail. You promote the slow-living pace, right?

Q: And because you are currently developing tourism, right? You are developing tourism here, aren't you? With the influx of funds, has there been any change to the village's overall environment, including its cleanliness?

JM: It has definitely changed quite a bit, changed a lot. Initially, we started developing around 2010. In the first few years, small vendors—us locals selling local products—would sell on both sides of this very road. Later, with planning, random street vending wasn't allowed anymore. They uniformly built small wooden stalls. If each family wanted to do this business, they could rent a stall themselves. It's like that. If you want to run your own stall, the rent is very cheap. The main purpose was to prevent disorder.

Q: Mainly to ensure cleanliness, right? Initially, it was to impose some rules, to have you operate uniformly over there, right? And now, while your village is developing tourism, does your family still farm?

JM: The land has been taken back. They planted red maple trees, ornamental flowers, things related to tourism. Very little is farmed now, right? There is some land, vegetable plots, we have some.

Q: Does your family raise any chickens or ducks now?

JM: We have chickens and ducks at home. But now you're not allowed to raise them outdoors; you have to raise them inside the house. It was allowed before. It's like the disorderly street vending—it's not allowed now either. They will catch them if you do.

Q: What about the water here? Has the overall water quality for your household improved now?

JM: The water quality isn't very good. Not very good anywhere. How to put it? It's a common issue in many places. Before, we drank water from the Dashan Reservoir. Later, it's water supplied uniformly from the Gaochun Water Plant. But it might not just be the water itself; maybe many of the pipes inside are aged, things are old.

Q: With this development, usually on holidays some people might come to visit, right?

JM: Now, does it affect your whole life? For example, for my family, there's no impact because I run an agritourism business. The households on both sides of this road do agritourism. But there are other families further in the village. It might affect them, and not necessarily in a good way. For example, if they have a baby trying to sleep, but a big crowd is being noisy, right? But for us running businesses, of course, the livelier the better, because that's how you earn money, right? For you, it might not have an impact.

Q: What about your pace of life? Is it different from before?

JM: We genuinely used to have a truly slow pace of life. Now, we are providing this concept to others, offering guests a slow-paced lifestyle. It's not exactly intentional... We ourselves have to be faster, otherwise, if we don't serve the dishes quickly, people might complain. Actually, now many guests from the city want to experience the countryside, right? People living nearby, because we all live in the countryside, don't necessarily want this particularly rural life. Rural people want to go to the city, city people want to come to the countryside. They are already accustomed to... they want this slow pace, but the speed of serving food needs to be very fast, just like in restaurants. Don't big restaurants nowadays all manage to serve food very quickly? Unlike us, where the chicken needs to be freshly killed, and then after they arrive...

JM: Furthermore, after being affected by the pandemic this year, very few people have come. It has greatly impacted your income, had a significant impact on this income.

JM: Yes, it seems this has affected many restaurants. Many restaurants in the city have closed too. They say big restaurants closed, but small restaurants are doing quite well.

Q: For example, I was just chatting with a friend, sent him my location, and the first thing he said was "Slow City". This suggests your branding has probably gotten through. Also, with the area being incorporated into the Nanjing district... it originally belonged to Nanjing, right? Now it's directly designated as a district, isn't it?

JM: Yes, more convenient. And the subway?

JM: The subway is open. Yes, the subway is very convenient for many people. Many people after retirement, they leisurely come here.

Q: Now, just chatting casually, after starting this tourism development, have there been changes to your local customs and traditions?

JM: They've become more prominent. Every tourist village needs excavate its local characteristics. Actually, some weren't particularly emphasized before. Maybe my father's generation knew more about them. But for us younger people working outside or employed elsewhere, we wouldn't come back for some holidays, only for the major ones. But it's different now. Now they work to promote local characteristics and pay more attention to these things for tourists to experience. For example, the "Wu Chang" dance existed long ago, but in later years—I married into this family 10 years ago—it wasn't practiced here 10 years ago. But before that, it was highly valued. There was a period in the middle where it wasn't emphasized. Now it's being revived because of development. This is our characteristic, your characteristic.

JM: And your local beliefs and practices are probably still pretty much the same as before, right?

JM: Yes. Haven't changed.
