# Supplementary material for: Rurality representation and changes in rural tourism destination
Source: PLoS One. 2026 Apr 21;21(4):e0347226. doi: 10.1371/journal.pone.0347226 (PMC13098982; doi:10.1371/journal.pone.0347226)
Supplement: S1 File — (ZIP) [file pone.0347226.s001.zip › supporting information/大山村漆桥村录音及转译文本/DS-JM 23.docx]

Q: So development started around 2010, with the Slow City initiative. The changes were probably quite significant then, right? They built some infrastructure and such.

JM: Now it's all pretty much the same. Living here every day, you don't really feel the changes anymore.

Q: Right. For rural tourism, what elements do you think best represent it? Anything, just say what comes to mind.

JM: I can't really think of anything... You could say home-style cooking is actually quite representative, because you can't get it in the city. Right? Now, either we raise the animals ourselves or grow the vegetables ourselves... we have a certain amount of vegetables. And chickens, things like that.

Q: These elements were probably less common before tourism development, right?

JM: Yes. Back then, people would tell you they grew just a little bit for themselves, a small amount to eat themselves. Now, with agritourism, most families who run businesses grow more, raise more chickens and such. So it's more than before.

Q: Originally, people farmed, right? And now the land...

JM: Originally they farmed, now they don't. The land was all expropriated by the state, and they give us a little rent.

Q: So your lifestyle changed because of that. Originally, you might have spent money... it was harder work. Now, if you don't farm, you have free time. If you don't run an agritourism business, you work as a migrant laborer. Those running agritourism at home get busy when guests come, and have quieter times otherwise.

JM: The vegetable gardens actually increased.

Q: Personal vegetable plots increased? For those running agritourism, can they grow more?

JM: Yes.

Q: Do they raise more poultry now?

JM: Basically, it's more than before. Here, people like us raise crabs.

Q: There are crab fields, right?

JM: Yes.

Q: Has the water quality here been affected by rural tourism development? Not thinking that more people mean more trash...

JM: Let me think... It's quite good. Because there are people managing it. Yes, we have operations management. So this aspect is better than before. The overall environment, the whole environment, this water... the water resources haven't been polluted.

Q: Water pollution is relatively less. Has the village layout changed at all?

JM: I see there's a newly built hotel over there. What was originally empty land became a hotel.

Q: So resident... does that count as commercial land or is it still our residential land?

JM: Some are residential. These are all residential, the family's own residence.

Q: Did the government fund it or did they build it themselves?

JM: All built themselves. They started with agritourism initially, later they stopped.

Q: That place, wasn't it originally said to be empty?

JM: That area is newly developed. It's from another small village, but that area was rented, and they were allocated this piece of land.

Q: So they moved over entirely? That also counts as adding some infrastructure for us, right? Weren't accommodations scarce here before?

JM: Yes.

JM: Because it was a small village, they moved over and merged into our larger village.

Q: Entire villages merging... many rural areas have this trend.

JM: Yes.

Q: How have neighborly relations been these past couple of years?

JM: Now, look, when the forest gets bigger... *[Note: This might be an idiom or incomplete thought meaning with more people/development?]* Interpersonal relationships are quite good, I think it's because...

Q: Is there any competition or anything?

JM: Not in our area. It's really very good here.

Q: Where were the social spaces originally? Like places to chat.

JM: Each household, sitting outside their own door, sitting in the pavilion, or strolling along the roads.

Q: Would you prefer to live in the city?

JM: How to say... the countryside is more comfortable, right? More convenient in a way.

Q: What about folk festival activities? Are there any?

JM: Yes. There's one called the "Jumping Horse" or something.

Q: Did that exist originally?

JM: It inherently existed here. The "Horse Lantern Dance" is a local characteristic, a heritage tradition passed down, a festive activity. Yes.

Q: Are there religious or sacrificial activities now?

JM: No.

Q: There probably were more originally, but not really anymore either?

JM: There's a temple, but of course nobody goes now, right?

JM: Fewer people go now. Some might go. Like, people in our generation, we didn't go before either, we don't know much about that aspect.

Q: How about social morals and customs compared to before?

JM: Quite good. Still fine.

Q: Here, you're developing Slow Culture tourism. What elements do you think embody this "slowness" here?

JM: So, our environment here is good, and there are no pollution sources or anything.

Q: What are the experiences of this Slow Culture, in your opinion?

JM: There are no large chemical factories or anything. Also, there are none here because of public security or background reasons... are there any large chemical factories? There are limits on visitor numbers.

Q: Is building the Slow Culture experience here different from other places?

JM: Compared to other rural tourism towns, ours is definitely different from others. This Slow City here... don't let them put it elsewhere...

Q: Probably using Slow City as the theme. Right, this is China's first International Slow City, so that distinguishes it from other places.

JM: Yes.
