# Supplementary material for: Rurality representation and changes in rural tourism destination
Source: PLoS One. 2026 Apr 21;21(4):e0347226. doi: 10.1371/journal.pone.0347226 (PMC13098982; doi:10.1371/journal.pone.0347226)
Supplement: S1 File — (ZIP) [file pone.0347226.s001.zip › supporting information/大山村漆桥村录音及转译文本/DS-JM 24.docx]

Q: Have there been no changes? Compared to before, have we changed much?

JM: The changes are significant.

Q: Can you tell us about them in detail? Please elaborate on what it was like originally and what it's like now.

JM: Originally, there were no proper roads. Now, transportation and roads are much more convenient. Some were dirt roads before, now they are asphalt.

Q: Anything else?

JM: Originally, people farmed. Now they don't. They don't farm now.

Q: What elements do you think best represent the countryside? We are developing rural tourism here; we are certainly different from the city. How are we different from the city?

JM: The city changes relatively quickly, while our countryside changes relatively slowly.

JM: Before, Yaxi was like this, and isn't it still like this now? Because the capital investment is somewhat less, and later there hasn't been much development; development is relatively slow. Economically, it definitely can't compare to the city. Our development here is relatively slow.

Q: Right, and aren't we also promoting 'Slow Culture' here?

What elements do you think embody this 'Slow Culture'? This is a 'Slow City', right? How is the 'Slow City' concept reflected now?

Q: Are vegetable gardens still being cultivated here?

JM: Vegetable gardens are cultivated. The rice paddies are definitely rented out to others.

Q: Is poultry still raised?

JM: Raising poultry... probably less. Compared to before, it's definitely much less, much reduced. Originally, people might have raised more, maybe three types.

JM: Things like raising pigs, cattle... they raised everything. There were many cattle and sheep. Now, they just raise a few chickens. The variety of poultry has decreased, and the numbers have also decreased. Yes.

JM: These livestock... they cause pollution.

Q: If there were many of these, it would mean pollution, unpleasant smells, and tourists wouldn't want to visit. It's not suitable for running businesses, right?

Q: Does your family run an agritourism business?

JM: My family doesn't. I'm old now, an old person.

Q: Couldn't your children come back to do it?

JM: One child does it, just up ahead.

Q: Has the river water quality gotten worse in recent years?

JM: We all use tap water. The water quality... the quality in the ponds now is definitely worse. With more agritourism businesses, more washing happens... compared to before, it's definitely a bit worse, a bit worse than before.

Q: Our domestic waste?

JM: Domestic waste is a bit more. There is more, but they clean it frequently, they keep it quite tidy. It's still pretty good.

Q: Has the village layout changed at all? The overall layout of the village, the buildings in this area? The mountains are in front, we're all at the foot of the mountains. Any changes? What changes?

JM: The houses are improved. Houses have expanded outwards, or built new courtyards and such. Living at home is very comfortable now. Air conditioning, electricity, fans, everything is very complete. For us, it's definitely better.

Q: What is our pace of life like? Originally, it might have been slower because of farming, but also busier at times?

JM: At that time, we started work tired, and when we came back we still had to raise pigs, chickens, sheep... just managed to get by. Now we are not as busy. Now it's more leisurely. We don't have to do that work now; they don't allow you to do it anymore.

Q: Originally, shopping options were probably fewer, now there should be more?

JM: Yes.

Q: Your family probably has a car now? Is transportation more convenient?

JM: It's definitely more convenient. Originally, there were public buses.

JM: And there are more markets now.

Q: Were neighborly relations better originally? Originally, in the countryside, neighborly relations involved mutual help...

JM: It can't be exactly the same. If there's some conflict, then it's different. That's not right either... If there's no conflict, it's good. If something happens with a neighbor and it doesn't go well, then there's conflict. Yes, it can't be guaranteed... there were conflicts before, and there are conflicts now. Neighborly relations are just like that. If nothing happens, relations are very good; if something happens, then it's not right... If there's no conflict, everyone is fine; if there's a little conflict...

Q: Where were the social spaces originally? Where did villagers chat? Where did we all used to go?

JM: We don't have that here. No.

JM: Now there isn't either. There isn't now. Originally, there was something called 'walking the paths' or something... in the morning you might walk and talk. There wasn't a specific place just for chatting.

Q: Have customs for weddings, funerals, and such changed at all?

JM: No change. They are all pretty much the same.

Q: Have social morals become worse because of the changes in recent years? Has it become more economically focused? Originally, people might have valued loyalty more.

JM: Indeed, that's true. Now it seems more commercialized.

Q: What about the rural atmosphere? Was it perhaps better originally? Now, with competition and such, is it more modernized?

JM: This is definitely true. Competition exists. After his family does well, my family wants to do better than his.

Q: Are neighborly relations affected because of this?

JM: Not really significantly affected.

Q: May I ask your age?

JM: 75.

Q: The education level of residents should be... right.

Participating in tourism... May I ask about your family's annual income?

JM: Should be about 100,000. Now I have no income. Government subsidies... how much? 400 RMB per month. Roughly four to five thousand a year. From farming. And you don't run an agritourism business, right?

JM: No.

Q: Let me ask you, this area is a 'Slow City', right?

What kind of cultural experiences does this 'Slow Culture' offer? Have they built any Slow City landmarks, do you know? Like snail sculptures or something?

JM: There is a snail.

Q: Also, has there been any revival of folk culture? Like 'Tiao Ma Zha', 'Tiao Wu Chang', 'Da Ma Deng' (local folk performances), are there any?

JM: No.

Q: Handicrafts? Are there any handicrafts sold, those kinds of things?

JM: Some old people make a little. Now people don't know how, children don't know how to make them either.

Q: The ecological environment has seen some improvement. Have characteristic festival activities increased?

JM: Not really increased. There aren't many distinctive festival activities. There are some additions... there is the Jinhuajie (Golden Flower Festival), and there is the Changjieyan (Long Street Banquet).

Q: When are these held?

JM: Jinhuajie is in March. Changjieyan is in January.

Q: How do you feel about these cultural experiences?

JM: They are quite good.

Q: Can you experience this slow pace of life?

JM: Basically, that's the result.

Q: You can experience the slow pace of life, there's a bit of a rural living atmosphere.

JM: Yes.

Q: Do you think this place is different from other tourist destinations?

JM: I haven't been to other places. I don't go out, don't look, don't know what it's like outside.

Q: What do you think the cultural experience of 'slow tourism' should be like?

JM: What should the cultural experience of slow tourism be like? This area is a Slow City, right? What do you think a Slow City should be like? We don't know either.
