# Supplementary material for: Rurality representation and changes in rural tourism destination
Source: PLoS One. 2026 Apr 21;21(4):e0347226. doi: 10.1371/journal.pone.0347226 (PMC13098982; doi:10.1371/journal.pone.0347226)
Supplement: S1 File — (ZIP) [file pone.0347226.s001.zip › supporting information/大山村漆桥村录音及转译文本/DS-JM 25.docx]

Q: Are you a local? Being a local, you should be quite familiar. Not entirely clear? Well, since they've been developing tourism here, has it brought any changes? What changes has it brought?

JM: Changes? There are some. For example, us elderly folks can't find work outside anymore, but at home we can run agritourism businesses. It's good. It has enriched our lives a bit; now we have something to do.

Q: So, what was the village like in your memory?

JM: Before, when we were at home—we haven't been home for many years—back then, there were no roads, actually there was nothing really, just a farming village. And when there was no rain? There was no water either. Now we don't farm anymore, right? It's not that there's no water for farming, we just don't farm now. At home, for old folks like us, we can manage things at home, earn some living expenses. It's much better than before.

Q: What things do you think best represent the countryside? Aren't we developing rural tourism here?

JM: Yes, it's good for the village. Most households, this whole row of ours, are agritourism businesses. We manage things at home, no need to go out for migrant work. Most are running agritourism at home. Even some young people don't go out anymore. Like us, being a bit further inside, while they run food services over in the residential houses. Their meal business is pretty good. We don't serve meals, we just do accommodation, and business is also good. They will bring people down to your place. Right now, not many people come during the hot season, but later it should pick up quickly, probably quite a lot. In October, there will definitely be more people; the rooms are almost all booked already.

Q: Do you think there have been major changes here? Besides that, about the fields—are they still being farmed now?

JM: The fields here, we don't farm anymore. I'm not sure what they are doing with them, something like planting flowers, or planting trees. In previous years, they didn't plant rice, but this year they planted rice. Before the pandemic, wasn't stuff imported from abroad? Now they don't import from abroad anymore, so China does it itself, grows its own. So they started planting.

Q: Do you still raise poultry now?

JM: We don't really raise them anymore. Before, there probably would have been more. The countryside has changed a lot in the last couple of years.

Q: Do you feel it's different from the rural village in your impression?

JM: The countryside now is very different from before. Yes.

Q: Was the countryside better before or now?

JM: It's better now. Makes living conditions more comfortable. Yes. Before, there were no roads... Our village was relocated from the village down below. Before, there were no roads. My two children went to school; on rainy days I had to carry the bicycle out to the road. The road wasn't even like a proper road, just stones and gravel, pieces this big. That was the path. Now there are roads. Every household has road access at their door. Much more convenient. This place originally, what was it? Farmland? Now it's developed as... how is it classified? Residential housing.

Q: Agritourism is considered... not commercial land use?

JM: No, it's not. We were relocated here. They resettled us in this place. We were from the small village down below.

Q: Have your living habits changed? Like, if you were farming before, it would...

JM: Habits are similar. But people don't have to do that work now. Getting up later doesn't matter. If you don't get up, it's fine. It's voluntary. Before, you had to get up early in the morning to work.

Q: But do you often leave the village to go shopping or such? Could there be more?

JM: We need to. The countryside doesn't have much for sale. We often go out, every couple of days we go to town to buy things and come back. The transportation is more convenient than before. Transportation is convenient. Later, we all drive ourselves, it's quite fast.

Q: What about neighborly relations? Any impact on neighborly relations?

JM: Seems not. My family hasn't experienced any. Our relations are quite good. We've always been villagers together, relatives and friends, all quite good.

Q: Have you bought property in the city? Do you think about living in the city, or prefer living here?

JM: We think about living here now. When we get old, also... yes, we want to stay in the original location. When you're old, if buying food is inconvenient, or cooking for yourself? It's more comfortable here. When you're old, wanting to buy groceries or something, having to run to town is very far. Now it's not a big deal, we can still manage. Now we grow some vegetables at the door, no need to buy. Buy some meat, buy some fruit, everything is quite convenient.

Q: Are there any new things here because of tourism development, like festivals, handicrafts, etc.?

JM: Handicrafts, I'm not clear. Actually, I'm from this village, but we haven't been home for about 20 years. Our house was demolished, we lived in the house built there, came back to run the agritourism business. For about 20 years, we worked as migrant laborers outside.

JM: There is some festival activity here, seems the more famous ones are the Golden Flower Festival and... the Golden Flower Festival and the Long Street Banquet. Actually, there are quite a lot of people, these two festivals are relatively bigger.

JM: Don't know how many people come for these two festivals. For the Long Street Banquet, a group of friends from Nanjing—actually, they are from this village—they've been organizing it for 7 years, or 6 years, I don't know. We didn't know either. Later they called us and said it's really fun, come over. They said every year you organize this, you must call me. They said they had never seen anything like it in all their lives, said they'd never seen so many people, so much fun. Now, in our village, my family was still busy that day, didn't go out to see. My daughter-in-law and son went, they said it was really fun. We ordered meals. They were on the road ahead, from the east side, tables set all the way down to the back village, they were set up too. Don't know how many.

Q: Your family also booked a spot there?

JM: We just wanted to eat, friends came. Our place, we don't cook ourselves, right? So we could order meals ourselves.

JM: Went over to the roadside. It was great. If you like eating on the street, you eat on the street. If you prefer eating at home, the agritourism families also set up tables at home to eat. Actually, eating outside is more fun.

Q: And there are opera performances?

JM: Yes, opera. Seems they said dozens of media were behind, near the Slow Visitor Center. Good. They took photos and sent them to our phones. Actually, busy people didn't go out. Lots of people came, family came and went, we also had to clean rooms, receive people, so we didn't go out. Good.

Q: Are there any other cultural experiences? This area is promoting slow tourism, right?

JM: Cultural stuff, you need to ask the older folks. We don't know. We only came to this village two years ago. Actually, our family business is not bad. When busy, we don't go out much. These past few days, yesterday and today nobody, tomorrow there will be people. Yesterday we had no one, so we went out to play, found time to go out. Only came home last night.
