# Supplementary material for: Rurality representation and changes in rural tourism destination
Source: PLoS One. 2026 Apr 21;21(4):e0347226. doi: 10.1371/journal.pone.0347226 (PMC13098982; doi:10.1371/journal.pone.0347226)
Supplement: S1 File — (ZIP) [file pone.0347226.s001.zip › supporting information/大山村漆桥村录音及转译文本/DS-JM 26.docx]

Q: Have there been significant changes?

JM: The changes are huge.

Q: What kind of changes?

JM: Look at our village's appearance these past few years; it's changed a lot. It used to be gravel roads, now they've been changed to asphalt, and some are cement now.

JM: The infrastructure has improved. Roads and everything are better. Asphalt roads have been built, and street lights are installed.

Q: And public buses are running now too.

JM: Yes.

Q: What are the bus schedules now?

JM: They start around 7:00 or 8:00 in the morning, and run until 7:40 in the evening... 7:40 PM... and until 2:40 PM in the afternoon. There are buses throughout.

Q: What was the village like further back in your memory?

JM: The gap compared to now is huge. Back then, it should have been all fields and such. Yes, everywhere was dirty. Now it's all kept clean. Before, trash was everywhere, all over the place. Now the environment and everything is better, including the greenery; the whole area is nicer now.

JM: In the past, nobody managed it.

Q: Has our lifestyle changed? Was it more leisurely before when we farmed? Now, with people running agritourism or small shops, do they get busier?

JM: Now it's busy too. With agritourism opened in the village, every household has become busier.

Q: Since we're developing rural tourism here, what elements do you think best represent the countryside? The elements that best represent rural tourism. We are different from city people, right? Our distinctive features might be that villagers are simpler and more honest, and the pace of life is slower.

JM: Yes, that's right. Yes.

Q: And our natural scenery is better because we have mountains and water.

What is your ideal countryside? Do you have a picture of an ideal village in mind?

JM: Before, the houses were demolished but not improved, everything was dilapidated. Now the government has developed it, everything is renewed, everything is much better.

Q: Before, people farmed, but now the farmland has probably decreased, right?

JM: Now all the farmland has been taken back, all rented out. Whatever they do with it, we don't manage, they just give us money.

JM: But vegetable gardens are still cultivated.

Q: Is there still poultry?

JM: We can't raise much poultry either. It's a tourist area, they don't allow raising many.

Q: Because it affects the environment, right? So now people raise less.

JM: Raise less. People might raise a few for themselves to eat.

Q: Has the water quality in rivers and lakes changed?

JM: The water quality has improved. Now, every household's wastewater and such is channeled to septic tanks, to the sewage treatment plant. This situation has improved.

Q: Has domestic garbage decreased too?

JM: Domestic garbage... someone cleans every day, someone collects it.

Q: Has the village layout changed due to rural tourism development? Has the built-up area of our village increased?

JM: Basically, it's okay, hasn't increased much.

Q: A hotel was built over there behind. What was that place originally?

JM: The state is developing it, it's done by the government.

Q: That place was also residential housing before, right?

JM: That piece of land had no residences, it was empty land. Then new houses were built. Yes, you could say the government has overall expanded our village layout.

JM: Yes, the infrastructure.

Q: Have our social spaces changed? Where did you chat before, and where now? Before tourism development.

JM: Chatting... we could chat everywhere while strolling, on the roads, sometimes chat at the square. There's a square at the back, this place is also a square, and also including... yes, the village has squares. In the evening, people chat here.

Q: Have neighborly relations changed? Is there competition because of rural tourism development?

JM: There is competitiveness, it's somewhat competitive. But no major changes. It's all good-natured, everyone is harmonious, it's still fine. Even though there's competition, the relationships are still good.

Q: What about folk festival activities? Any changes?

JM: They've increased. After work, they organize some cultural activities, things have increased.

Q: Probably because your government develops them with you.

This area is also a 'Slow City'. What kind of 'Slow Culture' experiences does it offer? What is 'Slow Culture'?

JM: We don't really know much about that either.

Q: For example, those symbols, like the snail symbol? How is that implemented, right? For slow tourism, right? Are there any other things, like organizing festivals?

JM: Yes, sometimes cultural... like what our older generation left behind, like 'Da Cha' (maybe a folk performance), weapon performances or something. Every year in the second half of the year, around New Year, they have to come out and perform. Still happens during the first month of the lunar year. Yes, and sometimes in the past it happened during ordinary times too.

Q: There's the Long Street Banquet. Has there been any revival of traditional handicrafts?

JM: Yes, there are. Like cakes, rice cakes, 'Tuanzi' (glutinous rice balls), fans, all sorts of things. 'Qing Tuan' (green dumplings) are a local specialty here. Yes, Qing Tuan, eaten during Qingming Festival.
