# Supplementary material for: Rurality representation and changes in rural tourism destination
Source: PLoS One. 2026 Apr 21;21(4):e0347226. doi: 10.1371/journal.pone.0347226 (PMC13098982; doi:10.1371/journal.pone.0347226)
Supplement: S1 File — (ZIP) [file pone.0347226.s001.zip › supporting information/大山村漆桥村录音及转译文本/DS-JM 3.docx]

Q: (It's been) year after year (increasing). You're not from around here, are you?

A: This area is a bit remote, but I'm still considered local. I'm just not from this immediate vicinity.

Q: What do you think are the feelings and experiences of 'slowness' in the Slow City?

A: Well, here our pace of life is relatively slow. The pace of life is slow.

Q: What cultural experiences do you think it provides for tourists? And, what do you think culture is? What is slow culture? And are there any issues with it afterwards?

A: Locally, for instance, we have the "Jumping Wu Chang" here, we have some performances. It's one of our local folk customs. Our Dashan Village is also a folk custom village. You can see it everywhere, like those buildings, and there's a Rui Family Ancestral Hall inside. There are local characteristics everywhere, so probably more people can experience and see them.

Q: Did these exist before as well?

A: Before, let's say the ancestral hall existed, but it wasn't promoted or publicized much. Also, this village was later renovated to be like this. Originally, the houses probably weren't like this, similar to other ordinary places. It was developed later, and they built those 'horse-head' gable walls. These buildings are very distinctive. That's quite good.

Q: How do you think the promotional work for the Slow City is done? Has there been a lot of effort to promote it?

A: The promotion is okay. Because our main focus here is eco-sightseeing, and also some experience projects developed later. Excessive promotion isn't too... particular? So, many people come here, find it nice, and then come back a second or third time. It's quite good here, very relaxing. For example, if you stay here for a day or so, like now, getting up for a morning run, the air is very good. Many tourists come several times a year.

Q: What entertainment facilities have been built?

A: Entertainment facilities... as mentioned earlier, those projects, like the rainbow slide, were also developed later. They didn't exist before. Before, there was nothing to see or do like this.

Q: What differences do you see between the local cultural development and other rural tourism destinations?

A: Locally, our Gaochun elements naturally have some differences compared to other places. But specifically, I haven't been to other places, so I don't have a basis for comparison.

Q: If you were a tourist going elsewhere, suppose a place promoting 'slow tourism', what do you think slow tourism should be like?

A: Suppose you go to another place, if you weren't in the Slow City, what should tourism be like in your mind? It should mean not overly commercialized. Then, having local original inhabitants and such. Local features definitely shouldn't be over-developed. It should be relatively quiet and serene. Yes, that would definitely be better.

Q: Do you think Gaochun has any areas that need improvement?

A: I can't really say.

Q: Can you give your age? Above 30?

A: 30.

Q: What was the countryside like in your memory, and what is it like now?

A: In memory, you mean like when we were kids? Right, just talk about your childhood. When we were kids, you could see animals everywhere, like lobsters and such, which you can't see now. Mainly animals and those things. Like, we could go everywhere catching dragonflies and such, now you can't see them anymore. Because the fields were consolidated, it's probably not like before. In summer, you could hear cicadas, now it's very rare.

Q: What elements do you think represent the countryside now?

A: Now, it definitely looks more standardized. Like those fields are consolidated and quite standardized. In the past, they were more scattered and uneven, it's different now. Rural construction is quite good now. Like the garbage, the living environment for residents – before there was no place to throw it, people just dumped it anywhere randomly. Now, the environment is handled a bit better. Before, if you had no place to dump trash, you'd just throw it everywhere, right? Now there are facilities. The village is cleaner too, and there are people responsible. People themselves are more aware, we also do better.

Q: What do you think the ideal countryside should be like? Think about it.

A: I can't think of anything right now, I don't really have any ideas.

Q: Do you think transportation, information, capital, and tourism have all poured into the countryside? What impact has this brought to the countryside?

A: It has brought convenience, for sure. The roads are built better. In the past, there were muddy roads that were hard to travel on. It has also promoted our local economic development. If those roads weren't fixed, who would be willing to come here?

Q: Do you think the impact on things like pastoral scenery is significant? Pastoral landscapes, rivers, lakes, ancient streets, and lanes.

A: The impact isn't very big. They consolidate things. It's probably just that the appearance isn't like before. Because some places have different development plans. For example, the Slow City aims for an original ecology. Other places, like our area, will definitely have unified farming arrangements for people, so it's certainly different from before. It seems the policies are different for each place, it's hard to say, really.

Q: Do you think there have been any changes in behavior? Behavior after tourism development, like changes in life pace, art practices, shopping, things like that. Neighborhood relations, you can talk about any of these, just speak freely.

A: Now, it has become a bit better, somewhat improved. Before, even if it was convenient, you wouldn't go out much or anything. Now with development, people go out more. Also, since this area is building a Slow City, the villagers' quality... our caliber has been raised. It's not just limited to this spot, but this whole large area up to now.

Q: What about spiritual elements, like farmers' identity? For instance, after tourism development, do people feel 'my hometown is really good', that kind of feeling? Or folk festival activities, cultural confidence – anything you want to share about before and after tourism development? These belong to the spiritual level.

A: Before, it probably wasn't as complex as you're describing.
