# Supplementary material for: Rurality representation and changes in rural tourism destination
Source: PLoS One. 2026 Apr 21;21(4):e0347226. doi: 10.1371/journal.pone.0347226 (PMC13098982; doi:10.1371/journal.pone.0347226)
Supplement: S1 File — (ZIP) [file pone.0347226.s001.zip › supporting information/大山村漆桥村录音及转译文本/DS-JM 4.docx]

[File Name]: DS-JM 4.docx

[File Content Begin]

Basic Information:

(1) ID:DS04(e.g., SA/DS/QQ-00)

(2)Gender:Female Age:33 Occupation:Owner of Fangfang Farmhouse Inn

(3) Role: √ Resident □ Tourist

(4) Education Level: □ Junior high school and below √ Senior high school (including technical secondary school) □ College and Bachelor's degree □ Master's degree and above

(5) Years of residence in this locality:33Participation in tourism:Yes

(6) Annual household income: □ ≤10,000 □ 10,001~50,000 √ 50,001~100,000 □ >100,000

(7) Sources of household income (multiple choices): □ Farming √ Tourism-related service industry □ Others (e.g., migrant work, salaried employment)

(8) Tourist's Occupation (if applicable): □ Enterprise employee □ Professional (doctor, lawyer, teacher, etc.) □ Self-employed / Freelancer □ Student

Q: May I ask how many years you have lived here, and since when have you been here?

A: I am from here. I've lived here since I was born, grew up here.

Q: During the development of slow tourism, what cultural experiences do you think are provided for tourists? The Slow City has its slow culture; what do you think slow culture is? For example, cultural settings, cultural landscapes, or folk culture?

A: Our Slow City here is a place that is self-sufficient, without large-scale industry, without large supermarkets—a place with a slow life and slow pace.

Q: What impact does this rhythm, this lifestyle, bring spiritually or sensorially?

A: For us, it's still okay. After developing tourism, the 'slow life' has actually become a faster pace.

Q: Are there any other changes you feel, personally, within your family, or for yourself?

A: It's okay. We used to work outside, but now relatively more young people have returned.

Q: What differences do you think exist between the establishment of the Slow City here in Gaochun and other rural tourism destinations?

A: Regarding the difference, I think it's okay. Because it was originally developed as a 'Beautiful Countryside'. By chance, a governor from Italy visited and thought it was quite similar to the first Slow City town they developed, so it was named China's first International Slow City.

Q: So you think perhaps the Slow City isn't very different from other rural tourism spots in China?

A: Probably quite similar.

Q: What form do you imagine slow tourism should take?

A: It shouldn't be so fast-paced; it should allow for relaxation of body and mind. Actually, I think it's right that the Slow City doesn't have too much entertainment. It's about a slow life, slow pace, emptying your mind, putting down your phone, and staying here for a few days. It seems like it's just farmhouse inns now, right? Letting your life rhythm and mood slow down. Have a small quiet bar, a small tea house, sit for a while, stay for two or three days, completely disconnecting from the outside world. But how many people can actually do that now, right? Not to mention young people, they still feel there's not much to do here, right? They come and immediately ask, 'What fun things do you have here?'

Q: Actually, the Slow City should be like that. What discrepancies do you see between the current development of the Slow City and your expectations? What needs improvement?

A: The overall planning is still not tidy enough. The area is too vast, making management difficult.

Q: Okay, can you recall what the countryside was like in your memory, and what changes have occurred in the present countryside?

A: Before, the roads were all dirt roads, mud paths. Now they are all asphalt roads. This change probably isn't unique to our Slow City; it should be like this everywhere.

Q: What elements do you think best represent the countryside? Specific symbols, markers, or memories that signify the rural areas?

A: Smoke curling up from kitchen chimneys, children playing and frolicking, the feeling of enjoying the cool air after dinner in the summer. Yes, it's rare now.

Q: If we consider some aspects of standard rural life now, what do you think they are?

A: It's not very obvious now. The rice fields are gone, the vegetable plots are gone. It's really not very prominent now.

Q: What impacts do you think transportation, information, capital, and tourism have brought to our Gaochun Slow City?

A: Farmers' income has increased. Transportation is still okay, but our transportation here isn't very developed.

Q: During the development of the Slow City, can you, for instance, still maintain the past habits like early to bed and early to rise, or keep that life rhythm? Is it because of the farmhouse inn business? Do you think it might be that the younger generation's inherent lifestyle simply cannot slow down?

A: (Implied by context: Possibly, the demands of running the business and modern life make it hard to maintain the old slow rhythm completely, especially for younger people.)

Q: Regarding your identity as a farmer, including folk festivals, clan rituals, moral customs, etc., what major changes do you perceive between the past and now?

A: Not really. There haven't been major changes. And to be honest, we young people don't really understand these things much either. It's not very prevalent. Even my father's generation probably isn't very clear about them anymore.

Q: I assume you have also traveled to many other rural areas for tourism. What aspects do you think need correction in rural tourism destinations, or what is your impression of the countryside?

A: For improvements here, the situation is different for each place. It's also quite difficult.

Q: May I ask your approximate age? No need to be too precise.

A: 33.

Q: What is your education level?

A: High school.

(On the phone)

The vegetables and grapes are available now.

Either is fine, or I can send you the location of the fruit-picking place.

What are your plans for tomorrow?

Do you want to go fishing?
