# Supplementary material for: Rurality representation and changes in rural tourism destination
Source: PLoS One. 2026 Apr 21;21(4):e0347226. doi: 10.1371/journal.pone.0347226 (PMC13098982; doi:10.1371/journal.pone.0347226)
Supplement: S1 File — (ZIP) [file pone.0347226.s001.zip › supporting information/大山村漆桥村录音及转译文本/DS-JM 5 小日子-LDY.docx]

Basic Information:

(1) ID: DS20 (e.g., SA/DS/QQ-00)

(2) Gender: Female Age: 32 Occupation: Tourism-related interpreter and planning staff

(3) Role: √ Resident □ Tourist

(4) Education Level: □ Junior high school and below □ Senior high school (including technical secondary school) √ College and Bachelor's degree □ Master's degree and above

(5) Years of residence in this locality: 32 Participation in tourism:Yes

(6) Annual household income: □ ≤10,000 □ 10,001~50,000 √ 50,001~100,000 □ >100,000

(7) Sources of household income (multiple choices): □ Farming √ Tourism-related service industry □ Others (e.g., migrant work, salaried employment)

(8) Tourist's Occupation (if applicable): □ Enterprise employee □ Professional (doctor, lawyer, teacher, etc.) □ Self-employed / Freelancer □ Student

Q: Our village is developing tourism, right? What changes do you think have occurred?

A: Many, huge changes. One could say earth-shaking.

> For instance, about 15 years ago, this was the poorest village in the entire vicinity, but now it's the wealthiest, richest village.

> For example, my mother told me that before, when raising pigs, because the mountain roads were inconvenient, transportation was very poor, there was this situation: we might raise a pig here. If it sold for 80 RMB outside, here we might sell it for 60 RMB – just an example – but people still weren't willing to come buy it because of the 20 RMB... Yes, with this example you should understand.

Q: What was your impression or blueprint of the rural village in the past?

A: I think now, whether it's our area developing tourism or some villages ahead that haven't been developed – villages not developing tourism – both transportation and the basic living conditions for farmers have improved significantly compared to before.

Q: Infrastructure-wise.

A: Infrastructure has improved a lot. Because now, whether it's the government or others, these infrastructure constructions, roads, and such have all seen great improvement and enhancement.

Q: What do you think a rural village should be like? One developing rural tourism.

A: I think our area... compared to the past, it's still good. But one bad aspect here is the fiercemalignant competition. And if you compare it to other places like Moganshan, it can't compare. Not to mention Moganshan, even places like Xuancheng in Anhui... For instance, our village belongs to Yaxi Town. The government developed this area because it has a historical and cultural allusion. That's why they chose our Dashan Village, including the Wenfeng Pagoda you see, and those like the Bronze Gong Well – not sure if you've seen them – and some village history. Because you must unearth cultural elements to make it succeed. So, we developed this village and renovated it because of these stories. Of course, government investment so far is nearly 50 million RMB, so it's the way you see now. This year is the 11th year of development. Currently, our government's focus is here. Actually, I don't know if you've been there these past two years... Earlier, I could recommend you go to a place called Xiaomaoshanjiao Village; it's now one of the typically well-developed villages here. Another is called Longshang Village. Because last year when I was doing government reception work, they also came to visit the 'Beautiful Countryside', and basically, the routes I led included going to Xiaomaoshanjiao Village. There, it's typical collective action boosting villagers' income. It's mainly driven by enterprises settling here and taking shares. Also, I don't know if you've seen one called Longshang Village? It's a very distinctive village here, different from us. We mainly operate in the farmhouse inn model, but they don't. There's a company called Slow Investment Culture Co., Ltd. there, which is long-term stationed and manages it. They operate in a corporate form, so their situation is relatively better done, and it's a very ideal rural model in my mind.

Q: What is its general scene like? Not like this uniform packaging and hanging lanterns here?

A: No, it's different from us. They don't have commercialization as severe as here. Here, you see farmhouse inns... because people, when they hear 'farmhouse inn', think it's relatively low-end, with not very high consumption levels. But there, it's mainly accommodation; they do high-end lodging. Here, we added the RV park behind, slightly more upscale, you've seen it, right?

> The RV park was also developed here in '16. Now their business is very good, already into its second phase; I heard they are negotiating a third phase, but I'm not sure of the details. Also, we originally developed a homestay village called Xiaorui Family Homestay Village, relocated from our village. Originally, because everyone had the surname Rui, it was planned to be developed like Dashan Village. But because we are from there... our village had 28 households, and basically the whole village relocated here, and that area was converted into a homestay village. But now, I don't know the situation, it's been years.

Q: How much does high-end homestay cost?

A: For the more high-end ones, like the RV park and those in Longshang, there's also one called Mijing, and another called Peach Blossom Valley. The boutique homestays there cost over 1000 RMB. It feels a bit like a secluded paradise, I quite like that feeling, somewhat similar to Moganshan. Quite boutique, whether in environment or other aspects, including dining, the restaurant atmosphere is different, very similar to Moganshan, because I've also been to Moganshan to study. Their consumption level, the whole vibe is higher, the tone is elevated, yes.

Q: So, they have much more to play and see there than here?

A: Yes. What's it like there? They started with accommodation first. After establishing accommodation – it was a company called Slow Du at the time – they acquired 13 civilian houses first... Let me clarify how they did it: they directly bought out the local villagers, 20,000 RMB per house. They were indeed very dilapidated. But the buyout wasn't random, like buying any house like mine. What kind of houses were they? Mostly the wooden structure houses we lived in as kids – I don't know if you've seen that kind of wooden house. There, including their tourist service center, it's also comprised of 13 civilian houses. After renovation, they have a dedicated café, a tea house, homestays, and now they've also opened a restaurant, quite high-end.

Q: Is it also within the Slow City area now?

A: It belongs to Dongba Town. We belong to Yaxi Town. Because Gaochun is now developing all-for-one tourism. What's all-for-one tourism?

> Initially here... have you been towards the urban area these past couple of days? That area we call the 'Fishing Countryside'. We used to divide Gaochun into two: we have many mountains here, called the 'Mountain Countryside'; that area has more water, more aquaculture, we call it the 'Fishing Countryside'. Because initially, we focused on... Dashan Village was the first place where tourism developed, so we call it the birthplace of the International Slow City, right here.

> Later, around 2015 or 2014, Gaochun started developing all-for-one tourism: Mountain Slow City and Water Slow City developing overall. It includes Dongba Town, and our nearby Youzi Mountain Scenic Area – because Xiaomaoshanjiao Village is right under that scenic area. Why did Longshang develop? Because a TV drama called "In the Name of the People" was filmed here. There's a 'Ten-Thousand-Mu Tea Plantation' there. Gaochun has a plan called 'Five Ten-Thousand-Mu': ten-thousand-mu tea plantation, ten-thousand-mu economic forest fruits, ten-thousand-mu flowers and seedlings, etc. The ten-thousand-mu tea plantation is one of them. And at that time, coincidentally, the drama filmed there and brought attention to the spot, called 'China's Most Beautiful Tea Plantation'. Indeed, the green tea produced here is quite famous, even at the central level, for its taste.

> Later, gradually, they built such a village based on tea culture, with Longshang relying on the tea plantation.

Q: Do many people go there?

A: Their model feels somewhat like customized service.

They mainly receive high-end government clients, mostly. For tourists, unless money is no object.

Q: What elements do you think best represent the countryside in developing rural tourism?

A: Representing rural elements in developing rural tourism? I think it's still the environment. If our natural environment weren't good, tourists wouldn't come. Clearly, the air here is different from the urban area, especially after rain. And our environmental protection efforts, including by the government, are quite strong. After we started developing here in 2010, garbage incineration stations were not allowed within a 10-kilometer radius.

Q: What about the economic, cultural, and spiritual aspects? Our village is also rich in cultural relics, right? The economy...

A: Regarding cultural and spiritual aspects, I think it's relatively rich. Like the Heaven and Earth Stage, this has always existed since I don't know when, since my childhood, there have been temple fairs and such. As for the cultural aspect, I think... I think it's related to people's sense of happiness. Before, if you were tired from farming every day and couldn't earn much money, who would care about culture, right?

Q: Now, can there be some revival? With better conditions, some work can be revived, including venues, etc.

A: Our cultural aspect is also currently under exploration. Because our tourist numbers have been relatively low these past two years, we are slowly... our local village committee is still focusing on this, because without doing anything related to tourism, it's impossible to attract tourists.

Q: Although the environment is still there? Culture is more special.

A: Yes, but it can be delved into deeper. But to be honest, our cultural and historical aspects here aren't very abundant, that's my current feeling, not particularly plentiful, especially compared to others.

Q: But efforts are being made to excavate them, aiming to retain tourists, right?

A: Yes. Because our situation here now should be considered... I know around 2013-2014, it still reached a peak. What was the peak state like? The main street in front was so crowded that if the front didn't move, the back couldn't move.

Q: Why did such a phenomenon occur at that time?

A: Because at that time, the Slow City... this is the birthplace of the Slow City. Many people were interested in the concept of 'Slow City', wondering what a Slow City was really like, was it the slow life they imagined, etc. It was related to government promotion; our advertisements were even on CCTV Channel 1.

Q: But why did it decline later?

A: Probably because people felt it wasn't what they imagined. And what we imagined... I think should be... anyway, not this kind of life. People came here feeling, 'Your Slow City is like this?' and were very disappointed, seeming no different from other places. I think everyone defines 'Slow City' differently.

Q: From your perspective, what do you think?

A: For us, the slow city life I hope for... I really like Longshang over there. When you go there, you completely don't need to play with your phone. If you want to eat, you can go to the Auntie's Canteen; if you want tea, you go; they have a café.

> The guests we get here are like, 'Do you have karaoke here? Do you have a chess/card room?' What is the meaning of you coming to the Slow City? (The viewpoint expresses how tourists influence the overall environment, which is also an important factor in fostering the slow atmosphere.)

> And they have accommodation there; I visited. The person in charge told me... I asked, 'How come you don't have TVs?' He said, 'Why should our guests come here to watch TV?' He said guests come here precisely to...

> I remember someone from Taiwan in the homestay business said something with a similar philosophy. He said something like there's a kind of life called 'small town life' – that's how he put it – saying if a guest comes just to watch TV, then they shouldn't come here, it's meaningless. You can fully watch TV at home or on your phone. You come here to put down your phone, not watch TV, and experience real natural life.

> So, every Thursday, roughly from 7:00 PM to 9:00 PM, they show a movie in the courtyard, screening it for the guests. I think that's quite interesting.

Q: Do you think one reason might be the government, and another might be the tourists' own quality? They don't know what they need, coming here but still wanting to sing karaoke.

A: The environment here attracts that kind of clientele. There's no way to go high-end unless you upgrade the entire place, upgrade everything. But they spent 50 million upfront to get it to this state; it's impossible to spend that much again to upgrade further for you. And honestly, many of our farmers here... I see now the ones operating well, whether in catering or accommodation, are mostly run well by young people like me. Those in their 40s or 50s who just run things casually at home, they don't have the mindset to think about these issues much, they can't think of these problems. So, asking them to upgrade, spend money to renovate again, to match the government's ideal target, is impossible.

> First, they are unlikely to invest that much capital. Second, they aren't sure if there will be business. That's how it is.

Q: What cultural experiences do you think the current Slow City provides for tourists? Speaking of the current situation.

A: Recently, we were still talking with our Party Secretary. He still wants to dig deeper into these cultural elements. Including, isn't Gaochun tofu quite famous? He wants to develop some hands-on, parent-child type projects. For example, setting up a storefront to let more tourists experience it, to retain people. It's still under exploration, because they feel there are too few cultural elements. Including when we went to study places like Yuanjia Village in Xi'an, they are completely different from us. I think what they have is very good, but truly replicating their model here just doesn't work.

Q: They aren't slow tourism though.

A: When they were planning, their secretary was probably used very well, including their current operations. Because they have a profit-sharing model, completely different from us. I think there are bad aspects here. For example, selling chicken. Some people upfront just try to rip off whoever they can, make whatever profit they can. Actually, it shouldn't be like this. If you sell a fake old hen, you might make 100 RMB at that moment, thinking 'I earned so much today,' but actually, you are damaging our Slow City's reputation. Over time, people just won't come back. Feeling this place is... how should I put it... our people here are quite... a bit not very honest.

Q: Isn't the whole village surnamed Rui? Earlier I interviewed that family, the one below Fangfang's place. They said everyone here is related by blood or marriage, right?

A: You can go to Chunniu... Actually, they are relatively unique. Their boss, I worked with him last year on rural development matters.

Q: Now, interviews show different viewpoints. On one hand, some say no soliciting, customers go to whichever house they enter, and since everyone is relatives... But there are also voices, like what you mentioned, saying such situations exist. What's the story?

A: Let's just say when it comes to interest, who isn't selfish? It's hard to explain. If I, as a local, say this place isn't good, then it's my fault. I know this place has problems, I also want it to be better. We, as young people – I returned to start a business – I definitely want to be positive. Not to say I can improve the overall environment, but I'll start by fixing up my own place first, then gradually influence others. But there's a situation: if your own family does well, others won't necessarily say 'Your family is good, I should learn from you.' They will imitate you. But imitation is fine, if you can do it as well. But I feel it's a kind of rural psychology, hard for me to explain. It's quite difficult, actually, doing things in the countryside is quite hard, especially for educated returnees like us. If you do well, it seems like you should; if others don't do well, they look down on everything.

Q: Has interpersonal relationships weakened somewhat due to tourism development? Or is it still okay?

A: I can't say there's none at all, or that tourism made it very bad. I think it's just that everyone's starting point is different. Some people... because at the very beginning, our government was very supportive. You know, maybe when development started around 2010, how did the government support us here? At that time, it was the Agriculture Bureau, the Culture Bureau, and others – anyway, six bureaus from upper-level Gaochun. They selected 6 farmhouse inns, helped them renovate, and brought people to eat there. Because they selected 6 households initially – not randomly chosen, but call villagers, seeing who would operate them. At that time, no one was willing to be the first to try, take such risks. But after those 6 households were renovated, the next year was incredibly popular, completely packed. And because your business was good, other families gradually started renovating spontaneously, doing farmhouse inns, getting into accommodation. Then gradually, by around 2014, 2015, it reached a peak. Then, maybe due to promotional reasons, or word-of-mouth reasons, plus various other issues, it gradually led to the current situation. Slowly, there aren't as many people now. However, you came at the right time, these two days are also the peak season. By September, October, there will be more people. Here, the peak seasons are March, April, May, and September, October, November – these six months are peak season. Tourism definitely has high and low seasons. The climate is better then, yes. But I think this year, considering the pandemic, this summer was still okay compared to last year, even better.

Q: I want to ask, have you felt that even though it's a Slow City, perhaps because of the influx of tourists, your own rhythm hasn't slowed down but actually become faster?

A: I think, how to put it... the impact definitely exists. But you should say, for those truly engaged in business, like running a farmhouse inn, they certainly are that way, including hoping to be busy. But people like my grandparents aren't affected; they still wake up at 4 AM, sleep at 6 PM, carry on as before. Because our people here were originally like that. Before, without electricity, or without much to do, without many cultural/recreational activities, early to bed, early to rise, and they were physically healthier. Now there are more cultural/recreational activities because they aren't farming now, not working the land, so when idle, they have nothing to do. Not exactly, I feel they now... over at Wolun, dancing, playing, not sure what they do... We don't participate. It feels like life, entertainment activities are definitely richer than before.

Q: A couple of years ago, wasn't there a Hainan tourism company that entered and built things like Slow Amusement Park? What do you think of those? Do they conflict with the Slow City concept? Because it's about slow life rhythm, slow life quality, then suddenly introducing so many entertainment things.

A: Actually, it's okay. I think there are pros and cons. What are the pros? Many tourists come with children. If you just see scenery, children might not want to look at scenery; they have no concept of things like air quality, they just want to play. Good aspects: some people are willing to say, 'This place is quite fun, I can stay a couple more nights.' That's the good side. The bad side, the impact, is things like blocking roads. For example, if you want to go somewhere, you must take this route, or must buy their ticket. Then tourists complain about it being blocked off. Anyway, our villagers here also complained, but it had little effect. But I think it's still okay, I don't think it affects us much. On the contrary, developing tourism is better, now there are more tourists.

> Yes, because some people come and ask, 'What's fun here?' I need to have something to recommend. I can't just say 'Go see the tea plantation.' Elderly might be willing, but would children want to see a tea plantation, right?

> And also, some come specifically for, say, the rainbow slide, for the slide ticket. Such guests exist. So, there are pros and cons, depends on your perspective, different standpoints, different angles. Those people going there are mainly for the play items, they go there to play, places with projects... kids are like that. Otherwise, they don't have much else to do. Before, I remember coming here, I used to juststare blankly on Lv Family's lawn. Later, now everyone goes to the rainbow slide. However, on Lv Family's large lawn in spring, many people fly kites. Yes, during the rapeseed flower season, it's divided into two areas, the rapeseed flowers are quite plentiful. For our area, I think it's alright, I quite like it too. Even though it's developed, honestly, I'm willing to take my kid there to play. I feel quite comfortable there, pitching a tent, just sitting there is quite nice for me too. It's better than having nothing, right? You can't juststare blankly at our doorstep. It's quite useful. Our environment here is very good. But honestly, the Slow City company... it should be different now, I heard it changed, probably Slow City Group. Now the Water Slow City and our Mountain Slow City here are under one big company. The issue is the charging item are too high.

Q: Actually, I felt before that the connection between Dashan Village and the core Slow City area wasn't very deep. Because people coming to Dashan Village rarely get recommended to go there or anything. Like previously interviewing Dashan Village villagers, one even said they hoped to borrow a library here, but actually there are book sales over there.

A: There is. Actually, we might work on that next year, I heard from our [official]. They said... have you been to Lvjia? Before, it was the same model as us, even worse than us. But this year they completely upgraded. From the exterior walls, they started imitating Longshang's model. They also have a café inside, a book bar is under construction, the entire road has lighting now, including all the signs for the farmhouse inns – they look very comfortable,casual take a photo and it looks nice. I feel maybe next year we might... that's my hope, I don't know if it's true. Who knows if they'll keep their word.

Q: May I ask, as an operator, what is your hope for the future? Do you want more and more Slow City tourists, constantly increasing tourist numbers, or should it stop increasing after reaching a certain point?

A: Just speaking for accommodation, I think it should be... for this flow-based clientele, I don't hope it increases further. I hope for quality clients. Have you been to the third floor of our place? No, right? I've done a good job on the third floor; the rooms have distinctive features. When I started this, my family was quite opposed, saying there aren't many people, why still do this? But actually, after I did it,but some quality customers came. And having these quality customers, their overall quality, including conversation... anyway, you don't have to deal with them like flow customers. They won't say, 'Let me sit down and play cards.' What will they do? They'll sit down and have a pot of tea. Do you think tea-drinking customers and card-playing customers are from two different levels? Completely different.

> Not that I'm comparing card-playing customers... I'm just saying the two groups probably have different education levels, etc. It's not that I, drinking tea, have higher 'style', or that card players have worse quality. Not at all, everyone has different interests, right? But the feeling they give is just different. Including this table of mine. Before I fixed it up, if you look, lifting the cloth, it was just so-so. But after I put this cloth on, it's different.

Q: What was your major before?

A: You might not believe it – Veterinary medicine.

Q: And your insights into tourism... Did the government take you to various excellent villages, like Yuanjia Village?

A: No, it's like this: After being with my husband and having a child, I quit my job. Later I decided to return home. Fortunately, the family had these premises, so I renovated and started this business. After doing this, I couldn't just stay home running the business, relying on fate for customers, right?

> So I went out to do something. I started as a local guide/interpreter. Initially, I led regular tourist groups, but honestly, that was too tiring. Later, I switched to... because we work in rural tourism, there are often government inspection tours. For example, civil servants coming for a week of study, maybe visiting here for a day, needing such an interpreter. But such interpreters weren't available, because government inspection groups ask tricky questions. It's not like a regular local Slow City interpreter can handle it. They ask things like 'What's the per capita land here? How much dividend per mu?' You probably asked similar things, right? Who knows? Only farmers know, but which farmer would explain these to you, right?

> So I asked my family, and compiled notes on various tricky questions, gradually gained experience, and started doing it. Then gradually, perhaps because there's an association here, they asked me to join, compile materials, and slowly built it up this way.

Q: How is it done here? Each manages their own? No profit-sharing in business?

A: No. Initially, there were subsidies, but not anymore.

Q: Without government subsidies, profits certainly aren't as substantial as before, right?

A: Right. Business now... the ones you see still open are doing okay. Like Fangfang's place east of here, yes, and the two places west, they are slightly better. Here, I think... honestly, I don't think there's much. I don't really find our area particularly fun myself. I truly think it's not very interesting. But after going out and turning around, coming back, I still feel our place is good. Maybe it's just familiarity.

Q: Has the Wenfeng Pagoda always been here?

A: Since 2012. The pagoda itself always existed, but it was bombed and destroyed during the War of Resistance. Then in 2012, our government found experts knowledgeable in this field to research and restore it based on documents.

Q: Including the Heaven and Earth Stage?

A: The Heaven and Earth Stage has always been there. These things exist, but maybe they were just made to look nicer. Our Heaven and Earth Stage has a 3800-year history.

> Every year on the 17th, 18th, and 19th of the third lunar month, they invite local opera troupes from Anhui, like Huangmei opera or some Yu opera troupes, to perform for three, about four days. It's related to the local people's faith. Opposite the stage is a temple.

Q: It's held as scheduled every year?

A: Yes. Dashan Temple actually enshrines a Bodhisattva named Zhang Bo. It's said he came here to perform widespread good deeds, so the temple was built to commemorate him. It also protects the peace of this area.

> And about Wenfeng Pagoda originally: Gaochun has a Square Treasure Pagoda. Wenfeng Pagoda and the Square Treasure Pagoda are mother-and-child pagodas. That one is the mother, this one is the child. During the Ming and Qing dynasties, Gaochun was promoting literary fortune, so they built this pagoda to protect the area's peace and produce scholars. Indeed, some scholars emerged.

Q: And the Bronze Gong Well?

A: Behind us is Gaocun... Back during the War of Resistance Against Japan, Gaocun... because it's located on higher ground. During the war, our villagers here also showed wisdom and buried many Japanese soldiers. Why is it called Bronze Gong Well? They say if you throw a stone down, you hear a sound like a bronze gong.

Q: Was the Rui Clan Ancestral Hall originally in use?

A: Every year, around New Year or something, we invite... inside, it holds the entire Rui family genealogy. Also, every year, during New Year or sometime, they provide a meal for local elderly aged 60, 70... over 70 years old? A meal for them. The government also organizes some activities, for the elderly... it serves as the village's cultural activity center.
