# Supplementary material for: Rurality representation and changes in rural tourism destination
Source: PLoS One. 2026 Apr 21;21(4):e0347226. doi: 10.1371/journal.pone.0347226 (PMC13098982; doi:10.1371/journal.pone.0347226)
Supplement: S1 File — (ZIP) [file pone.0347226.s001.zip › supporting information/大山村漆桥村录音及转译文本/DS-JM 6.docx]

Basic Information:

(1) ID: DS06 (e.g., SA/DS/QQ-00)

(2) Gender: Female Age: 14 Occupation: Student

(3) Role: √ Resident □ Tourist

(4) Education Level: √ Junior high school and below □ Senior high school (including technical secondary school) □ College and Bachelor's degree □ Master's degree and above

(5) Years of residence in this locality: 14 Participation in tourism: No

(6) Annual household income: □ ≤10,000 √ 10,001~50,000 □ 50,001~100,000 □ >100,000

(7) Sources of household income (multiple choices): √ Farming □ Tourism-related service industry □ Others (e.g., migrant work, salaried employment)

(8) Tourist's Occupation (if applicable): □ Enterprise employee □ Professional (doctor, lawyer, teacher, etc.) □ Self-employed / Freelancer □ Student

Q: May I ask how many years you have lived here?

A: I've lived here since I was born.

Q: What changes do you think have occurred here before and after the establishment of the Slow City?

A: The houses have been built better, and the roads have been widened.

Q: After the development, could you talk about the feeling and experience of 'slowness'? What cultural experiences do you think are provided for tourists after the Slow City was established here?

A: Slowness means putting aside all matters, you don't have to be so fast; you need to experience life slowly.

Q: In terms of food, accommodation, transportation, sightseeing, shopping, and entertainment, what cultural settings are there to experience? You can talk about the pace of life, quality of life, living atmosphere. Do you feel this place has a certain cultural feeling that is different from other places?

A: I really don't know about this, it's hard to say.

Q: Have you been to other rural areas for tourism?

A: No.

Q: Suppose this area hadn't been developed yet, and you heard that a Slow City was going to be built here, what were your thoughts or visions about slow tourism and the Slow City? What kind of place did you think it should be?

A: It should be a quieter place, somewhat isolated, like a 'secluded paradise'.

Q: What do you think people should usually do? Things like farming, etc.? What should be prepared when tourists come?

A: I don't know.

Q: What discrepancies do you see between the current reality and your imagination? Where do you think the development falls short or isn't good?

A: No major discrepancies, it's quite good.

Q: What was the countryside like before? And what is the countryside like now?

A: The countryside in my memory was very simple and plain, the houses weren't so luxurious. Now there are many farmhouse inns.

Q: Do you think the current construction is too luxurious, or still relatively simple? Do you think it has gone too far, or is it just right?

A: I think the village should retain a more ancient look, that would be better. Now many places are very new.

Q: Do you think it has any impact on rural behavior? What impact has tourism had on rural behavior? For example, early to bed and early to rise, slow pace of life, clothing, food, housing, transportation, shopping, etc.

A: No impact.

Q: What about spiritual elements? For instance, because of tourism development, does your family feel more well-known, a sense of 'our home is really good'? Things like cultural confidence, identity, festive activities – you can talk about these. Or language aspects.

A: No, I don't really feel anything either.
