# Supplementary material for: Rurality representation and changes in rural tourism destination
Source: PLoS One. 2026 Apr 21;21(4):e0347226. doi: 10.1371/journal.pone.0347226 (PMC13098982; doi:10.1371/journal.pone.0347226)
Supplement: S1 File — (ZIP) [file pone.0347226.s001.zip › supporting information/大山村漆桥村录音及转译文本/DS-JM 7.docx]

JM: In the past, it wasn't as good as it is now in terms of development. Honestly, we used to work outside the village too.

Q: What was the village originally like?

JM: The village was still like this originally, but the front section was a bit better. The households further back weren't as developed; they focused on developing this front street. The areas behind haven't been developed much. The roads are just a bit cleaner, tidier, that's all. The whole front part is better. They haven't developed the entire village; it's mainly this front street that's developed. The households in the back continue their normal lives, working outside or as migrant laborers. Look, every household in our front row runs businesses, but the back row doesn't. They started with this street, focusing on farmhouse inns. Now, you see, you can't even drive cars to the houses in the back.

Q: Yes.

JM: The roads are quite small, narrow, there's only this one road. This road is pretty good though. The scenery includes mountains and water at the foot of the hills.

JM: Anyway, after the government development, it's slightly better for us. But if you're talking about the entire village being boosted, no, it's just this pair, this street. To be honest, speaking conscientiously, those of us at the front can benefit a bit locally, we can do some business. But how can those in the back do business? They can't.

Q: They could do accommodation, I saw some new ones built over there, taking people there to stay.

JM: I know. For accommodation, the government uniformly relocated a small village from below to over there. Originally, there was no foundation there, where would you get the land?

Q: The government is doing the right thing.

JM: It's the government's work, regarding supporting facilities. If you're talking about private houses, with only the existing ones, I'd like to build guest rooms too, but I can't build many. It's not cost-effective. Renovating small spaces isn't worthwhile. Their approach was: based on how much land your household had, after compensation, the area was entirely larger, all made into guest rooms. There aren't that many households involved anyway, only about twenty-some. Our entire village, you haven't seen it, has 280 households.

Q: Not just this street.

JM: The whole village. You only see the front part, you can't see the back, it's all along this main road. The whole village has about 280 households.

Q: Before tourism development, you still had land, I saw it, not much now, right?

JM: Not much now. Each household just grows a bit for their own consumption.

Q: Originally, you might have farmed, but now it's basically just growing some vegetables for yourselves, and you buy wheat, rice, etc., right?

JM: We still grow a little. Because there's wasteland in the hilly areas, and small vegetable gardens that don't produce enough to sell. If you mean that, yes, we have some. For example, the corn at my home, I grow it myself. We still retain a bit.

Q: Have you noticed any changes in the natural environment?

JM: The natural environment is okay, actually a bit better than before, to be honest. Cleaner and all, roads are neater, and there are people cleaning. It's better than before. Honestly, it wasn't this good originally.

JM: In the past, every household raised pigs or something, which actually felt less sanitary. Now they don't raise them anymore, to be honest.

Q: What about water quality, any changes?

JM: The water quality is quite good. Honestly, the tap water we drink is the same as in Nanjing.

Q: So, if rural tourism hadn't developed, might the water quality in this rural area have become worse?

JM: Actually, if it weren't developed, it might be even better. Fewer things would be washed in the ponds, fewer people might make it better, honestly, speaking truthfully. Because now, without much development, not many people in the Slow City farm. People our age are mostly working outside. Also, the government took back the farmland, planting trees where appropriate, doing other things. There's not much pollution anyway, no industry. The countryside has no industry. Yes, that's good. Nowadays... originally, the water in the ponds, well, almost twenty years ago I think people here drank from them.

JM: Around 2000, water was actually scarce, some villages weren't connected, so they drank this, yes.

Q: Now there's more pollution, detergent, etc., right?

JM: We wash things by the pond here. I'm not saying someone might pour a bit of household detergent into the pond, it probably happens. Here, every day people wash clothes, rinse rice, wash vegetables in it. Even if the Slow City wasn't developed, it would be like this, because country people are used to it, it's fine. Mainly, without industry, the water flows. If it doesn't rain or something, sometimes stagnant water goes into the fields, then new water comes. It's actually okay. More people coming might mean more pollution. Definitely, if tourists come, there's more garbage, more cleaners are needed.

JM: On holidays, to be honest, there are a lot more thrown away items like plastic bags, food wrappers. It's better managed now, they don't allow littering, there are people reminding others. For example, if you sell sugarcane or something, the seller must provide a bag, telling customers not to litter casually. The road workers have a hard job too, honestly, sweeping in the hot weather. Things like spitting sunflower seed shells...

Q: So cooperation from both sides makes it better. Have neighborly relations changed in recent years?

JM: Neighborly relations are pretty much the same. In the countryside, everyone knows each other, unlike in the city where doors are closed. For example, sometimes when our farmhouse inn is busy and we run out of a certain dish, we borrow from the neighbor, and they borrow from us too. It's pretty much the same as before. Is there competition? Not really, it depends on one's own capability. The customer chooses where to eat, where to spend. Also, the cooking is each family's skill. If you cook well, you can retain customers; if not, they won't come back. It's all based on individual merit.

Q: Has your lifestyle changed here? Originally, it might have been farming, now it's running a farmhouse inn. Is that right?

JM: For us, not much has changed. Because we've basically never worked the fields. The farming was done by my in-laws, the older generation, people in their sixties, nearly seventy. I don't know how to do that stuff. At most, I can grow some vegetables for myself. My husband also works a job outside.

Q: You run a farmhouse inn at home. Your appliances and such must be quite good now, fully equipped, right?

JM: Basically, conditions in the countryside are decent now. Almost every household has a house and a car.

Q: So, do you buy apartments in the city?

JM: Basically, everyone can now. I mean, maybe not in Beijing or something, but for example, in our local county town, we can afford to buy a house. The conditions are quite good.

Q: Have you thought about moving to the city?

JM: Not for now, to be honest. I've already bought a house in the town, but there's no need to live there. I can run my business at home. When the kids grow up, things can be flexible; the house can be sold if needed.

Q: So you actually prefer the lifestyle here? Staying in the city isn't appealing.

JM: But in the city I wouldn't have a job,反而 there would be pressure. At home, I can always earn a bit.

Q: Since the development here is for slow tourism, what cultural experiences do you think could be offered?

JM: Well, but the play items or activities here are all paid. I went to play today too. Yes, we have things like the rainbow slide, canopy drifting, jungle trekking, all that.

JM: It's over in the Lvjia area. I have a map. You can go, but it's paid. It's cheaper to book online. The whole package costs 158 RMB per person. If you just do one item, it's probably expensive because they include the sightseeing shuttle. You can drive your private car too.

JM: It's not in the village itself, it's in another area, like a forest place. I went today, it was quite exciting. Anyway, if you pay, they provide the full set, including raincoats. But regarding the Slow City... is it slow culture? What exactly defines slow culture? Our area here, including Gaochun Old Street, is considered part of it.

Q: Gaochun Old Street is also considered part of the Slow City. What do you think are its typical features? Regarding Gaochun Old Street or slow culture aspects.

JM: It's the slow pace. The countryside isn't as fast-paced as the city. Other rural tourism places also have a slow pace – coming to eat a meal, stay over. Yes, because the environment is good, no pollution, very quiet at night, unlike the noise in the city. If there's no business, places close early. By around 8 PM, basically the whole street is closed.

Q: So in the evening, you just watch TV, play with phones at home, and close by 8 PM? If there's business, you might stay open, but basically there's none, right? Because this whole front street is farmhouse inns, not primarily lodging. Usually, guests finish dinner and leave. Dinner typically ends before 8 PM. Unless occasionally guests stay late drinking, but that's not every day, maybe during holidays.

Q: Has there been any effort to revive rural culture? Have you noticed any?

Q: Including activities like... [Mentions 'Long March Hospital'? Likely a misheard name or specific local event/brand] ... are there unified organizers? For example, every year during the Spring Festival period on our street, each household has at least 4 or 5 tables [of guests]. Yes, bookings are made online, or you can reserve through me, I'll save you a spot and give you a number. Sometimes it might not be at our place, you might specify, then we reserve that number for you, like 'I'm number X'. We manage the flow.

Q: So actually, the government is helping you...

JM: The specific date varies each year, depending on holidays. If it rains or snows, they might change it. It's always in the second half of the year.

Q: I know, during winter and the New Year period.

JM: Generally, we only have a few peak seasons here. Spring for rapeseed flowers, autumn for maple leaves. Soon, around National Day (Oct 1st), it's for eating crabs, you can come and play. These are the better periods. Summer is relatively low season here.

JM: Summer is basically the low season. It's hot, and we don't have water play activities here. Also, there are things like the 'Jumping Wu Chang' performance, 'Big Horse Lantern' etc.

JM: Those activities are during the first lunar month. Not this year though, due to the pandemic, it's an exception. Normally, from the 1st to the 7th of the first lunar month, there are scheduled activities you can see. They happen around the village, like a village fair. Our village has always had these, organized spontaneously. For example, people from our village arrange 'Horse Lantern' performances from nearby areas or somewhere, scheduled for the early days of the month. There's also dancing, opera, or dragon lantern dances. It's for a specific period, not all day, just a few hours in the morning until it ends, then other programs are scheduled. There are organizers because such large activities need approval from higher up. You can't just do it randomly.

Q: Right, so actually the government is helping you together to create these folk festival activities, right? Including folk culture, dances, but they can only be seen at specific times. If we come normally, we might miss them.

JM: You simply wouldn't know. If you want to experience something like the 'Long Street Feast', you *must* come on that specific day. The atmosphere is gone the next day. It only lasts half a day. Then all the tables are cleared away. They can't leave them on the road because vehicles need to pass. They only block the road for half a day. Then they have police etc. blocking it, not letting other people or vehicles in, including our own cars – we can't drive in or out until after the event. If you come, you have to walk in.

JM: You can't drive through. It's literally tables from one end to the other, how could you drive? Yes, I've seen photos with many people, very lively, actually quite good.

Q: What do you think should be done if we're developing slow tourism here? Any suggestions for improvement?

JM: This isn't something we can decide. It's up to the government. And for any related project, you must get government approval; without it, you simply can't proceed. For example, if I had money and were an investor wanting to invest here and build something, without government permission, it's impossible.

Q: So, in your impression, what should slow tourism be like?

JM: In my impression, no matter where it is, there should at least be some play facilities or something. Like, including here, you can't have the main attractions too far away, people might be unwilling to go. Every place should have something to make tourists stay. But here, I estimate it's not enough for a full day. Or if you don't want to play, you can finish in minutes, just walk from one end to the other and leave. There's not much fun. When I travel, I also feel I can't stay here for long.

JM: I arrive, meaning I've been to this place, I take a walk, I don't feel like playing anymore, at most have a meal. The key is the experience of stopping and staying. They create a lively atmosphere when there are events, but you can't have large-scale activities every day. And if you did, the cost in facilities and personnel would be too high? Last year's Long Street Feast was really good though.

Q: Can I ask for your basic information? Your age?

JM: I'm 38.

Q: And your income range?

JM: I should be in the 50,000 to 100,000 RMB range. Yes, almost everyone is around 50,000 to 100,000.
