# Supplementary material for: Rurality representation and changes in rural tourism destination
Source: PLoS One. 2026 Apr 21;21(4):e0347226. doi: 10.1371/journal.pone.0347226 (PMC13098982; doi:10.1371/journal.pone.0347226)
Supplement: S1 File — (ZIP) [file pone.0347226.s001.zip › supporting information/大山村漆桥村录音及转译文本/DS-JM 8.docx]

Basic Information:

(1) ID: DS08 (e.g., SA/DS/QQ-00)

(2) Gender: Male Age: 57 Occupation: Slow City Staff

(3) Role: √ Resident □ Tourist √ Staff

(4) Education Level: √ Junior high school and below □ Senior high school (including technical secondary school) □ College and Bachelor's degree □ Master's degree and above

(5) Years of residence in this locality: 57 Participation in tourism: Yes

(6) Annual household income: □ ≤10,000 □ 10,001~50,000 √ 50,001~100,000 □ >100,000

(7) Sources of household income (multiple choices): □ Farming √ Tourism-related service industry □ Others (e.g., migrant work, salaried employment)

(8) Tourist's Occupation (if applicable): □ Enterprise employee □ Professional (doctor, lawyer, teacher, etc.) □ Self-employed / Freelancer □ Student

Q: May I ask if you are a resident, tourist, or staff member here?

A: I am staff here.

Q: How long have you worked here?

A: The company has been [established? operating?] for 17 years. (Note: The original Chinese "The company has 17 years" is slightly ambiguous, but this is a common way to express company tenure or existence. Alternatively, it could mean "I've been with the company for 17 years," which is more likely for an individual's response. However, the literal translation of the provided text is "Company has 17 years.")

Q: Could you share your experiences and feelings regarding the 'slowness' in the Slow City?

A: Life in the Slow City is very comfortable.

Q: What cultural experiences do you think the Slow City provides for tourists?

A: Slow culture means slowing down.

Q: Could you be more specific? For example, in terms of food, accommodation, transportation, sightseeing, shopping, and entertainment – eating, staying, playing...

A: Just farmhouse inns, eating and playing. Only the Dashan area has these.

Q: What do you think about some of the Slow City's iconic landscapes, like the snails, grass sculptures, rice straw sculptures, and also the folk culture, ecological culture? How is the experience of these cultural elements?

A: Not bad, all quite good.

Q: Can you describe the feeling in more detail?

A: For ordinary folks? Don't really have much feeling.

Q: Could you share your thoughts on the slow pace of life, quality of life, and living atmosphere?

A: I don't really understand this.

Q: How do you think the Slow City's promotional work is going?

A: The promotional work is done quite well.

Q: Where is it promoted?

A: Vegetable-selling residential areas, small stalls, at the entrance area... also the local Farmhouse Association here.

Q: Yes. How do you think the infrastructure and entertainment activities are developed?

A: Infrastructure... I tell you, it's all over there. There's not much here.

Q: Have you been to other rural tourism destinations? What differences do you see between the cultural development here and other tourism places?

A: Each place has different customs and habits.

Q: Right. What do you think are the strengths and shortcomings here?

A: The situation here isn't good, I tell you. Later... it should be said that in the last two years, no development has been noticed here. The construction is somewhat stagnant. Because there was construction in earlier years, but these last two years it's still very primitive, no development.

Q: What did you imagine the slow tourism cultural experience should be like? You can share your vision. What did you imagine slow tourism should be like, and now that it has turned out this way, what do you think can be improved?

A: There are many things that need improvement. Including the roads, and including the Wenfeng Pagoda, all need proper improvement.

Q: May I ask your age?

A: I was born in '66. (Implies age 57-58 depending on exact year, consistent with Basic Info)

Q: What is your education level?

A: Primary school.

Q: I have a few more questions.

A: I don't have time, I need to go. No time left, I need to go.
