# Supplementary material for: Rurality representation and changes in rural tourism destination
Source: PLoS One. 2026 Apr 21;21(4):e0347226. doi: 10.1371/journal.pone.0347226 (PMC13098982; doi:10.1371/journal.pone.0347226)
Supplement: S1 File — (ZIP) [file pone.0347226.s001.zip › supporting information/大山村漆桥村录音及转译文本/DS-JM 9.docx]

Basic Information:

(1) ID:DS09 (e.g., SA/DS/QQ-00)

(2) Gender: Male Age: 17 Occupation: Student

(3) Role: √ Resident □ Tourist

(4) Education Level: □ Junior high school and below √ Senior high school (including technical secondary school) □ College and Bachelor's degree □ Master's degree and above

(5) Years of residence in this locality: 17 Participation in tourism: Yes, family operates a farmhouse inn

(6) Annual household income: □ ≤10,000 □ 10,001~50,000 √ 50,001~100,000 □ >100,000

(7) Sources of household income (multiple choices): √ Farming √ Tourism-related service industry □ Others (e.g., migrant work, salaried employment)

(8) Tourist's Occupation (if applicable): □ Enterprise employee □ Professional (doctor, lawyer, teacher, etc.) □ Self-employed / Freelancer □ Student

Q: Are you a resident here?

A: Yes.

Q: How many years have you lived here?

A: I've been here since birth.

Q: What changes do you think have occurred here before and after the establishment of the Slow City?

A: The changes have been significant. It started around 2010. Before that, I tell you, this area was farmland.

Q: Could you share your feelings and experiences regarding 'slowness' after the development?

A: The visitor flow has obviously increased. It's much livelier than before. Because previously, hardly anyone knew about this place, and it was just like this. Also, the area in front was all farmland. The plants on those mountains were denser than now, and there were many more places you couldn't access compared to now.

Q: And have you noticed any difference in your personal feelings? Regarding the experience and feeling of 'slowness'.

A: There hasn't been a major change in that feeling. Because I've always been used to it, I can't really pinpoint any specific changes; it's already habitual.

Q: Do you think any cultural experiences are provided for tourists here?

A: Yes, the Agricultural Culture Park.

Q: Can you tell us about it? And in terms of food, accommodation, entertainment, etc.

A: For entertainment, we don't have much major entertainment here; it's mainly sightseeing, focused on scenic spots. For actual play activities, you need to go over there, at least to the Peach Blossom Fan Square and further south. In those wooded areas over there, there are dedicated recreational activities.

Q: How do you feel about the slow pace of life, slow quality of life, and slow living atmosphere?

A: Frankly, from a personal perspective, the Slow City means life is slower than in the city, allowing people to experience a kind of... like a spring warmth? Because with development these days, cities have become fast-paced, so the Slow City lets them slow down. Specifically, I think they've done a decent job in this aspect. Just the electric carts and the road designs already restrict you from driving fast. Also, the village roads are quite complex, interwoven. So specifically, it's indeed better than before, the conditions have improved a lot, because the main source of income here now relies on the Slow City.

Q: But have you been to other rural tourism destinations?

A: No.

Q: When you first heard the term 'slow tourism', what form did you imagine it should take? Feel free to envision it.

A: I think it should generally focus on walking.

Q: Do you see any discrepancies between the current development and your initial imagine? Are there any areas for improvement compared to your childhood?

A: It's quite good. Compared to the past, it's much better. The roads are much better. There are also more activities now than before, like the Golden Flower Festival which happens every few years, and 'Celebrating the New Year in the Slow City'.

Q: May I ask how old you are now?

A: I was born in 2004.

Q: What changes have occurred in the countryside? What was the countryside like in your memory, and what is it like now? You've already mentioned a lot, but see if there's anything to add.

A: I was born in 2004. The preliminary renovations started when I was about seven or eight, so my memories from that time are really not very clear.

Q: What elements do you think represented the countryside in the past? And what elements represent the countryside now?

A: I think they are identical.

Q: Before and after tourism development, have you noticed any impacts regarding material elements, behavioral elements, and spiritual elements?

A: Material elements: Before, people raised poultry, all free-range, let loose on the streets. Now they are kept concentrate in the back. This is better for the environment in many ways. Nothing else has changed, because these were all our own homestead lands before.

Q: What about behavior?

A: Because the pace of life is habitual, you know? Also, I'm not home for long periods because my school is fully close, so I'm mostly not home. To be honest, there are indeed quite a few old houses in the village, but they are concentrated in the back. The front ones are either newly built; basically, this entire front row is newly built, from my father's generation, so the houses are relatively new. Basically, the older ones are further back.

Q: Rural characteristics in 2005.

A: Okay, let's take this spot as an example. This is now a farmhouse inn.

Q: What elements do you think best represented rural characteristics around 2009? And now in 2020, what elements represent it? List about five or six for each.

A: In 2005, the most prominent feature when you arrived was that it was all farmland. From this area here, right? All the way over to that area was fields. Very narrow paths, impassable by car. Those field paths were only about half a meter wide. People used carrying poles for farm work. The paths often had holes dug in them, prone to collapsing. So the environment is much better now, definitely much better.

Q: Do you think water quality was better before, with less pollution? And now, even though there might be more pollution, there's also purification. Do you think water quality is the biggest component?

A: There's the Dashan Reservoir behind, and the ponds here. The water is starting to get a bit muddy now.

> The Dashan Reservoir behind... Frankly, before the Slow City renovations, we drank directly from it. Later, it switched to the tap water supplied by Gaochun. This is also proof that the water has been polluted.

Q: What elements do you think still represent rural characteristics now?

A: I'd say the biggest one is the trees. The forest coverage is still quite high. For example, that ecological road over there, I often pass by when cycling.

> Let me tell you, there used to be a very large bare patch of earth on Dashan mountain. I don't know how it formed, but it was there since I was born. Later it gradually disappeared, covered by forest.

> Also, Wenfeng Pagoda. Although it existed before, it was destroyed during the War of Resistance. It was rebuilt later, construction started in 2012. It was smashed during the war, so it was gone. Later, they built a two-story watchtower-like structure directly on its original foundation. The original site was a large cement area, with remnants of stone foundations, just the base remained. Later they rebuilt it, and it became what it is now.

Q: Was it rebuilt because of tourism?

A: Probably. It was built between 2012 and 2014, which was after it became a Slow City. They realized it should be restored, since it originally existed but was later gone.

> The Bronze Gong Well over there is relatively old. Near that tree, there's an ancient tree over 600 years old – deep cultural heritage.

> The deepest cultural heritage here is the Gaochun dialect. I learned it since childhood from my grandmother. You definitely can't understand the Gaochun dialect because it's the only Wu dialect in Jiangsu Province. If forced to categorize, it should belong with the Zhejiang dialects.

Q: So even with so many tourists, this dialect hasn't been lost?

A: Because the older generation always... They aren't fluent in Mandarin precisely because of the Gaochun dialect.

> Here, seniority within families is preserved. The hierarchy is like this, and my own generational rank is quite high.

> Also, every year... let me tell you, the temples over there, the village has a Earth God Temple. Every year we make offerings, kowtow, etc. First, we worship the Bodhisattva, then Guanyin, then Rulai Buddha, and so on, and also the Earth God Temple. We also invite the ancestors; we did that today.

Q: What is the ancestral hall used for? I took a look earlier; it seemed like it's become an office.

A: You might not believe it, but I'm telling you a very practical issue.

> Around 2009, it was used firstly as a barbershop, and secondly for processing rice. It hasn't been used for sacrifices for a long time. Sacrifices are done at home, inviting ancestors is done at home. Making offerings happens at the temple.

> Over 95% of this village has the surname Rui. This surname ranks beyond 100th among the Chinese surnames.

> Nearby villages mostly have a representative surname. For example, the Liu Family over there. Jintang, my mother's village, is mostly surnamed Chen.

> The surname Rui evolved from the surname Ji. If you look up its meaning, you basically won't find much; the first definition is 'a surname', then it means 'the appearance of plants sprouting and budding'. That's it, very limited.

> Almost everyone nearby is related by blood or marriage. For example, this is my house, right? That's my father's younger brother's wife's family house? That's my cousin's house. The house behind is my elder brother's wife's family house, call it elder brother's house. The house further back belongs to my grandfather's elder brother. I can name them all. And next to my grandfather's is my grandfather's eldest brother's house. They are all relatives.

Q: So you all run farmhouse inns; doesn't that create competition? Haven't you thought about specializing – your family does this, my family does that?

A: It doesn't matter. As long as everyone earns money, it's fine. Since we all know each other, sometimes if we run out of a dish, we borrow from each other. We cooperate; we're all relatives.

Q: In some places, tourism development leads to fierce competition and strained neighborly relations. No wonder it's so harmonious here; it feels different even during the interview.

A: Let me tell you, the Gaochun dialect sounds very similar to Japanese. When in Nanjing's Xinjiekou area, in shops, I've been mistaken for Japanese.

> The Gaochun dialect is ranked number one in difficulty to learn within Jiangsu Province. If you try to learn it specifically, you'll find you simply can't, because it's essentially a separate language, hardly related to Mandarin. Before development, everyone spoke Gaochun dialect. Now, with tourism, a 'Gao-Pu' dialect has emerged, Mandarin mixed with Gaochun dialect.

Q: So now, to protect rural culture, this must be directly passed down.

A: Cultural heritage, to some extent, it truly is passed down from before. And let me tell you, the Gaochun dialect still hasn't... as far as I know, I haven't seen any specialized, systematic books about the Gaochun dialect. But our teacher compiled one, he made a PPT and once specifically taught us those Gaochun dialect characters, right? They can actually be written in Chinese characters, but the pronunciation is completely different, just like Cantonese. You recognize the character but the reading is totally different, and you find it's not the character you thought it was. There are many you probably don't recognize. For example, 'meitou' is written, meaning 'girl' in Mandarin, but pronounced roughly like "me de ge". So it's quite nice sounding, even though the syllables change. I'm not sure, it should be similar to the ancient form, as it was directly passed down without evolving; its syllables are completely different from Mandarin.

> The pronunciation differs from one village to another here, though we can understand each other.

> The Gaochun dialect was used as a code during the War of Resistance Against Japan. Others really couldn't understand it. They had to find locals to act as translators for receiving messages. If intercepted, they couldn't decipher it. It was also used during the Vietnam War.

> Let me tell you, our roads here actually went through several stages: dirt, gravel, cement, and the brick road over there. That one is paved with cement. Now, this area here is my elder brother's wife's family house. If this were 10 years ago, you'd be standing in a pond. This whole row was trees, then here was dirt. There was a natural bridge, like a tree root about this wide, connecting two patches of land. You can't imagine how bad the roads were, completely incomparable to now. Going home felt like crossing mountains and ridges.

Q: Your chickens here look very strong.

A: Raised by ourselves. Some families let them out in the evening. They roam freely and return on their own. Sometimes it's troublesome for drivers because these chickens are a bit stupid; they don't turn, don't avoid cars. They see a car and charge straight at it because they don't know how to reverse. One careless moment and you have an accident.

> The trees here have also gone through several stages. What you see now are camphor trees, right? Also plum trees, jujube trees. There used to be many birds, now the most common are cats and dogs.

Q: I see over there, a persimmon tree has a sign saying 'WeChat pay, 2 RMB each'.

A: The jujube trees here... you just pull a branch, shake it, the fruit falls, and you pick it up to eat. It's all for personal consumption. Look, this jujube tree belongs to their house; the house was built around it because it grew like this. They integrated the ecology into building the houses. Let me tell you, the orange tree at our house is small now, right?

> Because it froze to death a few years ago and regrew. You have no idea how big it was before.

> It was quite love dearly. Nothing could be done; that winter was really cold, and we didn't paint the trunk white.

> There was a small orange grove here planned to become a lawn. The village notified everyone: take them if you want, they were all uprooted. If you needed them, take them yourself. Our family took one.

Q: How was this specific road chosen for farmhouse inns, while the inner areas weren't?

A: Because this road was the largest and the only one in the village that directly connected the two village entrances.

Q: Do people from the inner areas cooperate well with those on the outside? For instance, eating at an outer restaurant but staying at an inner homestay?

A: Yes, that's how it is. Also, many families own more than one house, only those who moved away...

Q: Are there many outsiders here, buying houses to run farmhouse inns themselves?

A: No, these are all our own homestead lands.

Q: In a previous interview, someone mentioned that those who used to work outside have now returned.

A: My father originally worked in Nanjing, now he works nearby and sometimes helps out. It's good. Tourism has brought convenience. The main source of income for developed areas relies on the service industry.

Q: The mountainous area – do you think it was representative in 2005?

A: To be honest, you could climb Dashan mountain wherever you could find a way, not to mention the two newly built stone step paths, right?

> Actually, there was originally a road allowing you to drive up the mountain. You still can. It's a gravel road. Extremely hidden, it's a gravel path concealed on the mountain, possibly even covered by bushes now – we might not even find it. There's parking space near the temple.

Q: Do you think water was representative in 2005?

A: Yes, there were two ponds.

Q: Did it represent the countryside?

A: From washing vegetables and rice, right? To washing hair, even cleaning trash bins – all done in the same pond. You might not believe it, but it's true. Because it's flowing water, not chemical pollution. They raise fish in it. As long as it's not chemical pollution, washing things there is okay, fine. But younger people like us, after washing some things in the pond, often rinse them again at home. Before tap water, farmers relied entirely on pond water. For drinking water, they went further, to the reservoir – larger, cleaner. The Dashan Reservoir. There are five fish ponds here. There were fields near the ponds, but the Dashan Company took them over, collecting rent annually. There are still vegetable plots here.

Q: Do you think the rural lifestyle was representative of the countryside in 2005?

A: That period, around 2005, right? The lifestyle then... just pick any undeveloped, typical Chinese village, it was like that, very universal. The food was the same, nothing particularly local. The dishes now are also grown locally in rural homes. City guests often ask me for recommendations, but there's really nothing special to recommend. I've eaten this way since childhood, can't point out any specific item.

> Also, recommending entertainment... I don't think there's much fun either, just trees, mountains, water. Not much different.

Q: What about rural folk customs in 2005, like the Spring Festival Long Street Feast?

A: On the 17th, 18th, and 19th of the third lunar month, there were opera troupes.

> The troupes came to perform opera – Huangmei opera, Yue opera, etc. There's a Heaven and Earth Stage inside the temple compound, that's where they performed.

> It only lasts three days each year. Because of the pandemic this year, it wasn't held; otherwise, it wouldn't be cancelled. Did you notice the red circles marked over there with '2020'? If you look at the planned stall locations for the tourism boost, you'd see numbered squares drawn. That was for assigning stall positions this year, but due to the pandemic, it wasn't done, so the grass there hasn't been weeded. Normally, after the event each year, it wouldn't be so yellow. There might be performances next year.

Q: Were there many festive activities around 2005?

A: The Lunar New Year's Eve counts. Inviting the Bodhisattva and ancestors during New Year, we still do that. We invite ancestors every July too.

Q: I know in Suzhou they have something called 'Carrying the Bodhisattva', where they parade the statue.

A: We have 'Procession of the Bodhisattva' here too, but not in this specific village, other villages do. Their Bodhisattvas even come here. There are Major Bodhisattvas and Minor Bodhisattvas. Ours here is the largest, so it doesn't get carried. The 'boss' doesn't need that like the smaller ones.

> The Dashan Temple here is relatively large in this area. But historically, it can't compare to Liucun Village, which has over 600 years of history. Slightly older than here.

Q: But that place isn't developed for tourism.

A: Right, not there. But that place has an important site called the Zhang Xun Memorial Hall; this is his hometown.

Q: Okay. Do you think farmland and such things in 2020 still represent the countryside?

A: Let me tell you where the tea plants are – over there.

> There are tea plants there. We also have tea gardens, south of the Dashan Heaven and Earth Stage.

> There are some over there too, on a rocky patch of land.

Q: Is tea a local industry, or just for personal consumption?

A: Mainly for personal consumption, but if guests want to buy, we sell.

Q: What kind of tea is here?

A: Green tea. There's also a tea factory here, yes, the Slow City Tea Factory. If you go over towards the Peach Blossom Fan Square area, you can see a very large one. Liujia is divided into Upper Liujia and Lower Liujia. Upper Liujia is directly east. Lower Liujia is south. You can see very large tea plantations, really vast, and several of them. A nearby tea plantation was also the filming location for the tea factory in the drama "In the Name of the People".

Q: Do you think forest land can represent rural elements now? Forest land, meaning trees and such.

A: Regarding trees, just look around and you'll understand. The tree coverage here is quite high. They've done a good job with virescence. Let me tell you something interesting: the ecological road, right? Try cycling there at the hottest time of day. On a normal road, it's quite cool, but once you enter the ecological road, you'll know what cold is. It feels at least several degrees cooler than outside, like an air-conditioned room. The temperature is really low. A great place to escape the heat. There's even a bamboo sea over there.

> The verbena here looks like lavender. There's also a peony garden. It has everything you'd expect.

> Look, there are loofahs hanging from that bamboo, climbing up. The main plant is in the canopy. When the loofah ages and dries, the skin becomes brittle, and it can be used as a scrubber for pots.

> Let's talk about the Bronze Gong Well. It has a thick, round wooden board covering the opening. But before, there was a small gap; we threw stones in. It's quite deep.

Q: Are there other entertainment activities in the Slow City?

A: I went today. Cost over 300 RMB. Newly built, letting locals experience first, give feedback, do some promotion.

> How to describe it... It's a high-altitude thing, right? You know those be driven by the current boats that go down a water channel? It has turns, quite fun. What's different? First, it's set within the forest – how do you slide through the forest? Second, the slide path is made of fully tempered glass, transparent. It's cool, you can see the scenery.

> There's the Shijiu Lake Bridge nearby. You go to Gaochun, take Metro Line S9 towards Nanjing, in the direction of Nanjing Jiangning Airport. It's huge, visible on city-level maps. If you go in the morning, it's foggy, visibility less than 10 meters. Also, in the middle of the lake, there are small hump like little islands, each only about seven or eight square meters. I don't know their purpose.

> Maybe something grows there? Right. Like my grandfather, he has 5 fish ponds. In recent years, he doesn't really raise fish actively anymore; if there are fish, they just stay. The main focus is on river crabs – a pillar industry here. Grandpa also has a watermelon field. They grow corn over there. Two or three years ago, there were mulberries and wild strawberries around. These are millet dates.
