# Supplementary material for: Rurality representation and changes in rural tourism destination
Source: PLoS One. 2026 Apr 21;21(4):e0347226. doi: 10.1371/journal.pone.0347226 (PMC13098982; doi:10.1371/journal.pone.0347226)
Supplement: S1 File — (ZIP) [file pone.0347226.s001.zip › supporting information/大山村漆桥村录音及转译文本/DS-YK 1 .docx]

Basic Information:

(1) ID: DS13

(2) Gender: Male Age: 35 Occupation: Live Streamer for Tencent's Official Video Platform

(3) Role: □ Resident ☑ Tourist

(4) Education Level: □ Junior high school or below □ High school (incl. technical secondary school) ☑ College & Undergraduate □ Master's degree or above

(5) Years lived locally: Involved in tourism:

(6) Annual Family Income: □ ≤ ¥10,000 □ ¥10,001–¥50,000 □ ¥50,001–¥100,000 ☑ ¥100,000

(7) Resident Income Source (multiple choices): □ Farming □ Tourism-related services □ Other (Migrant work, Salaried job)

(8) Tourist Occupation: □ Company Employee □ Professional (Doctor, Lawyer, Teacher, etc.) ☑ Self-employed/Freelancer ☑ Student

Q: Do you feel any difference between this visit and your last one?

A: Yes, it's different. Last time, this place was unfamiliar; I found it by searching online. I was searching for villages around Nanjing and came across this Dashan Village. Last time, I came specifically to do a live stream, targeting something like a market. But when I arrived last time, I had stayed overnight in Shiqiangwei. The next morning, they said there was a market here, so I came over early at 5:00 AM. I wandered around from 5:00 until almost 9:00 AM, and I didn't see a single person, you know? It wasn't as described. So, I just started filming the beautiful scenery here, and then went to nearby places like Dashan Temple, the Heaven and Earth Stage, Wenfeng Pagoda. I looked around everywhere, including spots with activities like CS or canopy drifting. I basically circled the entire area. After seeing it, I felt it was pretty good. This time, I'm also preparing content for Tencent's official platform. As a professional online influencer and live streamer, I need to find a rural wood-burning stove here to cook rustic dishes. Last time I came, I saw some people doing farm work, like picking peanuts. I'd never seen it before, it felt very novel; you don't see this in the city. So I went over, watched, chatted with them, and got a rough idea of their family situation. But at that time, I didn't know they had a wood-burning stove, so I left. Yesterday, when I came back, I happened to see him chatting with someone. After they finished, I went up and asked. I thought if he didn't have one, I'd go to a place further ahead. Last time I came, I saw an elderly lady carrying a lot of firewood. If someone is carrying firewood, they must have this kind of wood-burning stove at home, right?

So, this was my first stop, to come ask and look first.

Then I asked, and after looking at his house, he said there's a small one, not very big, which fits my requirements.

I said, great, but do you also have a chicken coop?

Because I need to catch a chicken. I need the whole scene. He said yes. I said that's perfect. I said I could do everything here – eating, staying, cooking the whole set.

So, I've been working here from the day before yesterday until today.

Listening to you, it seems you've been here from a couple days ago until now. So in the future, if there are rural activities, I might choose this place again, since I'm quite familiar with it now. The most important point is, ideally, towards the end of the year, near Chinese New Year, they have a Changjieyan (Long Street Banquet) here. That Long Street Banquet is a massive scene. You could say many media outlets from the entire TV station come to do aerial shots and report on it. So, I'll see when I come next time. That would be very good. Have you seen it? Wait until the end of the year.

Q: So, regarding Slow City – you must have read about it online – it's defined as a Slow City. What is your understanding and definition of a Slow City? Do you feel it meets the Slow City standards?

A: I think, as the first Slow City in China, emphasizing slow pace, slow living... this kind of slow-paced life, I think

completely belongs to a type where you don't have the life pressures you find in the city. The pressures there are immense, but coming here, you can basically not think about those too much. You can immerse yourself in this environment, it's very relaxing, very comfortable, very relaxing. Especially such a tranquil life. I feel this completely pastoral life is something many city dwellers yearn for; it's basically impossible to find in the city now. Basically, people here... when I came last time, they said on Saturdays and Sundays this place definitely has visitors. Basically, many people from Nanjing come, as it's not far from Nanjing, so it's convenient to get here.

On weekdays, they are at work, then come here on Saturdays and Sundays.

Q: Do you feel this place offers you any cultural experiences? The wood-burning stove you mentioned counts, right? This kind of wood-burning stove, and also because there are some...

A: The first time I came, I stayed at Chun Niu's place at the head of the village, probably the first household. For filming needs back then, I started from the first house and walked past every single one, though I didn't necessarily chat with each or look very carefully. Sometimes I'd watch them early in the morning, observing rural life in the village. I saw people at the pond washing clothes by beating them with a big paddle. I said, this is something I've only seen in period dramas before, not in real life, but here it's real. I found it quite novel, so I ran over and asked, "Don't you use laundry detergent?" I assumed you must use it, so I asked, "What's the process if you use laundry detergent?"

He said they first scrub and wash the clothes at home, then go to the pond to beat them, rinse them clean after beating, and then go back home to wash them again.

It's like that. I also asked about washing vegetables, meat, and even machines, all in the pond?

Yes. And I said, "You wash those there too? And wash them again after bringing them back?"

But that day, at the pond further ahead, I saw an elderly lady and asked her. She said, "I don't wash here," referring to food items. I thought, "So some are more hygiene-conscious."

Later I learned that this pond has a specific person responsible for it. They have designated areas. For example, Dashan Village has six ponds in total. Each pond has a responsible person who ensures there are no floaters on the water and that the water quality meets certain standards. I learned all this in detail before. When you come to the countryside, you should understand these things, the stories behind the ponds.

Also, last time, I originally planned to film a segment for a live stream about a woman here and her "vegetable basket story" – her journey from starting a business to success. But last time, because I was live-streaming that day and she had a meeting in the evening, we couldn't film it, which was a pity. Her story involved a vegetable basket containing many products, like various sauces, and how she step by step, through WeChat moments and online promotion, gradually started selling the rural pastoral life of Dashan Village, including its agricultural products. I never got to film that. Are there other tourism products here? Branded tourism products? There are some local specialties. For example, there's a local chicken, a kind of 'wolf chicken' soup? Almost every tourist who comes here tries it. Their chickens are quite large, over a hundred yuan each.

Q: In your opinion, what improvements does this place need to develop better?

A: One thing might be transportation.

Ideally... when I came this time, I took the metro. Especially yesterday, I arrived around 5:00 PM. From the Gaochun station – the metro station – to this place, there was no bus, no public transport. So I had to take a taxi. One characteristic of taxis here is that they don't just charge the fixed price shown on the app (like DiDi); they add an extra fee on top. That day, I argued with the driver for a long time. I said, "I take taxis all day in Nanjing, long distances, and this is the first time I've encountered this." He said, "In our Gaochun, it's the rule." Then I immediately contacted someone from the local Gaochun government and asked them. They told me indeed there is such an 'unwritten rule'. Right. But for his trip, he might add 20 yuan. I asked how much extra, could it be 20? I said, "That's not much, but psychologically, I've never encountered this before. Why not just include it in the platform price, be clear and upfront? Don't add it separately." It made me feel uncertain, you know? It felt somewhat deceptive. That feeling wasn't very comfortable. Finally, through the government contact, I learned that this exists. They explained why: Dashan Village is located on the border between Nanjing and Anhui. When they return, they have no passengers, no customers taking taxis back, resulting in an empty return trip. So, for them, they feel they earn less this way.

My fare here was about 64-something yuan, less than 65. Think about it, if you subtract 20, he gets just over 40, and then the platform takes a cut, so he really doesn't make much. Right? That's the calculation. So when it was time to pay, I asked, "How should I pay you the 20 yuan?" I had recorded everything the government person said. On the way, the driver didn't seem happy or comfortable either; it was probably his first time facing this situation, just like it was mine. In the end, I said, "How much in total? I'll pay you."

He said, "Forget it, no need now. Consider it settled, I won't take it." So I didn't give it to him.

I figured all this out at the Slow City place. So, at the Slow City Visitor Center bus stop, there's a direct bus to Shuangpaishi. Right, Shuangpaishi Station. From Shuangpaishi, you can transfer there. To get to the Gaochun metro station, you can take the S9 line from there, which goes directly back to Nanjing South Station. There are also buses at Shuangpaishi.

I didn't take the bus last time; I took the metro directly. I thought the metro would be faster, so I took it. The best part of the journey last time was when I went back, I filmed the "Sky Mirror" at Shijiu Lake, because I travel around a lot.

Q: So you are a travel blogger, a travel influencer.

A: These are the original clips, unedited, no color grading, nothing added, you know?

Q: What differences do you see between this place and other rural tourism destinations in terms of cultural construction? Other places...

A: The architecture here... it feels somewhat scattered, lacking that kind of Ming-Qing ancient architectural cluster, ancient village feel. Because yesterday I was chatting with my homestay host, and he said this area was built later. I've heard of Nanjing's "Five Golden Flowers" before, but I haven't been to those places. Where have I been before? In Anhui. Jing County in Anhui. Including Yi County, I've been to those places. I was working on a project in Jing County before, so I visited all the surrounding scenic spots.

In the mornings, we developed the market, I took many sales reps to develop business, and in the afternoons, I took the driver and the sales reps to play in the scenic areas.

So, places like Zhaji, which has ancient architectural groups from the Yuan, Ming, and Qing dynasties,

spanning a long period. The preserved, authentic Hui-style architecture there, with houses dating back 700 years... or replicas from that era... gave a feeling of "small bridge, flowing water, homes" – that ancient village atmosphere was excellent, very authentic. Here, I feel it lacks that a bit. But each place has its own characteristics. This place, you could say, is somewhat lacking culturally. But if this place were to build a large number of those Hui-style buildings, creating that kind of imitation ancient village, do you think that would be good or bad? As a supplement? I think building a lot of that later, if you deliberately change the environmental structure to create it, wouldn't work. I prefer the original ecology, something that hasn't been disrupted. For example, take the forested mountains here – you might not be aware – the mountain opposite is Xiao Shan (Small Mountain), and over there is Da Shan (Big Mountain). In Xiao Shan, the village now strictly forbids anyone from entering the forest. Do you know why? Because there are wild boars and venomous snakes in the mountains?

Right in this mountain here. Especially people coming from Nanjing, many don't know. I only learned recently, from my two visits, that entering is prohibited. And there have been incidents here where people were injured, attacked by wild boars.

Wild boars are very powerful, almost like bears. There's another thing I still can't confirm whether it's true or not.

The first time I came, I used navigation and started from the Taicun Village Committee. I had to cross the Da Shan forest, past the Da Shan reservoir, to get here. It was towards dusk, the sun was setting, but I had to hurry through the forest before dark. It was my first time, I didn't know the situation of the mountain. I had my gimbal with me, ready for live streaming, thinking if there was an emergency, people in the live stream could call for help.

But considering something, I just held my phone, didn't mount the gimbal, and carried a stick, just in case. Later, the next day, I learned from an elderly lady at the pond. She told me, "There are wolves on the opposite mountain." So, I was walking very fast. I reached the national highway just before it got completely dark, around 7:00 PM. I thought if I were any later... I didn't know what was in there that day. Coming from that side is not feasible; it's about 5-6 km. For someone like me, often outdoors, my fitness is quite good. So, 5-6 km is nothing, I walk fast.

To this day, I'm still not clear about the details of that injury incident, or if there really are wolves. Some say yes, some say no, I'm not sure. But the presence of wild boars here is confirmed; they are definitely here. Don't they come down? Won't they come out together? They generally don't approach areas with people. Right. In the past, there were incidents with elderly people here,

but not now.

That time in the mountains, there was no one before or behind me, absolutely no one. It felt very isolated, completely silent, no one. Just me. On the road, I encountered three dogs, household dogs. You know, village dogs can be fierce. Yes, they bark at strangers.

The second time I walked that route, a dog was right in front of me, barking. I just ignored it because I know how to deal with dogs. Don't run, don't stare directly at them. If you lock eyes, they feel threatened and might charge and attack, you know? So, just ignore them, keep walking your path. That way, they relax their guard. It's about experience.

Q: When you first heard the concept of "Slow City" and "slow tourism," what was your expectation? What did you imagine this place should be like?

A: I imagined it would be very relaxing, completely pressure-free. Everything here would be slow-paced and pristine. That's what I was looking for, I think.

Q: What discrepancies do you see between that expectation and reality here?

A: The discrepancies I feel are, first, the distance seems... the agritourism spots are quite close together.

Second, the scenic spots here are few, not only that, but some are also too far apart. For instance, there's a place called Peach Blossom Fan that I still haven't been to. Peach Blossom Fan is just a large square, a lawn. Why haven't I gone? That day, I came down from the Heaven and Earth Stage. From there to the Peach Blossom Fan square is 7.5 km. Let's say 7 km. Round trip would be 14 km. I decided not to go. It's just too far. That day I had already walked quite a bit. Imagine, I walked around here, then went back to Wenfeng Pagoda – I walked the entire way to the top and back down, then to Dashan Temple, then to the Heaven and Earth Stage. To walk another 7-8 km, over 10 km more? I figured, forget it, I'm not going. And I heard it's disappointing anyway.

Q: Right. Do you feel any changes or differences between your first visit and this second one?

A: No changes, there haven't been any changes. I think, for me, because I need to find specific things for my live streams, like this wood-burning stove,

I need it to cook food, and I need to catch a chicken myself in the fields, live-streaming the whole process for my fans, showing them this pastoral scenery. It feels great. That's for them to see. But my personal feeling compared to the first time isn't much different, precisely because nothing has changed much. Also, one feeling about this place is that... it's not like in the city where shops open for business regardless. Here, if there's no business, they just close up. You don't know where anyone is. Yesterday when I came, it was almost evening. I started from the first house, Chun Niu's place. Along the way, only two households, Xianglixiangqin and another one, those two were open. I wondered, where are all the others? Are they not operating or something? I was really surprised, you know? Then I got here and was told they had all gone out. Where to? To the Slow City center to do square dancing.

Q: To the Slow City town?

A: No, to the Dashan Slow Visitor Center. They went there for square dancing.

Q: What is the rural village like in your memory? What was your impression of villages from childhood? What are villages like now? Are they different?

A: How should I put it? I didn't spend my childhood in a village; I have no such memory. Probably our parents' generation might have lived in villages, but for my generation, we don't have that memory. Sometimes we see it on TV. But actually going down to villages myself... the earliest was probably when I went to Anhui. When was that, roughly? That was... around 2008, I think, about 12 years ago. How does a specific village feel from 2008 until now? I suspect those ancient villages there probably haven't changed much. They've been preserved since the Yuan, Ming, and Qing dynasties for so long; they won't have major changes.

Q: What differences do you feel between those ancient villages and this place?

A: Over there, it's all Hui-style architecture, with small bridges and flowing water, and the scenery...

In Jing County, for example, it's surrounded by mountains on three sides, with one road leading directly to Wuhu and then to Nanjing. So, when you're in Jing County, no matter which direction you go, it's all scenic. Even the rocks and mountains along the road are scenic. There's much more to see; the whole journey is scenic, regardless of direction. I particularly remember the scenery to the south was very beautiful. I also went to Taiping Lake. Taiping Lake, part of Anhui's "Two Mountains One Lake" – Huangshan, Jiuhuashan, and Taiping Lake. When we went to Taiping Lake last time, I had a sales rep under me who knew someone there, an acquaintance who operated boats. So, a group of us went, and no one paid. Normally, entry would cost 400 RMB. Yes, 400 RMB. Sailing from the outside to the inside takes about an hour. The ticket covers several islands, including a Huangjin Island (Golden Island) inside.

Q: What do you think are the most representative elements of a rural village? What comes to mind when you think of a village now?

A: The elements of a village... I think first, the architecture should have characteristics, like ancient villages, with antique charm, not something built recently. Like those well-preserved ancient villages from the Yuan, Ming, and Qing eras. Also, there's a scene on Douyin (TikTok) – someone leading a cow, a farmer leading a cow across a small bridge, with thatched cottages nearby. I feel that kind of scene is basically unseen here now.

Q: What impact do you think the influx of transportation, information, capital, and tourism has brought to rural villages?

A: It definitely leads to different development. More tourists come. If developed well, it attracts more people. But here, sometimes shops are open, sometimes not. Last time I wanted to buy instant noodles, the person wasn't even there. The local customs here are very honest and simple. Households leave their doors open without worrying about theft. Yes, that left a deep impression on me last time – this place isn't afraid of thieves. Even the small shop selling instant noodles, alcohol, etc., the shopkeeper wasn't there, the door was wide open. I waited a long time, no one came. Finally, I found someone else who called the shopkeeper over. People are very trusting here. You couldn't do that elsewhere; things would get stolen immediately. Some places here have surveillance cameras installed, but some households don't bother.

It's because many people in Dashan Village share the surname Rui. They built a Rui Family Ancestral Hall to compile family records, and everyone is expected to behave according to certain rules, guided by this ancestral hall. So they are all relatives, less afraid [of theft], doors are left open, and they even borrow vegetables from each other. Like this family here, their relatives are behind, and over there are also relatives – all closely related, real aunts.

Q: What material changes has tourism brought to the village? Things like rice paddies, vegetable gardens... or you might have covered this. And behaviors? Any changes in the behavior of villagers or tourists?

A: After rural tourism development here... how to put it? Regarding the villagers, their customs still seem very honest and simple. Why is that? Your meaning is... actually, despite tourism development, it might have become somewhat materialistic, but the folkways remain pure? Unlike other places where tourism led to different commercial behaviors? So perhaps this is one reason attracting you to return? Probably partly because of the exceptional honesty. I think the people here are very good. It's not like once commercial elements are introduced, fraudulent practices emerge, damaging the original ecology. I don't really like that. I remember during my first visit, sitting at Chun Niu's place, a couple was there (I think, I wasn't speaking Nanjing dialect, I used Mandarin, so they didn't know where I was from, just a tourist). I was eating inside, and they were basically arguing over the price of a room. I felt that wasn't very good, especially in a place like this, where everything is so quiet, and all you hear is arguing over price. Either stay or leave, but don't disrupt the original ambiance. It felt very out of place. I found that quite unpleasant. So, some tourists have high quality, yes.

Q: What different feelings do you have between the culture of Southern Anhui (Wan Nan) and the culture here? In terms of rural cultural experience.

A: How to say... I still prefer the Hui-style culture, probably. This might be due to personal reasons; I'm a photographer myself, so I have a preference for these things. The way we photographers see and capture images is different from non-professionals. The beauty we find and the angles we appreciate differ greatly. The difference is significant. So, regarding Hui culture... it's hard for me to explain simply. It's difficult to describe Hui culture in a few words. Because it involves... there were many scholars and artists in Anhui. Here, I feel... perhaps my understanding of this place isn't deep enough, as my time here has been short. So, discussing deeper cultural aspects, I truly can't articulate it clearly.
