# Supplementary material for: Rurality representation and changes in rural tourism destination
Source: PLoS One. 2026 Apr 21;21(4):e0347226. doi: 10.1371/journal.pone.0347226 (PMC13098982; doi:10.1371/journal.pone.0347226)
Supplement: S1 File — (ZIP) [file pone.0347226.s001.zip › supporting information/大山村漆桥村录音及转译文本/QQ-JM 1.docx]

Q: What are your thoughts on developing the "intangible" aspects? Since they are intangible, how do you actually make them concrete and substantial?

A: Talking about how to do it... no one has a complete, all-encompassing understanding. But the first step, if I were doing it, would be to convene extensive forums to listen to the opinions of the general public. Based on that, I would figure out how to make it vibrant and successful. This is what the government *should* do, but unfortunately, the government hasn't done it.

Second, how do I do it well? From my personal perspective, first of all, this "Slow City"... I'd say it possesses neither remarkable mountains nor water features, and even less historical depth. It lacks all three. So what does it have? How do you promote "slow culture," a "slow pace"? You must remember to identify and build upon your unique local characteristics, things others don't have. These local characteristics are valuable and have development potential. Coordinating with tourism, consider aspects like food culture. What do tourists do when they come? They look, they gain knowledge, they experience food culture, they sample local cuisine.

Third, they buy some souvenirs. Currently, we lack in all these aspects. What souvenirs are there to buy? If we excavated the local culture... there are many local foods, but people are unwilling to develop them properly. Instead, they engage in short-sighted restaurant practices. I'm talking about cooking a little, preparing some dishes, some rice... if the going rate outside is 100, they charge 150. This has the opposite effect. It's not good; it creates a negative impression for outsiders.

Thirdly, this negative impact... but what is there for tourists to see here? Just a slightly better environment, a somewhat larger area. That's it. Other places have environments like this too. Look at Guilin, Yunnan, Guizhou, etc. – aren't they superior to this place? I'd say they surpass this Slow City by a hundredfold, or at least several dozen times. But you need to find your unique local cultural characteristics, things other places don't have. It seems they lack initiative. As I said before, for example, our traditional ways of production and life, traditional food preparation methods, etc., our traditional virtues, cultural morals... where are they embodied here for me to learn from? How, from the present, do I become a model of morality? Even if there's not much to see, if you come to me, I should make you feel your trip wasn't wasted. I should make you feel that the people here are truly good.

Q: So that visitors, and their children, can learn something and be inclined to return?

A: Right. Regardless, if they come directly, they should feel their children received a meaningful education, and they themselves gained some unique insights. They could even personally participate in productive labor. For instance, the process from grain processing to cloth making, or the entire process from a single grain of rice to a bowl of cooked rice – the four steps involved – or from a silkworm cocoon to a piece of cloth. These processes embody the wisdom of hardworking people.

Furthermore, for example, regarding food, or the moral norms among farmers, like respecting the elderly and cherishing the young during the Spring Festival... How are moral concepts instilled through ancestral hall culture? How is self-discipline practiced? None of this is being manifested or implemented. How can you develop without it? Of course, I worry they might say, "Who are you to talk?"

Q: They really wouldn't say that, Grandpa. You are...

A: They have never, for instance, the "Slow City" people have never once sought our opinion. Regardless, I've been involved in cultural relics and archaeology for over 40 years, and also in folk customs.

It's really unacceptable. There are many things here that are unacceptable. What is their mindset? They think that if I hold an official position, I'm smarter than hundreds of thousands of people, smarter than anyone anywhere. I'm omniscient. If I weren't omniscient and smarter, how could I hold such a high position? So, I'm smarter than you. That's their concept.

The second concept? I'm an official here, probably for just 3-5 years before I'm transferred. Why should I care about your well-being for the next generation? I only care about the immediate present. I'll "harvest the leeks" today and consume them immediately, without regard for the next step. Short-sighted! They lack a long-term development perspective.

Q: What do you think are the cultural characteristics of Gaochun, especially the Slow City area?

A: Many! For example, as I mentioned earlier, one must know oneself and know others. I need to understand where my true advantages lie. I may not have great mountains or waters, nor historical depth, but what do I have? I have simple, honest folk customs. I have excellent qualities like diligence, frugality, and hard work. These are my first local characteristics. Then there's the warmth towards guests, our local dialect, our series of local foods, including how they change with the four seasons, the cycle of grain cultivation, weather proverbs, how we observe the daily weather and natural environment...

There's actually a lot of cultural depth here, but it's not being brought out. Let me give a simple example. Suppose I walk somewhere, what provides me with personal and moral education?

Sadly, they don't have this. Let me give a very simple example. Suppose I walk there and see a naturally shaped stone with an inscription, for instance: "If it doesn't rain on July 7th, don't return home on August 8th." People might ask, what does this mean? I could have calligraphers write this phrase, different calligraphers creating different versions. Then I explain that this is a proverb farmers have accumulated over thousands of years based on weather observation. It means if it doesn't rain on the 7th day of the 7th lunar month – not even a little – then it will be a period of good weather and rain (or potentially drought, depending on interpretation). If it doesn't rain at all, it means drought in the middle of that month.

Then we change to another spot. For example: "East wind in spring, west wind in summer, take the horse and send provisions." First part, right? What does it mean? If an east wind blows in spring, and a west wind in summer, it's about to rain heavily. Then you should quickly prepare food and send it to those working outside. If we were to provide this kind of information, including everyday economic culture and proverbs, I could provide hundreds, even thousands of items. But they haven't manifested a single one! How to farm, the "Three Knows" in farming? What are they? How about proverbs about weather and their application in agriculture? In the Slow City, without mentioning too many, I could contribute 3,000 – 3,000 points, 3,000 ideas. For these phrases, I could invite famous Chinese calligraphers. You could start smaller, using different scripts on different stones. If I really can't manage that, even placing a large, old cracked jar or a vat at a home's entrance, with a phrase written on the outside... wherever you walk, at the doorstep, it should constantly reflect its cultural connotation. They haven't done this.

It's a great pity. After you go there, it's just agritourism, all agritourism.

Then, food culture. For example, what is the meaning of "cake" (Gao)? Under what circumstances is it used? After Spring Festival, when people marry, build houses (raise the roof beam), have children, move into a new house... in such situations, if there is "Gao" (cake, often homophonous with "high" or "prosperity"), it symbolizes good fortune for you. In the past, if a family directly offered "Gao," it signified prosperity.

Besides this, actually, many Chinese people find it difficult... every action has local cultural significance, I know. For example, the way I pour tea for you... Previously, when I was on that Gaochun TV program, the one about Gaochun history and folk culture – it was an excellent program – I talked about this.

For instance, I discussed tea culture. Gaochun's tea culture is very interesting. How do we embody tea culture? The Slow City has tea. You could create a series of stories around tea. For example, when I pour tea for you, I remember my grandmother telling me since I was little: you must receive it with both hands and thank the person. You can't just have someone pour water for you while you're on your phone – it shows a lack of upbringing. Another example, let's talk about young people, say, unmarried ones looking for a partner. Especially when the woman's family visits the man's family to check the situation and talk, if things progress, there are many customs, including one related to tea.

The man's family will prepare nice things, like food. The first step upon meeting is to pour tea for the guests – the way to treat visitors. If you drink the tea from the man's family, it implies... in our custom, what does it mean? "A family's daughter does not drink tea from two families." You cannot drink tea from two families. In your life, if you, as the matchmaker, see that I am interested... it's not just about that cup of tea. Before you leave, they also give you a packet of tea leaves. If you accept this packet of tea leaves and drink it, it completely signifies agreement. If you go back on your word, it's not acceptable. Why? Because in the past, tea trees could not be transplanted; they would die if moved. Among all shrubs, only the tea tree was like this. Nowadays it's possible, with scientific methods, but in the past, it meant you couldn't return or withdraw. The tea served represented a firm commitment.

Therefore, "a family's daughter does not drink tea from two families." The stories about tea in our Gaochun are numerous and profound, aren't they?

There are many. I've given specialized lectures. I've given several sessions on Gaochun TV about Gaochun's tea culture, starting from the Spring Festival. During Spring Festival, tea isn't just about the concept of tea leaves; it has representative foods, including eggs.

On the morning of the first day of the lunar New Year, generally speaking, on New Year's Day in Gaochun, when you visit someone's home, they serve what's called the "Three Cups of Tea." If people don't understand, what is the "Three Cups of Tea"? You should pay attention when you go. After you arrive, they first pour you a cup of sweet water – sugar water. What does this represent? It means they wish you sweetness from the beginning of the year to the end, from the first month to the last month – a sweet and harmonious year.

The second cup... in the sweet water, if they also put two or three red dates, it means "early." Especially for newlyweds, it means "may you have a child early." But some say it's not just for young men and women; it can be for old and young alike. They prepare the cup with two dates inside. Generally, for newlyweds, it means "wishing for a son early."

The third cup is real tea, brewed with tea leaves. They pour half a cup, not a full cup, so the water doesn't overflow. This is very particular etiquette. They also offer it with both hands, and you must also receive it properly. You cannot receive it with one hand; you must slowly accept it and promptly set it down, also expressing thanks.

Thirdly, what is the third item? The third is that each person is served three boiled eggs, sweetened. What do the three eggs symbolize? A sweet life. It must be three, not one or two. Only three, symbolizing "coming first in three examinations" (a wish for success). There are many, many such customs.

Many of these could be excavated today. If they were properly experienced, they could really make tourists stay. I've talked about all this on Gaochun TV.
