# Supplementary material for: Rurality representation and changes in rural tourism destination
Source: PLoS One. 2026 Apr 21;21(4):e0347226. doi: 10.1371/journal.pone.0347226 (PMC13098982; doi:10.1371/journal.pone.0347226)
Supplement: S1 File — (ZIP) [file pone.0347226.s001.zip › supporting information/大山村漆桥村录音及转译文本/QQ-JM 2.docx]

Q: Regarding our village... the residential areas, like here 13 years ago, the buildings here hadn't been touched. 13 years ago, they were unchanged. Wasn't it like that?

13 years ago, unchanged, not like this.

JM: The tiles, everything has been changed. The tiles over there were repaired, right? Look, this entire section hasn't been touched, but all the other areas have. Look at this door panel, see? This door panel has been replaced. This house was built in 2009. 2009 has the highest proportion, 2007 was even earlier, two years prior. Before, these houses were all untouched. Completely untouched. The changes only happened in the last three years. In total, what has been changed... and what remains unchanged is still fine. Don't think everything here has been altered; these old houses haven't been touched.

Q: Can you see that the stones are still the original ones?

The original... based on the residents... because some people say it's good, but actually, some things were just filled in later. The forms aren't necessarily as you just described, matching reality. Because you are involved and connected to it, right? I don't really know how to put it, to be honest. Is it better to be honest, or to submit something slightly different from others? My information might be a little off compared to others.

JM: You think that if back in '07, the village buildings had fewer repairs, they should still retain more of the original...

JM: Similar, but they have been repaired. They don't have the old style anymore. The residential areas have lost it. The original architecture, probably from the Ming and Qing periods, is gone. In 2007, it should have been better. Now, you might see things dated 2020 added behind. Some people, as I said, are not realistic. Let me be frank with you, it's better to be realistic, isn't it?

JM: Are there three? Are there any others you think? Around here.

Q: Qiqiao and the residents' lifestyle, their agricultural production... this depends on the individual, like now. If your conditions are good, you're a student; I'm a migrant worker or a farmer. The standard of living varies for each household. I'm not talking about living standards, but rather, back in 2007, the overall lifestyle of residents here was relatively more leisurely, right? The mode of production was primarily agriculture. That could reflect rural characteristics, right? Our main focus here was agriculture, so it should be quite representative of its character.

And are there any spiritual or cultural aspects?

JM: Baoping Well. Baoping Well existed before, but before 2007, the well was stopped. The mouth of the ancient well... for how many years? We were still young then. It could already be stolen. The wellhead had an opening; the wellhead was stolen.

Q: What does 'the wellhead was stolen' mean?

JM: Stolen means someone took it.

JM: It was stolen by someone, gone. Later, they just put a frame around it. When we were young, I remember it was right up front. Then it was filled in. Wasn't it filled in because they were afraid children or the elderly might have accidents? They filled it with earth. What was that about? Also, some locals had poor quality; previously it was gone, or they burned garbage and dumped it in there, into this net [area], into here.

Q: Isn't it repaired now?

JM: Now it's superficially repaired, but actually, it's not really. The well hasn't been dug out. Still not. No water, no water. It's not the same. It's a surface-level project, essentially. For instance, the Earth God Temple.

JM: The Earth God Temple was originally in the front, near the city gate area. Now, you've made it so there's a ditch there. The Earth God Temple has been moved here. The site is the earth god's land. It's like building a house; the temple's foundation should belong to it.

Q: Is the temple foundation at Nanling Pass? Roughly?

You, Nanling Pass... I'm from one village, but people are outside, I don't know.

Forest land... we have relatively little forest land here. Like around here, there is none. I can't just make things up.

Q: The situation in 2007: Was the river water quality relatively poor?

JM: Water quality... you have to base it on the water quality of the rivers. The well water... rarely mentioned. If you look at it, there is well water. The water quality now still isn't good. Really. Wild vegetables, weeds were plentiful. Animals... back in 2007, the number of animals wasn't that high either. Ancient architecture... the ancient Junzhou Old Street... the original风貌 (style/appearance) did exist.

Q: Was the ancient bridge repaired later?

JM: The ancient bridge... 2017... 2017... 13 years... 13 years ago it seems it had already been worked on.

Q: It was repaired, right?

JM: It was worked on. The original风貌 (style/appearance) was still okay, relatively good. The ancient streets and lanes are just like this.

Q: Rural modes of production... The ancient streets, lanes, fields... nothing much.

JM: Modes of production... modes of production: farming, growing vegetables. We were all farmers here before.

Q: Rural leisure and entertainment...

JM: Chatting, chatting... that is, some people also play cards. Their life, their livelihood... it's all about farmhouse fare.

Because our area has always... they could implant things long-term.

I don't know how our marriage is calculated or preserved... but our... regarding Baoping Well, good... especially the Nanjing City Cultural Relics Protection Unit. Right. You also... unlike how I would say... like I, truly protecting it here is also very good. You can only rate it three... rating it three times is normal. No matter where you go, this thing... brother, come to our Earth God Temple, etc. The ancient market... middle... pond and temple... relaxed. You rate it three or four... eight times three is twenty-four... because the Zhongchi Ancestral Hall is inside this gold ingot, nothing else. Temple.

Buddhism... look, if I... you rate three, rate three... you don't believe in Buddhism anyway. It's not about believing or not; I am happy, but according to your form, it doesn't exist. For example, me wearing these clothes gives people that kind of... the thing is gone, doesn't exist. Our Zhongci... it's gone later. It existed originally, right? Originally there was an ancestral hall. Our village mostly has the surname... many named Kong, few named Zha. Mixed surnames, they came from elsewhere. Before, people my age, like your parents or grandparents, they came here fleeing from disaster. Life was hard back then, it was like this. For example, if you are female and come to live here, it's also possible.

It's like this. Anyway, there are many happy people here... one name... our village originally should have been happy. We just talked about... culture, simple folk customs. In 2007, how was it? Simple and... cultural identity, that is, whether they identify with the village, cultural identity with the village... whether they like the village or want to go to the city. If the team is established, then it counts as not identifying. If they want to stay in the countryside, feel our village is very good, that is identification.

Now, people with slightly better conditions have mostly moved away. What do you think? I think in the countryside, later... your old... the land, you can't move it, right? From what you've heard, I think you still quite identify with it. You filled in 4... history... later people control... or you can listen... We now have a 2020 situation for Qiqiao Village. You can choose a few... the bottom goes to the door side... wrote small house bottom Li Fang became... the next four is doing that matter, not arranging about to投身 (devote/join), right? I'm fine all at once.

Q: 2020's Qiqiao Village, what elements do you think best represent its characteristics?

JM: Vegetables... go down and talk later, you wait a moment. Very comfortable. Are those three all telling me? This thing means the expert directly told him to pay attention to this problem. You go first, I haven't finished here yet. Also here. So now, is studying good or not?

Right? Isn't that fun? For example, I'll go see the front, there are seven... I'll call you two. First, which university? Nanjing Normal University. You haven't seen it? I've seen three Qiqiao. Since the child has come, they should experience it personally, right? Then people say you talk a lot, you tell them east and west, you tell them it's okay, you yourself don't know the situation. Since you've come, you should go experience it yourself. In 2020, what do you think best represents our... the biggest face? [Likely means 'most representative aspect']

Okay, let me ask Uncle. You say. You, from Nanjing Normal University... Nanjing Normal University can... become teachers, being a teacher is also good. Students from Nanjing Normal University, getting in wasn't easy back then either. Vegetable large... 0 construction site... now it's less, none... like my city, it's basically very little, but there is a bit, don't... There is a bit, right? A bit. Vegetable greenhouses. Vegetables are all agriculture. Agricultural vegetable greenhouses... some families do it for survival too. Only... also quite a bit more. Now, little by little... Xietian (thank the fields?) not very many. Behind are some... fields. Xietian is okay. Yangxian, this way.

Q: When did you start crab farming? I see you farm crabs.

JM: Crab farming... roughly 5 years. Five years. Started uniformly, about 5 years. Five years. Roughly... I can basically only say this. Actually, I'm not at home. Forest land is gone. Water city... has residents living... village residents... residential areas... also can't be counted, can't be counted. Anyway, people like us here aren't many. People... are young people? A few... young people? No, they are all elderly people, here for medical treatment. Some people work, after earning money, they buy houses, buy this and that. I'm not planning to come back either. Some who bought are elsewhere, some are in our... up there in Shuangpaishi area, Shuangfucheng... that garden area, how is it? Are they considered relocated households? Or not relocated? Our area uniformly moved there. They wouldn't agree. Some people still live here. Some are willing to go there, just go there. You don't want the money.

Q: Qiqiao Old Street... Qiqiao Old Street, what does it mean? We need to understand this street.

Our street, isn't it the characteristic of our village?

JM: This one is.

Q: You can stop. Residents' lifestyle, residents' mode of production. So we need to... lifestyle, depends on adjusting to each family's conditions, won't require... for example, some also... for example, if I want to eat better today, or have relatives to entertain for a few days, or endure hardship myself... this way, I really can't identify with it, right? I can't identify. Why can't I identify? Because each family's situation is different.

Q: Residents' leisure life...

JM: How do I say leisure... they also... law judgment? Not very short of money. Still busy. Some are busy, some aren't. Only the elderly, like those in their seventies or eighties, are all elderly. You can't say they are at leisure; elderly people are just like that. The young people's shortcoming is equivalent to... you young people... or like now, dancing a bit in the evening... college entrance exam? Generally, basically, city people have leisure activities. Here, it's very few, still relatively few. They work during the day, where... still relatively few, right?

Q: The last three, can we choose? Nanling Pass, how is it? Now Nanling...

You didn't have it before, and then it was newly built, right? You can put this on. There's also an evaluation. This is the last page. It's fine.

In 2020, how representative was the farmland for the village?

JM: I think, teacher, when you went to Bazhong later, caught a cold and handed in... then math... at least comprehensive wetland... not learning guitar like this anymore... not even last year... who knows who can come grasp it? Farmland is very representative. Isn't it the same as just mentioned? That was for 2007? You said 13 years ago? Now is 2020. How is it now? Farmland is gone, right? So... must be... farmland quantity is small. Common people also... forest land is gone. That is also... Xietian, these days all exist.

Q: Water areas, got better or worse?

JM: Originally, it should be... filled was three.

Water... got worse. You listen, SAD, no good. Don't believe me, I'll take you down to see. What you originally filled was one category, now is also one... those you can... now someone owes you... position, how? Eaten weeds or what? You rate two... door.

When... people are very few now. Ancient architecture. Ancient architecture. But when he got to Beijing, absolutely can only rate... nothing much, nothing. Actually, can only be like this. Did you see? You saw it yourself, one blow. This is what I saw with my own eyes, and what I said. You can only fill it like this. Go feel it yourself. If you're willing to fill it, then fill it. Fill is fill... sky I go fill... To be honest, you think it should be... I don't feel great about this. My conscience tells me, I'm still young, and it's not easy for you either, right? Let's look again. Good is good, bad is bad, it's fine.

I think it's okay.

Q: You think it's good? Including this row of newly built ones, actually the feeling is not bad. Maybe that gate... ancient... old street and... I can govern people... up to three. You can't rate four... language... because haven't talked about where, not repaired well yet, needs improvement. Ancient bridge upgrade.

2013 ancient bridge Qiqiao had evacuation. Repair meaning? Repair is still ongoing. Compared to before, now with repairs, it's more complete, more good-looking, more beautiful. When Americans good. No. Didn't hear. Can only hear the top.

To... before, like my parents' time, they couldn't reach this level at all. Money was saved... like you, like now, like these are all somewhat vain. According to the elderly, can only hear... now is also not good, right?

No... back then... at that time, people walked back and forth on our street, like a market. Now you can't reach that level. Your maintenance is not good either. Really, why would I lie to you? Look, after a few times it's broken. Originally, if people lived there, they would definitely repair it well. Young people at home... school, old street... ancient... this is now... the current... human resources... Tian'er not good, right? I think they are all poets. Open a shop, then people... I moved away. It's like renting a house; I moved away, this door is still closed. Commercial shops are not good, not like... look now, no one. It's not like that.

Small city things: farming, growing vegetables, commercial work, migrant labor. Now, this is our village's current mode of production. Probably the middle ones are really few now, right? Overall less. This... comparatively... Zhongcaishi... from the mountain... migrant workers all exist. Do you think this lifestyle, this mode of production, can represent the countryside? Our countryside, actually, should be... from this perspective, it can represent. Now people are all outside. Represent... you call him... he won't... poet take money out. No, so those from outside are all from other places.

From other places. Now goods are all about buying and selling. Isn't the stuff sold in the front all from elsewhere? Situation... are there locals? All are locals. All are local people. Local are local people. You also can't... His mode of production and lifestyle... anyway, not far from our road. Can you choose three? You choose three.

Q: Leisure and entertainment activities, now basically playing on phones is more common. Playing phones, computers, tourism... his small target... our college, these, some, not many, very few, right?

JM: Less. Just put less. Its representativeness will be poorer, right? Lifestyle, leisure, entertainment methods, right? Giving you the choice is not wrong.

Life is relatively busier. Everyone is busy, bustling. Before, maybe... historic sites, relics... these changes should not be big. Not big changes. We chose 3 and 5 before, but now choose what? Contract? In 2007, it was not good, average. Now is also like this. Now also hasn't been touched.

Because isn't this... the ancestral hall is gone now? Isn't that worse? The ancestral hall is gone. None. Yours is still like this, then that's it. It's like this. No need to be so polite, it's fine. I'm mainly... today also... went to university. Your nephew, which university did he go to? He's in aviation... Nanjing, Nanhang? So impressive? Is it better than your normal university? Impressive? Then... big man good, how... by male student... is he in first year? He's in third year. This year is the second year? He studies 4 years, second year or third year? End of the year, all second year. Second year should be... take responsibility... new phone, don't swipe. It's fine, thank you. I'll go find them. Thank you, thank you. Thank you, Teacher Ma. Welcome, you all later.
