# Supplementary material for: Rurality representation and changes in rural tourism destination
Source: PLoS One. 2026 Apr 21;21(4):e0347226. doi: 10.1371/journal.pone.0347226 (PMC13098982; doi:10.1371/journal.pone.0347226)
Supplement: S1 File — (ZIP) [file pone.0347226.s001.zip › supporting information/大山村漆桥村录音及转译文本/QQ-YK 1.docx]

Q: May I ask where you have traveled from?

A: Nanjing.

Q: What was the rural village like in your memory?

A: The countryside was quite similar to this too. Growing rice, growing wheat, and then having vegetables at your own doorstep.

Q: And what were the leisure activities in the countryside before?

A: There were none. Farmers didn't have activities, didn't have such needs. Otherwise, they'd watch movies.

Or, activities back then were watching movies together on a big screen. Actually, I... anyway, you didn't have to buy it, you could just go watch it yourself when the time came.

Q: What about shopping and such?

A: Generally didn't buy much either. Anyway, our parents would buy things here, that's how we got what we needed. Yes, now we all go to the supermarket to buy, right? Before, in the countryside, regarding appliances... adults found... ...Thief spiritual? This phrase... especially... it was parents worrying, we didn't bother. Parents worried, you didn't need to bother. And before,

The land area for each household was very large. Yes, some had several hundred mu, even several thousand mu of land. That was the People's Commune. Especially, this piece of land was our production capacity, contracted by a production team, and then allocated to each household within it – it was collective.

Now it's all built up with houses, otherwise, it's used for greening, building parks.

Q: Do you still raise poultry now?

A: Before, families raised chickens, ducks, geese, pigs... (ears?)... and raised rabbits. Then sold them... this month's procurement... had 10 specifically buying fresh ones, took them out to sell, for export.

Q: May I ask, what about your household registration now? Is it urban registration?

A: Yes.

Q: Would you choose to have an urban registration or a rural registration?

A: You want to try it? Because now rural registration comes with land. We ourselves are urban registration, have been since childhood, urban registration. And now we don't want to change it anymore.

Q: What elements do you think best represent the countryside?

A: This village is quite good. For example, pastoral scenery, right? Small rivers, crops, these things.

Q: What things make you think, "This is the countryside"?

A: Temple fairs, dragon and lion dances. These belong to one kind of folk activity.

Nowadays, various rural areas in inland China still have them.

Dragon and lion dances – you can still see them during festivals. Just add a festival and you can see them. Yes, if there's that festival, they have it too.

Q: In the past, what elements best represented the countryside? What do you think were the most representative elements of the countryside?

A: Still being able to maintain these old traditions. Actually, I... when I go out... it's things like the customs during the New Year period that most represent the countryside. The countryside was livelier before?

Q: And what is your ideal countryside like? What is your vision of an ideal countryside?

A: Gaochun and Lishui are different. The countryside has its own various geological conditions; the geological situations are different.

Q: Where do you think the differences lie?

A: Some places can grow things, can produce grain; other places cannot. It's related to the soil quality, related to all aspects.

Gaochun is half paddy fields, half vegetable plots. It's not against mountains; it's quite good, quite prosperous.

Q: Then may I ask, what are your thoughts on the current state of transportation, information, and capital?

A: No need to worry about transportation, it's quite good, right?

Q: And the impact on the countryside?

A: It lets outside tourists come... people from wherever... I come to travel and still pay.

Poverty alleviation, because the investment is quite good. The construction of a moderately prosperous society accelerates.

Q: Would you choose to experience the agritourism here?

A: I feel the agritourism is pretty good.

Q: For example, in some places, the water quality of rivers and lakes, air quality...

A: The air in the countryside is better than in the city.

Q: And what cultural experiences do you think Gaochun provides to tourists during the process of building the Slow City tourism?

Q: For example, in terms of food, accommodation, transportation, travel, shopping, entertainment – these are the experiential cultural scenarios.

Have you experienced any of the local folk culture here, for example, Tiao Wu Chang, Da Ma Deng, Da Shui Hu (likely local folk performances)?

A: I've seen these mentioned in hotel brochures, but haven't experienced them myself.

During Chinese New Year, there are many people.

Wood carving... thousand-layer... ceramics, making pottery. No.

Q: What about festival activities? There's also the Jinhuajie (Golden Flower Festival), Changjieyan (Long Street Banquet), have you heard of them? Have you heard?

A: Rapeseed flowers.
