# Supplementary material for: Rurality representation and changes in rural tourism destination
Source: PLoS One. 2026 Apr 21;21(4):e0347226. doi: 10.1371/journal.pone.0347226 (PMC13098982; doi:10.1371/journal.pone.0347226)
Supplement: S1 File — (ZIP) [file pone.0347226.s001.zip › supporting information/大山村漆桥村录音及转译文本/QQ-YK 2.docx]

Q: May I ask where you are traveling from and how long you've been here?

A: I'm a tourist. I've been here for 4 days; it's my first time.

Q: What do you think about the consumption level in the Slow City?

A: I think accommodation is quite cheap, but the food... this aspect, I feel there's not much difference from the city, might even be slightly more expensive. It could be because I chose agritourism establishments.

Q: How did you find out about the Slow City in Yaxi? What are your experiences and feelings regarding the "slow tourism" experience?

A: I learned about it from some promotional videos and articles. My experience with slow tourism is average, and my feelings are that it doesn't have particularly distinctive features.

Q: What about the cultural experiences during your trip to the Slow City?

A: I did see some Slow City landscapes, like snails, grass sculptures, wood carvings, etc. I also saw some traditional handicrafts, like feather fans. But regarding folk culture and characteristic eco-cultural festival activities – maybe because I came at the wrong time or for other reasons – I didn't experience much.

Among the intangible cultural aspects, the slow pace of life – indeed, the pace of life slows down here. The quality and atmosphere of slow living are present. As for interacting with residents, it was mainly with people at the agritourism places and the homestays where I stayed. I didn't have much interaction or feel much connection with the actual community residents, those locals not involved in the tourism industry.

Q: What are your thoughts on the publicity and infrastructure of the Slow City?

A: I feel Gaochun's Slow City promotional work is actually quite good and well-known. Many people around me know about it, probably also because I'm from the area, close to Gaochun, near Nanjing, so everyone knows something about it.

Regarding infrastructure, it feels divided into two parts. Firstly, the construction inside is actually good – transportation roads, including tourist public toilets, etc., are quite well-built, not worse than some 4A-level scenic spots. As for entertainment facilities, after all, it's a Slow City; having too many entertainment facilities would, I feel, somewhat contradict the concept of a Slow City. The state of entertainment facilities seems fairly normal; perhaps they don't need to be built so extensively. But there's one problem: getting from downtown Nanjing to Gaochun requires driving. Driving in by self-driving tour and getting back is especially inconvenient.

I think Gaochun could do better in terms of public transportation – having public transport bring you here so I don't have to drive in myself and then drive around inside. Because if you come by self-drive, you might think of driving everywhere, which反而 might not allow you to slow down the pace. Maybe with friends or on your own, you might sometimes get lazy; if it's a good scenic spot, you just drive there and don't feel like walking. But walking might be more characteristic of a Slow City experience for landscape appreciation. Of course, this might also relate to us tourists' own self-discipline.

Q: How does the local cultural construction differ from other rural tourism destinations? What are its strengths and weaknesses?

A: This is one of the points I most want to discuss. I feel that compared to general rural tourism, the Slow City probably has better infrastructure, but it doesn't differ greatly from other rural tourism places. If I go from the city to the countryside for rural tourism, my pace of life will definitely slow down. But the cultural construction experience is supposed to be a distinctive feature. Here, I might have come at the wrong time, or perhaps I didn't perceive a difference in cultural reception compared to other tourism. What form should the cultural experience of slow tourism take? Actually, I feel that the Slow City's slow culture lacks a clear, overall, or unified standard, etc.

Coming to the Slow City this time, perhaps because my level of immersion or experience wasn't sufficient, I feel that Gaochun's Slow City might need more of a DIY format. For example, being here and seeing some characteristics, like feather fans, or things you mentioned like traditional handicrafts – wood carving, layered cloth shoes, Tiao Wu Chang, Da Ma Deng – I actually didn't see any of these. I feel they could open some shops for DIY involvement, teaching you how to make layered cloth shoes, how to make feather fans, how to do wood carving, and then watching Wu Chang performances, Da Ma Deng, Da Shui Hu, even performing it yourself, having someone teach you to perform.

That would feel great, I think. Or having some tea houses, etc., where you pick tea leaves yourself, then come back to brew and drink it. Or having villagers take us to some mountains where people usually don't go much, but safety is guaranteed, to walk around and look. I feel that might give me a greater sense of impact culturally. Coming here this time, I actually feel this place is essentially just agritourism plus some recreational facilities and activities. Of course, the scenery is very nice, but which rural tourism destination doesn't have agritourism?

If it's just about things like drifting, flowers, plants, and such projects, I might as well go to Disneyland or an amusement park. If Gaochun's Slow City wants to be truly exceptional, I hope it aligns with the memories we tourists hold about the countryside. The countryside is not an amusement park; the countryside is not merely a place to eat. Tourism is also not just a place to eat. Tourism involves food, accommodation, transportation, sightseeing, shopping, and entertainment—they are often interconnected. Focusing on just one sector is definitely not enough and is insufficient. This is the discrepancy between my expectations for Gaochun's slow tourism and the reality, and an area that needs improvement.

Q: If you were to travel to the Slow City again, would you come?

A: If I were to visit the Slow City again, I would definitely come. But if I had to give a true evaluation, I might prefer to use an expectant perspective for the rating. I also hope the Slow City can get better and better, and that each time I come, it shows progress.
