# Supplementary material for: Rurality representation and changes in rural tourism destination
Source: PLoS One. 2026 Apr 21;21(4):e0347226. doi: 10.1371/journal.pone.0347226 (PMC13098982; doi:10.1371/journal.pone.0347226)
Supplement: S1 File — (ZIP) [file pone.0347226.s001.zip › supporting information/大山村漆桥村录音及转译文本/QQ-YK 3.docx]

Basic Information:

(1) ID: DS21

(2) Gender: Female Age: 27 Occupation: Teacher

(3) Role: □ Resident ☑ Tourist

(4) Education Level: □ Junior high school or below □ High school (incl. technical secondary school) □ College & Undergraduate ☑ Master's degree or above

(5) Years lived locally:Involved in tourism:

(6) Annual Family Income: □ ≤ ¥10,000 ☑ ¥10,001–¥50,000 □ ¥50,001–¥100,000 □ > ¥100,000

(7) Resident Income Source (multiple choices): □ Farming □ Tourism-related services □ Other (Migrant work, Salaried job)

(8) Tourist Occupation: □ Company Employee ☑ Professional (Doctor, Lawyer, Teacher, etc.) □ Self-employed/Freelancer □ Student

Q: How many days have you been here?

A: Two days.

Q: How many times have you visited?

A: Once.

Q: What do you think of the consumption level in the Slow City?

A: It's okay, not very high.

Q: What cultural experiences do you think the Slow City provides for tourists?

A: The International Slow City symbol, the snail... It seems I haven't learned about local distinctive things yet.

Q: What about the intangible cultural aspects? Regarding the pace of life, quality of life, and the general atmosphere?

A: They are similar to the rural life I knew before – early to bed and early to rise, getting up to do some farm work. I saw some local residents getting up at 6:00 AM, picking vegetables from the fields and such. Seeing this feels very leisurely.

Q: What do you think of the infrastructure and recreational facilities here?

A: The infrastructure is passable; the roads are built quite well. And that place over there, does it count as a scenic area? The lawn we stayed at yesterday is part of the scenic area, including the rainbow slide and those things. It's acceptable. It seems the infrastructure could still be developed further.

Q: What differences do you see between the local cultural development here and other rural tourism destinations? What are the strengths and weaknesses?

A: I haven't really experienced much of a cultural sense.

Q: The overall atmosphere?

A: The overall atmosphere actually seems... because I also grew up in the countryside before, it seems our pace of life was quite similar to this, also very simple and honest. And they seem quite warm towards outsiders.

Q: You feel it's quite similar to your hometown. Does your hometown develop tourism?

A: No.

Q: So, this place is similar to rural villages that haven't developed tourism? Can I say that?

A: It's not the same either. The architecture here has a sense of being planned, deliberately planned. Ours is more like self-built rural houses, not like the unified style of the buildings here.

Q: What form do you imagine the cultural experience of 'slow tourism' should take? Can you envision it freely?

A: I think here... aren't the play facilities a bit few? It's suitable for slow walks, but maybe the season isn't right. If it were cooler, I might feel more like going for a walk after getting up in the morning, or taking a stroll after dinner to look around.

But there seem to be very few people here; you hardly see any residents. When we drove through, we didn't really see many people, just some houses that look quite well-built, giving a sense of being refined. I feel it's like a 'curated' countryside.

Q: Where do you think improvements are needed?

A: I feel there might be a bit too much human intervention.

Q: It should restore a more natural state, right?

A: Yes. With some planning and things, you can clearly tell it definitely wasn't like this originally. It was later redesigned and renovated to become like this.

> Also, for example, looking at the surroundings, I actually don't see distinctive local characteristics. Like those flowers – it feels like the whole country is planting that kind of flower. I went to Hebei before, and they were also sowing the same kind of flower, probably because they are very tenacious. I was wondering if they could plant some local species, things we can't see elsewhere, something unique that can only be seen here.

Q: What elements can you imagine that represented the countryside in the past?

A: In the past, I imagine wildflowers and weeds, then maybe pets wandering around, like native dogs, some local dogs, and cats. Also, houses that don't look very refined, but each house reflects the owners' own preferences. For example, some people like certain colors, making them different, not like the uniform grey or white. You could tell what style the homeowners preferred – that kind of feeling. Then, having a small yard, some small vegetable gardens, and often elderly people watering plants or picking vegetables in the morning – all very leisurely. Also, in the countryside, we had characteristic spots where, for instance, young people would gather to play cards or something, and elderly people would gather to play mahjong or chess – things like that. I haven't seen that here. I wonder if the connections between residents here are fewer than before.

Q: Some say the connections are deeper now, because they exchange recipes and share experiences, etc.

A: Maybe we just didn't see it.

Q: What differences do you see between the elements of the current countryside and what you just described? When you think of the 'current' countryside, what comes to mind?

A: Like what I've encountered, which also hasn't been designed for tourism, it's mostly similar to before. But indeed, regarding houses, it seems everyone builds them uniformly, quite grandly. Because in the countryside, self-built houses easily lead to keeping up with the Joneses – 'I earned money outside, made a fortune, I'll come back and build a tall, luxurious building.'

Q: What is your ideal countryside like?

A: My ideal countryside is very leisurely, with a plot of land in front where I can grow vegetables to eat or plant flowers, making it look beautiful. And preferably, there's a small pond too.

Q: Do you think tourism has a significant impact on the countryside now?

A: Quite significant.

Q: What aspects does it mainly affect? For example, good or bad impacts?

A: There should be many good impacts. Roads and such are built much better. Before, they were probably gravel, right? Or without asphalt, just cement roads. But now many seem to have asphalt. Also, probably the residents' lives... because of this, their income might be higher than before, which is also good. But there should be drawbacks too. For example, once it involves rural tourism, they might think about standardizing everything, which aspects to standardize, making everything look quite similar. Many rural tourism places seem very alike.

Q: If you were given some funds, would you prefer to develop its material, spiritual, or behavioral aspects? Can you rank them? Which would you prioritize first, then next, and so on?

A: Actually, I think the material aspect is okay; it really doesn't need to be as convenient as in the city. Material can be ranked last. The spiritual aspect is actually fine; you can experience that leisurely, relaxed feeling.
