# Supplementary material for: Rurality representation and changes in rural tourism destination
Source: PLoS One. 2026 Apr 21;21(4):e0347226. doi: 10.1371/journal.pone.0347226 (PMC13098982; doi:10.1371/journal.pone.0347226)
Supplement: S1 File — (ZIP) [file pone.0347226.s001.zip › supporting information/大山村漆桥村录音及转译文本/QQ-YK 4.docx]

Q1: May I ask how many times you have been here?

A1: I have been here at least a double-digit number of times—more than ten times.

Q2: How would you describe the cultural experiences this place has brought you?

A2: Let's see. It was probably five or six years ago, when this place was just being built. Back then, it was in the suburbs of Nanjing, quite far from the main urban area. Unlike Jiangning, which has many beautiful villages that are more attractive to urban residents, this place needed something extra. To spark people's memories of the countryside—using an intellectual's term—it had to adopt a more sophisticated name: "Slow City." I remember Slow City originated from Italy as a cultural response to aging. It promotes a slow-paced lifestyle. Chinese people didn't used to favor this, but after experiencing fast-paced life, they've come to appreciate different ways of living. It lets them see what modern rural areas are really like, making this place a good representative of high-quality new countryside.

Q3: Beyond cultural experiences, have you noticed any changes in your lifestyle—like adopting a slower rhythm or improving your quality of life—from traveling here?

A3: Just visiting for a short trip won't let you feel it deeply. My suggestion is: if you have the chance, don't just "tour" Slow City—stay for a few days. Only then can you truly experience the "slowness." This slowness reflects contentment with nature, one's life, and current state. It's only when humans, nature, and heaven are in harmony that you can feel the benefits of this slowness—whether it's spiritual enjoyment, physical and mental pleasure, or relaxation after intense stress. Chinese people often travel in a hasty way: taking photos, eating, singing, and then forgetting everything once they leave. That's a common issue. What's needed is a deep, immersive way of leisure.

Q4: You mentioned the village has a long history. Could you elaborate?

A4: This village does have a decent history. It preserves traditional stories and cultural elements from bygone eras—for example, ancient trees that are 500 to 600 years old, proving its long-standing existence. After so many years, the village has evolved into a modern and charming rural area, which shows it has a profound cultural foundation. Traditional culture has strong cohesive power; otherwise, the village would have scattered. With urbanization, many young people have moved to cities, leaving behind mostly the elderly, rural women, and left-behind children in many villages. But you rarely see that here. It means the village is doing well economically, and people are satisfied with their lives.

Q5: Do you think there's any gap between your ideal Slow City and this place? Are there areas for improvement? A5: Well, every time I come, I mainly want to experience what Slow City is like and see if the trip meets my personal needs. For middle-aged and elderly people like me, it has a great positive impact on the mind. But I wouldn't recommend it to young people—they might not adapt to the rural lifestyle. There's not much "fun" in the conventional sense; to put it bluntly, it's not much different from just waiting around with nothing to do if you can't calm down. It's perfect for people who are busy with work, have a stable or high income, and want to enjoy peace and quiet. Young people with restless minds won't appreciate it.

Q6: How does this place differ from other rural tourist spots? What are its strengths and weaknesses?

A6: Let's start with strengths. As for weaknesses, the farm stays here are developing too fast. Too many commercial farm stays make the area feel overly commercialized. What we really like is going to a local family's home and having a casual meal. I've been to Hongcun and Xidi in Anhui. They have many homestays that preserve their cultural heritage without damaging their original appearance. You can go to a local's home, and they'll immediately serve you food. Here, though, you can tell at a glance which places are homestays or businesses—with signs, deliberate decorations, and commercialized settings. We don't need that. A simple courtyard should be enough; when you arrive at a home, you naturally expect to have a meal without all the commercial frills.

Q7: Do you have any other suggestions for the development of Slow City?

A7: First, there aren't enough entrepreneurial opportunities for young people.创客 (makers) are popular now, but there's a lack of elements to attract young entrepreneurs here. I suggest encouraging local college students or young people to start businesses—like running homestays, but with a young perspective. They could add modern touches, such as typical wall paintings or contemporary books, which would make the place feel different. The village committee and town government should consider supporting young entrepreneurs. Second, some cultural facilities are underutilized. I've been here many times but haven't visited places like Slow City Town, the market, or Slow City Academy. Some built facilities are deserted because they're too far from the tourist center. People just stay for a night, eat, and leave—nothing leaves a deep impression. Third, adhere to the principle of "restoring old buildings to their original state." There used to be a Farming Culture Museum here. I remember there was even someone playing drums there before, but now it's messy with clutter. If we don't incorporate some commerce, we can't achieve economic benefits. But if we overdo it, we lose the essence of "slowness." It's a difficult conflict to balance between culture and economy.

Q8: What does the countryside look like in your memory?

A8: When I think of the countryside, I think of the 1970s and 1980s—curling smoke from kitchen chimneys. Three scenes stand out: first, many elderly people chatting idly; second, middle-aged and elderly women washing clothes and vegetables by the river, so there should be more waterfront areas in the construction; third, raising cats and dogs, though now there are more restrictions on breeding, so you don't see that much anymore.

Q9: With transportation, capital, and tourism pouring into the countryside, what impacts do you think this has brought?

A9: It's normal for the internet, communications, and transportation to enter rural areas with modern life—it's a result of reform and opening up. General Secretary Xi has made it clear that sharing the fruits of reform is a basic principle of the Communist Party of China's governance. Without this, the Party wouldn't have the over 80% approval rating among the people, which is an extraordinary achievement. The government is truly committed to rural development, including the Beautiful Countryside initiative and poverty alleviation. Nanjing completed poverty alleviation in 2009, but we still need to consolidate the results with the same intensity as before. Nowadays, rural areas look beautiful everywhere, but there are invisible issues. For example, some people remain poor due to illness, disability, or accidents. However, village committees are often rigid in addressing these problems. We need social organizations to provide professional support—such as social governance and villager autonomy—instead of just one-time assistance. I once visited a family: the husband, under 40, was disabled in a car accident. His wife left because she couldn't bear the hardship, leaving their child in the care of his parents, who are in their 70s or 80s. The government provides some annual subsidies, but when the elderly pass away, the disabled man—who will still be middle-aged—will have no one to take care of him. This is where professional social organizations are needed to support such groups. --- Would you like me to help you refine the English translation of specific Q&A segments (e.g., adjusting the tone to be more formal or colloquial) or create a bilingual Q&A document for easier reference?
