# Supplementary material for: Rurality representation and changes in rural tourism destination
Source: PLoS One. 2026 Apr 21;21(4):e0347226. doi: 10.1371/journal.pone.0347226 (PMC13098982; doi:10.1371/journal.pone.0347226)
Supplement: S1 File — (ZIP) [file pone.0347226.s001.zip › supporting information/大山村漆桥村录音及转译文本/QQ-YK 5.docx]

Basic Information:

(1) ID:DS07(e.g., SA/DS/QQ-00)

(2) Gender: Male Age: 39 Occupation: Corporate Employee

(3) Role: □ Resident √ Tourist

(4) Education Level: □ Junior high school or below □ High school (incl. technical secondary school) √ College or Undergraduate □ Master's degree or above

(5) Length of local residence:Participation in tourism:

(6) Annual Household Income: □ ≤10,000 □ 10,001~50,000 √ 50,001~100,000 □ >100,000

(7) Source of resident income (multiple choice): □ Farming □ Tourism-related services □ Other (Migrant work, Office job)

(8) Tourist Occupation: √ Corporate Employee □ Professional (Doctor, Lawyer, Teacher, etc.)

□ Self-employed / Freelancer □ Student

Q: May I ask if you are a resident, a tourist, or staff here?

A: Tourist.

Q: How many days have you been traveling here?

A: Two days.

Q: How many times have you been here?

A: Many times.

Q: How many days do you plan to stay this time?

A: Three days.

Q: What do you think about the consumption level here?

A: Average.

Q: How did you hear about this place? Through recommendations from friends/relatives, official accounts, travel agencies, etc.?

A: Official account.

Q: What is your experience and feeling regarding the Slow City and slow tourism?

A: It's mainly a feeling about life. Perhaps like someone from a cultural background, it's a feeling of an open lifestyle.

Q: What kind of cultural experiences does the Slow City provide during your slow tourism? Because it's a Slow City, it must have a slow culture. How do you understand it? Feel free to share.

A: I think 'slow culture' in China should represent a kind of happiness. But it lacks... currently, I feel what the Slow City lacks most is this cultural depth. It's missing cultural depth.

Q: In terms of the things you can see and experience - like food, accommodation, transportation, sightseeing, shopping, entertainment - what cultural sense do you get from them?

A: I feel the strongest thing it gives me now is a restoration of the old rural lifestyle. That's about it for now.

Q: Regarding the intangible cultural aspects, such as pace of life, quality of life, life atmosphere, what are your thoughts? For people like us, what are your feelings? I think for us...

A: For people with a fast-paced life and work rhythm, it might be an experience of slow living.

Q: What do you think about the current publicity efforts for the Slow City?

A: The publicity is okay. The channels for promotion are quite extensive, reaching many places. Even CCTV has promoted it.

Q: What do you think about the infrastructure and entertainment activities here?

A: I think the infrastructure is quite good. But entertainment activities might be somewhat contradictory to the concept of a Slow City.

Q: How is the local cultural development here different from other rural tourism destinations? What are its strengths and weaknesses?

A: Local cultural development... each place's culture is definitely different. You can't have two places with the exact same culture, that's for sure. It just depends on how you excavate your local culture, how you use your local culture, highlight your strengths, and make a comparison with others.

Q: What differences do you see between the current development here and other well-developed places?

A: Currently, regarding rural tourism everywhere, it's largely the same, stereotypical. This place hasn't really built any distinctive features. I think if you look at the whole Nanjing area, its rural tourism also hasn't achieved 'One Village, One Product'.

Q: When you first heard about slow tourism and Slow City, what kind of vision did you have for this place? What did you think it should be like?

A: I think for something called Slow City and slow tourism, from our perspective, it should have a feeling of 'misty and rainy Jiangnan'. The scenery here isn't quite good enough yet. Actually, I think it's natural scenery, they haven't focused on it... I think its biggest characteristic is what? This place is genuine, without much added embellishment. For example, things like trees aren't overly decorated, it's still different from artificially created urban landscapes.

Q: Yes. What differences or deviations do you see between the current state of development and your ideal vision of a slow tourism destination?

A: I think its development, considering this specific location, the rural planning is probably still quite similar.

Q: What were the key elements in your memory [of rural areas], and what are they now?

A: The rural area in memory should have the flavor of childhood. The rural area now... probably because many things change with time, the countryside definitely changes too, it's also being built and developed. So I feel many of the original folk customs and culture are not preserved now, they are lost.

Q: What do you think best represented the rural areas in the past? And what best represents them now? You can compare the two.

A: I think perhaps the old rural culture was 'work at sunrise, rest at sunset'. For instance, when people had meals, they would even set up tables at the village entrance. The key element for rural areas now, I think, should be having complete infrastructure and places for enriching the villagers' spiritual and cultural activities.

Q: What is your ideal rural area like?

A: My ideal rural area should be livable. It should encompass a very broad range, containing many things, I think.

Q: What impacts has the influx of transportation, information, capital, and tourism into rural areas brought?

A: What's promoted now is rural tourism. Whether it's improved transportation or the injection of tourism funds, it definitely brings vitality to the villages. These things bring some vitality.

Q: What impact does tourism have on the physical elements of rural areas? For example, paddy fields, vegetable plots, ancient wells, old streets, old alleys, etc.

A: I don't think it has much impact.

Q: What about on behaviors, such as early to bed, early to rise, slow pace of life, etc.?

A: For rural areas, I think it might be complementary. Rural tourism brings economic income to the locals, but you inevitably have to give up some things. You can't expect to maintain the original lifestyle and still earn money; that's certainly unrealistic.

Q: And on spiritual elements, such as farmers' identity, ethnic festivals, religious beliefs, moral customs, cultural confidence, etc.?

A: I think this should be an autonomous behavior of the villagers.

Q: Okay, what attracts you to visit rural tourism destinations?

A: I think the reasons for me coming to rural tourism are varied, there are many reasons.

Q: What is your favorite rural element?

A: My favorite rural element is... I think coming to the countryside, I can feel this kind of simple, honest folk customs.
