# Supplementary material for: Rurality representation and changes in rural tourism destination
Source: PLoS One. 2026 Apr 21;21(4):e0347226. doi: 10.1371/journal.pone.0347226 (PMC13098982; doi:10.1371/journal.pone.0347226)
Supplement: S1 File — (ZIP) [file pone.0347226.s001.zip › supporting information/世凹村录音及转译文本/jsa11d.doc]

Q: I'd like to know, what things attracted you to come here?

A: YK: Leisure, rest. The air is good here.

Q: How many times is this for you, coming here?

A: YK: Come often.

Q: Compared to previous visits, what changes have you noticed here? Maybe not too significant?

A: YK: I came when it first started development.

Q: Did you know this area before the development started?

A: YK: Didn't know. After the shops opened, that's when you came... (unclear). Been coming here for many years.

Q: Now, you see, this is a rural tourism spot, right? What elements do you think a rural tourism spot should contain to attract visitors?

A: YK: This place is alright. Fewer people is good. The food is okay.

Q: What do you think is the most special aspect of this village?

A: YK: Quite relaxing. Not too many people.

Q: How has the transportation been here in the last couple of years?

A: YK: Transportation is very convenient.

Q: After the tourism development here, do you think it has damaged the rural landscape, or better preserved it?

A: YK: Can't really call it damage. It's better now than before. Before the development, this place was all shabby and run-down.

Q: When was the earliest time you came?

A: YK: You've been coming for 5 years? I've been coming for more than 5 years.

Q: It was developed around 2012.

A: YK: Roughly. I started coming around 2012.

YK: When I came, it was already quite nice. Already like this.

Q: What do you think of the architectural style here?

A: YK: I think the village's architectural style is quite good, quite distinctive. Yes, the black and white ancient residential style, that kind.

Q: Have you experienced any agricultural activities here, like fruit picking or farming?

A: YK: No.

Q: If such activities were available, would you be interested?

A: YK: Yes, definitely would take kids. Would take kids. Definitely... no other option (likely meaning 'definitely would'). The two of us just come for tea and meals. When the kids are older, we'll bring them.

Q: Does this village have any folk culture, based on your understanding from visiting so many years? For example, we understand there's Zheng He's Tomb here, it belongs to this area. There are some historical and cultural sites. Are you interested in those?

A: YK: Don't really know the past history. I don't come here to study. We just come to play, relax. Can't be bothered with those things.

Q: In your ideal view, what kind of rural village do you envision, what kind of countryside do you yearn for?

A: YK: Just like this. Exactly like this. Few people. But, man-made landscapes are not as good as the original, that would actually be damaging, right?

YK: There are no local specialties here.

YK: Maybe just some dishes.

YK: Haven't really seen any special dishes either.

YK: I haven't seen any of the households having farmland.

Q: Right, they have no farmland now. This area used to be fields. Originally, it was farmland, right?

A: YK: Gone. And this area was fields.

Q: Right, now there might still be some... maybe some families have small reserved plots, grow some vegetables. There might still be a little bit.

A: YK: There are quite a few chicken farms here and such.

Q: How old are you?

A: YK: 55.

Q: What is your occupation?

A: YK: Office worker. Sit in an office.

Q: What is your education level?

A: YK: College Diploma (Da Zhuan).

Q: (Likely summarizing YK's opinions): You think this place is good, the architecture is acceptable. You feel the countryside should be like this, first the houses were changed to Hui-style architecture, which looks quite nice. Like the Shitang Renjia in front, Shitang Renjia is also like this.

A: YK: The areas around Nanjing seem to be basically like this.

YK: In Jiangning here, basically all developments are like this. They are all okay.

YK: We come to the tourist spots in Jiangning all the time. Come here too often.

YK: No fruit picking. There was an orchard over there before, a peach orchard.

YK: Logically, it should be a peach orchard.

Q: Look at this 2003 map. Look at the old map of this area, these fields. Look, our current square is this area.

A: YK: If you say it was like that back then, how unattractive! When I first came, it was only half built, the back part wasn't done. So it felt terribly bad. But the front part, once developed, felt very nice. Just different.

Once those landscapes were built, once the roads were connected...

YK: Back then, Niushou Mountain wasn't developed yet. Right, Niushou Mountain wasn't built.

YK: If the countryside doesn't have this... it wouldn't be called countryside. This is the manifestation of the countryside.
