# Supplementary material for: Rurality representation and changes in rural tourism destination
Source: PLoS One. 2026 Apr 21;21(4):e0347226. doi: 10.1371/journal.pone.0347226 (PMC13098982; doi:10.1371/journal.pone.0347226)
Supplement: S1 File — (ZIP) [file pone.0347226.s001.zip › supporting information/世凹村录音及转译文本/jsa12.m4a.doc]

Q: When did you come here?

A: JM: Been here for a few years now.

Q: What was this place like when you first arrived?

A: JM: When I first came, this place was all bamboo, undeveloped, a very natural kind of landscape.

This was farmland, this was a pond, this was fields, over here were all fields. And there were original single-story tile-roofed houses. Now, look, there are these agritainment businesses. Look, new roads were built, many new agritainment places were constructed.

Q: So, may I ask, since we are developing rural tourism here, what do you think is the element that best represents the rural village? What comes to mind when you think of the countryside or a rural town?

A: JM: Probably the good environment. The environment is very good.

Q: What about the pace of life?

A: JM: The pace of life is definitely good now. The pace of life is better.

Q: What was it like originally?

A: JM: Originally, it was just the typical rural kind...

Q: Was it busier before?

A: JM: I don't know about originally.

Q: Did you live in the city before?

A: JM: Haven't lived in the city. I wasn't here back then either. Anyway, I've been here for a while.

Q: Are there any characteristics of rural people? Are rural people different from city people? For example, in how they treat people or handle affairs?

A: JM: Not really different in that aspect. Just now, relatively, people are the same as city people.

Q: Are you talking about their quality, or something else? Their quality, including their character – are city people sharper, while rural people are more simple and honest?

A: JM: The tourists who come here are also quite okay, their quality is quite high too.

Q: Okay, maybe you can't perceive a difference. Since you've been living here recently, do you feel interpersonal relationships have become more distant, or anything like that? Neighborly relations.

A: JM: Seems like there's no relationship? What do you mean? I wasn't in this village before anyway.

Q: How long have you been here roughly? Been here for years? Have you noticed any changes over these years? Is everything else okay or not?

Q: Just based on your impression, do you think interpersonal relationships in rural towns are any different from now? In your mind, what was the feeling of rural relationships actually like?

A: JM: Don't know. The boss is also from out of town. Do you know where the boss is from? He has several partners inside, several people opened it together, right? Right. It's very hard. They've been doing it for four or five years already.

It's the 4th year. How's business this year? I'd like to ask you.

JM: Business is a bit worse this year.

Q: Is the boss here?

A: JM: The boss isn't here.

Q: Is there someone in charge I can talk to?

(No direct answer recorded)

Q: The original countryside involved farming. Now, the countryside probably has less farming, development is getting better and better, right? Originally, crops like growing vegetables, was there more of that before, and is it becoming less now? Growing vegetables.

A: JM: Definitely. Now, if you grow vegetables, look, having your own vegetables is at the very least greener than buying from outside, right? Probably tastier and healthier. Vegetables like these are all grown by ourselves. This plot, that piece, are all grown by us.

Q: Besides these changes, do you think there are others, for example, regarding household waste? Any changes?

A: JM: Household waste is handled quite well now.

Q: What was it like originally?

A: JM: Originally... originally, someone said... now there are still people who collect it. It has always been maintained, this is quite good. It should be outsourced by the government here. Outsourced to them to handle. Someone comes to collect, someone manages it.

Q: So the whole environment in the village is very tidy and clean now, right?

A: Right.

Q: When we were young, the countryside might have been more natural but also unmanaged, right? Probably messier.

A: JM: Yes. Right? Yes. Look now, for example, the countryside has specific people collecting garbage now. This aspect is managed. Did we have this in the countryside before? Right? Before it was just dirt ground, then... dumped in the fields or ditches. Right. Now look, the countryside is developed quite well. For example, this point about developing tourism, right?

Q: What about the water quality here originally? Any changes in water quality in the last couple of years?

A: JM: The water quality has always been like this.

Q: It's always been tap water, right? Not well water. I mean, the water quality in the lake, how is it?

A: JM: The water quality in the lake has always been like this. You haven't noticed any changes? It's always been this way. It was originally like this.
