# Supplementary material for: Rurality representation and changes in rural tourism destination
Source: PLoS One. 2026 Apr 21;21(4):e0347226. doi: 10.1371/journal.pone.0347226 (PMC13098982; doi:10.1371/journal.pone.0347226)
Supplement: S1 File — (ZIP) [file pone.0347226.s001.zip › supporting information/世凹村录音及转译文本/jsa13.m4a.doc]

Q: You're probably not very familiar with this area, are you?

A: It's alright. What questions do you have?

Q: Look, this shows the changes in this rural area. Originally it was like this. This is a remote sensing image of the area. All this was farmland.

A: JM: Wow. How many years ago was this?

Q: 2000. It's from 2000. The current situation is that new agritainment businesses have been built, roads were built, a square was constructed. What changes have you noticed in the village these past couple of years?

A: JM: For our village this year, regarding the pandemic... hasn't been... because I only came this year.

Q: Looking at these two pictures I showed you, what would you say is the biggest change in the village?

A: JM: Cleaner and more sanitary than before. And there are more agritainment businesses than before. Also, their upgrades... seems like hospitality services are better than before. The service level is higher. Also, there seem to be more customers than before. This year, even with the pandemic, our place, because we are in a relatively good environment, has been okay, business is alright.

Q: So, since you are developing rural tourism here, what do you think is the element that best represents the rural village?

A: JM: I think it still needs to have some distinctive features, and some local characteristics. The elements here... it's the local dishes, the cuisine of the agritainment businesses. As for other things, I think this tourist spot relies on Niushou Mountain. Besides that, there isn't much... it depends on Niushou Mountain, including its Buddhist culture and its natural scenery. But our natural scenery here around March-April should be quite nice, all peach blossoms, peach orchards. Right. Because this is, after all, Shi'ao Taoyuan (Peach Blossom Spring). The cultural atmosphere is still quite strong.

Q: Based on your impression, what were interpersonal relationships like in the village before? What are they like now?

A: JM: Now, because there seems to be a community group chat, interpersonal relationships... we are all fine. Communication online is probably more frequent, both online and in person. Right, neighborly relations are often still quite good. And we do mutual referrals. If we have guests, we introduce them... for example, if someone needs accommodation, and we don't have lodging here but someone has finished eating and drinking and wants a place to stay for clients, we will refer them to a place that has lodging. If lodging guests want a meal, then we will also refer them to our restaurant. So it's a mutual... what's it called? Helping each other, mutual referral.

Q: What is the pace of life like here? Is it fast, slow, or relatively leisurely?

A: Preferentially slower, right.

Q: Compared to before, meaning before tourism development in this place.

A: JM: So maybe not too familiar with the town, because our shop has only been here for ten years. Before that, we weren't too clear.

Q: Just asked, have there been any changes in water quality here?

A: JM: Water quality... from what I understand, the water quality here is okay. Before it seems it wasn't very good, but now it's pretty good. Went from 'okay' to 'good'.

Q: You mentioned the attached pictures I just showed you? What do you think about the overall changes? Do you think this kind of change is for the better? Or not good? For example, regarding some old buildings, do you think they should have been preserved, or things like that?

A: JM: Transportation is also getting more and more convenient. Originally it was probably a very isolated place, seems like it should have been a very isolated place. Now transportation is more and more convenient, right? Public buses, Didi, you can hail a ride.

I JM think the way we are now is still quite good. Much better than before. Because before I think it was a bit damaged and dilapidated. Probably before it still felt a bit rundown here. Right. Now, after the organization, I feel the renovations are quite good.

Q: I see many buildings here use Hui-style architecture. But do you think creating this standardized rural style is more reasonable, or should they have preserved the original rural environment?

A: JM: I feel the current Hui-style feeling is quite alright, very nice. Gives people a very comfortable feeling.

Q: Your family's shop actually represents a capital investment, right?

A: JM: There was investment coming in. Actually, it changed the rural environment, including the basic material conditions. Right, because originally there was only this place. All those things were built by us. And originally, when our boss came here to develop, he hauled away over 20 truckloads of household garbage. Originally this place was all household garbage.

Q: They used to pile garbage here, right?

A: JM: Not that they... these places, they didn't really maintain them. Because we wanted to recreate a good environment here, so the soil and household garbage, we entirely hired... removed over 20 truckloads. So I think it's much better now than originally. And originally, the farmer residents, anyway, made their own land very dirty. It wasn't like now, after this renovation, the environment has become so good. Originally it was all household garbage, lots of it, thrown everywhere. But now we've changed it very well. Look, it's relatively clean and sanitary.

JM: Meaning, you've also done a lot of good deeds here, should be considered as contributing a lot for business, and also made many improvements to the village appearance. Our shop... our boss invested over 2 million to improve this place, that's why it's like now. Originally the vegetable garden up there and everything was all household garbage, all messy stuff. We entirely changed it, renovated it. Now it's our own vegetable garden. So it was all cleaned up and organized.

Q: What impact do you think tourism has brought here?

A: JM: This year, the pandemic... but if it weren't for the restrictions, tourism, relying on Niushou Mountain, would be pretty good. Because this is the West Gate entrance, it's still good. Tourism can drive the local economy, it's acceptable.

Q: Did you come here because the village committee contacted you? Or was it local management personnel who contacted you?

Our shop, our boss?

Q: Yes.

A: JM: Our boss... seems like it should have been during investment promotion, or our boss came here for a meal, went down to eat, saw this place and decided to make it a place for his own... cultivation of sentiment. Because there are several partners, their cultural awareness is quite strong. So they thought this place could be away from the city's noise, and the air is fresh here. Afterwards, he wanted to cultivate his sentiment. The several partners originally built this place to drink tea and chat with friends. Originally we had accommodation here, you could stay. But later felt that cultivating sentiment was quite stressful, because creating all these things was quite difficult.
