# Supplementary material for: Rurality representation and changes in rural tourism destination
Source: PLoS One. 2026 Apr 21;21(4):e0347226. doi: 10.1371/journal.pone.0347226 (PMC13098982; doi:10.1371/journal.pone.0347226)
Supplement: S1 File — (ZIP) [file pone.0347226.s001.zip › supporting information/世凹村录音及转译文本/jsa14.m4a.doc]

[File Name]: jsa14.m4a-Transcript Result.doc

Q: I'd like to ask, what changes do you feel the development of the last couple of years has brought to the village?

A: JM: The environment is better, the environment is tidier. Their income from running agritainment businesses is higher, their quality of life is higher. It's like this.

Q: And what about the neighborly relationships among villagers? Any changes?

A: JM: Neighbor relations... well, the neighbors have mostly moved away, basically all gone, relocated due to demolition. The people here now are all outsiders who came to do business. We basically don't really know each other. So, neighborly relations are definitely not as good as before. That's why many people moved away. Now the expropriated houses are all rented out to others. Many are like that upstairs. They moved into residential compounds in the town.

Q: Actually, you mentioned tourism here. What element do you think represents the rural village?

A: JM: It's the agritainment businesses.

Q: It mainly relies on agritainment to attract you. Do you have an ideal vision of what a rural village should be like in your mind?

A: JM: Food, drink, fun, entertainment... like eating a meal, accommodation, all-in-one. Feels too monotonous here.

The business formats are still too homogeneous.

Q: Apart from the relocation of residents, are there any other changes? For example, our transportation methods?

A: JM: Originally, this village had no bus service. Now there's a public bus. Every family has a car.

JM: The economic conditions here are quite good. Every family has one, but it's definitely all quite good.

Q: The Zheng He's Tomb here has always existed, right?

A: JM: Zheng He's Tomb is old, it's been there for ages, has always existed. Zheng He's Tomb has been there since the 1950s. I didn't know before that either, but anyway, it has always been here, passed down here all along.

JM: Zheng He Lake was created when they developed the Niushou Mountain Scenic Area. Before it had no name, before it was just called the reservoir. Before it didn't have this name, before it was just called the reservoir. Later, when they developed the Niushou Mountain Scenic Area, they called it Zheng He Lake.

Q: And the legends about Yue Fei have always existed?

A: JM: Yue Fei stationed troops here. It has always existed. Also, his old trenches and ruins are up there, have always been there.

Q: Your village has always passed these stories down.

I heard your village has descendants of Tang Xianzu?

A: JM: We are all surnamed Tang. Our ancestors, according to the clan genealogy, said they moved from Southern Anhui, that area. You still have a clan genealogy, a sense of family? For example, back then there probably wasn't much... is there still an ancestral hall?

JM: No, that kind of ancestral hall, that kind doesn't exist. Now it's agritainment and such, no.

Q: In the last couple of years, what changes have you noticed in rural moral customs?

A: JM: The residents' quality... is getting higher and higher. Just getting higher and higher.

Q: Do you prefer living in the countryside or would you consider the city? How do you feel about your rural identity?

A: JM: If I think about it, when I'm old later, I still want to come back here to live.

Quite identified with the countryside.

JM: Now I work in the town, live over there, in Dongshan Town where I am, it's more convenient there. When I'm old later, I might come back, still play mahjong.

JM: Actually still quite like it, even though you probably work over there.

Q: Over the years, the changes are mainly in the natural aspect, right? And the space for social interaction has probably been somewhat compressed? Originally, people might have chatted initially, now they look at their phones, watch TV, that kind of thing. Probably there's less face-to-face time now, maybe looking at phones more, right.

A: JM: Originally, we would also farm a bit of land, grow some vegetables. Now the fields have all been expropriated, basically no fields left.
