# Supplementary material for: Rurality representation and changes in rural tourism destination
Source: PLoS One. 2026 Apr 21;21(4):e0347226. doi: 10.1371/journal.pone.0347226 (PMC13098982; doi:10.1371/journal.pone.0347226)
Supplement: S1 File — (ZIP) [file pone.0347226.s001.zip › supporting information/世凹村录音及转译文本/jsa15.m4a.doc]

Q: What changes have you noticed in rural life over the past couple of years?

A: Life is better.

Q: Anything else?

A: JM: And a strong sense of happiness.

Q: Getting happier, right? The countryside is getting better, isn't it?

Q: What was the countryside like in your memory?

A: JM: Now, first of all, there were mud roads, inconvenient and not good. Every household had pigs... (mentions 'Jane Eyre' likely as a metaphor for animals/pests) crawling underneath, stank terribly. Dirty, messy, poor. Now, as they say, it's like 'birds singing and flowers fragrant', a 'Shangri-La'. The environment is very good now.

Q: You mentioned this place is developing rural tourism. What elements do you think best represent the countryside here? What can't represent it, meaning...

A: JM: The elements that best represent the countryside are growing your own vegetables. Organic vegetables. And doesn't every household have peach trees? They all have fruit trees.

Q: Are there characteristics that differentiate rural residents from city dwellers, things that truly represent rural features?

A: JM: Now it seems like there's not much difference from the city. They still dance square dance, right? Now, at the very least, workers like us have pensions, right? Old folks can now get over 1000 yuan, right? Life is basically quite affluent. Culturally, it's good now too. If you have the time, the Elderly University is also very good. The Elderly University has Go, singing, chess... whatever you want to learn, it's available. This kind of leisure life is now much more diverse and rich. Compared to before, when it was just farming, and your whole mind was on farming and earning money. Now, while earning money, cultural life has also enriched.

Q: Have there been any changes in neighborly relations among residents in the last couple of years?

A: JM: Neighborly relations are all quite good, have always been good. But back then, we didn't have time to chat? Back then, you could hold your bowl and go to their house to eat, sit at their table and eat. 'My dish is here', then come to my house to eat. Now that's impossible. This point has become somewhat distant. There is a bit of distance. Because everyone is busy with their own things now, everyone runs agritainment, right? Not like before, really, you hold your bowl and come to our place, 'what dish does your family have? Our family's dish is here, I'll come over', come to your house to take a couple bites of food. That's impossible now. Now we're busy to death. Right. Because communication is less, that's telling the truth.

JM: (Adds about phone/TV use impacting interaction, and personal anecdote about binge-watching TV series affecting eyesight, specific details about shows and episodes).

Q: (Likely prompted about household appliances) Originally there might have been fewer, but now they are all equipped?

A: JM: Don't watch TV, just look at the phone.

Q: Any changes in buildings? Originally single-story, now maybe three stories? Are they two or three stories now?

A: JM: Where? Single-story, still single-story. No upstairs.

Q: So your house wasn't rebuilt, presumably. They just added the horse-head gable, right? The government helped you install the horse-head gable, right?

A: Right.

Q: Was the pace of life originally slower? After you started the agritainment business, did it become busier and more exhausting?

A: JM: No more early to bed, early to rise. I get up early, but I see people drinking until midnight, you also have to wait, then can sleep. Late to bed, early to rise. Too tired. Doing this now is very tiring, it's really very tiring. You see, we close whenever the customers leave. Their meal times might also be later; for our lunch, it's usually around 3 or 4 PM.
