# Supplementary material for: Rurality representation and changes in rural tourism destination
Source: PLoS One. 2026 Apr 21;21(4):e0347226. doi: 10.1371/journal.pone.0347226 (PMC13098982; doi:10.1371/journal.pone.0347226)
Supplement: S1 File — (ZIP) [file pone.0347226.s001.zip › supporting information/世凹村录音及转译文本/jsa16.m4a.doc]

Q: What changes have occurred after the development of rural tourism?

A: JM: The changes are quite significant.

Q: What kind of changes?

A: JM: Everything has been developed and renovated. It wasn't like this originally. Before, the scenery wasn't this good, the transportation wasn't this good, the houses weren't this pretty. It's all been renovated and developed, meaning the physical environment, including roads and everything, has been upgraded.

Q: What was it like originally?

A: JM: Originally, there's a display picture here... originally it was just ordinary buildings like common people built, no horse-head gables, no road in the middle, no road in the front. This square was all farmland, you know? It was all rural. Including the front Taoyuan area was all farmland. Then it was developed in 2011, officially developed and opened in 2012. The backbone entered... originally the village was quite poor, poor and backward, like a small, poor mountain gully. After the renovation, it became incredibly good. Now the environment is good, look, even the air is good.

JM: (Responding to a question about elements representing the countryside) You ask me, I don't know the meaning... your meaning is representing the good aspects of the countryside, something representing the countryside. For example, flowers, plants, trees, mountains, water... probably elements relatively scarce in the city, right? Because actually, cities don't have so many trees... so it creates our environment, this oxygen content is relatively high, because we have these mountains, water, forests, fields, lakes, right? It's different from the city.

From a behavioral perspective, our rural lifestyle habits are definitely different from the city. Because the city requires daily work, our countryside is relatively slower. Culturally, the city might be centered on economic interests, while the countryside is still more harmonious, focusing more on harmonious interaction among villagers, involving less economic interest between them.

Q: So, what elements do you think best represent the countryside?

A: JM: I think they are quite good too. Because every family runs agritainment, and each household is running agritainment, so their income is quite good, their standard of living and quality of life are quite good. So good, I feel it's quite nice.

Q: Does your family participate in agritainment?

A: Our family doesn't.

JM: We all live in this area. We work in sanitation on the street, doing environmental cleaning.

Q: You described the past countryside: farmland, dirt roads, single-story houses. Now it's changed to three or two-story houses, right? And every household's doorstep is cemented.

A: JM: All developed and renovated. Before, this place was no good. Transportation wasn't this good either.

Q: What has transportation development brought here?

A: JM: It brought huge changes.

Not just tourists, but also your residents travel much more conveniently.

JM: Before, transportation was inconvenient, travel was inconvenient. Now it's well-connected everywhere, you can reach anywhere.

Q: Information reception? You probably receive more information now, right? Mobile phones provide more, faster information. Financially, many residents spontaneously built agritainment, so residents' own funds actually changed the village's external environment and appearance, right?

A: JM: Developing agritainment... they all spent their own money. The first year of renovation, there was definitely government support. Things like the external horse-head gables were done by the government, not related to them, not their own doing. They just used their own houses to open agritainment, that's all.

Q: So the government and individual families co-invested to build the basic village structure?

A: JM: At the very beginning of the development, things like painting the outside walls white, and security windows, were done by the government initially.

Q: Do you still raise poultry here, do you know?

A: JM: Now it's rare. In this village, one or two families raise some, others don't. Originally, everyone raised them. Now very few raise them, and it's also not really allowed. That's a big change.

Q: Rural changes include building roads and a square. The resident square... seems used less by residents, mainly for tourists to play, right?

A: Tourists come here to play, to rest, sit under the pavilion.

Q: As a rural resident, has your pace of life changed? For example, what was your life like before 2000?

A: JM: Now it's definitely better.

Q: What was it like originally for comparison?

A: JM: Originally people were definitely poorer. Now conditions are definitely better, I tell you. For example, before 2000, ordinary folks like us rarely bought vegetables to eat. You had no money to buy vegetables, right? Look how much life has improved now. Buying vegetables... originally you might grow your own, like preserved vegetables. Before, you rarely had money to buy, unless guests came, then you'd buy some meat dishes. Now it's different. Now every family wants meat in every meal.

Q: What about shopping?

A: JM: Originally, shopping was very infrequent, even less. Now it's much better.

JM: Now much better. Originally, eating out was impossible. Now it's normal.

Q: Now, household appliances should be quite complete?

A: JM: Now every family is like this. Originally, people might just have a TV. Before, at most a small TV. Before 2000, no air conditioning. Now the changes are different.

Q: Transportation? Did families have cars originally?

A: JM: Around 2000, no cars, just small bicycles. Having a bicycle was very good back then. Just bicycles, nothing else.

Q: Where was the social interaction space originally? Where did villagers communicate?

A: JM: Back then, working all day, where was there time to communicate? Everyone was farming the land. Now nobody farms. There's time now. Back then, farming the land, where was the time to communicate? Work until evening, exhausted, just sleep. No time back then. Now it's really different from then.

Q: How were neighborly relations?

A: JM: Originally, it was quite good. Now we live in residential compounds. Anyway, no conflicts. Everyone just closes their door after entering home, no contact, don't do anything together.

Q: You live in apartment units now? In Guli Street?

A: JM: Have you been? We came by taxi.

JM: We live in a compound. We moved out to the compound in 2005. So now, contact with neighbors... very little contact. Individuals enter home, close the door, that's it. Don't play together or do anything. At most go downstairs to play or something.

Q: So there's not much neighborly relationship now, right?

A: JM: Because once you enter, door closed, individual enters home and closes the door. I don't know you, you don't visit anymore. Not like before in the countryside before demolition, when you'd run over to my house, next-door neighbors. Now that doesn't exist.

Q: Have neighborly relations become more distant?

A: JM: Not exactly distant, still good. But it's not like before, playing together every day. For example, eating, just holding a rice bowl we could be together, next-door neighbors. Right, now it's different, the nature is different.

Q: Do you prefer the countryside or the city?

A: JM: I think... how to say... the countryside air is good. The city... definitely each has its benefits.

Q: Do you identify as a city resident now? Or are we still rural?

A: We are like relocated households. Don't farm anymore, were resettled. Anyway, that's how it is. Land is gone too.

Then comparing like this, of course living in a compound is better, because there are fewer mosquitoes in summer, much fewer. Anyway, don't farm the land, just work, come home from work, cook, wash up, nothing else.

Q: From your talk, I feel you still somewhat identify with the resident identity, the urban identity.

A: JM: Anyway, I don't know... you see, I'm confused. You actually think each has its good points, how to say...

JM: My personal feeling is that the difference between countryside and city now isn't as big as before. Like before, the difference between city and countryside was huge. Now, like us rural people relocated, some villagers got five or six apartments, plus money. We relocated early, in 2003. We didn't get much then. Now people get more from relocation.

Because we are in Jiangning District, but close to the center. If it were further away, it might be different.

Maybe our community would be far and inconvenient, or maybe need to migrate far for work, right?

JM: Like people here, they basically work locally, don't go far.

Q: Were there any festive activities originally?

A: JM: Originally no. Now in the evening, we don't come to this place. In our compound in the evening, many people dance square dance. One group here, one group there, full. Outside the compound, one group here, one group there, everywhere. Definitely a big change compared to before. Now it's quite common, many square dance groups. Originally there were none. Before, say 2007, where were there any?

Q: Have the procedures for weddings and funerals been simplified?

A: This year seems like haven't seen any... who knows recent years... these recent years compared to before, definitely more elaborate now, even more elaborate. Before, rural people got married, brought the bride home, held it at home. Now at home? No, all in restaurants, hire an emcee, do this and that... definitely different.

Seems quite cumbersome too, needing what? Picking an auspicious date, many steps. Although we originally in the countryside seemed... relatively poor, but the rules... you mean the rules.

Before the rules were bigger, now the rules are smaller. Now people don't care so much, just pick a good day. Before people would check, calculate... now no, this aspect is gone. Seems now people focus on convenience. For example, pick a Sunday. Now people pick a hotel, whichever day you have time, like a Tuesday, pick a date, whenever you have time. Seems like it's all like that now. The rules are indeed fewer.

Q: Are there still religious sacrifices here? Like Qingming Festival, you still worship ancestors, right?

A: JM: Ancestor worship... this year we didn't. The pandemic... no, that's not right, the pandemic made everything simpler? That's impossible.

Q: Are there any religious activities?

A: JM: Seems not very familiar. Generally, not really.

Q: Originally, your village probably had a village chief. Now it should be a residents' committee, right?

A: JM: Before it was the team leader. The team leader led people to work. Before it was all like that. Anyway, rarely contacted you so much, so many questions. Contact when there's business, no contact when there's none. Generally, no contact now. Now you have no business, relocated, don't work the land, no land, nothing left. Before... handing over grain tax, then state taxes, agricultural tax. Now none, fewer matters.

Q: Compared to the city, have there been changes in rural morality or values in recent years?

A: JM: Compared to before, definitely big changes. How comfortable people are now! Not like back then farming, so exhausting, you know? Exhausting. This time of year... was daily planting rice in muddy fields, planting until your back and legs ache, unbearable.

Q: Morality-wise, originally... maybe there's a good change?

A: JM: Now morality... compared to before, it's better. People's quality is higher now. Not like before, in the countryside, arguing and fighting over a bundle of firewood on the field ridge. Now people are better, they don't want such things. Much better than before.

Q: Those myths and legends, including Zheng He, Yue Fei, these things existed originally?

A: JM: Before, we weren't very familiar with this place, but we learned while working here that this place has them. They existed before, always had these existing sites. Because Zheng He's Tomb always had people visiting. Before this place was renovated, people already went there to visit.

Many Nanjing people know about Zheng He's Tomb. These spiritual cultures have been passed down in this place all along.

Q: Can I ask for your basic information? Age?

A: JM: 51.

Q: Education level? Primary, Junior High, Senior High?

A: JM: Primary school. Didn't even finish primary school.

Q: How many years have you lived here? Counting Guli.

A: JM: We were probably born in Guli, birthplace is Guli.

Q: Your family doesn't participate in tourism, I assume. Your income level?

A: JM: We aren't involved. Our annual household income... long time... we sweep floors, earn just over 2000 yuan a month. No one asks me... if you count the whole family together, right?

Combined, not 50,000-100,000?

I JM... we just get over 2000 a month. Look, 50,000 yuan... roughly over 50,000.

Q: Okay. What do you think best represents the elements of the countryside? The pace of life, the simplicity of rural people, or the natural environment?

A: JM: I think the natural environment. The natural environment. The mountains, water, forests, fields, lakes... these natural environments are what you can feel more distinctly.

Q: Originally, in the past, what represented the countryside?

A: JM: The past... the past was just farming, farming the land, agriculture-based. That was the people then.

Q: Now, what represents the countryside?

A: JM: Now representing the countryside... in my opinion, good air, the environment.
