# Supplementary material for: Rurality representation and changes in rural tourism destination
Source: PLoS One. 2026 Apr 21;21(4):e0347226. doi: 10.1371/journal.pone.0347226 (PMC13098982; doi:10.1371/journal.pone.0347226)
Supplement: S1 File — (ZIP) [file pone.0347226.s001.zip › supporting information/世凹村录音及转译文本/YSA1.doc]

Q: What should the countryside be like? In the text above, it suggests the countryside should have flowers and grass, be developed like this... Do you agree with this view?

A: The scenery should be better, the scenery should be nicer, the air should be very fresh. It can basically achieve that.

Q: You mentioned this place, Shi'ao Taoyuan, is a 'Most Beautiful Village' and is done quite well. Do you think there are any areas that need improvement here?

A: We basically... learn very little from what's above (?). I'm not a local, right? Did you come here by applying for a job, or...?

Q: What do you think are the elements that best represent the countryside? Compared to the city. The things that represent how it's different from the city.

A: Having lived this long... in the city... the countryside definitely has more beautiful scenery, better air, a better environment. Nowadays, what people demand is the environment, right? Air pollution, right?

Q: But is there anything else? What do you think about the countryside?

A: Regarding what's above... I haven't been up there. I basically don't go up there either.

Q: Okay, thank you. Also, friends, welcome to our... (text cuts off)
