# Supplementary material for: Rurality representation and changes in rural tourism destination
Source: PLoS One. 2026 Apr 21;21(4):e0347226. doi: 10.1371/journal.pone.0347226 (PMC13098982; doi:10.1371/journal.pone.0347226)
Supplement: S1 File — (ZIP) [file pone.0347226.s001.zip › supporting information/世凹村录音及转译文本/ysa10.m4a.doc]

Q: Regarding rural construction, most families are involved. What changes do you think the 'Beautiful Countryside' construction has brought?

YK: The roads are easier to travel.

Q: Specifically for Shi'ao Village, what are your feelings?

YK: Shi'ao Village... I feel there's nothing much to do here. It's probably meant to serve Niushou Mountain, right?

Q: Yes, it's considered a supporting facility for Niushou Mountain, as there are fewer dining options up there.

YK: So, they built roads and have more agritourism , but inherently, if you want to play, there's nothing to play.

YK: From a tourism perspective, the visitable elements are even fewer. There's very little scenery. It's all agritourism. Yes, anyway, after walking around, there's simply no scenery to speak of.

Q: Yes. What was the countryside like in your memory?

YK: The countryside in memory had those earthen houses, those chickens, ducks, dogs, and so on, and some farmland, things like that. Now it's basically these small agritourism houses, these uniformly planned houses. It's clean and tidy now, one should say. Two or three-story small foreign-style buildings, and the roads are easier to travel. If it's uniformly planned, it's like lavender gardens, sunflowers, rapeseed flowers – that kind of thing is more common.

YK: But this place, it's mainly an agritourism village supporting area. Its positioning is low, it's not suitable for installing those things. For example, this village originally didn't have much land. Look at its original remote sensing image, there are just these two plots of land inside, this is land. So they don't grow things, they can only convert buildings. It's a village on the mountain, so it also has no land.

YK: So it's just about eating. If they want to develop this village further, it's about creating more spots where people can take photos, and places to sit down and rest.

YK: These landscapes need to be a bit more, just a bit more is enough. You should be able to stay for two hours. Come in the morning, stay for two hours, then have lunch here in the afternoon, and leave – that could be quite nice.

Q: You mentioned this place is developing rural tourism. What do you think is the element that most represents the countryside? What is the thing that most represents them?

YK: Representing the countryside... each countryside, I think each village and town has its own characteristics. This is hard to say.

Q: For example, from the perspective of the physical landscape, what should it be like?

YK: I think it should be the scenery. The scenery is very beautiful. Yes, there must be open space, chickens running all over the ground, and the air should be better, the air should be fresh. Unlike in the city where it's all buildings and such. The air here is still pretty good.

YK: I think their... this activity could be developed further, give people something to see. There is a lakeside path; they could do something with the lakeside path, then expand it a bit, build some small bridges, so you can actually walk around. You can't just look, see it and it's over, there's nothing to look at. Where there is water, plant some lotus flowers. There are peach blossoms in front. After the peach blossoms are gone, are there other scenes to keep people here? That would be best.

YK: Like our peach blossoms... you can't just have people come only when the peach blossoms are blooming, and then afterwards... The local residents here have the habit of 'Spring Niushou' outings here. Yes. But of course, this spring coincided with the pandemic, nobody came out for other reasons.

Q: What about the rural residents? Do they have any representative... perhaps compared to city people...

YK: You think it's mainly the material level, like rice paddies, pastoral scenery? There would have been more before, now it's less. Was it more natural before?

YK: Now the modern atmosphere is stronger.

Q: What about domestic waste? Do you feel any changes?

YK: Waste sorting. Nanjing is implementing waste sorting.

Q: Do you feel any changes in the lifestyle habits of the rural residents here?

YK: Didn't know before. Their... haven't understood their lifestyle habits.

Q: What were the transportation methods in the countryside originally?

YK: Originally it was tractors. Now it's all cars.

Q: Did you feel that villagers originally had a sense of identity with the countryside?

YK: Originally? I feel they are much more civilized now. I think everyone's civility is getting better and better. The modern atmosphere is so strong, and they've started waste sorting too. It's not like before when they were uncivilized, spitting everywhere and such. It's definitely much better. But you can still feel... you can still see dog poop and things.

Q: (Implied: Is cleaning outsourced?)

YK: It's all outsourced. It should be that people come regularly to handle it.

Q: What about the historical figures of this countryside? Do you know any?

YK: Basically don't know.
