# Supplementary material for: Rurality representation and changes in rural tourism destination
Source: PLoS One. 2026 Apr 21;21(4):e0347226. doi: 10.1371/journal.pone.0347226 (PMC13098982; doi:10.1371/journal.pone.0347226)
Supplement: S1 File — (ZIP) [file pone.0347226.s001.zip › supporting information/世凹村录音及转译文本/ysa12.doc]

Q: What do you think defines the characteristics of the countryside?

YK: The countryside... the air is very good. The people are good.

YK: The countryside is good, it's just that you can't stay for three or four days and then it's no good. If you eat delicacies every day, you'll start wanting salted fish and radish. People want to come and adjust. If they are in the big city every day, they think about coming to the countryside to experience it. But if they were in the countryside for a long time, they wouldn't be willing to stay.

Q: So what attracts them then?

YK: I just like digging for wild vegetables.

Q: What is it that attracts people to the countryside?

YK: For some, it's the scenery. For some, it's wild game, wild vegetables. For some, it's everyone gathering together, breathing the fresh air, classmates or colleagues getting together here for a gathering. Some people find the city worse than the countryside; they come here for the peace and quiet. Country folks like to go into the city, city folks like the countryside.

The countryside has its good points, the city has its good points.

YK: Roughly, the city is 70% good, the countryside 30%.

Q: So you have a preference for the city.

YK: If it's for health preservation, then it's the countryside, because the air is good.

Q: In your impression, what was the countryside like? Do you have a fixed image of the countryside?

YK: Looking at the development in recent years, it's still good.

YK: It's quite good. But in the past? When we were young, the past was too poor. In the past, we didn't even have enough to eat. Living conditions were very hard. You guys don't know, you haven't seen it. In the past, they were all thatched houses, almost no multi-story buildings, no tile-roofed houses. All were thatched houses, houses built with earth and straw. That was just 30 years ago. And no food, no drink... pitiful. If you were sick, you just endured it yourself. Now it's okay. Now rural development, everywhere is the same as anywhere else in the country.

Q: Yes. Life in the countryside used to be quite hard.

YK: Not just hard, incredibly hard. Even eating was a problem. Digging for wild vegetables. When times were poor, people even ate tree bark, elm bark, locust tree flowers. Just thirty-some years ago... what era... you don't understand. Those of us who lived through that era, then slowly developed, after the reform and opening up, developed gradually. Now the countryside has better conditions, definitely better. Every rural household has electricity and water. They've built multi-story houses. People go out to work as migrant labor. Economically and in all aspects, it's acceptable. Small villas... And also now, for example, our Nanjing city used to have just four or five million people. Now, including outsiders coming to buy houses and settle in the big city, it's over 10 million people.

Q: What do you think are the elements that most represent the countryside?

YK: The elements that most represent the countryside... well, the air is fresh, the environment is relatively quiet. On the material side, in the countryside, you can say the food and drink, the vegetables, everything is green food, from their own homes. Country people usually grow their own. When they farm, they use chemical fertilizer and stuff, GMO things... they sell that to others to eat. The food they eat themselves, they don't use those things. They usually use their own urea... pure natural.

YK: That stuff is pure natural, healthier, more natural.

Q: Nowadays, compared to the city, do you think the countryside has developed any negative aspects? For example, maybe their values before were a certain way...

YK: There's nothing bad. The biggest difference compared to city people is in the area of pensions and medical insurance. And their pensions are too small, just a few hundred RMB, some even just seven or eight hundred, a thousand RMB. In the city, generally... they have their advantages.

YK: The countryside has pure natural things, right? Away from the hustle and bustle, away from all that... mainly transportation costs more... but every place is developed now, don't mention that. Now transportation is developed everywhere, I tell you, right? Not just the countryside, anywhere, transportation is developed. They could manage it even before. The good thing about the countryside is the good air, natural, quiet, away from the hustle and bustle. The good thing about the city is, like for retirement, elderly care... but they don't guard against dust... Don't think... if a rural person gets sick and goes to the hospital, how much do they have to pay themselves? When I was in the hospital, rural people cry over thirty or forty thousand RMB. Rural insurance covers less after all, only 20%-30%. In the city, houses are small. In the countryside, houses are big, live more comfortably.

Q: Were villagers in the past relatively more honest and simple? Were interpersonal relationships better than now?

YK: Interpersonal relationships... rural people in the past were very, very good. Yes, they helped each other. For example, during the busy farming season, they helped each other. The poor helped the poor. Now it's not like that. Now it's about keeping up with the Joneses. After the reform and opening up, it's all about comparison. Now your family compares with mine. Your family builds a multi-story house, my family must too. Your family buys a car, my family must too. The past was very good, but the past was very poor. So poor, let me tell you, we were so poor we didn't even have clothes to wear. So poor. When we were kids, we wore clothes with patches on top of patches, all patches. Having a pair of... what, Huanyang shoes? Or cloth shoes to wear was a big deal. Buying a bicycle, Fenghuang, Yongjiu, these bicycles cost one or two years' income, couldn't afford them. But people in the past lived happily.

Q: What do you think the ideal countryside should be like?

YK: What ideal countryside? There is no ideal countryside. The ideal countryside would be with birds singing and flowers fragrant, fields green and lush, and family harmony. But how is that possible? It's pitiful now. Rural elderly are pitiful. Now the children have all gone out to work. The old folks are stuck at home. It's not like before when, even though poor, everyone was together, the whole family together eating and drinking, enjoying family happiness.

YK: Now what? Now the elderly, generally, some people work outside, don't return home for two or three years. The elderly are pitiful, just stuck at home waiting for the children. The children come back, maybe just ten-some days a year, then leave again. In the countryside, there's still the issue of children leaving their parents.

YK: The countryside now is an aging society. The elderly have it too hard. The middle-aged have all gone out to work. If you're working, the elderly suffer. Even though we were poor before, everyone was together, enjoying family happiness. The son farmed, the daughter-in-law grew vegetables. In the evening, everyone was together, could play cards, chat. Now that's gone. Now it's rare, only during New Year or holidays.

YK: Yes, now in people's hearts, it's all about the economy. Now other things, like feelings or anything, are all fake. Once you talk about the economy, that's everything. Not like the past.

YK: Reform and opening up brought in good things, but also brought in bad things. Long ago, there was no pornography, gambling, drugs. Now look, there's a whole bunch of that. Also, long ago people didn't have major illnesses. Now, what, novel coronavirus, SARS, look. These are man-made... what, food additives, all sorts of things. In the end, our country... not as good as before. Now this food pollution is terrible.
