# Supplementary material for: Rurality representation and changes in rural tourism destination
Source: PLoS One. 2026 Apr 21;21(4):e0347226. doi: 10.1371/journal.pone.0347226 (PMC13098982; doi:10.1371/journal.pone.0347226)
Supplement: S1 File — (ZIP) [file pone.0347226.s001.zip › supporting information/世凹村录音及转译文本/ysa2-20200625.doc]

Q: Do you often go for rural tourism?

YK: I don't go very often, maybe about two or three times a year.

Q: What do you think is the biggest attraction of rural tourism for you?

YK: Speaking of tourism, the most attractive aspects here are, firstly, the better environment. After being in the city for a long time, it gets quite frustrating, so you just want to get out and walk around. The countryside happily satisfies this – you can take walks here, there's rural cuisine. Some even better rural places also have picking experience activities, which is even better. Picking strawberries when they're ripe, picking peaches when they're ripe... seems like we've already missed the peach season now.

Q: Do you think the countryside here is different from other rural areas you've visited before?

YK: This countryside area, actually, compared to others... I think their biggest characteristic is actually that they are quite similar, quite standardized. The architecture... like many rural tourism spots around Nanjing, they are basically all this model. Probably because Jiangsu uniformly promotes 'Beautiful Countryside'. The architecture is all standardized. Then they create some plant landscapes. And the plant landscapes here aren't like some rural spots that might have some original ecology; the landscapes here are all artificially created later,leaning towards urban garden-style landscapes. If I must name one characteristic, it might be the food here. I understand that here, it seems every household has been selected for one specialty dish. This should be considered their unique feature. It might also attract some people to come back regularly at different times to try each family's cuisine.

Q: What is your ideal countryside like?

YK: My ideal countryside... preferably, the houses shouldn't be so uniform and identical. They should be picturesquely scattered . Then, those country paths crisscrossing fields ... where you can take walks in the fields. It seems this place doesn't have that feeling. Here, it's quite different from my ideal countryside.

Q: You said that your ideal countryside... is it suitable for developing tourism?

YK: Suitable? Yes, it's also suitable for developing tourism, right?

Q: What impacts do you think tourism brings to the countryside?

YK: I think it's a... Looking at China's rapid urbanization in recent years, many villages are facing decline. I think a positive promoting effect is that with tourists coming, it can help preserve some of the countryside's characteristic elements. Like, we also know that in many villages, some traditional crafts can thus be passed down. Like before, maybe things like oil pressing, or tea making... these things maybe young people don't do anymore now. But because some tourists want to experience it, or want to buy, or take children to see it... these farming activities or crafts, or rural activities... can all be preserved, that's one aspect. But on the other hand, tourism also brings negative impacts. Especially some very popular rural tourism spots, they can damage the original folk atmosphere of the village. This is a very important point for people who come... like many say that having few people is a characteristic. So tourism has these two sides. On one hand, it helps preserve some farming activities, rural memories, and rural cuisine gets new development and is retained. On the other hand, it also brings some environmental pollution and damage. Plus, in our country, due to the needs of urban residents, there's also increased effort to improve and build 'Beautiful Countryside'. This also improves the living standards of rural residents.

Q: Do you think the countryside has changed through tourism?

(Note: The question seems cut off, but YK continues his point about impacts.)

Q: The roads in the countryside, including its infrastructure, compared to other rural areas you've visited, which is better?

YK: Actually, the roads here in the countryside... they are fully hardened/paved. Well. Actually, some rural spots might originally have brick-paved roads or flagstone paths. If they are preserved, I feel that kind of rural atmosphere is even stronger . But this kind of countryside, whether the road entering the village is asphalt, or the internal roads are all cement... this aspect I think is not done well. This point reduces the feeling and atmosphere of the countryside.

Q: Have you experienced any local folk customs here, or had any in-depth communication with local residents?

YK: Here, it seems not really. There seems to be an orchard over there, but no picking activities either. Other rural experiences also feel lacking. There aren't any specific... also didn't see any introductions, like specially having a museum or exhibition. And there's no one to guide you to specific rural experiences. Nothing like that.

Q: For example, regarding some aspects you don't like, what is your favorite rural element?

YK: My favorite rural element... I think maybe one is the food. More material...

Q: Is rural culture or folk customs an attractive aspect for you?

YK: This aspect I think is also an attractive factor. But how to say... As a tourist coming here to play, it's actually very hard to truly understand its folk customs. Unless you establish a very... with the local residents, or with the shop owners, establish a deep friendship, I think maybe then you can deeply understand the local folk customs. Otherwise, it's more of a relationship where they are the shopkeeper, and I am the customer. But you can still feel that the villagers here are very enthusiastic.

Q: Any other questions? Do you like the pace of life here?

YK: Other aspects are okay, quite leisurely. Compared to the city, the difference is still very big and obvious. Yes, seems very obvious. I think another thing... we saw a few lakes, not sure if they could... do something a bit bigger? Right, meaning the experiential rural activities here are too few. This aspect still needs more improvement.
