# Supplementary material for: Rurality representation and changes in rural tourism destination
Source: PLoS One. 2026 Apr 21;21(4):e0347226. doi: 10.1371/journal.pone.0347226 (PMC13098982; doi:10.1371/journal.pone.0347226)
Supplement: S1 File — (ZIP) [file pone.0347226.s001.zip › supporting information/世凹村录音及转译文本/ysa3-20200625.doc]

Q: Do you often come to this rural village?

A: Often... well, what counts as often? About once a month to this place.

Q: What attracts you to rural tourism?

A: It's quite leisurely.

Q: Do you particularly like the rural atmosphere?

A: Yes! The environment is quite comfortable.

Q: Did you ever live in the countryside before?

A: No, we are from Nanjing.

Q: If not, what do you think an ideal rural life should be like?

A: The mood described by Tao Yuanming: 'Plucking chrysanthemums under the eastern hedge, I calmly view the southern hills'. It's more about that kind of artistic conception , a feeling. Very comfortable.

Q: Do you think this place differs from your ideal?

A: It's quite good. This Shi'ao Taoyuan is quite good. We've been here many times, it's always quite good. And there aren't too many people.

Q: What is its biggest characteristic, in your opinion?

A: I think it's the small hills, small waters, small scenes. The whole place is quite compact. And it's leisurely. Also, there aren't too many people. Mainly that there aren't too many people. If there are crowds, well, in Nanjing it's all people, right? When we go out, we don't want to see crowds everywhere.

Q: When did you first come to the countryside?

A: The first time? The earliest time... I can't remember. Not sure when we came. The earliest, right? I can't remember. Anyway, at the very least... you said you came earlier than me. I've been coming for about 5 years.

(Note: The conversation seems to involve multiple speakers here, likely the tourist (A) and a companion (JM))

A (JM?): I think it was developed in 2012?

A (Tourist?): When I first came it was already quite good, already like this. Now it's just cleaner.

Q: Do you see any differences between when you first came and now?

A (Tourist?): It was like this when I came. So no big difference. It has developed steadily. There were actually more people before, now there are fewer. The overall character has been preserved.

Q: Having come here over the years, do you think the tourism development has become more in-depth? Do you think tourism development has had any impact on the countryside?

A: It definitely has an impact on them.

A (JM?): Also, the villagers now live more prosperously.

A (JM?): It affects their income.

Q: Have you noticed changes in the village's infrastructure over this process, like the roads?

A (JM?): It has definitely developed quite well. If it hadn't been developed, the roads everywhere wouldn't be so good. It gets better year by year. Every year you come, you experience better infrastructure. The overall environment is truly getting better and better.

Q: Do you interact much with the local residents when you come here?

A (JM?): We do, but not excessively. We just chat with the proprietress when we eat, and leave after the meal.

Q: What do you think of the local folk customs?

A (JM?): They are quite good. It matches my ideal quite well. I think this place, among the many villages here, is quite good. Yes, this one.

Q: Things like the 'Beautiful Countryside' initiative?

Q: What areas do you think need improvement?

A (JM?): Nothing major. They shouldn't build randomly. Artificial landscapes can反而 (instead) damage the rural character. This place could also sell some local specialties. This place doesn't have many special products.

A (JM?): They don't have farmland anymore. No farmland.

Q: For example, our village is surrounded by hills, trees, water, fields, right? Do you think these hills, waters, or the local animals and plants enhance the rural atmosphere?

A (JM?): Of course, having these things is necessary to reflect the rural atmosphere. If it's the same as the city, how can it be reflected? We come hoping it's different from the city, hoping for hills, water, and fields.

Q: Do you think the representative quality of its hills, water, and fields is strong?

A (JM?): Anyway... compared to other places, it's okay, average. The scenery here I think is average. Average.

Q: What about the air and environment?

A: The air is very good. The environment is also very good. Yes, the environment is good.

A (JM?): The overall rural architecture here is also done quite well. They have renovated it quite nicely. It immediately feels quite strong in rural character. Just like this. Because the education... although they have to deal with the houses and the people... it becomes quite pleasing to the eye.

Q: What about the rural cuisine here?

A (JM?): We like that too. The atmosphere of rural is quite strong. It's different from what we eat in the city. Yes, we want to eat this kind of rural local dishes .

Q: What about agricultural activities here?

A (JM?): We rarely see agricultural activities here. Can't see them. We would like to see agricultural activities, but there isn't that agricultural interaction. It's not very interesting. It's not like... they don't have things like orchards or vegetable picking. None of that. There are some, but they don't belong to them (the village). This orchard used to belong to their village, but actually, I found out it's contracted out. Yes, it's a 'Peach Spring' (Taoyuan), but look, you don't seem to see large stretches of peach trees.
