# Supplementary material for: Rurality representation and changes in rural tourism destination
Source: PLoS One. 2026 Apr 21;21(4):e0347226. doi: 10.1371/journal.pone.0347226 (PMC13098982; doi:10.1371/journal.pone.0347226)
Supplement: S1 File — (ZIP) [file pone.0347226.s001.zip › supporting information/世凹村录音及转译文本/ysa5-20200625.doc]

Q: Uncle, are you a local?

YK: No.

Q: Are you here as a tourist?

YK: We are working here. Migrant workers.

YK: No work on the construction site today, so we came out.

Q: Have you lived in the countryside before? What was rural life like in your opinion?

YK: At our age, if you're talking about how it was before, that's hard to describe... it's a long story. Yes.

Q: Are you familiar with this village?

YK: We just arrived, saw your hotel and came over.

Q: Is this your first time here?

YK: Yes.

Q: What do you think is the main difference between rural life and city life?

YK: Rural life is more open, people can be more casual. City life, right, you step out the door and it's the main street, right?

Q: Do you like the countryside?

YK: At our age, of course we like the idea of rural life.

Q: You think the countryside is suitable for you to relax in, but not suitable for you to live in? Do you agree?

YK: To live... you're just more accustomed to your own environment.

Q: Have you been to any... like now there are many 'Beautiful Countryside' scenic spots around Nanjing, have you visited any of these spots for tourism?

YK: Rarely. Because we are migrant workers.

Q: Have you been to some, like the ones among these rural areas? Because now there are many developing rural tourism. Do you think different 'Beautiful Countryside' spots have different characteristics?

YK: We've been to very few, so we don't know about the differences. It's like this all over the country now, everywhere is doing it. Our hometown in Huaian is also doing it. Some are pretty similar. Of course, some places aren't like this place. This place was originally livable, the topography has mountains and water, so it's developed quite well. In our place, it's a waste of effort and money – it was originally just a plain. Our place is just flat land. They piled up so much earth to make it like small hills. And how much good farmland, land that grew grain, was destroyed... At first, when it was just developed and opened, it looked quite nice. Many people went to see it. But you can't look at it every day; life isn't about going to see it every day. You need to have money to live, and only then go see it. If you can't even eat your fill, would you still go see it, right? Like the one near our place, I went once. It's been over 600 days now, almost two years, I only went that one time, just for a day, and only for a few hours. For me, having seen it once is enough.

Q: What did you think of that place?

YK: It was built... I didn't even think about going again after that one time. I went once and never went again. I mean, like in our Jiangsu Jinhu, we have the Lotus Pond. Have you heard of that place? I went there. To see the lotuses, you have to go in summer, they only bloom around May-June. If you go then, it's unbearably hot. In winter, or on mild days, you could go, but there are no flowers, nothing much to see.

Q: What impact do you think this kind of rural development has had on the local residents in your hometown?

YK: For working people, not much impact. Because you still focus mainly on work, right? Students focus on school, right? For the nearby residents, it's more like having a leisure place. If I have nothing to do, I can run over and take a look, right? You can't, if it's dozens of kilometers away, just drive there today for no reason. You need some economic means to go, right? You can't just start driving there in the morning, rest a bit, drive back, eat breakfast, and then go to work – there are no such constraints. This is just the view of someone our age on this matter.

Q: So, for example, if this village develops tourism, occupies farmland, and changes it into buildings, then the villagers can't farm anymore. What do the villagers get? For example, people here run agritourism businesses; some make money, some don't. What are the villagers in the place you mentioned doing now? What's their situation?

YK: You probably don't understand very well. Very few farmers farm the land now. Even if you mainly farm, let's say your family has a lot of people, 10 mu of land. If you stay home farming all spring, one person normally staying home for a year, after deducting costs, and only if you have a good harvest, you might make over 10,000 RMB, nearly 20,000 RMB. Do you think it's worth having one person stay home to earn this money? So, no one farms anymore. If someone occupies it, they occupy it, right? No one really disputes it. They give a one-time buyout. For example, if my family has 5 mu of land, maybe the compensation is over 20,000... over 100,000 RMB? I get the money in hand, right? I can use that 100,000+ RMB elsewhere. I'm not counting on this money to get rich, right? But when a real famine year comes, and there's no food to eat, then they will know. But maybe you'll never encounter it in your lifetime, or maybe it's right around the corner. As people say now, who knows whether accident or tomorrow comes first, right?

Q: So, looking at what you said, they are now involved in the development...

YK: It's different from what I described. It's different here, after all it's in Nanjing, with lots of people coming and going. Our place is just a small county town, over 200,000 people. Developing this stuff... I'm not criticizing here, I'm criticizing our hometown's situation.

Q: What impact do you think this kind of tourism development brings to the countryside?

YK: Of course, if you don't develop and utilize it, it remains countryside forever, and no one comes to give you... (politics?/investment?).

Q: So after developing tourism, the village's overall appearance improves.

YK: Yes, of course. The human environment definitely changes, improves. And income is the same; because they develop tourism, they might do some business and earn some money.

Q: Do you think this village has encountered any negative impacts after developing tourism?

YK: Because I just arrived at this place, I'm not very familiar with it.

Q: You can talk about the village you mentioned earlier.

YK: The bad aspects... because we don't go to that place often either. We go occasionally, don't know the locals, don't have any interaction, so I don't really know the specifics. But just from my own perspective, at my age, with my viewpoint, is investing so much able to be recouped? But one can only say that this place has such a scenic spot. Yes, that's how it is. But how many people go? It seems besides the initial opening days when people went, it's still relatively deserted now, not many people. Occasionally when there's a holiday, like a major festival, people nearby might go in to play for a while.

Q: Having lived in the countryside for so long, do you have an ideal 'Beautiful Countryside' in your heart? What is it like? A countryside you particularly yearn to live in?

YK: For people our age, this idea is: being able to not worry about food or clothing, receiving a pension every month, that's enough. Nothing more. No illness, that's enough.

Q: Because when we get old and retire, we also need leisure, right?

YK: Leisure... now like we live in the county town, we also have parks, etc. It's a large concentrated area of people. In places with concentrated populations, doing this is necessary. In places with no people, I think it's unnecessary.

Q: So you're saying large county towns build parks, squares, etc., to provide leisure. What about the countryside developing to that stage?

Q: Maybe in the future, you yourself would still want to live in a place with a slightly better living environment?

YK: Yes. But county towns also have their advantages.

Q: Would you be willing to live in a city that is well-developed and has a good environment like this?

YK: Of course, why wouldn't I be willing? But I can't come here.

Q: Uncle, roughly how old are you?

YK: 57.

Q: Your education level?

YK: Junior high school.

Q: Annual income roughly?

YK: Twenty to thirty thousand RMB.
