# Supplementary material for: Rurality representation and changes in rural tourism destination
Source: PLoS One. 2026 Apr 21;21(4):e0347226. doi: 10.1371/journal.pone.0347226 (PMC13098982; doi:10.1371/journal.pone.0347226)
Supplement: S1 File — (ZIP) [file pone.0347226.s001.zip › supporting information/世凹村录音及转译文本/ysa9.m4a .doc]

Q: Why did you choose to come to this place?

YK: It's close. Only about seven or eight kilometers by car.

Q: What is it about this place that attracts you?

YK: It's quite spacious. I can bring the child here to play. The overall environment is quite open, quite suitable for children to play.

Q: You have a child? So it's a short visit, won't stay too long, leave after a while, right? Are you also from nearby?

YK: Yes, from Tiexinqiao . Tiexinqiao is very close.

Q: Do you think the countryside, including this place, has changed much in recent years?

YK: For comparison, I might not be able to tell, because when I started coming, it was already like this now.

Q: Look, this is a remote sensing image from before. It was roughly like this. Originally, it was mainly residential buildings, some dirt roads, and some rice fields. Now, mainly some new agritourism facilities have been built, many new agritourism places, and a new road was built. This is the main change in the physical environment.

Q: What changes have there been over the years, in your view?

YK: On the whole, actually, not just looking at this place, but on the road, nationwide, the countryside has changed quite significantly in recent years. Firstly, the roads are probably better, infrastructure, including supporting facilities like parking lots, agritourism. Then, the overall rural environment has also improved greatly. It's also a supplement to professional scenic spots. Because places like this are for leisure, just bringing children over. There are places with water, to play with fish. The more open spaces are also quite nice. Having mountains and water, a good environment, quite suitable for children to contact nature . It's not too crowded, unlike some scenic spots you go to that might be very crowded and unsafe. This kind of place is good.

Q: What was the countryside like in your memory?

YK: The countryside in memory, probably before, was mainly just countryside, the way rural areas are. Probably didn't have supporting recreational facilities. Basically, it was just the village's own fields, a few fields, then a house. Maybe some also had fish ponds, but those were also private, those fish ponds for farming. Now, they say there are supporting facilities, some agritourism. Before, there were places to eat? Now there are places to eat. Then maybe there are playgrounds, small ones for children to dig in sand. Then the fish ponds might also be opened up, for example, letting you play with fish or eat there, go fishing. There's more to do now. Before, it was basically just a living function.

Q: What do you think is the element that best represents the countryside? First, isn't it about developing rural tourism? What most represents the countryside?

YK: It's the rural cuisine . Yes,. And then activities centered around the agritourism, doing some peripheral things.

Q: What elements represented the countryside in the past? What do you think most represented it?

YK: In the past, it might have been muddy paths, dirt roads, and some irregular fields.

Q: And now?

YK: Now, when mentioning rural tourism, the first thing that comes to mind is probably still centered around agritourism, and then some facilities for children to play, entertainment facilities.

Q: What is your ideal countryside? You must have an ideal picture of the countryside in your mind. What do you think the ideal countryside should be like? Not just including material aspects, but also interpersonal relationships, or the spirit of the countryside.

YK: Firstly, each countryside should maintain its own distinctive characteristics, its local features . Then, centered around these local characteristics, improve the supporting facilities, the basic amenities for eating, drinking, and having fun, make them better around that core.

YK: You feel that basically meets your basic needs. Then you would come to visit.

Q: In recent years, up to now, farmland is still being reduced.

YK: Originally, this place also grew vegetables. Or, your family originally grew vegetables, but doesn't now, right? Originally, there might have been poultry, now it's not allowed.

Q: Have you noticed any changes in the water quality of rivers and lakes?

YK: This area has seen quite noticeable improvement. That is to say, because of tourism development, the basic environmental management has definitely been enhanced to a certain degree.

Q: What about domestic waste? Any changes in recent years?

YK: Yes. Basically, before, in the countryside, people might just throw garbage around their surroundings, or even dump it in the fields. Now, basically, whenever you go to other places, you can still see centralized waste management , including supporting trash bins.

Q: Have there been any changes in rural lifestyle habits? Because they developed rural tourism, have you noticed any changes here?

YK: I don't think the changes are very significant. Not major. Originally, the pace of life might have revolved around how to farm, how to improve one's own... probably the previous sources of income were quite singular, like farming, raising poultry, or operating fish ponds. Now, the sources of income are probably shifting. For rural tourism, probably most of their economy revolves around how to attract tourists.

YK: Yes. So actually, they probably have to pay attention to more and more things.

Q: Do you think their neighborly relations have changed? Can you tell from recent years?

YK: I don't have a very clear sense of that. Neighborly relations mainly depend on the village's own inherent situation.

Q: What about your hometown? Or do you have any personal experience with neighborly relations?

YK: In our hometown, for example, neighborly relations are still quite good. Everyone gets along well as neighbors. If something happens, people help each other. It's still like that now.

Q: Was it better before?

YK: No, it has always been very good. Yes.

Q: In the past, what were the social spaces in the countryside? For example, where did people usually chat, in your opinion?

YK: Probably... now, maybe just find a place under a big tree, an open area, or some main thoroughfares in the village. Now, maybe some villages have a slightly larger activity square for dancing public square dancing . Then they also have fixed places for square dancing. Around the square dancing area, there will be some fixed spots.

YK: Here in Nanjing, maybe people playing cards, for example, playing 'Guàndàn' is quite common.

Q: Any other questions? What about the situation with household appliances? What was it like originally, based on your understanding?

YK: This depends on the time span. If we're talking about around the year 2000, at that time, probably the biggest appliance in a home was the television. Now, basically, every household has the basic set to meet living needs: refrigerators, air conditioners, washing machines.

Q: What was housing like originally?

YK: Before, it was brick and tile houses. The basic condition, right? Maybe slightly better-off families had a two-story building, a three-story building. Mainly now, the characteristics are more distinct. According to their own various ideas, some build rural villas, some... some might not build very high, but it's very distinctive.

Q: Have there been changes in the spiritual aspects of the countryside? For example, anything like religious beliefs?

YK: It should be more diverse than before. Before, exposure was relatively limited. Now there are still various kinds, like Christianity, you remember? Probably a bit more.

Q: Do rural residents identify with their resident status? Do you think they prefer to go to the city, or prefer to stay in the countryside?

YK: I think, looking at it now, and a few years ago, probably everyone identified more with the city. But looking now, there is still a large portion that identifies with the rural identity. Some even have the means to change their household registration , but want to keep the rural .

Q: Religious or sacrificial activities? Have there been any changes recently here? Sacrificial activities?

YK: There were more before. Because before, it was probably more convenient. Now, maybe government control is slightly stricter, including burning paper money, there might be centralized places for that.

Q: Can I ask for your basic information? Your age?

YK: 30.

Q: Education level?

YK: Undergraduate.

Q: Family annual income?

YK: 100,000.
