# Supplementary material for: Rurality representation and changes in rural tourism destination
Source: PLoS One. 2026 Apr 21;21(4):e0347226. doi: 10.1371/journal.pone.0347226 (PMC13098982; doi:10.1371/journal.pone.0347226)
Supplement: Attachment — Submitted filename: Response to Reviewers.docx [file pone.0347226.s003.docx]

Response letter for the Qianda Zhuang, Ph.D. Guest Editor and peer reviewers

Re: Manuscript ID: PONE-D-25-26199

Title: Rurality Representation and Changes in Rural Tourism Destination

Dear Editors and Reviewers,

We would like to express our sincere appreciation to the editors and reviewers for their time and invaluable suggestions, which have significantly enhanced the quality of our paper. In response to all the points raised, we have undertaken comprehensive revisions to the manuscript. Detailed point-by-point responses follow. To clearly indicate the changes, all substantive modifications have been color-highlighted.

We greatly appreciate your time and consideration.

*****reviewer 1*****

**1.**Comment**：The statement on page 4, lines 16 - 17 (“little is known about the content and change of rurality in rural tourism destinations”) should be substantiated and further explained. There appears to be a substantial body of literature on this topic; therefore, clarifying the specific perspective or contribution of the current article would strengthen its position.**

****Reply**: We thank the reviewer for this important observation. Our original statement indeed overstated the research gap by overlooking existing literature. The revision strategy was to acknowledge the established scholarship while precisely defining our contribution through two aspects: first, by recognizing the consensus on rurality’s socially constructed nature while noting limitations in existing approaches; second, by framing our study’s value in providing a multidimensional and evolutionary perspective. Accordingly, we have replaced the original statement with: “While scholarly consensus confirms the socially constructed and thus dynamic nature of rurality perception, existing research remains largely confined to static depictions or unidimensional analyses(Brohman, 1996).A comprehensive empirical understanding of rurality’s multidimensional essence and evolution in the context of rural tourism destinations therefore remains an important scholarly frontier.” This revision maintains our core argument while more accurately positioning our study within the existing literature.**

****2Comment：**Several statements throughout the manuscript require clarification, as they appear ambiguous or potentially affected by issues in English translation. The following examples illustrate this concern:**

**-“The rurality is the key attraction of tourism” -This claim seems overly generalized, as tourism encompasses a wide range of forms and motivations. A more nuanced formulation would be advisable to reflect the diversity of tourism drivers.**

**- “Given the diversity of quantitative research on rural representation of rurality as rural is diversity, there is a need to identify the sub-dimensions or components of rurality representation”.**

**-“sheep” (line 22, page 9).**

****Reply**: Thanks for reviewer’s suggestion regarding language clarity. We have thoroughly reviewed the manuscript and revised these statements:**

(1) **“The rurality is the key attraction of tourism” has been changed to “The representational system of rurality, which serves as a fundamental attraction in rural tourism, constitutes the critical research focus of this study” to specify the scope,and cite references to provide a more detailed explanation and supplementation of this passage. Translate into English.**
(2) **We sincerely thank the reviewer for highlighting the lack of clarity in our original statement regarding the research rationale. The initial sentence suffered from grammatical issues and failed to effectively communicate our research positioning. In response to this valuable feedback, we have thoroughly revised the relevant section to present a clearer and more logical explanation of our research motivation and theoretical foundation.**

**The original problematic sentence has been replaced with the following refined statement in the Introduction section: “Guided by social representation theory, this study selects three typical rural tourism destinations to investigate the core representational content of rurality. Through in-depth interviews with both residents and tourists, the research analyzes the specific connotations of typical rurality representations. The representational system of rurality, which serves as a fundamental attraction in rural tourism, constitutes the critical research focus of this study. The representations of rurality in tourism destinations exhibits significant diversity, creating an imperative to systematically identify its sub-dimensions and representational connotations.”**

**The revision addresses the grammatical redundancy and unclear logic by: first, explicitly anchoring the research in social representation theory; second, clearly outlining the case study and interview methodology; and third, establishing a logical progression from theoretical foundation to research objectives. This creates a coherent argument that maintains our core research intent while improving academic rigor.**

**(3)Regarding “sheep”, we have clarified the context: “Urban gentrification typically emphasizes bucolic landscapes—characterized by elements such as farmland, country lanes, and pastoral scenery—alongside leisure and entertainment infrastructures including restaurants, pubs, art galleries, museums, and sports facilities (Ramazannejad, Zarghamfard, Hajisharifi, and Azar, 2021).”**

****3. Comment:**The manuscript states that “This study focuses on the two periods before and after the development of tourism to see how rurality representations altered after social change”; however, the only temporal reference provided is “June 14–15 and August 27–30, 2020.” Greater clarity is needed.**

****Reply:**We thank the reviewer for highlighting the need for greater clarity regarding our temporal framework. The original description failed to adequately explain the rationale behind our period selection and methodological approach. Our revision strategy aimed to address this by: first, explicitly justifying the selection of circa 2000 and circa 2020 as comparison periods based on the substantive phase of tourism development around 2010； second, clarifying the relationship between data collection timing (2020) and the studied temporal framework; and third, explaining our methodological approach of using retrospective narratives to reconstruct pre-tourism conditions.**

**Accordingly, we have added the following clarification in Section 3.1: “To capture the dynamic evolution process, given that rural tourism development in all three case sites entered a substantive phase around 2010, the pre- and post-tourism development periods were selected as critical temporal nodes for interviews. This enables a comparison of typical rurality representations and their transformations between the pre-development period (circa 2000) and the post-development era (circa 2020). Interview data collection was completed during field surveys conducted from June 14-15, 2020 (in Shi’ao Village) and August 27-30, 2020 (in Dashan Village and Qiqiao Village), capturing both contemporary rurality representations and retrospective narratives of the pre-tourism period. This methodological approach, which contrasts recalled past experiences with present realities, facilitates an understanding of shifts in the constitutive dimensions of rurality representation.”**

****4**. **Comment:**Furthermore, the statement on page 7 — “Rurality is not only a subjective perception but also a product of social construction” — is particularly significant. This claim would benefit from further elaboration and the inclusion of relevant scholarly references to strengthen its theoretical grounding and enhance its impact.**

****Reply:** We thank the reviewer for recognizing the significance of this theoretical statement and suggesting its further development. The original claim lacked sufficient theoretical support and contextual explanation. Our revision strategy addressed this by developing a coherent theoretical progression that: first, establishes the objective-subjective duality of rurality; second, introduces the tourism context as a crucial domain for understanding its socially constructed nature; and third, systematically elaborates the social representation processes through which rurality is continuously negotiated and reproduced.**

**Accordingly, we have expanded the original statement into a comprehensive theoretical discussion: “Rurality is not only a subjective perception but also a product of social construction. In general rural studies, it is commonly quantified using objective indicators such as population density and economic structure. However, in tourism contexts, visitors’ subjective perceptions of rurality take on critical importance (Pu, Lu, & Chen, 2022). This distinction reveals that while rurality may have an objective foundation, its meaning and significance are continually shaped through social interpretation and interaction (Wang & Liang, 2020). As rural characteristics evolve with societal development, the very essence of rurality undergoes constant redefinition. Its meaning is negotiated through social representation—a process through which shared understandings emerge among members of society. Thus, rurality functions as a dynamic system of shared meanings, continuously produced and reproduced through social interaction, discursive practice, and lived experience within specific socio-historical contexts (Lan, Kerstetter, & Hunt, 2017).”**

**This revision provides the necessary theoretical grounding while maintaining the original insight’s significance.**

****5. Comment**:It can be inferred that “agritainment” derives from a combination of “agriculture” and “entertainment”; however, the term should be clearly defined, and it should be clarified whether it has been used in previous academic literature. The term “angertainment” (page 22, line 5) appears to be a likely misspelling of “agritainment.”**

****Reply:** We appreciate the reviewer’s attention to terminology precision. We have:**

**(1)Corrected all instances of “angertainment” to “agritainment”.**

**(2)We thank the reviewer for suggesting clearer definition and academic grounding for the term “agritainment”. In response, we have explicitly defined this term upon its first appearance in Section 3.1 by incorporating an established conceptualization from the literature. The revised text now states that we selected “typical agritainment—a business model that Phillip et al. conceptualize as working farms diversifying into providing recreational, hospitality, and educational services for tourists” as one of our major research types.**

6.****Comment：**In the Methodology section, it is recommended to provide a more detailed explanation of the process through which the three core categories were distilled from the initial 28 main categories.**

****Reply：**We thank the reviewer for this valuable suggestion regarding methodological transparency. To address the need for a clearer illustration of our qualitative analysis process, we have added “Table 2. The coding process of material rurality representation” “Table 3. The coding process of social rurality presentation” “Table 4. The coding process of cultural rurality reprsentation” .These tablea systematically documents the complete analytical procedure through which we distilled the three core categories from the initial 28 main categories, following the grounded theory approach. It visually demonstrates the progression from open coding to axial and selective coding, showing how we identified logical relationships and hierarchical structures among concepts. This revision provides readers with a transparent overview of our analytical rigor and enhances the methodological credibility of our findings.**

Table 2. The coding process of material rurality representation

| **Selective coding** | **Axial coding** | | **Open coding** | |
| --- | --- | --- | --- | --- |
| Core category | Main category | Subcategory | Pre-Tourism development | Post-Tourism development |
| Material rurality representation | Natural environment | Natural scenery | Rice plant, Wheat, Rapeseed, Farmland, Vegetable plots, Bamboo | Peach blossoms, Lotus flowers, Maple leaves |
|  |  | Flora and fauna | Rapeseed flowers, Maple leaves, Eagles, Poultry, Hares, Pheasants, Woodpeckers, Cats/Dogs | Poultry, Cats/Dogs |
|  |  | Water bodies and resources | Washing clothes in the river, Washing rice in the river, Washing vegetables in the river, River water irrigation | Regularly cleaned, Ornamental |
|  |  | Climate and environment | Fresh air, Picturesque landscape (with hills and streams), Poor sanitation, Underdeveloped | Picturesque and tidy environment |
|  | Rural architecture | Settlement landscape | Derelict, Decrepit, Chaotic | Tidy, Methodical |
|  |  | Transportation facilities | Muddy, Paths | Asphalt, Cement, Pavement, Spacious |
|  |  | Architectural features | Uniform, Shabby, Disordered, Ordinary | Black and white, Ancient dwellings, Hui-style, Horse-head gables, Comfortable |
|  |  | Leisure facilities | Benches at home entrance | Small long corridors, Pavilions |

Table 3. The coding process of social rurality presentation

| **Selective coding** | **Axial coding** | | **Open coding** | |
| --- | --- | --- | --- | --- |
| Core category | Main category | Subcategory | Pre-Tourism development | Post-Tourism development |
| Social rurality presentation | Production and living | Production methods | Farming | Farmhouse cuisine, Agritainment |
|  |  | Production tools | Manual labor, Animal power | Machinery |
|  |  | Labor and livelihood | farming, Toilsome, Poultry raising | Easier, Less free |
|  |  | Pace of life | Busy farming season, Leisurely | Leisurely, Busy |
|  |  | Information technology | - | QR code payments, Mobile phones |
|  |  | Transportation means | Bicycles | Buses, Private cars |
|  |  | Consumption patterns | Rarely going out | Express delivery |
|  |  | Local cuisine | Monotonous, Limited | Healthy, Western-style |
|  |  | Local language | Dialect | Mandarin |
|  | Interpersonal communication | Neighborhood relations | Strong bonds, Mutual aid, Deep-rooted Ties | Competitive relations |
|  |  | Interaction principles | Relational exchanges | Transactional exchanges |
|  |  | Rural customs | Unspoiled and sincere, Industrious and frugal, Assiduous, Heartfelt hospitality | Unspoiled, Diligent |
|  |  | Resident quality | There are some illiterate individuals | Legal awareness, Educational attainment, Follow current affairs |

Table 4. The coding process of cultural rurality representation

| **Selective coding** | **Axial coding** | | **Open coding** | |
| --- | --- | --- | --- | --- |
| Core category | Main category | Subcategory | Pre-Tourism development | Post-Tourism development |
| Cultural rurality representation | Folk-custom and festival | Rural folklore | Lost | Horse-Lantern Dance, Opera Performances, Long-Street Banquets, Pitchfork Martial Arts Demonstration, Jinhua Festival |
|  |  | Traditional festivals | Making Zongzi, Writing couplets, Pasting couplets | Consumption-oriented |
|  |  | Recreation and leisure | Lacking | Square Dancing, Strolling, Guandan (A popular chinese card game) |
|  | Residents’ spiritual orientation | Clan and ancestral halls | Disregarded | Genealogies, Cultural preservation, Inheritance |
|  |  | Spiritual beliefs | Baoping well, Wenfeng pagoda, Niushou mountain, Pagoda, Buddhist relics | Baoping Well, Wenfeng Pagoda, Niushou Mountain, Pagoda, Buddhist Relics |
|  |  | Values and outlook | Diligence, Striving for improvement | Better living, Positive, Enjoyment of life |
|  |  | Local identity | Rural poverty | Rural comfort |

7. ****Comment:**The opening lines of the Data Collection section should be clarified or reformulated, as they currently suggest that interviews with tourists were a plan B option due to an insufficient number of interviews with residents.**

****Reply:**** **We thank the reviewer for this important observation regarding the methodological description. The original phrasing inaccurately suggested that tourist interviews were a secondary option. Our revision repositions this as a deliberate methodological choice by: first, establishing the strategic selection of two stakeholder groups; second, clarifying the complementary value of each perspective (residents’ longitudinal view and tourists’ external gaze); and third, emphasizing the theoretical rationale for this dual-perspective approach in studying social representations.**

**Accordingly, we have replaced the original text with:“To comprehensively capture the representations and evolution of rurality, this study strategically engaged two key stakeholder groups: local residents and tourists. In-depth interviews with residents provided a longitudinal perspective on the changes in rurality, given their direct lived experiences over time. Complementing this, interviews with tourists were conducted to capture the external gaze and immediate perceptions that shape the consumption of rurality. This dual-perspective approach ensures that the social construction of rurality is examined from both the insider (resident) and outsider (tourist) viewpoints, thereby offering a more holistic understanding. It is noteworthy that a significant portion of the tourists were repeat visitors from nearby urban areas, which enhanced their familiarity with the rural destinations and the depth of their insights.” This revision clearly presents our research design as an intentional strategy rather than a contingency plan.**

*****reviewer 2：*****

1.****Comment:**In terms of the results report, the author presents the representation of rurality and its change in rural tourism destinations based on the Social Representation Theory. However, the authors did not strictly follow the procedures of the Grounded Theory analysis paradigm to present the outcomes of open coding, axial coding, and selective coding.**

****Reply:** We thank the reviewer for this valuable suggestion regarding methodological rigor. To address the need for greater transparency in our analytical process, we have added “Tab. 2: The coding process of rurality in rural tourism destination” at the beginning of the Results section.**

**This table systematically documents the complete analytical procedure through which we distilled the three core categories from the initial 28 main categories, following the grounded theory approach. It visually demonstrates the progression from open coding through axial coding to selective coding, clearly showing how we identified logical relationships and hierarchical structures among concepts.**

**This addition provides readers with a transparent overview of our analytical rigor and enhances the methodological credibility of our findings by making the coding process explicit and traceable.**

2.****Comment:****.**Also, the results are superficial and descriptive, without in-depth analysis and synthesis. The presentation of the results can be enhanced by including visual representations such as graphs to summarise key points and ensuring that the analysis goes beyond descriptive findings to provide in depth insights and synthesis. Suggest making appropriate modifications.**

****Reply:**We thank the reviewer for this constructive suggestion regarding the presentation of our grounded theory analysis. To better demonstrate our analytical procedure, we have added “Figure 3: The Dimensional Structure and Evolutionary Trajectory of Rurality Representation” at the begining of the Results section.**

Fig3. The dimensional structure and evolutionary trajectory of rurality representation

**This figure, grounded in Social Representation Theory and structured through the analytical logic of open coding, axial coding, and selective coding, systematically delineates the three core dimensions of rurality representation (material, social, and spiritual) in rural tourism destinations along with their evolutionary trajectories. It visually demonstrates the transformation process of rurality representations from the pre-tourism to post-tourism periods. Through this graphical representation, we have further reinforced the grounded theory analytical approach adopted in this study, making both the coding structure and evolutionary trends more intuitive and systematic for readers.**

**Meanwhile, the author has revised the Results section by adding summary and synthesis paragraphs in the penultimate paragraph of each of the six sub-dimensions. These revisions are intended to move beyond purely descriptive findings, offering deeper insights and a more comprehensive analytical synthesis.**

**To address the need for a clearer illustration of our qualitative analysis process, we have added “Table 2. The coding process of material rurality representation” “Table 3. The coding process of social rurality presentation” “Table 4. The coding process of cultural rurality reprsentation” .These tablea systematically documents the complete analytical procedure through which we distilled the three core categories from the initial 28 main categories, following the grounded theory approach. It visually demonstrates the progression from open coding to axial and selective coding, showing how we identified logical relationships and hierarchical structures among concepts. This revision provides readers with a transparent overview of our analytical rigor and enhances the methodological credibility of our findings.**

Table 2. The coding process of material rurality representation

| **Selective coding** | **Axial coding** | | **Open coding** | |
| --- | --- | --- | --- | --- |
| Core category | Main category | Subcategory | Pre-Tourism development | Post-Tourism development |
| Material rurality representation | Natural environment | Natural scenery | Rice plant, Wheat, Rapeseed, Farmland, Vegetable plots, Bamboo | Peach blossoms, Lotus flowers, Maple leaves |
|  |  | Flora and fauna | Rapeseed flowers, Maple leaves, Eagles, Poultry, Hares, Pheasants, Woodpeckers, Cats/Dogs | Poultry, Cats/Dogs |
|  |  | Water bodies and resources | Washing clothes in the river, Washing rice in the river, Washing vegetables in the river, River water irrigation | Regularly cleaned, Ornamental |
|  |  | Climate and environment | Fresh air, Picturesque landscape (with hills and streams), Poor sanitation, Underdeveloped | Picturesque and tidy environment |
|  | Rural architecture | Settlement landscape | Derelict, Decrepit, Chaotic | Tidy, Methodical |
|  |  | Transportation facilities | Muddy, Paths | Asphalt, Cement, Pavement, Spacious |
|  |  | Architectural features | Uniform, Shabby, Disordered, Ordinary | Black and white, Ancient dwellings, Hui-style, Horse-head gables, Comfortable |
|  |  | Leisure facilities | Benches at home entrance | Small long corridors, Pavilions |

Table 3. The coding process of social rurality presentation

| **Selective coding** | **Axial coding** | | **Open coding** | |
| --- | --- | --- | --- | --- |
| Core category | Main category | Subcategory | Pre-Tourism development | Post-Tourism development |
| Social rurality presentation | Production and living | Production methods | Farming | Farmhouse cuisine, Agritainment |
|  |  | Production tools | Manual labor, Animal power | Machinery |
|  |  | Labor and livelihood | farming, Toilsome, Poultry raising | Easier, Less free |
|  |  | Pace of life | Busy farming season, Leisurely | Leisurely, Busy |
|  |  | Information technology | - | QR code payments, Mobile phones |
|  |  | Transportation means | Bicycles | Buses, Private cars |
|  |  | Consumption patterns | Rarely going out | Express delivery |
|  |  | Local cuisine | Monotonous, Limited | Healthy, Western-style |
|  |  | Local language | Dialect | Mandarin |
|  | Interpersonal communication | Neighborhood relations | Strong bonds, Mutual aid, Deep-rooted Ties | Competitive relations |
|  |  | Interaction principles | Relational exchanges | Transactional exchanges |
|  |  | Rural customs | Unspoiled and sincere, Industrious and frugal, Assiduous, Heartfelt hospitality | Unspoiled, Diligent |
|  |  | Resident quality | There are some illiterate individuals | Legal awareness, Educational attainment, Follow current affairs |

Table 4. The coding process of cultural rurality representation

| **Selective coding** | **Axial coding** | | **Open coding** | |
| --- | --- | --- | --- | --- |
| Core category | Main category | Subcategory | Pre-Tourism development | Post-Tourism development |
| Cultural rurality representation | Folk-custom and festival | Rural folklore | Lost | Horse-Lantern Dance, Opera Performances, Long-Street Banquets, Pitchfork Martial Arts Demonstration, Jinhua Festival |
|  |  | Traditional festivals | Making Zongzi, Writing couplets, Pasting couplets | Consumption-oriented |
|  |  | Recreation and leisure | Lacking | Square Dancing, Strolling, Guandan (A popular chinese card game) |
|  | Residents’ spiritual orientation | Clan and ancestral halls | Disregarded | Genealogies, Cultural preservation, Inheritance |
|  |  | Spiritual beliefs | Baoping well, Wenfeng pagoda, Niushou mountain, Pagoda, Buddhist relics | Baoping Well, Wenfeng Pagoda, Niushou Mountain, Pagoda, Buddhist Relics |
|  |  | Values and outlook | Diligence, Striving for improvement | Better living, Positive, Enjoyment of life |
|  |  | Local identity | Rural poverty | Rural comfort |

3.****Comment:**The third conclusion presents the differences in the perception of rurality between tourists and residents. However, the author does not strictly distinguish between tourists and residents in the result analysis section, leading to insufficient evidential support. Suggest making appropriate modifications.**

****Reply:**In the analysis section, the author clearly distinguishes between tourists and residents. The initial part of each subsection presents interview data from local residents, the second-to-last paragraph summarizes the residents’ descriptions, and the final paragraph focuses on tourists’ perceptions. However, the discussion of tourists was not systematically developed and mainly served as illustrative examples. Therefore, in revising the six sub-dimensions, the author has added extended discussions in the final paragraphs to enhance analytical depth.**

****4.Comment:**Also, it is suggested that the authors provide explanations for some rural customary activities mentioned in the results section, such as Five Fierce Deities, guandan game, Long Street Banquet and so on.**

****Reply：**We thank the reviewer for the suggestion to enhance cultural context. We have added concise explanations for these culturally specific terms in Section 4.3.1:**

**（1）“Five Fierce Deities”(a vibrant performance rooted in folk beliefs for warding off evil spirits)**

**（2）“guandan”((a hugely popular card game in Jiangsu and Anhui provinces)**

**（3）“Long Street Banquet” (a community feast where tables are connected along a long street, symbolizing unity and prosperity)**

****5. Comment:**Suggest supplementing relevant literature appropriately.
TANG Chengcai, LIU Yaru, WAN Ziwei, LIANG Wenqi. Evaluation system and influencing paths of the integration of culture and tourism of traditional villages. Journal of Geographical Sciences, 2023, 33(12): 2489-2510.
TANG Chengcai, Yang Yuanyuan, LIU Yaru, Xiao Xiaoyue. Comprehensive Evaluation of the Cultural Inheritance Level of Tourism-oriented Traditional Villages: The Example of Beijing. Tourism Management Perspectives, 2023, 48, 101166.
Tang Chengcai, Han Ying, Pin Ng. Green consumption intention and behavior of tourists in urban and rural destinations. Journal of Environmental Planning and Management, 2023, 66(10): 2126-2150.
Liu Yaru, Tang Chengcai, Wan Ziwei. Multi-scenario analysis and the construction of the revitalization model of green development in tourism traditional villages. Journal of Resources and Ecology, 2023, 14(2): 239-251.
Chengcai Tang, Qianqian Zheng, Pin Ng. A Study on the Coordinative Green Development of Tourist Experience and Commercialization of Tourism at Cultural Heritage Sites. Sustainability,2019, 11, 4732.**

****Reply：** We thank the reviewer for the valuable suggestion to strengthen our literature foundation. In response, we have supplemented relevant contemporary references at key points in our manuscript to better situate our research within current scholarly conversations. Specifically, we have incorporated “Tang et al. (2023)” in our discussion of folk activity revival through tourism, added “Campón-Cerro et al. (2017)” and “Tang et al. (2023)” to support our analysis of region-specific rurality representation, and included “Tang et al. (2023)” when examining the profound impact of the development of social productive forces on traditional rural lifestyles. These strategic additions enhance the theoretical grounding of our arguments while maintaining the original structure and analytical flow of our study, ultimately strengthening the scholarly contribution of our work.**
