# Supplementary material for: Rurality representation and changes in rural tourism destination
Source: PLoS One. 2026 Apr 21;21(4):e0347226. doi: 10.1371/journal.pone.0347226 (PMC13098982; doi:10.1371/journal.pone.0347226)
Supplement: Attachment — Submitted filename: Response_to_Reviewers_auresp_2.docx [file pone.0347226.s004.docx]

Response letter for the Qianda Zhuang, Ph.D. Guest Editor and peer reviewers

Re: Manuscript ID: PONE-D-25-26199

Title: Rurality Representation and Changes in Rural Tourism Destination

Dear Editors and Reviewers,

We would like to express our sincere appreciation to the editors and reviewers for their time and invaluable suggestions, which have significantly enhanced the quality of our paper. In response to all the points raised, we have undertaken comprehensive revisions to the manuscript. Detailed point-by-point responses follow. To clearly indicate the changes, all substantive modifications have been color-highlighted.

We greatly appreciate your time and consideration.

*****reviewer 1*****

**1.**Comment**：The observations from the first phase of the review have been addressed. By also responding to the comments of the other reviewer, the paper now appears more solid.However, the paper still needs more careful English editing. Here is just one example: “pre-development period (circa 2000) and the post-development era (circa 2020).” Since the exact dates of data collection are provided later in the text, the formulations “circa 2000” and “circa 2020” are not the most appropriate choices.**

****Reply**:**

**Thank you very much for your insightful suggestion regarding the linguistic precision of our manuscript. We completely agree that the phrasing regarding the time periods needed refinement to avoid ambiguity, especially since the exact data collection dates are specified later in the text.**

**As advised, we have re-evaluated the description of our temporal sampling strategy. We have moved away from the imprecise term “circa” and have instead anchored the timeline more rigorously to the known inflection point of tourism development (2010). By conceptualizing 2010 as a "temporal pivot" and applying a symmetrical ten-year interval, we have established 2000 and 2020 as fixed reference points for the "recalled past" and the "observed present," respectively.**

**The revised text now reads as follows (changes have been implemented in Section 3.1):Since substantive rural tourism development in all three sites began around 2010, this year was used as a temporal pivot to examine the evolution of rurality representations. By extending ten years in both directions, the year 2000 and the year 2020 were established as critical reference points for interviews—representing the pre-development period and the post-development period, respectively. This framework enables a comparison of typical rurality representations and their transformations between the recalled past and the observed present. Interview data were collected during field surveys conducted from June 14–15, 2020 (Shi’ao Village) and August 27–30, 2020 (Dashan and Qiqiao Villages). These interviews captured contemporary representations of rurality alongside retrospective narratives of the pre-tourism era. By contrasting recalled past experiences with current realities, this methodological approach illuminates changes in the constitutive dimensions of rurality representations.**

**Morever，we sincerely thank the reviewer for pointing out the need for more careful English editing. In response, we have engaged the professional language editing service Editage to conduct a comprehensive revision of the manuscript’s language, focusing on grammar, phrasing, clarity, and overall academic tone. The editing certificate is attached to this resubmission as supporting documentation.**

**We have carefully reviewed and incorporated all suggested changes, which we believe have substantially improved the readability and precision of the paper. Should any further adjustments be required, we remain open to additional refinements.**
